# eFluorination for the Rapid Synthesis of Carbamoyl Fluorides from Oxamic Acids

**DOI:** 10.1021/acs.orglett.4c01605

**Published:** 2024-07-17

**Authors:** Feba Pulikkottil, John S. Burnett, Jérémy Saiter, Charles A. I. Goodall, Bini Claringbold, Kevin Lam

**Affiliations:** School of Science, Faculty of Engineering and Science, University of Greenwich, Chatham Maritime, Chatham, Kent ME4 4TB, United Kingdom

## Abstract

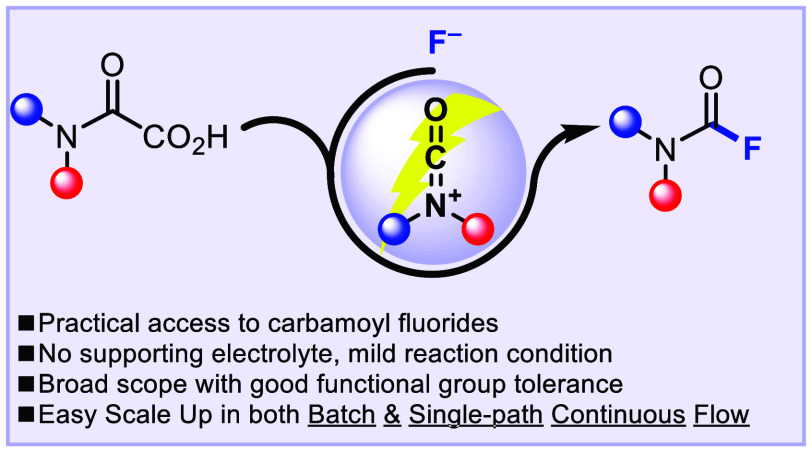

In this letter, we disclose the anodic oxidation of oxamic acids in the presence of Et_3_N·3HF as a practical, scalable, and robust method to rapidly access carbamoyl fluorides from readily available and stable precursors. The simplicity of this method also led us to develop the first flow electrochemical preparation of carbamoyl fluorides, demonstrating scale-up feasibility as a proof of concept.

## Introduction

**Figure 1:**
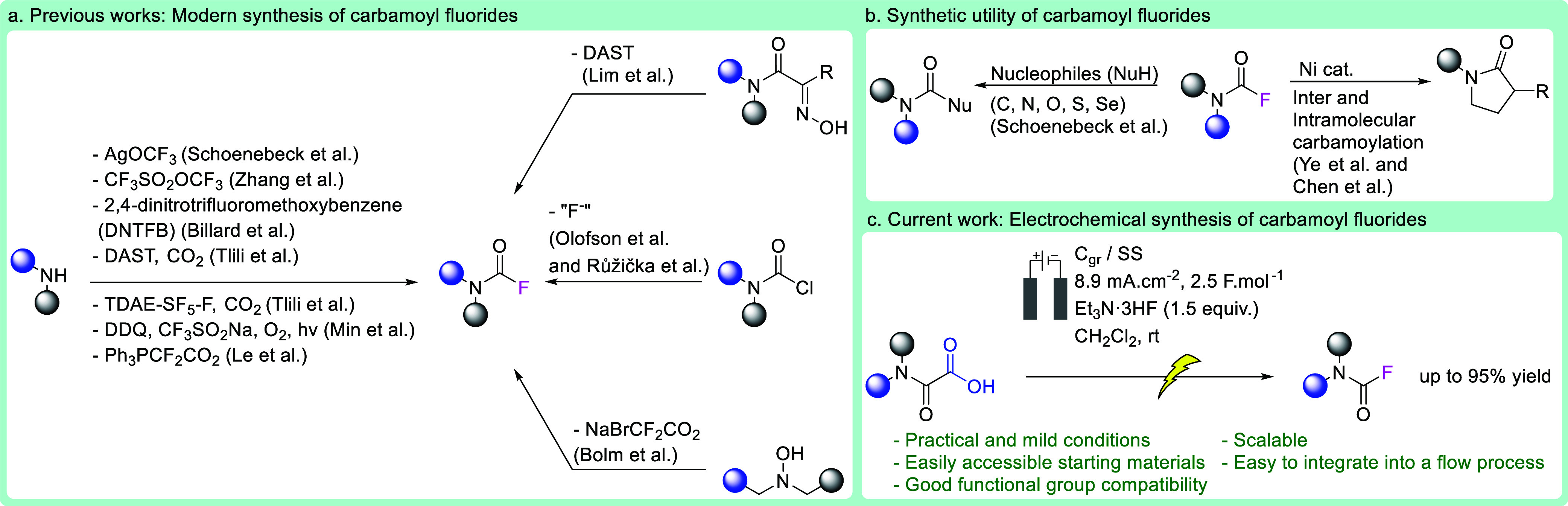
Syntheses and uses of carbamoyl fluorides.

Fluorine has long held a special place in many areas of chemistry as a result of its ability to impart or enhance remarkable physical and chemical properties to a molecule when present in its framework.^1−7^ Carbamoyl fluorides have attracted much attention for their use as insecticides and esterase inhibitors.^8−10^ In addition, because carbamoyl fluorides exhibit greater stability and selectivity than carbamoyl chlorides, they represent exceptional building blocks in the synthesis of hydrazines,^11^ isocyanates,^12^ carbamates, thiocarbamates, ureas,^13^ and amides.^8−10,13,14^ Unfortunately, their synthesis can remain a challenge (Figure 1).^15,16^ The primary preparation method is to treat a carbamoyl chloride with nucleophilic sources of fluoride.^17,18^ While this method seems straightforward, it still requires the preparation of the highly reactive and often unstable chloride analogue, which is usually produced using expensive and highly toxic phosgene derivatives.^19,20^ The past decade has seen a rapid increase in the development of novel methods for preparing carbamoyl fluorides, confirming the growing interest of the synthetic community in this fluorinated motif.^21−24^ However, most use impractical conditions, often combined with highly air- and water-sensitive, toxic, hazardous, and expensive reagents, such as carbonyl difluoride,^13,25−28^ carbon disulfide,^29^ highly reactive and unstable carbamoyl chlorides,^28^ or explosive diethylaminosulfur trifluoride (DAST)-type reagents.^30,31^ Some other methods need expensive Ag salts,^32^ significant excess of reagents leading to time-consuming chromatographic purifications,^33,34^ or high temperatures.^35^ Although some early work has yielded the desired products in single-step reactions, using DAST and silver salts presents a significant challenge to scale up the reaction, especially under process-friendly conditions.^13,25−28^ There is an urgent need to develop a novel, practical, sustainable, inexpensive, and milder way to access carbamoyl fluorides rapidly. Herein, we disclose an unprecedented practical and robust anodic synthesis of carbamoyl fluorides from readily available stable oxamic acids in the presence of a fluoride salt.

## Results and Discussion

### Optimization

On the basis of our previous experience with the anodic oxidation of oxamic acids,^36^ we started our investigations with electrolysis substrate **1a** in CH_2_Cl_2_ using 2 equiv of the mild and less corrosive fluorinating reagent Et_3_N·3HF,^37,38^ at a current density of 8.9 mA cm^–2^. We used carbon graphite (C_gr_) as the anode as a result of its low cost and ability to perform multiple electron transfers. For the cathode, we chose a platinum foil electrode, known to have a low hydrogen overpotential, favoring the benign reduction of protons. To our delight, desired carbamoyl fluoride **2a** was obtained with a yield of 95% under these conditions. The reaction parameters were then further investigated (Table 1).

**Table 1:**
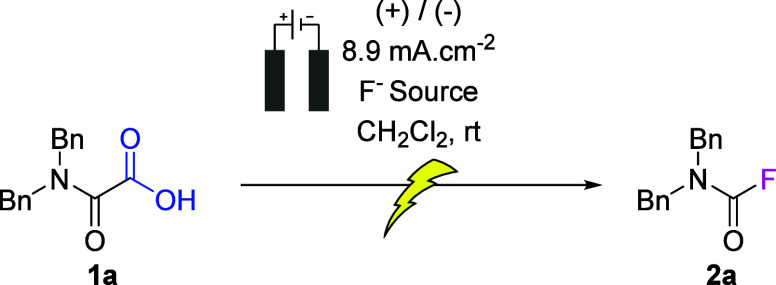
Optimization Results^a^

entry	+	–	F^–^ source	equiv	F mol^–1^	yield (%)^b^
1	C_gr_	Pt	Et_3_N·3HF	2	3	95
2	C_gr_	C_gr_	Et_3_N·3HF	2	2.5	89
3	C_gr_	Ni	Et_3_N·3HF	2	2	94
4	C_gr_	SS	Et_3_N·3HF	2	2.5	96
5^c^	C_gr_	SS	Et_3_N·3HF	2	2	60
6^d^	Pt	SS	Et_3_N·3HF	2	2	0
7^c^	C_gr_	Pt	CsF	2	2.5	32
8^c^	C_gr_	Pt	KF and 18-Crown-6	5.5	4	67
9	C_gr_	Pt	Bu_4_N·H_2_F_3_	1	3	97
10	C_gr_	SS	TMAF	1.5	2.5	
11^b^	C_gr_	SS	TMAF	1.5	2.5	19
12^e^	C_gr_	SS	Et_3_N·3HF	1.5	2.5	96
13^f^	C_gr_	SS	Et_3_N·3HF	1.5	2	93
14	C_gr_	Pt	Et_3_N·3HF	3	3	69
15	C_gr_	Pt	Et_3_N·3HF	2	3.5	92
16	C_gr_	SS	Et_3_N·3HF	1	3	82

a Conditions: Unless otherwise stated, the solvent is CH_2_Cl_2_ with 0.4 mmol of oxamic acid and current density = 8.9 mA cm^–2^.

b ^1^H nuclear magnetic resonance (NMR) yield was calculated from the NMR ratio product and bromoform used as an internal standard, with MeCN as the solvent.

c MeCN as the solvent.

d No conversion of starting material was observed.

e Current density = 4.45 mA cm^–2^.

f Current density = 13.35 mA cm^–2^.

Each optimization experiment was followed by high-performance liquid chromatography–mass spectrometry (HPLC–MS) until complete consumption of starting oxamic acid, which in most cases occurred after 2.5–3.0 F mol^–1^, revealing a highly efficient anodic oxidation with average faradaic yields ranging from 66 to 80%. As expected, the electrode material proved to be an important factor (entries 1–4 for the cathode and entries 5 and 6 for the anode). While the use of carbon graphite as a cathode, which has a higher hydrogen overpotential, did not affect the yield of the reaction (entry 2), the use of less noble metals with low hydrogen overpotentials, such as Ni and stainless steel, not only performed as well as a platinum cathode but also gave very clean transformations without significant byproducts (entries 3 and 4). Therefore, cheap and readily available stainless steel was chosen as the cathodic material. Carbon graphite was superior to platinum for the anode (entries 5 and 6). In terms of solvents, CH_2_Cl_2_ was shown to be better than MeCN (entries 5, 7, and 8), probably as a result of the formation of tighter ion pairs, which facilitate the addition of fluoride. With regard to the nature of the fluoride source (entries 6–9), surprisingly, even weaker nucleophilic fluorides, such as KF/18-Crown-6 or CsF, led to the formation of desired carbamoyl fluoride, albeit in modest yields along with numerous byproducts. While Bu_4_N·H_2_F_3_ was found to be as efficient as Et_3_N·3HF in achieving the desired fluorination (entry 9), the tetrabutylammonium salt led to the formation of significant amounts of tributylamine via cathodic Hofmann elimination, contaminating final carbamoyl fluoride. Interestingly, the use of tetramethylammonium fluoride (TMAF), a better nucleophilic fluoride donor than Et_3_N·3HF as a result of the lack of hydrogen bonding, did not result in any improvement.

TMAF is poorly soluble in CH_2_Cl_2_ and did not provide the necessary conductivity for the electrolysis to proceed (entry 10). At the same time, only 19% yield was obtained in MeCN, together with many unidentified byproducts (entry 11). The effect of current density on the course of the reaction was also investigated. On the one hand, a lower current density of 4.45 mA cm^–2^ led to similarly excellent yields (entry 12) but surprisingly required a longer electrolysis time as a result of a lower faradaic efficiency. On the other hand, a higher current density of 13.35 mA cm^–2^ resulted in lower yields (entry 13). Using a significant excess of Et_3_N·3HF was detrimental to the reaction (entry 14). Finally, the ideal amount of Et_3_N·3HF was found to be 1.5 equiv (entries 15 and 16).

### Substrate Scope

**Scheme 1:**
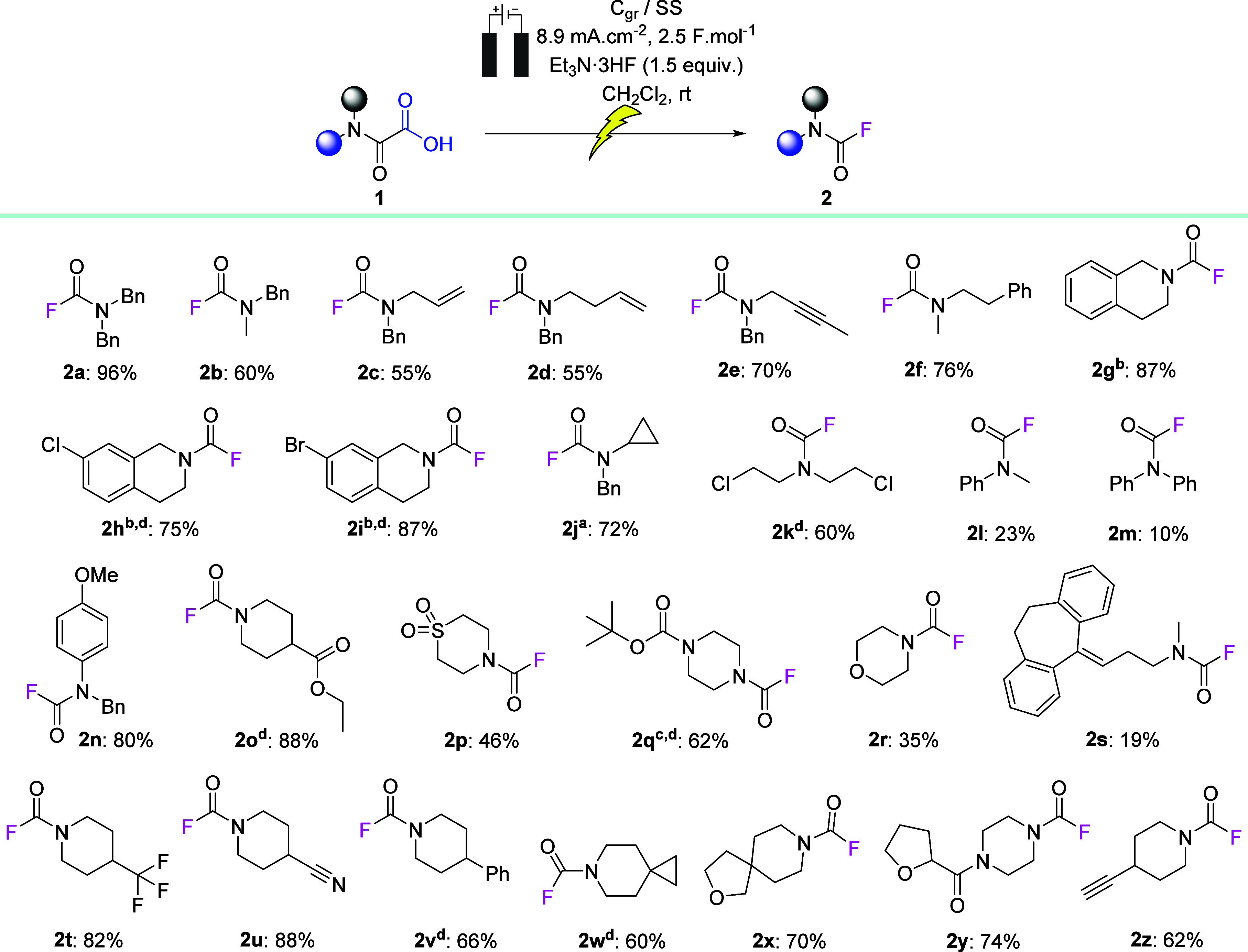
Substrate Scope Obtained using a 13.35 mA cm^–2^ current density. Obtained using 2 equiv of Et_3_N·3HF. Obtained using 3 F mol^–1^. Purified through a silica pad. Conditions: oxamic acid (0.4 mmol), Et_3_N·3HF (0.6 mmol, 1.5 equiv), CH_2_Cl_2_ (5 mL), electrodes: carbon graphite (anode)/stainless-steel (cathode), 8.9 mA cm^–2^, and 2.5 F mol^–1^.

**Figure 2:**
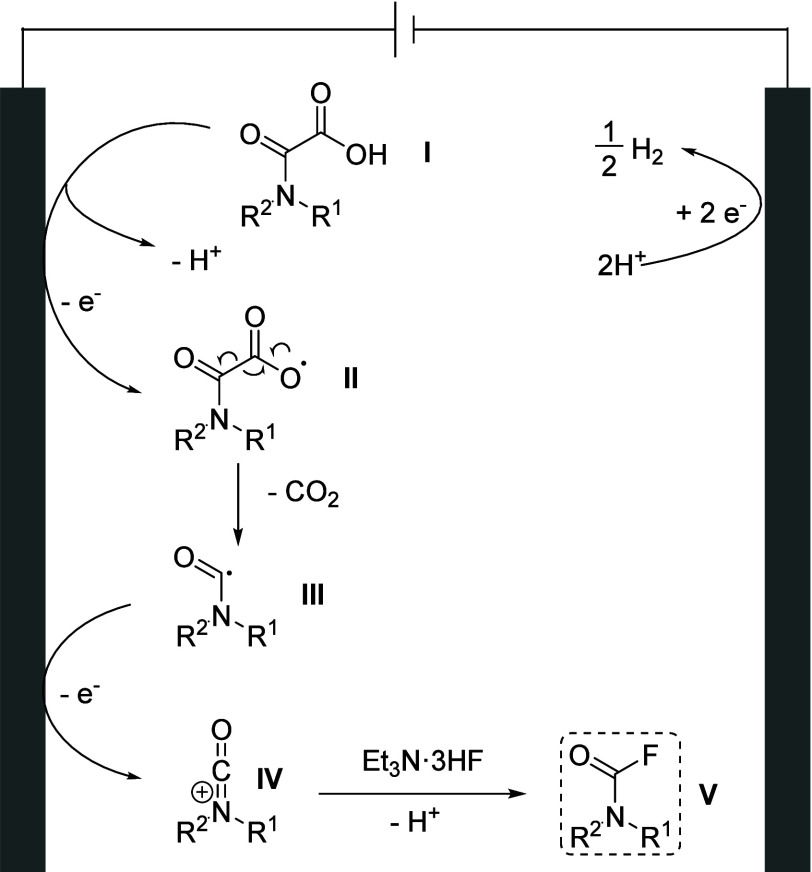
Proposed reaction mechanism.

With the optimal reaction conditions in hand, the scope and limitations of the novel fluorination reaction were investigated, focusing on motifs that may be relevant to medicinal chemistry applications. The main results are summarized in Scheme 1. The oxamic acid precursors were all readily prepared from corresponding secondary amine, often without the need for chromatographic purification (see the Supporting Information). Notably, the reaction is compatible with a wide range of functional groups, including alkenes (**2c** and **2d**) and internal and terminal alkynes (**2e** and **2z**), in acceptable yields. Unfortunately, highly activated and redox-active alkenes, such as the nortriptyline drug derivative **1s**, gave only modest yields of the desired fluorinated compound **2s** with unidentified byproducts. Electrolysis of the biologically active tetrahydroisoquinoline–oxamic derivatives gave excellent yields of up to 87% of carbamoyl fluorides (**2g**–**2i**) highlighting the efficency of the method.^27,39−41^ The novel methodology was shown to be compatible with both aliphatic and aromatic halides (**2h**, **2i**, and **2k**), providing the desired products in 60–87% yields. Spiro-type compounds, used in medicinal chemistry for their three-dimensional (3D) properties, were compatible with the electrolytic conditions, providing compounds **2w** and **2x** in 60 and 70% yields, respectively.^42^ Furthermore, the successful preparation of compound **2j** in 72% yield confirms the compatibility of cyclopropanes, which are redox-active moieties, with the electrolytic conditions. Numerous other functional groups, such as esters (**2o**),^34^ sulfones (**2p**), tetrahydropyrans (THPs, **2r**), trifluoromethyls (**2t**), nitriles (**2u**), and amides (**2y**), were compatible and led to the formation of functionalized carbamoyl fluorides in average to excellent yields. Interestingly, Et_3_N·3HF is close to neutral and, therefore, did not deprotect the Boc carbamates,^38^ allowing for the formation of compound **2q** in 62% yield. Lower yields of carbamoyl fluorides were obtained with aniline derivatives. For example, **2l** and **2m** were obtained in only 23 and 10%, respectively. However, when an electron-rich methoxy group was present on the aromatic ring, compound **2n** was obtained in 80%, highlighting the importance of the availability of the nitrogen lone pair in the formation of the N-centered cation (**IV**; Figure 2). For all reported transformations, the pure product was obtained in most cases without chromatographic purification.

On the rare occasions when the product required further purification, rapid filtration through a silica pad proved sufficient to avoid the need for a detrimental flash chromatographic separation. In fact, we found that purification by standard column chromatography methods to obtain pure carbamoyl fluoride dramatically reduced its isolated yield.

### Scale-up of the Anodic Fluorination

**Scheme 2:**
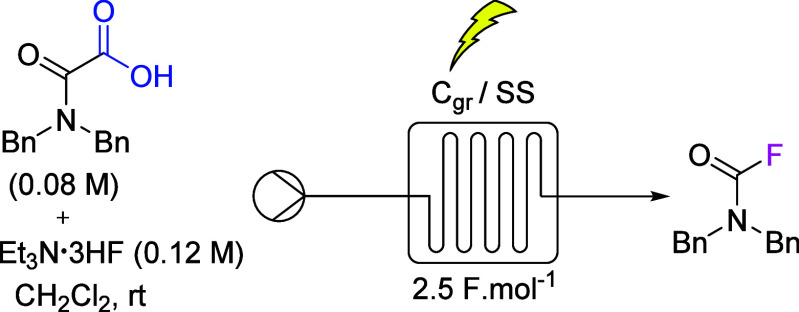
Flow Chemistry Process

As mentioned in the Introduction, to our knowledge, the large-scale synthesis of carbamoyl fluorides remains a challenge. Therefore, as a proof of concept, we investigated whether the developed anodic oxidation could be transferred to flow electrochemistry to establish a continuous manufacturing process of carbamoyl fluoride using mild, safe, and inexpensive reagents (Scheme 2). Flow chemistry has proven to be a particularly successful method for rapidly scaling up electro-organic reactions.^43^ The smaller interelectrode gap in a flow system compared to a batch process allows for a lower ohmic drop and better mass transfer, which usually leads to better performance. Focusing on the fluorination of compound **1a**, we started our flow optimization study (Table 2) using similar conditions (i.e., a current density of 8.9 mA cm^–2^) to the batch reactions, resulting in the use of a flow rate of 0.5 mL min^–1^ to achieve the transfer of 2.5 F mol^–1^. Simply pumping a mixture of starting material and Et_3_N·3HF in CH_2_Cl_2_ into the electrochemical cell yielded 80% of desired carbamoyl fluoride (entry 1). Decreasing the current density to 5.6 mA cm^–2^ and using a 0.31 mL min^–1^ flow rate led to an identical result (entry 2). Additional decreases of the current density to 2.8 and 1.4 mA cm^–2^ (with flow rates of 9.28 and 18.56 mL min^–1^, respectively) provided an increased yield of 95% (entries 3 and 4). Notably, these conditions allowed for a greater amount of product to be produced in a shorter time than in the previous batch process (Table 2), as shown by the respective space–time yields of the processes: 60 g h^–1^ L^–1^ versus 11 g h^–1^ L^–1^, providing further evidence of the benefits of flow chemistry. However, it should be noted that a lower current density would also induce a lower flow rate and a longer residence time, thus suppressing one of the benefits of this flow process.

**Table 2:** Flow Process Results

entry	current density (mA cm^–2^)	pressure (bar)	flow rate (mL min^–1^)	*t*_R_ (*t*_collect_ for 5 mL) (min)	yield (%)
1	8.9	1	0.5	2.97	80
2	5.6	1	0.31	4.79 (16.12)	80
3	2.8	4.5	0.16	9.28 (31.25)	95
4	1.4	4.5	0.08	18.56 (62.5)	95

Finally, we performed the anodic oxidation on a 1.0 g scale to show that our fluorination could withstand the change in scale without adverse effects (see the Supporting Information). As a result of the slower conversion observed on a large scale, the current density of the reaction was increased to 13.4 mA cm^–2^, and pure carbamoyl fluoride was obtained in 98% yield after passing 3.5 F mol^–1^, demonstrating that scale-up in batch and flow processes is feasible.

### Plausible Mechanism

On the basis of our previous work,^36,44^ a plausible mechanism for the electrochemical transformation is shown in Figure 2. Cyclic voltammetric experiments have confirmed that a chemically irreversible EC-type (electrochemical event followed by a chemical event) anodic oxidation of oxamic acid (**I**) occurs at *E*_pa_= 1.54 V versus Fc^+^/Fc in CH_2_Cl_2_ (see Figure S1 of the Supporting Information), leading to the formation of the unstable carboxyl radical (**II**), which rapidly loses carbon dioxide to give the acyl radical (**III**). A rapid second electron transfer then occurs to form the highly electrophilic cationic isocyanate derivative (**IV**), which is finally captured by nucleophilic fluoride from Et_3_N·3HF, leading to the formation of desired carbamoyl fluoride (**V**).

## Conclusion

In conclusion, we have developed a novel mild, practical, robust, and safe electrochemical synthesis of carbamoyl fluorides from oxamic acids using Et_3_N·3HF as both an inexpensive nucleophilic fluoride source and supporting electrolyte. The reaction can be carried out at room temperature with high faradaic efficiency using solvents straight from the bottle under non-strictly anhydrous and degassed conditions. In addition, the complete synthetic sequence starting from the amine can be achieved in most cases without chromatographic purification. Finally, taking advantage of the simplicity of our method, we have also demonstrated, as a proof of concept, the feasibility of scale-up both in batch and by transferring the methodology to flow electrochemistry. Further studies on the electrochemical synthesis of potential derivatives of carbamoyl fluorides are currently underway in our laboratory.

## Data Availability

The data underlying this study are available in the published article and its Supporting Information.

## Supporting Information Available

Optimization tables, synthetic procedures, and characterization data (PDF)
