# Supplementary material for: eFluorination for the Rapid Synthesis of Carbamoyl Fluorides from Oxamic Acids
Source: Org Lett. 2024 Jul 17;26(29):6103–8. doi: 10.1021/acs.orglett.4c01605 (PMC11287745; doi:10.1021/acs.orglett.4c01605)
Supplement: Supplementary file 1 — ol4c01605_si_001.pdf [file ol4c01605_si_001.pdf]

Supporting Information

eFluorination for the Rapid Synthesis of Carbamoyl Fluorides from Oxamic Acids.

Feba Pulikkottil,[a] John Burnett,[a] Jérémy Saiter,[a] Charles A. I. Goodall,[a] Bini Claringbold[a] and Kevin Lam*[a]
[a]School of Science, Faculty of Engineering and Science, University of Greenwich, Chatham Maritime, Chatham, Kent, ME4 4TB, U.K.
*K.Lam@greenwich.ac.uk

Contents

Material and methods ... 2
General Experimental Procedures ... 2
Batch and Flow Electrochemical Reaction Setup ... 3
Table 1: Optimisation table ... 5
Synthesis of secondary amines ... 6
Synthesis of oxamic acids ... 8
Electrochemical preparation of carbamoyl fluorides ... 20
Cyclic Voltammetry Studies ... 33
NMR Spectra ... 34
Bibliography ... 112

Material and methods

General Experimental Procedures

All reactions were carried out under aerobic conditions unless otherwise stated. All solvents and commercially available reagents were purchased from standard vendors and used without further purification unless otherwise stated. Electrolyses were performed using an IKA Electrasyn 2.0 using carbon graphite (**C_{gr}**) working electrode (**WE**) and stainless-steel (**SS**) counter electrode (**CE**) (distance between the electrodes = 6 mm) using a variable stirring rate between 600 - 1500 rpm with a stir bar of size 10x3 mm. Analytical thin-layer chromatography (TLC) was performed using silica gel plates (0.25 mm thickness) on aluminium support. Visualization was accomplished by irradiation with a UV lamp and/or staining with either $KMnO_4$ or ninhydrin. Column chromatography was performed over Silica gel 60 Å (40-63µ mesh) using a CombiFlash Rf Lumen automatic flash chromatography system. Residual solvent was removed using a static oil pump (< 10 mbar). The cooling of reaction mixtures was achieved using an ice bath (0 °C).

NMR spectra were obtained using a JEOL ECZR 400 (^{1}H 399.78 MHz; ^{19}F 376.17 MHz; ^{13}C 100.53 MHz) or ECA 500 (^{1}H 500.16 MHz; ^{13}C 125.77 MHz) spectrometer and are reported relative to the residual solvent resonances. All heteronuclear NMR spectra were ^{1}H decoupled and recorded at room temperature unless otherwise stated. Data for ^{1}H NMR spectra are reported as follows: chemical shift (δ, ppm), coupling constant (Hz), multiplicity (s, singlet; d, doublet; t, triplet; m, multiplet; br, broad) and integration. Data for ^{13}C and ^{19}F NMR are reported in terms of chemical shift (δ, ppm). IR spectra were recorded on a Perkin Elmer Spectrum Two instrument as neat samples.

High Resolution Mass Spectrometry (HRMS) data were obtained by Dr. Iain Goodall and Bini Claringbold of the University of Greenwich Mass Spectrometry Service using a Waters Synapt G2 hybrid Quadrupole-orthogonal acceleration time-of-flight configuration (Waters, Manchester, UK) operating in Resolution Mode (M/ΔM ≥ 18,000), fitted with a Waters Acquity UPLC binary solvent chromatographic pump system. The column used was a reversed-phase Acquity BEH C18 2.1 x 50 mm, 1.7-micron bead, running a 3- minute separation with an A:B eluent mixture comprising of either deionised water with 0.1% (v:v) formic acid and acetonitrile with 0.1% (v:v) formic acid (negative mode) respectively or deionised water with 0.1% (v:v) ammonium hydroxide and acetonitrile with 0.1% (v:v) ammonium hydroxide (positive mode) respectively. Mass calibration of the instrument was performed using sodium formate cluster ions, and an orthogonal Lock-SprayTM ESI probe was used with a lock mass calibrant, leucine-enkephalin. The pseudomolecular leucineenkephalin ion at *m/z* = 554.2615 (Negative Ion Mode), and *m/z* = 556.2771 (Positive Ion Mode), was used as the internal mass correction calibrant. Additional samples were analyzed on a Thermo LTQ Orbitrap XL coupled with a heated electrospray source (HESI). The capillary temperature was set to 275 °C and a voltage of 21 V. The sheath gas and auxiliary gas flow were set to 10 and 5 L h^{-1} respectively and the source current and voltage set to 100 µA and 5 kV. A solution of analyte (0.1 mg/ml) and sodium formate (1% v/v) in acetonitrile was added by direct infusion (10 µL/min) into the mass spectrometer using a Hamilton syringe (250 µL).

Gas-Chromatography Mass Spectrometry (GC-MS) data were obtained using a Shimadzu Nexis GC-2030 gas chromatograph connected to a GCMS-QP2020 NX gas chromatograph mass spectrometer, equipped with an AOC-20i Plus auto injector. The column was a CD-5MS capillary column (30 m x 0.25 mm x 0.25 µm), with helium as the carrier gas. The sample injection volume was 1 µL, and separations run over a 5-minute period with an increasing oven temperature (gradient) between 40 – 280 °C. Results were visualised and processed using LabSolutions GCMS solution version 4.50.

High-Performance Liquid Chromatography-Mass Spectrometry (HPLC-MS) data were obtained using a Shimadzu LC-2050C 3D coupled with a Shimadzu LCMS-2020 FCV-20AH2. The column was an Ascentis

Express 90Å AQ-C18, 2.7 µm. Results were visualised and processed using LabSolutions GCMS solution version 5.114.

Cyclic voltammetry studies were carried out using an Autolab 302N potentiostat interfaced through Nova 2.1 software to a personal computer. Electrochemical measurements were performed in a glovebox under an atmosphere of dinitrogen with oxygen and water levels of less than 5 ppm at 298 K, with solvents that had been thoroughly degassed and purified by passing through an alumina-based purification system. Sample concentrations of 1.0 mM were used, alongside 0.1 M [$^nBu_4N$][PF_6] supporting electrolyte concentrations. Experiments were conducted using a standard three-electrode setup comprising of a glassy carbon disc working electrode, platinum wire counter electrode, and AgCl-coated silver wire as a pseudo-reference electrode. Potentials are reported relative to the [$FeCp_2$]$^{+/0}$ redox couple, obtained through the addition of ferrocene to the analyte solution. The glassy carbon surface of the working electrode was prepared by successive polishings with Buehler diamond paste interspersed with washings (acetone and water) and sonications and final drying under ante-chamber vacuum.

Flow chemistry experiments have been realized using an Asia® Electrochemistry Flow Chemistry System including Asia® Syringe Pump, FLUX Electrochemistry reactor with a carbon gasket electrode (WE) and a stainless-steel electrode (CE) (internal volume of the cell = 225 µL) and Pressure Controller. PTFE tubbings of 0.5 mm internal diameter have been used. Yields have been evaluated after reaching steady state.

Batch and Flow Electrochemical Reaction Setup

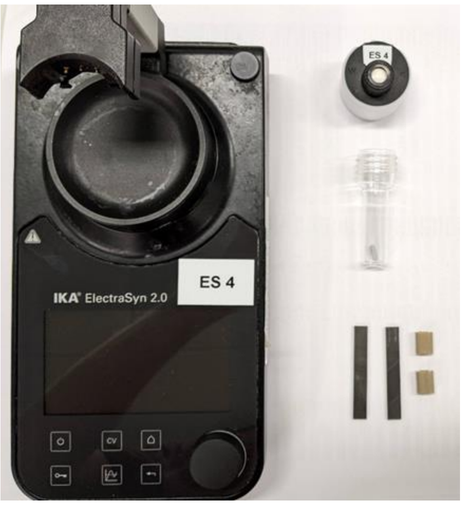

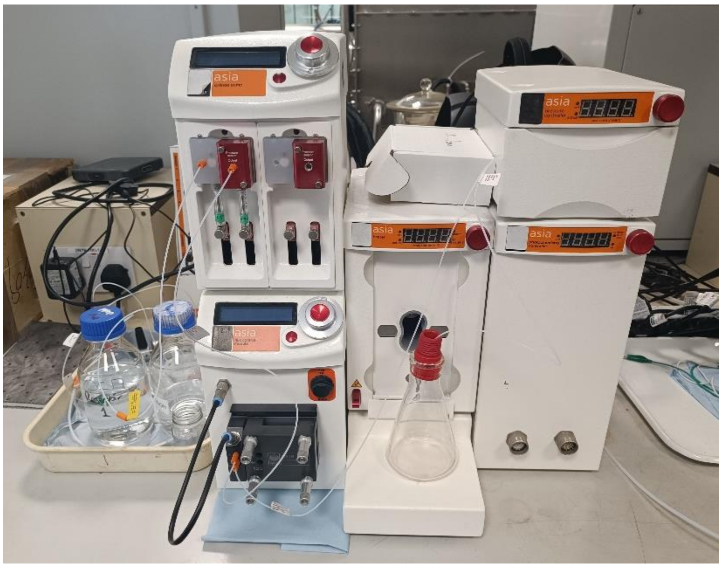
asia
asia
asia
asia
asia

Table 1: Optimisation table

Entry	(+)	(-)	F^- Source	Equiv.	$F.mol^{-1}$	Yield (%)	Entry	(+)	(-)	F^- Source	Equiv.	$F.mol^{-1}$	Yield (%)
1	C_{gr}	Pt	$Et_3N\cdot3HF$	2	3	95%	23	C_{gr}	Ni	$Et_3N\cdot3HF$	2	2.5	96
2[a)]	C_{gr}	Pt	$Et_3N\cdot3HF$	2	3	79%	24	C_{gr}	SS	$Et_3N\cdot3HF$	2	2.5	95
3	C_{gr}	Pt	$Et_3N\cdot3HF$	3	3	69%	25[c)]	C_{gr}	Pt	$Et_3N\cdot3HF$	2	3.5	98
4	C_{gr}	Pt	$Et_3N\cdot3HF$	4	2.5	71%	26[d)]	C_{gr}	Pt	$Et_3N\cdot3HF$	2	4.5	92
5[a)]	C_{gr}	Pt	$Et_3N\cdot3HF$	1	2	76%	27	C_{gr}	Pt	$Et_3N\cdot3HF$	2	3.5	92
6[a)]	C_{gr}	Pt	CsF	5	2.5	negligible	28[a)]	C_{gr}	Pt	$Et_3N\cdot3HF$	2	2.5	100
7[b)]	C_{gr}	Pt	$Coll\cdot HBF_4$	5	2	negligible	30	C_{gr}	Pt	$(Bu)_4N\cdot H_2F_3$	1	3	97
8	C_{gr}	Pt	$Et_3N\cdot3HF$	1.5	2	94.5	31	C_{gr}	Pt	CsF	1	2.5	27
9	C_{gr}	Pt	$Et_3N\cdot3HF$	2	2	99	32[a)]	C_{gr}	Pt	CsF	2	2.5	32
10	C_{gr}	C_{gr}	$Et_3N\cdot3HF$	2	2	83	33[a)]	C_{gr}	Pt	CsF	3	2.5	27
11	C_{gr}	Ni	$Et_3N\cdot3HF$	2	2	94	34[a)]	C_{gr}	Pt	KF, 18-Crown-6	5,5	4	67
12	C_{gr}	SS	$Et_3N\cdot3HF$	2	2	93	35[a)]	C_{gr}	Pt	KF, TFA	5,5	2	0
13[a)]	C_{gr}	Pt	CsF	5	2	8	36[a)]	C_{gr}	SS	$Et_3N\cdot3HF$	1	2	60
14	C_{gr}	Pt	$Et_3N\cdot3HF$	1.5	3	95	37	C_{gr}	SS	$Et_3N\cdot3HF$	1	3	82
15	C_{gr}	Pt	$Et_3N\cdot3HF$	2	3	100	38	C_{gr}	Pt	$Et_3N\cdot3HF$	2	4.5	76
16	C_{gr}	C_{gr}	$Et_3N\cdot3HF$	2	3	86	39	C_{gr}	SS	$Et_3N\cdot3HF$	2	2.5	96
17	C_{gr}	Ni	$Et_3N\cdot3HF$	2	3	96	40	C_{gr}	Ni	$Et_3N\cdot3HF$	2	3	94
18	C_{gr}	SS	$Et_3N\cdot3HF$	2	3	96	41	C_{gr}	Pt	$Et_3N\cdot3HF$	1	4	76
19[a)]	C_{gr}	Pt	CsF	5	3	7	42	Pt	SS	$Et_3N\cdot3HF$	2	2	0
20	C_{gr}	C_{gr}	$Et_3N\cdot3HF$	2	2.5	89	43	C_{gr}	SS	$Et_3N\cdot3HF$	1.5	3	78
21	C_{gr}	Pt	$Et_3N\cdot3HF$	2	4	99	44 [a)]	C_{gr}	SS	TMAF	1.5	2.5	19
22	C_{gr}	Pt	$Et_3N\cdot3HF$	2	3	84	45	C_{gr}	SS	TMAF	1.5	2.5	-

[a)] MeCN as solvent, [b)] in presence of 100 mg of molecular sieve, [c)] in presence of 1 equiv. of collidine, [d)] in presence of 1 equiv. of Et_3N.

Synthesis of secondary amines

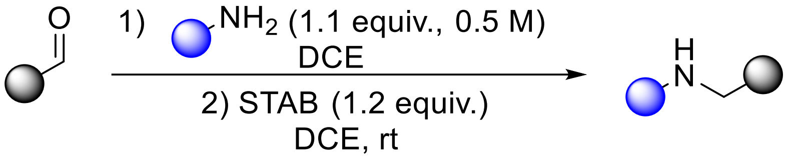

General Procedure A:[1] 1.1 equiv. of the primary amine, 1.0 equiv. of the aldehyde and 1,2-dichloroethane are added to a round bottom flask. Sodium triacetoxyborohydride (STAB) (1.2 equiv.) is added portionwise to the reaction. The mixture is stirred at room temperature until completion, as determined by thin-layer chromatography. The reaction is quenched using a saturated Na_2CO_3 solution and extracted with EtOAc. The combined organic layers are washed with brine, and then dried over $NaSO_4$. After filtration, the filtrate is concentrated under reduced pressure. The crude mixture is purified via flash column chromatography if necessary.

General Procedure B:[1] 1.0 equiv. of primary amine was added to a dried round-bottomed flask filled with methanol (0.5 M with respect to the amine), followed by the aldehyde (1.5 equiv). The reaction was stirred at room temperature for 4 h and then cooled to 0 °C with an ice-water bath. 2 equiv. of sodium borohydride was then added portion wise. The reaction was stirred for 4 h at room temperature. The solvent was removed under reduced pressure and EtOAc was added to the residue. The solution was washed with water and brine. The organic layer was dried over Na_2SO_4 and concentrated under reduced pressure. The crude mixture was purified via flash column chromatography if necessary.

1ca. Allylbenzylamine

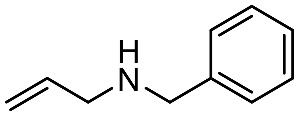

Obtained following the general procedure B as a pale-yellow liquid (1.0 g, 10.0 mmol, 68%)

^{1}H NMR (400 MHz, $CDCl_3$) δ 7.31 (d, *J* = 4.5 Hz, 4H), 7.27 – 7.21 (m, 1H), 6.00 – 5.85 (m, 1H), 5.24 – 5.06 (m, 2H), 3.78 (s, 2H), 3.26 (d, *J* = 5.9 Hz, 2H), 1.32 (s, 1H).

^{13}C NMR (101 MHz, $CDCl_3$) δ 140.4, 136.9, 128.5, 128.2, 127.0, 116.0, 53.3, 51.9.

IR: 3028, 1643, 1105, 915, 697.

HRMS (ESI) m/z: $[M+H]^+$ calcd for $C_{10}H_{14}N$ 148.1126, found 148.1132

In accordance with previous reports.[1]

1da. *N*-benzyl-3-butenylamine

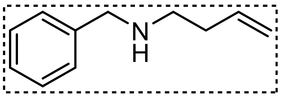

Obtained following the general procedure B as a pale-yellow liquid (810 mg, 10.0 mmol, 50%)

^{1}H NMR (500 MHz, $CDCl_3$) δ 7.48 – 7.09 (m, 5H), 5.85 – 5.73 (m, 1H), 5.13 – 5.00 (m, 2H), 3.80 (s, 2H), 2.71 (t, *J* = 6.8 Hz, 2H), 2.34 – 2.24 (m, 2H).

^{13}C NMR (126 MHz, $CDCl_3$) δ 140.6, 136.6, 128.5, 128.3, 127.0, 116.5, 54.0, 48.4, 34.4.

IR: 3316, 3029, 1790, 1641, 1227,1125, 913, 698.

HRMS (ESI) m/z: $[M+H]^+$ calcd for $C_{11}H_{16}N$ 162.1283, found 162.1268

In accordance with previous reports.[1]

1ea. *N*-benzylbut-2-yn-1-amine

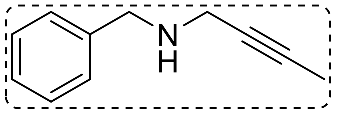

Obtained following the general procedure B as a yellow liquid (1.32 g, 15mmol, 55%)

^{1}H NMR (400 MHz, $CDCl_3$) δ 7.38 – 7.21 (m, 5H), 3.85 (s, 2H), 3.38 (q, *J* = 2.3 Hz, 2H), 1.84 (t, *J* = 2.4 Hz, 3H).

^{13}C NMR (101 MHz, $CDCl_3$) δ 139.9, 128.5, 128.5, 127.1, 79.3, 77.3, 52.6, 37.9, 3.6.

IR: 3028, 2243, 2052, 1792, 1223, 1027, 698.

HRMS (ESI) m/z: [M+H]+ calcd for $C_{11}H_{14}N$ 160.1126, found 160.1155

In accordance with previous reports.[2]

1na. *N*-benzyl-p-anisidine

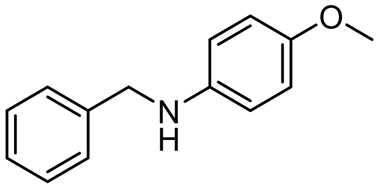

Obtained following the general procedure A as a pale-yellow liquid (1.63g, 10.0 mmol, 76%)

^{1}H NMR (400 MHz, $CDCl_3$) δ 7.42 – 7.20 (m, 5H), 6.78 (d, *J* = 9.2 Hz, 2H), 6.60 (d, *J* = 8.9 Hz, 2H), 4.28 (s, 2H), 3.77 (s, 1H), 3.74 (s, 3H).

^{13}C NMR (101 MHz, $CDCl_3$) δ 152.3, 142.6, 139.8, 128.7, 127.7, 127.3, 115.1, 114.2, 56.0, 49.4.

IR: 3308, 1792, 1598, 1362, 1264, 1106, 697

HRMS (ESI) m/z: $[M+H]^+$ calcd for $C_{14}H_{16}NO$ 214.1232, found 214.1252

In accordance with previous reports.[1]

Synthesis of oxamic acids

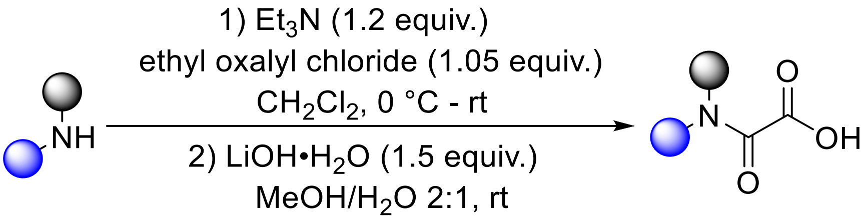

General procedure: To a 0.4 M solution of the amine in DCM is added 1.2 equiv. of Et_3N, followed by 1.05 equiv. of ethyl oxalyl chloride at 0°C. The solution is warmed to room temperature and followed by TLC or HPLC (usually less than 1 hour). Then the crude mixture is diluted with 1 M HCl and extracted with DCM. The organic layers are then washed with $NaHCO_3$ and dried with Na_2SO_4. The solvent is removed under reduced pressure. The crude mixture was purified via flash column chromatography if necessary.

The oxalyl ester is dissolved in a mixture of $MeOH/H_2O$ 2:1 (0.4 M). Then 1.5 equiv. of $LiOH.H_2O$ are added and the reaction is monitored by HPLC (usually 30 minutes. If longer, add more LiOH). Then the crude mixture is diluted with 1 M HCl and extracted with Et_2O. The organic layers are then combined and dried with Na_2SO_4. The solvent is removed under reduced pressure. The obtained carbamic was always found to be pure at the end of this procedure. If not, the minute impurities can be removed by crystallization in chloroform/hexane solution.

1a. 2-(Dibenzylamino)-2-oxoacetic acid

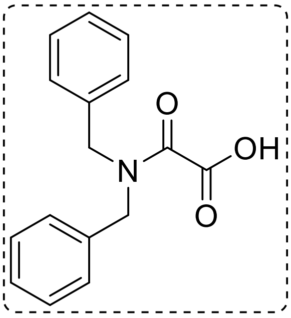

Obtained following the general procedure followed by crystallisation in chloroform/hexane solution to obtain a colorless solid (5.1 g, 18.9 mmol, 64 %).

^{1}H NMR (400 MHz, $CDCl_3$) δ = 7.45 – 7.30 (m, 6H), 7.29 – 7.19 (m, 4H), 4.93 (s, 2H), 4.56 (s, 2H).

^{13}C NMR (101 MHz, $CDCl_3$) δ = 163.8, 162.7, 134.9, 134.5, 129.4 – 128.0 (m), 51.0, 47.0.

IR: 2980, 1650, 1600

HRMS (ESI) m/z: $[M-H]^-$ calcd for $C_{16}H_{14}NO_3$ 268.0974, found 268.0983.

1b. 2-(Benzyl(methyl)amino)-2-oxoacetic acid

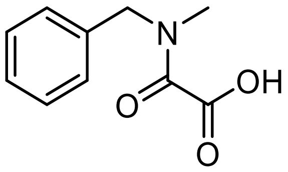

Obtained following the general procedure as a solid (1.8 g, 10 mmol, 93%)

^{1}H NMR (400 MHz, DMSO-d_6, both rotamers) δ 7.44 – 7.20 (m, 5H), 4.52 (s, 2H), 4.46 (s, 2H), 2.87 (s, 3H), 2.72 (s, 3H).

^{13}C NMR (101 MHz, DMSO-d_6, both rotamers) δ 165.1, 164.9, 163.0, 163.0, 136.2, 135.8, 128.7, 127.8, 127.7, 127.6, 127.49, 52.5, 48.3, 34.4, 30.6.

IR: 3032, 2825, 1992, 1600, 1454, 1361, 1222, 700.

HRMS (ESI) m/z: [M-H]$^+$ calcd for $C_{10}H_{10}NO_3$ 192.0661, found 192.0675

1c. 2-[Allyl(benzyl)amino]-2-oxo-acetic acid

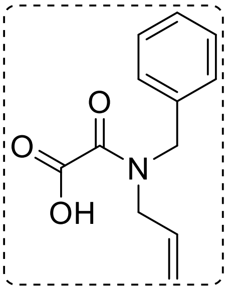

Obtained following the general procedure as a liquid (2.03 g, 10 mmol, 93%)

^{1}H NMR (400 MHz, $CDCl_3$, both rotamers) δ 11.26 (s, 1H), 7.40 – 7.22 (m, 5H), 5.89 – 5.64 (m, 1H), 5.37 – 5.08 (m, 2H), 4.70 (s, 2H), 4.63 (s, 2H), 4.04 (d, J = 6.0 Hz, 2H), 3.92 (d, J = 6.0 Hz, 2H).

^{13}C NMR (101 MHz, $CDCl_3$, both rotamers) δ 162.6, 162.5, 162.3, 162.0, 135.1, 134.8, 132.0, 130.7, 128.9, 128.5, 128.3, 128.2, 128.1, 119.7, 119.4, 51.3, 50.3, 48.1, 47.0.

IR: 3031, 2929, 1955, 1611, 1453, 1195, 931, 700

HRMS (ESI) m/z: [M-H]$^+$ calcd for $C_{12}H_{12}NO_3$ 218.0817, found 218.0843

1d. [Benzyl(but-3-en-1-yl)carbamoyl]formic acid

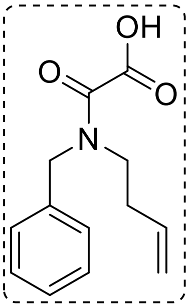

Obtained following the general procedure as a liquid (1.82 g, 10.0 mmol, 78%)

^{1}H NMR (400 MHz, $CDCl_3$, both rotamers) δ 11.37 (s, 1H), 7.68 – 6.81 (m, 5H), 5.76 – 5.62 (m, 1H), 4.96-5.12 (m, 2H), 4.65 (s, 2H), 3.42 (dt, *J* = 46.8, 7.5 Hz, 2H), 2.40 – 2.21 (m, 2H).

^{13}C NMR (101 MHz, $CDCl_3$, both rotamers) δ 163.1, 162.7, 162.3 135.2, 134.8, 134.2, 133.7, 128.8, 128.3, 128.1, 128.1, 127.9, 117.9, 117.4, 52.3, 48.5, 47.3, 44.4, 32.7, 30.9.

IR: 2939, 1739, 1608, 1452, 1193, 918, 698.

HRMS (ESI) m/z: $[M+H]^+$ calcd for $C_{13}H_{16}NO_3$ 234.1130, found 234.1121.

1e. 2-[Benzyl(but-2-yn-1-yl)amino]-2-oxoacetic acid

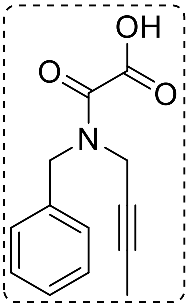

Obtained following the general procedure as a liquid (2.29 g, 10 mmol, 99%)

^{1}H NMR (400 MHz, $CDCl_3$, both rotamers) δ 10.11 (s, 1H), 7.57 – 6.86 (m, 5H), 4.90 (s, 2H), 4.77 (s, 2H), 4.42 – 4.01 (m, 2H), 1.79-1.83 (m, 3H).

^{13}C NMR (101 MHz, $CDCl_3$, both rotamers) δ 161.6, 161.4, 160.9, 134.9, 134.6, 129.0, 128.7, 128.4, 128.4, 128.3, 82.1, 81.5, 72.4, 71.9, 51.0, 48.9, 37.9, 34.5, 3.6.

IR: 3454, 2921, 2235, 1619, 1439, 1028, 699.

HRMS (ESI) m/z: $[M+H]^+$ calcd for $C_{13}H_{14}NO_3$ 232.0974, found 232.0988.

1f. 2-[Methyl(2-phenylethyl)amino]-2-oxoacetic acid

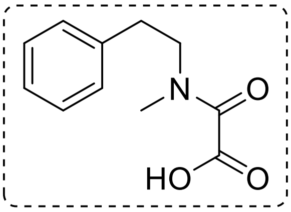

Obtained following the general procedure as a solid (2.04 g, 10 mmol, 99%)

^{1}H NMR (400 MHz, $CDCl_3$, both rotamers) δ 7.36 – 7.17 (m, 5H), 3.99 – 3.89 (m, 1H), 3.71 – 3.63 (m, 1H), 3.24 (s, 1H), 3.05 – 2.89 (m, 4H).

^{13}C NMR (101 MHz, $CDCl_3$, both rotamers) δ 161.1, 161.0, 160.9, 160.7, 160.7, 138.1, 137.5, 129.0, 128.9, 128.8, 128.8, 127.0, 126.9, 53.0, 51.3, 37.4, 35.3, 33.1.

IR: 3661, 2936, 2554, 1755, 1617, 1457, 1207, 723.

HRMS (ESI) m/z: $[M+H]^+$ calcd for $C_{11}H_{14}NO_3$ 208.0974, found 208.0982

1g. 2-[3,4-Dihydroisoquinolin-2(1H)-yl]-2-oxoacetic acid

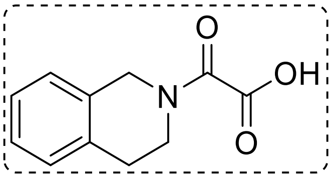

Obtained following the general procedure followed by recrystallisation from chloroform/hexane as a mixture of two rotamers as a white powder (1.806 g, 8.80 mmol, 57%).

^{1}H NMR (400 MHz, $CDCl_3$, both rotamers) δ = 11.27 (s, 2H), 7.32 – 7.14 (m, 8H), 5.13 (s, 2H), 4.84 (s, 2H), 4.13 (t, *J*=5.8 Hz, 2H), 3.93 (t, *J*=6.1 Hz, 2H), 3.0 (t, *J*=6.0 Hz, 4H).

^{13}C NMR (101 MHz, $CDCl_3$, both rotamers) δ = 161.2, 161.0, 160.6, 160.0, 133.9, 133.8, 131.9, 131.4, 128.7, 128.7, 127.4, 127.2, 127.0, 127.0, 126.6, 126.4, 48.2, 45.4, 44.7, 41.9, 29.4 28.1.

IR: 3660, 2981, 2890, 1382

HRMS (ESI) m/z: $[M+H]^+$ calcd for $C_{11}H_{12}NO_3$ 206.0828, found 206.0817.

1h. 2-(7-Chloro-1,2,3,4-tetrahydroisoquinolin-2-yl)-2-oxoacetic acid

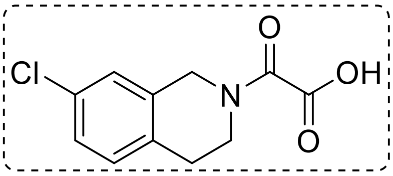

Obtained following the general procedure followed by recrystallisation from chloroform/hexane as a mixture of two rotamers as a white powder (692.2 mg, 2.896 mmol, 54%).

^{1}H NMR (400 MHz, $CDCl_3$, both rotamers) δ = 7.21 - 7.09 (m, 6H), 5.20 (s, 2H), 4.76 (s, 2H), 4.22 (t, *J*=6.0 Hz, 2H), 3.88 (t, *J*=6.1 Hz, 2H), 2.95 (t, *J*=6.0 Hz, 2H), 2.91 (t, *J*=6.0 Hz, 2H).

^{13}C NMR (101 MHz, $CDCl_3$, both rotamers) δ = 160.8, 160.5, 159.9, 159.2, 133.8, 133.2, 132.7, 132.6, 132.3, 132.3, 130.1, 130.1, 127.6, 127.5, 126.6, 126.4, 47.9, 45.2, 44.5, 41.9, 29.0, 27.6.

IR: 2980, 1787, 1655, 1602

HRMS (ESI) m/z: $[M+H]^+$ calcd for $C_{11}H_{11}NO_3Cl$ 240.0427, found 240.0439.

1i. 2-(7-Bromo-1,2,3,4-tetrahydroisoquinolin-2-yl)-2-oxoacetic acid

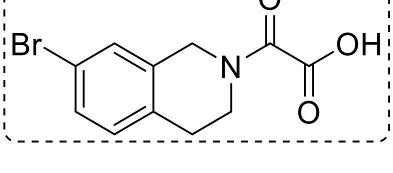

Obtained following the general procedure as a mixture of two rotamers as a white powder (1.0274 g, 3.62 mmol, 78%).

^{1}H NMR (400 MHz, $CDCl_3$, both rotamers) δ = 7.39 – 7.32 (m, 2H), 7.31 – 7.28 (m, 2H), 7.05 (d, *J*=8.1, 2H), 5.19 (s, 2H), 4.76 (s, 2H), 4.32 – 4.12 (m, 2H), 3.88 (t, *J*=6.1, 2H), 2.86-2.98 (m, 4H).

^{13}C NMR (101 MHz, $CDCl_3$, both rotamers) δ = 160.1, 159.9, 159.4, 158.8, 134.2, 133.6, 132.8, 130.6, 130.5, 130.4, 129.5, 129.3, 120.5, 47.8, 45.5, 44.6, 42.2, 29.1, 27.7.

IR: 3660, 2981, 2889, 1740, 1653, 1619

HRMS (ESI) m/z: $[M+H]^+$ calcd for $C_{11}H_{11}NO_3Br$ 283.9922, found 283.9934.

1j. 2-(Benzyl-cyclopropylamino)-2-oxoacetic acid

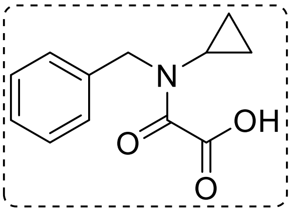

Obtained following the general procedure as a solid (1.68 g, 10 mmol, 77%)

^{1}H NMR (400 MHz, DMSO-*d$_6$*) δ 7.31 – 7.10 (m, 5H), 4.41 (s, 2H), 0.70 – 0.52 (m, 4H).

^{13}C NMR (101 MHz, DMSO-*d$_6$*) δ 165.5, 165.2, 136.9, 128.7, 127.5, 127.3, 48.1, 29.6, 7.4.

IR: 3366, 3240, 2364, 1681, 1173, 883.

HRMS (ESI) m/z: $[M+H]^+$ calcd for $C_{12}H_{14}NO_3$ 220.0974, found 220.1003.

1k. 2-[Bis(2-chloroethyl)amino]-2-oxoacetic acid

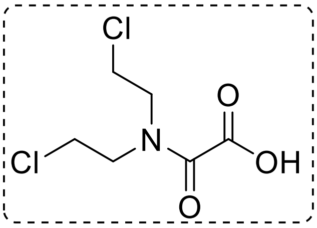

Obtained following the general procedure as an off-white solid (3.2091 g, 14.99 mmol, 75 %)

^{1}H NMR (500 MHz, $CDCl_3$) δ = 4.13 (t, *J*=6.2 Hz, 2H), 3.84 (t, *J*=5.9 Hz, 2H), 3.75 (t, *J*=6.2 Hz, 4H).

^{13}C NMR (126 MHz, $CDCl_3$) δ = 161.3, 160.4, 52.2, 51.0, 42.3, 40.5.

IR: 1735, 1630, 1173

HRMS (ESI) m/z: $[M-H]^-$ calcd for $C_6H_8NO_3Cl_2$ 211.9984, found 211.9912.

1l. 2-[Methyl(phenyl)amino]-2-oxoacetic acid

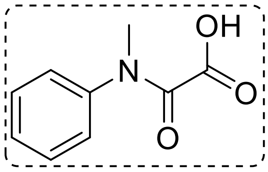

Obtained following the general procedure as a solid (1.5 g, 10.0 mmol, 84%)

^{1}H NMR (400 MHz, DMSO-*d$_6$*) δ 7.47 – 7.40 (m, 2H), 7.38 – 7.30 (m, 3H), 3.24 (s, 3H).

^{13}C NMR (101 MHz, DMSO-*d$_6$*) δ 164.4, 162.8, 141.5, 129.5, 128.0, 126.4, 35.3.

IR: 1969, 1791, 1592, 1340, 1118, 698.

HRMS (ESI) m/z: $[M-H]^+$ calcd for $C_9H_8NO_3$ 178.0504, found 178.0507

In accordance with previous reports.[3]

1m. 2-(Diphenylamino)-2-oxoacetic acid

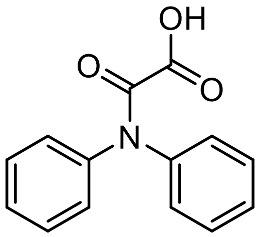

Obtained following the general procedure as a solid (2.39 g, 10 mmol, 99%)

^{1}H NMR (400 MHz, DMSO-*d$_6$*) δ 7.49 – 7.26 (m, 10H).

^{13}C NMR (101 MHz, DMSO-*d$_6$*) δ 164.0, 162.5, 162.4, 140.5, 129.6, 129.2, 129.0, 128.0, 127.1, 127.0.

IR: 3200, 2257, 2164, 2011, 1650, 1493, 1295, 698.

HRMS (ESI) m/z: $[M-H]^+$ calcd for $C_{14}H_{10}NO_3$ 240.0661, found 240.0661

In accordance with previous reports.[3]

1n. 2-[Benzyl(4-methoxyphenyl)amino]-2-oxoacetic acid

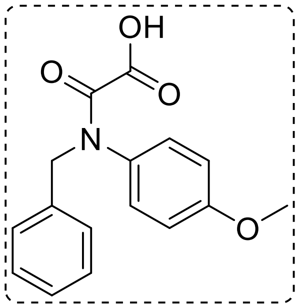

Obtained following the general procedure as a solid (2.5 g, 10 mmol, 88%)

^{1}H NMR (400 MHz, $CDCl_3$) δ 8.82 (s, 1H), 7.30 – 7.23 (m, 3H), 7.22 – 7.14 (m, 2H), 6.95 – 6.90 (m, 2H), 6.84 – 6.73 (m, 2H), 4.86 (s, 2H), 3.76 (s, 3H).

^{13}C NMR (101 MHz, $CDCl_3$) δ 163.0, 161.8, 159.7, 135.5, 132.1, 129.0, 128.9, 128.7, 128.1, 114.6, 55.5, 53.9.

IR: 2936, 2839, 2552, 1887,1623, 1511, 1249, 1025, 698.

HRMS (ESI) m/z: $[M-H]^+$ calcd for $C_{16}H_{14}NO_4$ 284.0923, found 284.0923

1o. 2-(4-Ethoxycarbonylpiperidin-1-yl)-2-oxoacetic acid

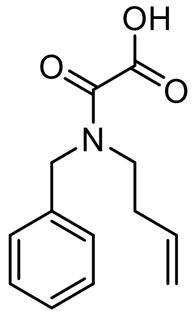

To a solution of oxalyl chloride (0.43 mL, 5.00 mmol) in THF (8.5 mL) at 0 °C under N_2 atmosphere is added ^{t}buOH (0.47 mL, 5.00 mmol). The mixture is stirred for 1 h at 0 °C. Ethyl piperidine-4-carboxylate (0.84 mL, 5.5 mmol) and triethylamine (2 mL, 15 mmol) are then added, and the solution is further stirred for 2 h. The reaction is quenched with saturated aqueous NH_4Cl solution and evaporated under reduced pressure. The aqueous layer is extracted with EtOAc. The combined organic layers are then washed with water and brine, dried over Na_2SO_4, and concentrated under reduced pressure to afford pure ethyl 1-(2-(tert-butoxy)-2-oxoacetyl)piperidine-4-carboxylate. After dissolution of the ester in 5 mL of DCM, TFA is added slowly while stirring at 0 °C. The reaction mixture is then stirred at r.t. overnight. Solvent is then removed under reduced pressure and the pure product is dried under high vacuum to afford a light brown solid. (1.06 g, 4.65 mmol, 93%).

^{1}H NMR (400 MHz, $CDCl_3$) δ = 4.73 – 4.59 (m, 1H), 4.30 (dt, *J*=13.5, 4.3 Hz, 1H), 4.16 (q, *J*=7.1 Hz, 2H), 3.53 – 3.40 (m, 1H), 3.17 – 3.04 (m, 1H), 2.62 (tt, *J*=10.2, 4.1 Hz, 1H), 2.03 (dq, *J*=12.7, 3.9 Hz, 2H), 1.81 (m, 2H), 1.27 (t, *J*=7.1 Hz, 3H).

^{13}C NMR (101 MHz, $CDCl_3$) δ = 173.9, 162.2, 160.4, 61.1, 46.0, 42.0, 40.5, 28.4, 27.5, 14.3.

IR: 1723, 1589, 1196, 1445, 1196

HRMS (ESI) m/z: $[M+H]^+$ calcd for $C_{10}H_{16}NO_5$ 230.1028, found 230.1065.

1p. 2-(1,1-Dioxo-1,4-thiazinan-4-yl)-2-oxoacetic acid

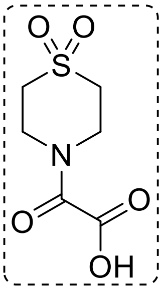

To a 0.4 M solution of thiomorpholine (0.5 mL, 5 mmol) in 12.5 mL of DCM is added 1.2 equiv. of Et_3N (0.81 mL, 6 mmol), followed by ethyl oxalyl chloride (0.59 mL, 5.25 mmol) at 0°C. The solution is warmed to room temperature and stirred for 1 hour. The crude mixture is diluted with 1 M HCl and extracted with DCM. The combined organic layers are then washed with $NaHCO_3$ and dried with Na_2SO_4. The solvent is removed under reduced pressure to quantitatively afford the pure ethyl 2-oxo-2-thiomorpholinoacetate. The product is dissolved in DCM (1 mL) and 3-chloroperoxybenzoic acid (1.93 g, 12.5 mmol) is added at 0 °C. Stirring at room temperature for 16 h is followed by washing of the reaction mixture with $Na_2S_2O_4$. The organic layer is then dried with Na_2SO_4 and the solvent is removed under reduced pressure to afford ethyl 2-(1,1-dioxidothiomorpholino)-2-oxoacetate (823 mg, 3.5 mmol, 70 %). Without any further purification, the previous product is dissolved in 9.5 mL of a 2:1 mixture of MeOH/H_2O. Lithium hydroxide monohydrate (219 mg, 5.25 mmol) is then added and the reaction is stirred at room temperature for 1 h. The crude mixture is diluted with 1 M HCl and extracted with Et_2O. The organic layers are then combined and dried with Na_2SO_4. The solvent is removed under reduced pressure to afford the pure desired product as a white solid (79 mg, 0.38 mmol, 11%)

^{1}H NMR (500 MHz, DMSO-d_6) δ = 3.93 – 3.85 (m, 2H), 3.85 – 3.75 (m, 2H), 3.32 – 3.15 (m, 4H).

^{13}C NMR (126 MHz, DMSO-d_6) δ = 164.0, 161.4, 51.1, 50.5, 44.2, 39.1.

IR: 1645, 1460, 1293, 1121, 932

HRMS (ESI) m/z: $[M-H]^+$ calcd for $C_6H_8NO_5S$ 206.0123, found 206.0143.

1q. 2-(4-Tert-butoxycarbonylpiperazin-1-yl)-2-oxoacetic acid

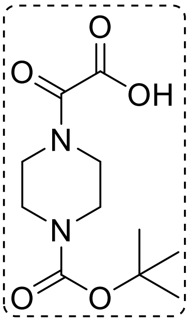

Obtained following the general procedure as a white solid (775 mg, 3.0 mmol, 75%)

^{1}H NMR (400 MHz, $CDCl_3$) δ = 4.17 – 4.03 (m, 2H), 3.74 – 3.63 (m, 2H), 3.57 – 3.46 (m, 4H), 1.48 (s, 9H).

^{13}C NMR (126 MHz, $CDCl_3$) δ = 160.5, 159.2, 154.6, 81.1, 46.6, 28.5.

IR: 1744, 1675, 1603, 1423, 1206

HRMS (ESI) m/z: $[M+H]^+$ calcd for $C_{11}H_{19}N_2O_5$ 259.1294, found 259.1306.

1r. 2-Morpholino-2-oxoacetic acid

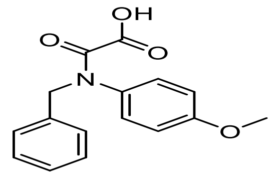

Obtained following the general procedure as a solid (0.636 g, 10 mmol, 40%)

^{1}**H NMR** (400 MHz, DMSO-D_6) δ 3.63 – 3.54 (m, 4H), 3.50 – 3.43 (m, 2H), 3.41 – 3.34 (m, 2H).

^{13}**C NMR** (101 MHz, DMSO-D_6) δ 164.5, 161.3, 66.2, 65.7, 45.7, 40.7.

IR: 2857, 2466, 1919, 1739, 1597, 1444, 1209, 1115.

HRMS (ESI) m/z: $[M+H]^+$ calcd for $C_6H_{10}NO_4$ 160.0610, found 160.0612.

In accordance with previous reports.[3]

1s. 2-((3-(10,11-Dihydro-5H-dibenzo[a,d][7]annulen-5-ylidene)propyl)(methyl)amino)-2-oxoacetic acid

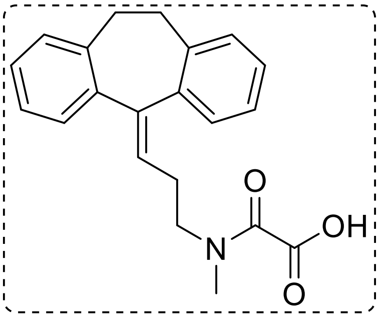

Obtained following the general procedure as a solid (2.1 g, 10.0 mmol, 63%)

^{1}**H NMR** (400 MHz, CDCl$_3$, both rotamers) δ 8.60 (s, 1H), 7.37 – 6.98 (m, 8H), 5.81 (t, *J* = 7.5 Hz, 1H), 3.91 – 3.81 (m, 1H), 3.57 – 3.44 (m, 1H), 3.43 – 3.15 (m, 3H), 3.08 – 2.72 (m, 4H), 2.60 – 2.38 (m, 2H).

^{13}**C NMR** (101 MHz, CDCl$_3$, both rotamers) δ 160.0, 159.7, 159.5, 159.3, 146.3, 145.9, 140.7, 140.7, 139.6, 139.5, 139.5, 137.2, 137.2, 130.3, 130.3, 128.6, 128.6, 128.3, 128.3, 128.1, 127.9, 127.9, 127.5, 126.4, 126.3, 126.0, 126.0, 126.0, 50.8, 50.0, 37.0, 35.5, 33.9, 32.1, 32.0, 28.7, 26.9.

IR: 2921, 1923, 1741, 1619, 1485, 1184, 757.

HRMS (ESI) m/z: $[M+H]^+$ calcd for $C_{21}H_{22}NO_3$ 336.1600, found 336.1637

1t. 2-Oxo-2-(4-(trifluoromethyl)piperidin-1-yl)acetic acid

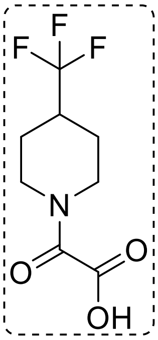

Obtained following the general procedure as a pale pink solid (2.6 g, 13.1 mmol, 30%)

^{1}H NMR (400 MHz, DMSO-*D*$_6$) δ 4.32 (d, *J* = 13.3 Hz, 1H), 3.64 (d, *J* = 13.7 Hz, 1H), 3.19 (td, *J* = 13.7, 2.9 Hz, 1H), 2.89 – 2.57 (m, 2H), 1.96 – 1.84 (m, 2H), 1.43 – 1.22 (m, 2H).

^{13}C NMR (101 MHz, DMSO-*D*$_6$) δ 164.6, 161.3, 127.4 (q, *J* = 278.4 Hz), 44.1, 38.7, 38,6 (q, *J* = 27.0 Hz), 24.6 (d, *J*=2.2 Hz), 23.6 (d, *J*=2.0 Hz).

^{19}F NMR (376 MHz, DMSO-*D*$_6$) δ -72.48 (d, *J = 9.0 Hz*, 3F).

IR: 2878, 2706, 1746, 1586, 1453, 1157, 1084, 1003.

HRMS (ESI) m/z: [M+H]$^+$ calcd for $C_8H_{11}F_3NO_3$ 226.0691, found 226.0705.

1u. 2-(4-Cyanopiperidin-1-yl)-2-oxoacetic acid

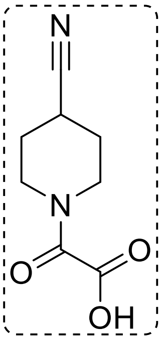

Obtained following the general procedure as a colorless solid (1.3 g, 18.1 mmol, 40%)

^{1}H NMR (400 MHz, DMSO-*D*$_6$) δ 3.86 – 3.08 (m, 7H), 1.98 – 1.85 (m, 1H), 1.76 – 1.59 (m, 1H).

^{13}C NMR (101 MHz, DMSO-*D*$_6$) δ 165.2, 161.7, 122.1, 44.4, 39.0, 28.9, 27.9, 25.8.

IR: 2923, 1949, 1731, 1574, 1207, 1043.

HRMS (ESI) m/z: [M+H]$^+$ calcd for $C_8H_{11}N_2O_3$ 183.0770, found 183.0786.

1v. 2-Oxo-2-(4-phenylpiperidin-1-yl)acetic acid

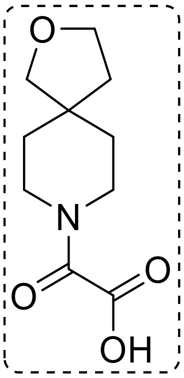

Obtained following the general procedure as a white solid (886 mg, 3.8 mmol, 95%)

^{1}H NMR (500 MHz, DMSO-*d*$_6$) δ = 7.35 – 7.25 (m, 2H), 7.28 – 7.15 (m, 3H), 4.37 (ddt, *J*=13.1, 4.2, 1.9 Hz, 1H), 3.65 (ddt, *J*=13.2, 4.2, 1.8 Hz, 1H), 3.26 (dd, *J*=26.1, 2.7 Hz, 1H), 2.89 – 2.74 (m, 2H), 1.85 (dtd, *J*=15.9, 5.3, 3.4 Hz, 4H), 1.53 (dqd, *J*=25.6, 12.7, 4.3 Hz, 2H).

^{13}C NMR (126 MHz, DMSO-*d*$_6$) δ = 165.0, 161.2, 145.2, 128.5, 126.7, 126.3, 46.0, 41.4, 40.6, 33.1 32.2.

IR: 1739, 1637, 1600, 1450, 1203.

HRMS (ESI) m/z: $[M+H]^+$ calcd for $C_{13}H_{16}NO_3$ 234.1130, found 234.1162.

1w. 2-Oxo-2-(6-azaspiro[2.5]octan-6-yl)acetic acid

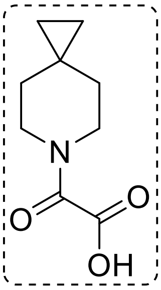

Obtained following the general procedure as a pale yellow solid (1.85 g, 13.6 mmol, 74%)

^{1}H NMR (400 MHz, DMSO-*D*$_6$) δ 3.52 – 3.45 (m, 2H), 3.37 – 3.30 (m, 2H), 1.33 (dt, *J* = 14.4, 5.6 Hz, 4H), 0.35 (s, 4H).

^{13}C NMR (101 MHz, DMSO-*D*$_6$) δ 165.6, 161.9, 46.3, 40.8, 35.3, 34.3, 18.1, 11.6.

IR: 2848, 2643, 1742, 1590, 1373, 1203, 1100.

HRMS (ESI) m/z: $[M+H]^+$ calcd for $C_9H_{14}NO_3$ 184.0974, found 184.1010.

1x. 2-Oxo-2-(2-oxa-8-azaspiro[4.5]decan-8-yl)acetic acid

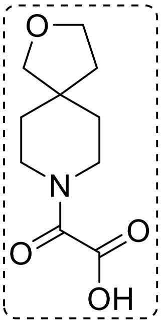

Obtained following the general procedure as a pale yellow solid (500 mg, 11.2 mmol, 21%)

^{1}H NMR (400 MHz, DMSO-*D*$_6$) δ 3.74 (t, *J* = 7.1 Hz, 2H), 3.53 (dt, *J* = 11.1, 6.1 Hz, 1H), 3.46 (s, 2H), 3.42 – 3.26 (m, 3H), 1.74 (t, *J* = 7.1 Hz, 2H), 1.50 (dt, *J* = 15.9, 6.3 Hz, 4H).

^{13}C NMR (101 MHz, DMSO-*D*$_6$) δ 165.0, 161.2, 76.7, 66.4, 43.7, 41.8, 38.2, 36.3, 34.5, 33.6.

IR: 2876, 2537, 1722, 1640, 1470, 1187, 1031.

HRMS (ESI) m/z: $[M+H]^+$ calcd for $C_{10}H_{16}NO_4$ 214.1079, found 214.1104.

1y. 2-Oxo-2-(4-(tetrahydrofuran-2-carbonyl)piperazin-1-yl)acetic acid

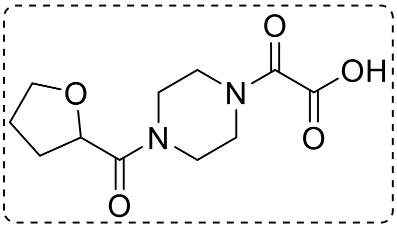

Obtained following the general procedure as a pale yellow solid (1.126 g, 4.394 mmol, 22%)

^{1}H NMR (500 MHz, $CDCl_3$, both rotamers) δ = 10.41 (s, 1H), 4.59 (q, *J*=7.1 Hz, 1H), 4.12 – 3.18 (m, 10H), 2.33 – 1.62 (m, 4H).

^{13}C NMR (126 MHz, $CDCl_3$, both rotamers) δ = 171.1, 170.9, 162.6, 162.5, 161.0, 160.7, 75.8, 75.7, 69.2, 69.2, 46.3, 45.9, 45.4, 44.8, 42.3, 41.8, 41.6, 41.5, 28.6, 28.5, 25.6.

IR: 2980, 1735, 1635, 1439, 1381.

HRMS (ESI) m/z: $[M-H]^-$ calcd for $C_{11}H_{15}N_2O_5$ 255.0981, found 255.1044.

1z. 2-(4-Ethynylpiperidin-1-yl)-2-oxoacetic acid

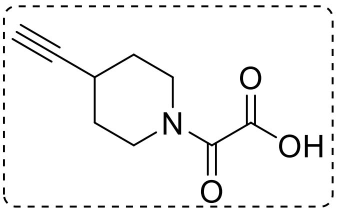

Obtained following the general procedure as a half-white solid (2 g, 13.8 mmol, 80%)

^{1}H NMR (500 MHz, DMSO-D_6) δ 3.75 (ddd, *J* = 13.2, 5.8, 3.8 Hz, 1H), 3.45 (ddd, *J* = 13.7, 6.3, 3.9 Hz, 1H), 3.29 – 3.15 (m, 2H), 3.02 (d, *J* = 2.4 Hz, 1H), 2.73 (tdd, *J* = 8.6, 6.4, 3.9 Hz, 1H), 1.80 (tdd, *J* = 12.5, 6.0, 3.3 Hz, 2H), 1.46 (dddt, *J* = 22.5, 13.9, 9.2, 4.5 Hz, 2H).

^{13}C NMR (126 MHz, DMSO-D_6) δ 164.9, 161.2, 86.1, 72.5, 44.1, 38.7, 31.4, 30.5, 25.9.

IR: 3282, 2960, 1740, 1605, 1448, 1265, 1207, 1054.

HRMS (ESI) m/z: $[M+H]^+$ calcd for $C_9H_{12}NO_3$ 182.0817, found 182.0838.

Electrochemical preparation of carbamoyl fluorides

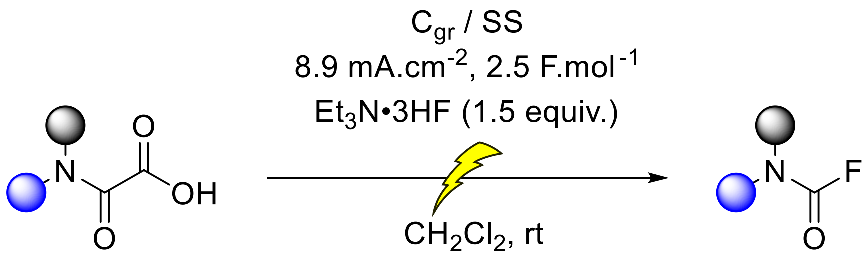

General procedure: 0.4 mmol of oxamic acid, 1.5 equiv. of $Et_3N.3HF$ and 5 mL of DCM are added to a 5 mL one compartment Electrasyn vial. The solution is then electrolyzed with a 8.9 $mA.cm^{-2}$ current density for 2.5 or 3 $F.mol^{-1}$ (ie. 1.34 h or 1.6 h)using a carbon graphite anode (WE) and a stainless-steel cathode (CE). The reaction is monitored by HPLC. After full consumption of the starting acid, the reaction mixture is quenched with a saturated aqueous solution of $NaHCO_3$, extracted with Et_2O. The organic layers are then combined and dried with Na_2SO_4. The solvent is removed under reduced pressure. The crude mixture was purified via flash column chromatography if necessary.

Large scale reaction:

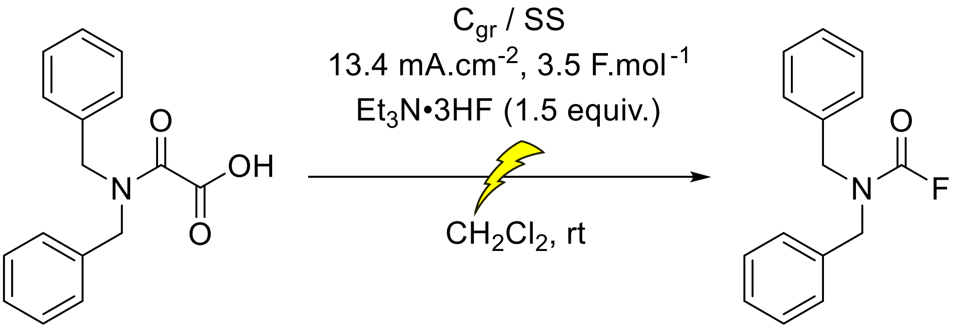

1a **2a**

2-(Dibenzylamino)-2-oxoacetic acid (**1a**) (1.0 g, 3.73 mmol, 1.0 eq.) $Et_3N.3HF$ (902 mg, 5.6 mmol, 1.5 eq.) and DCM (20 mL, 0.19 M) are added to a 20 mL one compartment Electrasyn vial. The solution is then electrolyzed at 30 mA for 3.5 $F.mol^{-1}$ (11.6 h) using a carbon graphite anode (WE) and a stainless-steel cathode (CE). The reaction is monitored by HPLC. After full consumption of the starting acid, the reaction mixture is quenched with a saturated aqueous solution of $NaHCO_3$, extracted with Et_2O. The organic layers are then combined and dried with Na_2SO_4. The solvent is removed under reduced pressure to obtain pure product (885 mg, 3,6 mmol, 98%).

Flow chemistry procedure: A solution of 2-(Dibenzylamino)-2-oxoacetic acid (**1a**) (1 equiv.) and $Et_3N.3HF$ (1.5 equiv.) in DCM (0.08 M related to the oxamic acid) was pumped at 0.08 $mL.min^{-1}$ through an Asia® FLUX reactor (225 µL, 25 mA) followed by an Asia® Pressure Controller set at 4.5 bar. After

the steady state was reached, 5 mL were collected for 62.5 min. The mixture is quenched with a saturated aqueous solution of $NaHCO_3$, extracted with Et_2O. The organic layers are then combined and dried with Na_2SO_4. Removal of the solvent under reduced pressure affords pure carbamoyl fluoride as a colourless liquid (92.4 mg, 0.38 mmol, 95%).

2a. Dibenzylcarbamic fluoride

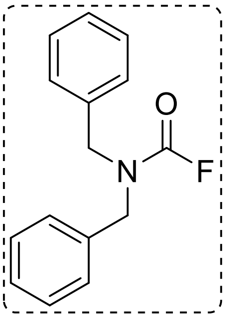

Obtained following the general procedure as a colourless liquid (94.5 mg, 0.388 mmol, 95%.)

^{1}H NMR (400 MHz, $CDCl_3$) δ = 7.33 – 7.08 (m, 10H), 4.33 (s, 2H), 4.25 (s, 2H).

^{13}C NMR (101 MHz, $CDCl_3$) δ = 148.3 (d, *J*=287.9 Hz), 135.5, 135.4, 129.1, 129.0, 128.5, 128.3, 128.28, 127.9, 50.6, 49.8 (d, *J*=3.6 Hz).

^{19}F NMR (376 MHz, $CDCl_3$) δ = -22.52.

HRMS (ESI) m/z: $[M+H]^+$ calcd for $C_{15}H_{15}FNO$ 244.1138, found 244.1145

In accordance with previous reports.[4]

2b. Benzyl(methyl)carbamic fluoride

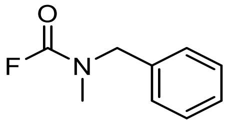

Obtained following the general procedure as a colourless liquid (40 mg, 0.4 mmol, 60 %)

^{1}H NMR (400 MHz, $CDCl_3$, both rotamers) δ 7.42 – 7.21 (m, 5H), 4.47 (s, 2H), 4.43 (s, 2H), 2.92 (s, 3H), 2.87 (s, 3H).

^{13}C NMR (101 MHz, $CDCl_3$, both rotamers) δ 148.4 (d, *J*=287.1 Hz), 147.7 (d, *J*=286.1 Hz), 135.5, 129.1, 129.0, 128.3, 128.2, 127.6, 53.7, 53.2 (d, *J*=3.9 Hz), 35.1, 33.9 (d, *J*=3.9 Hz).

^{19}F NMR (376 MHz, $CDCl_3$, both rotamers) δ -21.68, -23.33.

IR: 1779, 1401, 1211, 1111, 698

HRMS (ESI) m/z: $[M+H]^+$ calcd for $C_9H_{11}FNO$ 168.0825, found 168.0815.

In accordance with previous reports.[4]

2c. Allyl(benzyl)carbamic fluoride

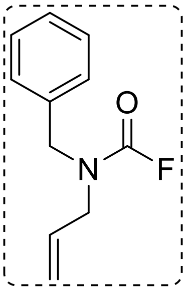

Obtained following the general procedure as a colourless liquid (43 mg, 0.4 mmol, 55 %)

^{1}H NMR (400 MHz, $CDCl_3$, both rotamers) δ 7.43 – 7.19 (m, 5H), 5.85 – 5.65 (m, 1H), 5.30 – 5.13 (m, 2H), 4.44 (d, *J* = 22.7 Hz, 2H), 3.85 (dd, *J* = 6.2, 1.6 Hz, 1H), 3.76 (dt, *J* = 5.8, 1.5 Hz, 1H).

^{13}C NMR (101 MHz, $CDCl_3$, both rotamers) δ 148.2 (d, *J*=288.5 Hz), 147.6 (d, *J*=288.5 Hz), 135.7 (d, *J* = 4.8 Hz), 131.7, 131.3, 129.0, 129.0, 128.4, 128.2 (d, *J* = 2.4 Hz), 127.8, 119.2, 118.5, 51.0, 50.1 (d, *J* = 3.4 Hz), 49.9, 49.0 (d, *J* = 3.4 Hz).

^{19}F NMR (376 MHz, $CDCl_3$, both rotamers) δ -22.00, -22.54.

IR: 1779, 1415, 1225, 935, 698.

HRMS (ESI) m/z: $[M+H]^+$ calcd for $C_{11}H_{13}FNO$ 194.0981, found 194.0965.

In accordance with previous reports.[1]

2d. *N*-benzyl-N-but-3-enylcarbamoyl fluoride

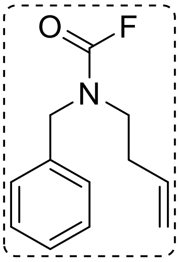

Obtained following the general procedure as a colourless liquid (46 mg, 0.4 mmol, 55 %)

^{1}H NMR (400 MHz, $CDCl_3$, both rotamers) δ 7.41 – 7.19 (m, 5H), 5.81 – 5.63 (m, 1H), 5.13 – 5.01 (m, 2H), 4.46 (d, *J* = 20.3 Hz, 2H), 3.27 (ddd, *J* = 32.5, 8.0, 6.6 Hz, 2H), 2.39 – 2.21 (m, 2H).

^{13}C NMR (101 MHz, $CDCl_3$, both rotamers) δ 148.5 (d, *J*=287.5 Hz), 147.6 (d, *J*=288.5 Hz), 135.9, 134.3, 134.0, 129.0, 129.0, 128.2, 127.6, 118.0, 117.7, 51.8, 51.3 (d, *J* = 3.4 Hz), 47.4, 46.4 (d, *J* = 2.9 Hz), 32.7, 31.6.

^{19}F NMR (376 MHz, $CDCl_3$, both rotamers) δ -21.45, -21.94.

IR: 2941, 1780, 1642, 1218, 917, 698.

HRMS (ESI) m/z: $[M+H]^+$ calcd for $C_{12}H_{15}FNO$ 208.1138, found 208.1139.

In accordance with previous reports.[1]

2e. Benzyl(but-2-yn-1-yl)carbamic fluoride

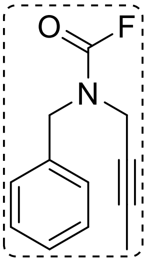

Obtained following the general procedure as a colourless liquid (57 mg, 0.4 mmol, 70 %)

^{1}H NMR (400 MHz, $CDCl_3$, both rotamers) δ 7.43 – 7.20 (m, 5H), 4.57 (d, *J* = 13.0 Hz, 2H), 3.99 (q, *J* = 2.4 Hz, 1H), 3.88 (q, *J* = 2.4 Hz, 1H), 1.83 (q, *J* = 2.4 Hz, 3H).

^{13}C NMR (101 MHz, $CDCl_3$, both rotamers) δ 147.44 (d, *J*=289.5 Hz), 147.22 (d, *J*=288.5 Hz), 135.3, 129.0, 128.9, 128.5, 128.3, 128.0, 81.71 (d, *J*=28.3 Hz), 72.3, 72.2, 50.6, 49.8 (d, *J* = 3.3 Hz), 37.2, 36.4 (d, *J* = 4.8 Hz), 3.5 (d, *J* = 5.4 Hz).

^{19}F NMR (376 MHz, $CDCl_3$, both rotamers) δ -22.03, -22.12.

IR: 1782, 1416, 1223, 1043, 941, 698.

HRMS (ESI) m/z: $[M+H]^+$ calcd for $C_{12}H_{13}FNO$ 206.0981, found 206.0983.

2f. *N*-methyl-N-(2-phenylethyl)carbamoyl fluoride

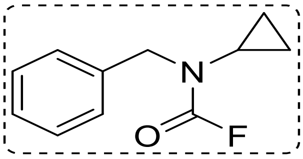

Obtained following the general procedure as a colourless liquid (55 mg, 0.4 mmol, 76%)

^{1}H NMR (400 MHz, $CDCl_3$, both rotamers) δ 7.40 – 7.14 (m, 5H), 3.53 – 3.43 (m, 2H), 2.94 – 2.81 (m, 5H).

^{13}C NMR (101 MHz, $CDCl_3$, both rotamers) δ 147.7 (d, *J*=285.6 Hz), 147.6 (d, *J*=287.5 Hz), 138.1, 137.8, 128.9, 128.9, 128.8, 127.0, 126.8, 51.9, 51.3 (d, *J*=2.9 Hz), 35.9, 35.2 (d, *J*=3.9 Hz), 34.7, 33.5.

^{19}F NMR (376 MHz, $CDCl_3$, both rotamers) δ -20.55, -23.11.

IR: 2935, 1780, 1603, 1402, 1301, 1114, 988, 699.

HRMS (ESI) m/z: $[M+H]^+$ calcd for $C_{10}H_{13}FNO$ 182.0981 found 182.0991

2g. 3,4-Dihydroisoquinoline-2(1H)-carbonyl fluoride

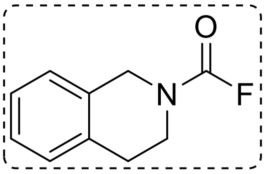

Obtained following the general procedure, using 2 equiv. of $Et_3N.3HF$, as a mixture of two rotamers as a colourless liquid (63.2 mg, 0.353 mmol, 87%).

^{1}**H NMR** (500 MHz, $CDCl_3$, both rotamers) δ = 7.40 – 6.97 (m, 4H), 4.68 (s, 2H), 3.76 (t, *J*=6.0 Hz, 1H), 3.74 (dt, *J*=6.0 Hz, 1H), 3.11 – 2.79 (m, 2H)

^{13}**C NMR** (101 MHz, $CDCl_3$, both rotamers) δ = 146.9 (d, *J*=282.3 Hz), 146.8 (d, *J*=281.9 Hz), 133.9, 133.7, 131.9, 131.6, 129.0, 128.8, 127.3, 127.2, 126.9, 126.9, 126.4, 126.2, 46.7, 46.2 (d, *J*=3.5 Hz), 42.8, 42.7, 28.9, 28.3.

^{19}**F NMR** (376 MHz, $CDCl_3$, both rotamers) δ = -20.13, -23.08.

IR: 3024, 2906, 2854, 1779, 1430, 1408, 1285, 1221, 743

HRMS (ESI) m/z: $[M+H]^+$ calcd for $C_{10}H_{11}NOF$ 180.0825, found 180.0839.

In accordance with previous reports.[1]

2h. **7-Chloro-3,4-dihydro-1H-isoquinoline-2-carbonyl fluoride**

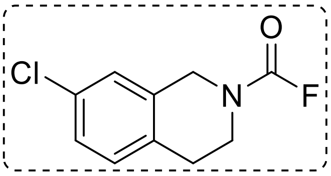

Obtained following the general procedure, using 2 equiv. of $Et_3N.3HF$, and after flash column chromatography (Hex:EtOAc 1:0 to 9:1) as a colourless liquid, as a mixture of two rotamers (63.9 mg, 0.299 mmol, 74%).

^{1}**H NMR** (500 MHz, $CDCl_3$, both rotamers) δ = 7.22 – 7.07 (m, 6H), 4.60 (s, 2H), 4.59 (s, 2H), 3.74 – 3.65 (m, 4H), 2.93 – 2.85 (m, 4H).

^{13}**C NMR** (101 MHz, $CDCl_3$, both rotamers) δ = 146.7 (d, *J*=288.9 Hz), 146.6 (d, *J*=287.8 Hz), 133.6, 133.3, 132.6, 132.6, 132.3, 132.1, 130.4, 130.2, 127.5, 127.4, 126.4, 126.2, 46.4, 45.9 (d, *J*=4.2 Hz), 42.7, 42.5 (d, *J*=3.7 Hz), 28.4, 27.8.

^{19}**F NMR** (376 MHz, $CDCl_3$, both rotamers) δ = -19.97, -23.04.

IR: 2901, 1780

HRMS (ESI) m/z: $[M+H]^+$ calcd for $C_{10}H_{10}NOFCl$ 214.0435, found 214.0403.

2i. 7-Bromo-3,4-dihydro-1H-isoquinoline-2-carbonyl fluoride

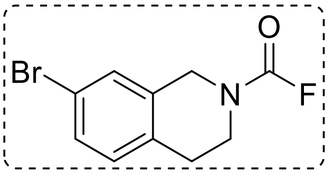

Obtained following the general procedure, using 2 equiv. of $Et_3N.3HF$, after filtration through a bed of silica (Hex:EtOAc 9:1) as a mixture of two rotamers as a colourless liquid (91.2 mg, 0.353 mmol, 87%).

^{1}**H NMR** (500 MHz, $CDCl_3$, both rotamers) δ = δ = 7.37 – 7.34 (m, 1H), 7.34 – 7.32 (m, 1H), 7.30 – 7.27 (m, 1H), 7.26 – 7.24 (m, 1H), 7.05 (d, *J*=8.2 Hz, 1H), 7.04 (d, *J*=8.2 Hz, 1H), 4.60 (s, 4H), 3.71 (t, *J*=6.0 Hz, 2H), 3.68 (t, J=6.0 Hz, 2H), 2.90-2.84 (m, 4H).

^{13}C NMR (126 MHz, $CDCl_3$, both rotamers) δ = 146.7 (d, *J*=287.5 Hz), 146.6 (d, *J*=286.6 Hz), 133.9, 133.6, 132.8, 132.6, 130.7, 130.5, 130.4, 130.3, 129.3, 129.1, 120.5, 120.4, 46.2, 45.8 (d, *J*=4.2 Hz), 42.6, 42.4 (d, *J*=3.6 Hz), 28.5, 27.9.

^{19}F NMR (471 MHz, $CDCl_3$, both rotamers) δ = -23.02, -19.94.

IR: 2920, 2850, 1779, 1429, 1406

HRMS (ESI) m/z: $[M+H]^+$ calcd for $C_{10}H_{10}NOFBr$ 257.9930, found 257.9941.

2j. *N*-benzyl-N-cyclopropylcarbamoyl fluoride

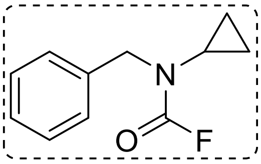

Obtained following the general procedure using a 13.4 $mA.cm^{-2}$ current density as a colourless liquid (55.6 mg, 0.4 mmol, 72%)

^{1}H NMR (400 MHz, $CDCl_3$, both rotamers) δ 7.42 – 7.21 (m, 4H), 4.45 (d, *J* = 15.9 Hz, 2H), 2.64 – 2.46 (m, 1H), 0.89 – 0.73 (m, 4H).

^{13}C NMR (101 MHz, $CDCl_3$, both rotamers) δ 149.2 (d, *J*=291.4 Hz), 147.9 (d, *J*=292.4 Hz), 136.4, 128.9, 128.8, 128.1, 128.0, 127.5, 52.6, 30.5, 29.1, 7.9, 7.7.

^{19}F NMR (376 MHz, $CDCl_3$, both rotamers) δ -14.61, -15.63.

IR: 3031, 1780, 1399, 1261, 874, 698.

HRMS (ESI) m/z: $[M+H]^+$ calcd for $C_{11}H_{13}FNO$ 194.0981, found 194.0984

2k. bis-(2-chloro-ethyl)-carbamoyl fluoride

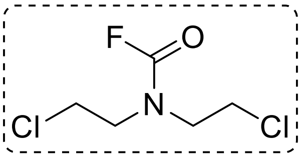

Obtained following the general procedure after flash column chromatography (Hex:EtOAc 1:0 to 9:1) as a colourless liquid (45 mg, 0.4 mmol, 60%).

^{1}H NMR (500 MHz, $CDCl_3$) δ 3.77 – 3.62 (m, 8H).

^{13}C NMR (101 MHz, $CDCl_3$) δ 147.08 (d, *J* = 291.5 Hz), 51.7, 51.2, 41.7, 41.0.

^{19}F NMR (376 MHz, $CDCl_3$) δ -19.02.

IR: 1778, 1415, 1183, 1066, 949.

HRMS (ESI) m/z: $[M+H]^+$ calcd for $C_5H_9Cl_2FNO$ 188.0045, found 188.0071

2l. *N*-methyl-N-phenylcarbamoyl fluoride

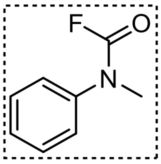

Obtained following the general procedure as a colourless liquid (14 mg, 0.4 mmol, 23 %)

^{1}H NMR (400 MHz, $CDCl_3$, both rotamers) δ 7.46 – 7.17 (m, 5H), 3.35 (s, 3H).

^{13}C NMR (101 MHz, $CDCl_3$, both rotamers) δ 146.5 (d, *J*=287.1 Hz), 141.7, 140.8, 129.5, 129.4, 127.8, 127.2, 125.7, 124.7, 39.0, 38.0.

^{19}F NMR (376 MHz, $CDCl_3$, both rotamers) δ -15.83, -15.98.

IR: 1781, 1598, 1369, 1115, 698,

HRMS (ESI) m/z: $[M+H]^+$ calcd for C_8H_9FNO 154.0668, found 154.0669.

In accordance with previous reports.[4]

2m. Diphenylcarbamic fluoride

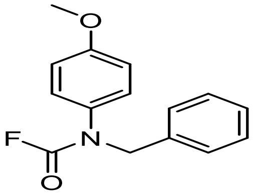

Obtained following the general procedure as a colourless liquid (9 mg, 0.4 mmol, 10 %)

^{1}H NMR (400 MHz, $CDCl_3$) δ 7.47 – 7.19 (m, 10H).

^{13}C NMR (101 MHz, $CDCl_3$) δ 145.4 (d, *J*=290.9 Hz), 129.6, 128.1, 127.2, 125.5.

^{19}F NMR (376 MHz, $CDCl_3$) δ -8.77.

IR: 3068, 1796, 1591, 1493, 1347, 1187, 988, 695

HRMS (ESI) m/z: $[M+H]^+$ calcd for $C_{13}H_{11}FNO$ 216.0825, found 216.0854.

In accordance with previous reports.[5]

2n. *N*-benzyl-N-(4-methoxyphenyl)carbamoyl fluoride

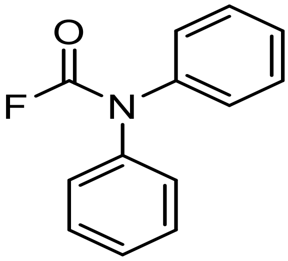

Obtained following the general procedure as a colourless liquid (83 mg, 0.4 mmol, 80 %)

^{1}H NMR (400 MHz, $CDCl_3$, both rotamers) δ 7.37 – 7.28 (m, 3H), 7.24 – 7.07 (m, 2H), 6.99 – 6.88 (m, 2H), 6.88 – 6.77 (m, 2H), 4.77 (s, 2H), 3.77 (s, 3H).

^{13}C NMR (101 MHz, $CDCl_3$, both rotamers) δ 159.2, 159.0, 147.3 (d, *J*=287.1 Hz), 147.1 (d, *J*=291.4 Hz), 136.1, 135.9, 133.3, 132.0, 128.9, 128.8, 128.7, 128.3, 128.2, 128.0, 127.7, 114.7, 114.6, 55.9 (d, *J*=1.9 Hz), 55.5.

^{19}F NMR: (376 MHz, $CDCl_3$, both rotamers) δ -14.99, -18.27.

IR: 2937, 1779, 1608, 1392, 1248, 835, 706.

HRMS (ESI) m/z: $[M+H]^+$ calcd for $C_{15}H_{15}FNO_2$ 260.1087, found 260.1060.

In accordance with previous reports.[1]

2o. Ethyl 1-(fluorocarbonyl)piperidine-4-carboxylate

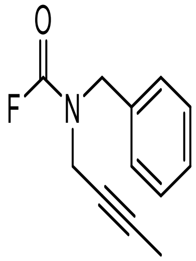

Obtained following the general procedure after flash column chromatography (Hex:EtOAc 1:0 to 8:2) as a colourless oil (81.75 mg, 0.35 mmol, 88%)

^{1}H NMR (400 MHz, $CDCl_3$) δ = 4.15 (q, *J*=7.1 Hz, 2H), 3.94 (dtd, *J*=14.8, 3.8, 1.5 Hz, 1H), 3.86 (dt, *J*=13.8, 4.4 Hz, 1H), 3.15 – 3.00 (m, 2H), 2.51 (tt, *J*=10.5, 4.0 Hz, 1H), 2.05 – 1.87 (m, 2H), 1.74 (dqd, *J*=14.2, 10.4, 4.2 Hz, 2H), 1.26 (t, *J*=7.1 Hz, 3H).

^{13}C NMR (101 MHz, $CDCl_3$) δ = 173.8, 146.5 (d, *J*=286.5 Hz), 60.9, 44.4, 44.1 (d, *J*=4.5 Hz), 40.3, 27.8, 27.4, 14.3.

^{19}F NMR (376 MHz, $CDCl_3$) δ = -24.40.

IR: 1782, 1727, 1178,

HRMS (ESI) m/z: $[M+H]^+$ calcd for $C_9H_{15}NO_3F$ 204.1036, found 204.1055.

In accordance with previous reports.[6]

2p. 1,1-Dioxo-1λ^6-thiomorpholine-4-carbonyl fluoride

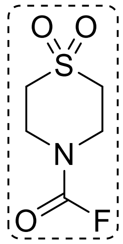

Obtained following the general procedure as a white solid (33 mg, 0.18 mmol, 46%)

^{1}H NMR (400 MHz, DMSO-d_6) δ = 3.88 – 3.72 (m, 4H), 3.38 – 3.19 (m, 4H).

^{13}C NMR (101 MHz, DMSO-d_6) δ = 145.3 (d, *J*=286.3 Hz), 50.3, 50.1, 43.7, 43.4 (d, *J*=4.6 Hz).

^{19}F NMR (376 MHz, DMSO-d_6) δ = -23.96.

IR:1777, 1433, 1319, 1123

HRMS (ESI) m/z: $[M-H]^+$ calcd for $C_5H_7NO_3FS$ 180.0131, found 180.0176.

2q. Tert-butyl 4-(fluorocarbonyl)piperazine-1-carboxylate

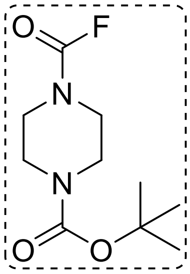

Obtained following the general procedure using 3 F.mol^{-1} after flash column chromatography (Hex:EtOAc 1:0 to 7:3) as a white solid (58 mg, 0.25 mmol, 62%).

^{1}H NMR (400 MHz, $CDCl_3$) δ = 3.81 – 3.14 (m, 8H), 1.46 (s, 9H).

^{13}C NMR (101 MHz, $CDCl_3$) δ = 154.4, 146.4 (d, *J*=287.0 Hz), 80.8, 44.7 (d, *J*=3.9 Hz), 28.4.

^{19}F NMR (376 MHz, $CDCl_3$) δ = -23.43.

IR: 1788, 1688, 1235, 1163

HRMS (ESI) m/z: $[M+H]^+$ calcd for $C_{10}H_{18}N_2O_3F$ 233.1301, found 233.1313.

In accordance with previous reports.[5]

2r. Morpholine-4-carbonyl fluoride

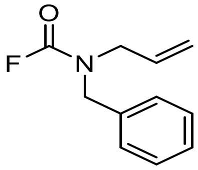

Obtained following the general procedure as a colourless liquid (18.6 mg, 0.4 mmol, 35%)

^{1}H NMR (400 MHz, $CDCl_3$) δ 3.75 – 3.64 (m, 4H), 3.51 – 3.41 (m, 4H).

^{13}C NMR (101 MHz, $CDCl_3$) δ 146.3 (d, *J*=286.6 Hz), 144.9, 66.2, 45.0.

^{19}F NMR (376 MHz, $CDCl_3$) δ -24.23.

IR: 2864, 1778, 1427, 1236, 1115, 848.

HRMS (ESI) m/z: $[M+H]^+$ calcd for $C_5H_9FNO_2$ 134.0617, found 134.0619

2s. (3-(10,11-Dihydro-5H-dibenzo[a,d][7]annulen-5-ylidene)propyl)(methyl)carbamic fluoride

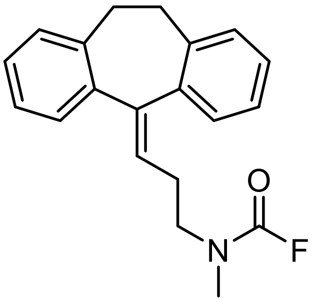

Obtained following the general procedure as a colourless liquid (24 mg, 0.4 mmol, 19%) in acetonitrile solvent.

^{1}H NMR (500 MHz, $CDCl_3$, both rotamers) δ 7.31 – 7.01 (m, 8H), 5.87 – 5.77 (m, 1H), 3.42 – 3.27 (m, 4H), 3.01 – 2.92 (m, 1H), 2.79 (d, *J* = 14.8 Hz, 4H), 2.57 – 2.30 (m, 2H).

^{13}C NMR (126 MHz, $CDCl_3$, both rotamers) δ 147.8 (d, *J*=286.8 Hz), 147.7 (d, *J*=285.6 Hz), 146.2, 145.7, 140.8, 140.7, 139.7, 139.6, 139.5, 139.5, 137.2, 137.1, 130.3, 130.2, 128.7, 128.6, 128.3, 128.3, 128.1, 128.0, 127.9, 127.8, 127.5, 127.4, 126.5, 126.2, 126.1, 126.0, 49.7, 49.2 (d, *J*=2.3 Hz), 35.2, 34.2 (d, *J*=3.6 Hz), 33.8, 32.0 (d, *J*=3.6 Hz), 28.0, 27.2.

^{19}F NMR (376 MHz, $CDCl_3$, both rotamers) δ -20.61, -23.06.

IR: 2923, 1782, 1402, 1122, 995, 748.

HRMS (ESI) m/z: $[M+H]^+$ calcd for $C_{20}H_{21}NOF$ 310.1607, found 310.1611

In accordance with previous reports.[5]

2t. 4-(Trifluoromethyl)piperidine-1-carbonyl fluoride*

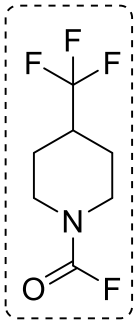

Obtained following the general procedure as a colourless liquid (65 mg, 0.4 mmol, 82%).

^{1}H NMR (400 MHz, $CDCl_3$) δ 4.15 (d, *J* = 13.6 Hz, 1H), 4.04 (d, *J* = 13.3 Hz, 1H), 3.01 – 2.83 (m, 2H), 2.23 (dqq, *J* = 15.8, 8.0, 3.9 Hz, 1H), 2.00 – 1.87 (m, 2H), 1.58 (pd, *J* = 12.7, 4.7 Hz, 2H).

^{13}C NMR (101 MHz, $CDCl_3$) δ 146.2 (d, *J* = 286.6 Hz), 126.8 (q, *J* = 278.1 Hz), 43.9, 43.7 (d, *J* = 4.8 Hz), 40.0 (q, *J* = 27.9 Hz), 24.5 (d, *J* = 2.9 Hz), 24.0 (d, *J* = 2.4 Hz).

^{19}F NMR (376 MHz, $CDCl_3$) δ -24.24, -73.83 (d, *J* = 8.9 Hz, 3F).

IR: 1779, 1432, 1336, 1255, 1140.

HRMS (ESI) m/z: $[M+H]^+$ calcd for $C_7H_{10}F_4NO$ 200.0699, found 200.0719

*Possibly low boiling.

2u. 4-Cyanopiperidine-1-carbonyl fluoride

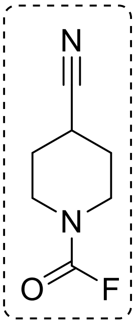

Obtained following the general procedure as a colourless liquid (53 mg, 0.4 mmol, 88%).

^{1}H NMR (500 MHz, $CDCl_3$) δ 3.72 – 3.59 (m, 2H), 3.58 – 3.44 (m, 2H), 2.96 – 2.88 (m, 1H), 2.05 – 1.85 (m, 4H).

^{13}C NMR (101 MHz, $CDCl_3$) δ 146.1 (d, *J* = 287.1 Hz), 120.25, 43.0, 42.7 (d, *J* = 4.3 Hz), 28.3, 27.8, 25.8.

^{19}F NMR (376 MHz, $CDCl_3$) δ -24.12.

IR: 2924, 2242, 1780, 1430, 1043.

HRMS (ESI) m/z: $[M+H]^+$ calcd for $C_7H_{10}FN_2O$ 157.0777 , found 157.0762

2v. 4-Phenylpiperidine-1-carbonyl fluoride

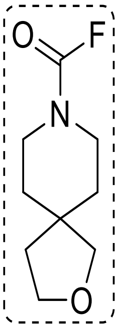

Obtained following the general procedure after flash column chromatography (Hex:EtOAc 1:0 to 7:3) as a white solid (55 mg, 0.26 mmol, 66%)

^{1}H NMR (400 MHz, $CDCl_3$) δ = 7.36 – 7.30 (m, 2H), 7.29 – 7.16 (m, 3H), 4.28 – 4.15 (m, 1H), 4.15 – 3.99 (m, 1H), 3.13 – 2.95 (m, 2H), 2.71 (tt, *J*=12.2, 3.7 Hz, 1H), 1.99 – 1.87 (m, 2H), 1.82 – 1.64 (m, 2H).

^{13}C NMR (101 MHz, $CDCl_3$) δ = 146.6 (d, *J*=285.4 Hz), 144.8, 128.8, 126.8, 126.8, 45.9, 45.6 (d, *J*=4.5 Hz), 42.2, 33.1, 32.6.

^{19}F NMR (376 MHz, $CDCl_3$) δ = -24.49.

IR: 1776, 1428, 1215, 743

HRMS (ESI) m/z: $[M+H]^+$ calcd for $C_{12}H_{15}FNO$ 208.1138, found 208.1150.

In accordance with previous reports.[4]

2w. 6-Azaspiro[2.5]octane-6-carbonyl fluoride

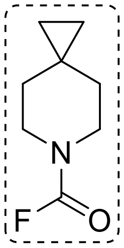

Obtained following the general procedure after flash column chromatography (Hex:EtOAc 1:0 to 9:1) as a colourless liquid (38 mg, 0.4 mmol, 60%).

^{1}H NMR (400 MHz, $CDCl_3$) δ = 3.53 – 3.42 (m, 4H), 1.45 – 1.35 (m, 4H), 0.36 (s, 4H).

^{13}C NMR (101 MHz, $CDCl_3$) δ = 146.7 (d, *J*=285.1 Hz), 45.3 (d, *J*=1.9 Hz), 45.0 (d, *J*=4.3 Hz), 34.9, 34.3, 17.2, 11.4.

^{19}F NMR (376 MHz, $CDCl_3$) δ -24.53.

IR: 2918, 1640, 1426, 1226, 1101.

HRMS (ESI) m/z: $[M+H]^+$ calcd for $C_8H_{13}FNO$ 158.0981, found 158.0995

2x. 2-Oxa-8-azaspiro[4.5]decane-8-carbonyl fluoride

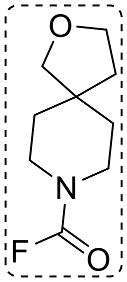

Obtained following the general procedure as a colourless liquid (53 mg, 0.4 mmol, 70%).

^{1}H NMR (400 MHz, $CDCl_3$) δ 3.73 (t, *J* = 7.1 Hz, 2H), 3.50 – 3.14 (m, 6H), 1.70 – 1.59 (m, 2H), 1.49 (m, 4H).

^{13}C NMR (126 MHz, $CDCl_3$) δ = 146.3 (d, *J*=285.6 Hz), 77.3, 67.2, 43.1 (d, *J*=1.8 Hz), 42.8, 42.7, 41.6, 36.7, 34.4, 33.9.

^{19}F NMR (376 MHz, $CDCl_3$) δ -24.66.

IR: 2929, 1779, 1641, 1430, 1240, 1039.

HRMS (ESI) m/z: $[M+H]^+$ calcd for $C_9H_{15}FNO_2$ 188.1087, found 188.1108

2y. 4-(Tetrahydrofuran-2-carbonyl)piperazine-1-carbonyl fluoride

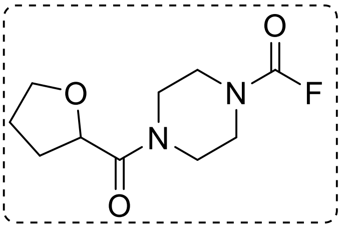

Obtained following the general procedure as a colourless liquid (68 mg, 0.4 mmol, 74%).

^{1}H NMR (500 MHz, $CDCl_3$, both rotamers) δ = 4.56 (q, *J*=6.2 Hz, 1H), 4.99 – 3.69 (m, 4H), 3.69 – 3.25 (m, 6H), 2.51 – 2.25 (m, 1H), 2.09 – 1.83 (m, 3H).

^{13}C NMR (101 MHz, $CDCl_3$, both rotamers) δ = 170.0, 169.9, 146.3 (d, *J*=286.9 Hz), 146.3 (d, *J*=286.4 Hz), 76.2, 76.2, 69.2, 45.2, 45.0, 44.7, 41.7, 41.5, 28.0 (d, *J*=6.0 Hz), 25.9.

^{19}F NMR (376 MHz, $CDCl_3$, both rotamers) δ = -24.1.

IR: 2875, 1787, 1651, 1432, 1225, 1020.

HRMS (ESI) m/z: $[M+H]^+$ calcd for $C_{10}H_{16}FN_2O_3$ 231.1145, found 231.1196

2z. 4-Ethynylpiperidine-1-carbonyl fluoride

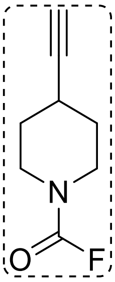

Obtained following the general procedure as a colourless liquid (38 mg, 0.4 mmol, 62%).

^{1}H NMR (400 MHz, $CDCl_3$) δ 3.63 (dddd, *J* = 14.3, 11.0, 8.0, 3.7 Hz, 2H), 3.38 (tdd, *J* = 14.0, 7.5, 3.8 Hz, 2H), 2.69 (tt, *J* = 6.7, 3.4 Hz, 1H), 2.13 (d, *J* = 2.5 Hz, 1H), 1.83 (ddq, *J* = 15.8, 7.9, 3.8 Hz, 2H), 1.76 – 1.63 (m, 2H).

^{13}C NMR (101 MHz, $CDCl_3$) δ 146.5 (d, *J* = 285.6 Hz), 85.1, 70.7, 43.2, 42.9 (d, *J* = 4.3 Hz), 31.0, 30.5, 26.0.

^{19}F NMR (376 MHz, $CDCl_3$) δ -24.66.

IR: 3256, 2964, 1769, 1432, 1215, 1101, 1017.

HRMS (ESI) m/z: $[M+H]^+$ calcd for $C_8H_{11}FNO$ 156.0825 , found 156.0839

Cyclic Voltammetry Studies

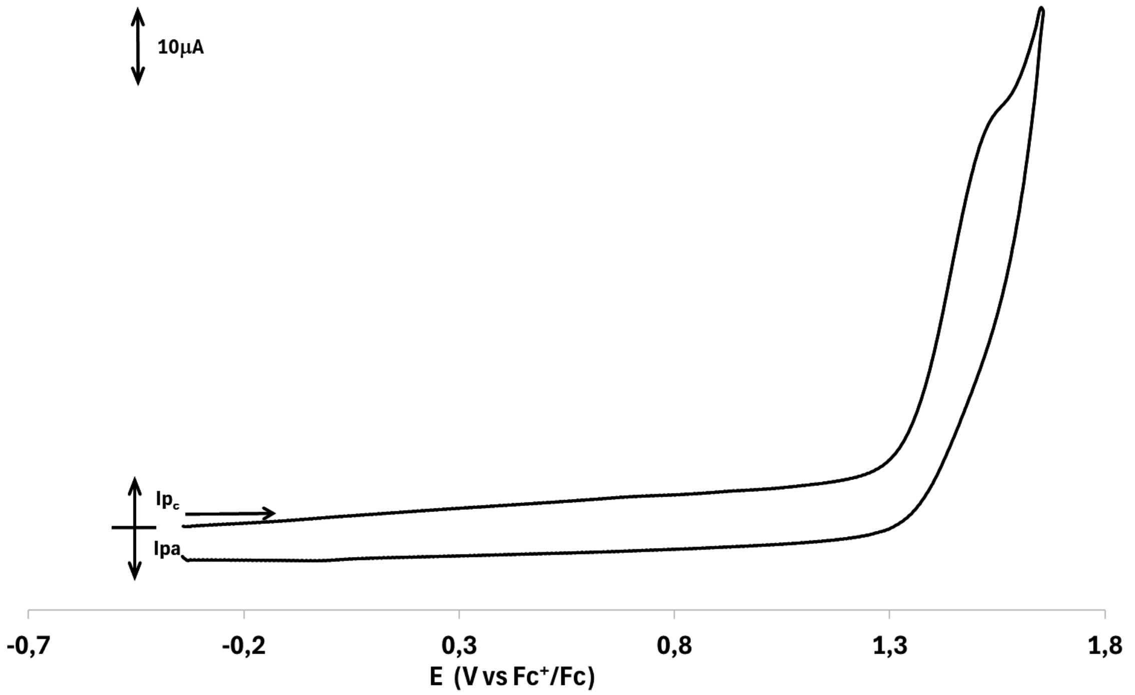

Figure S1: Cyclic voltammogram on glassy carbon (diameter 3mm); Pt wire as the counter electrode; Ag/AgCl as pseudo reference, of a 2mM solution of oxamic acid **1a** in CH_2Cl_2 containing 0.1M of $[NBu_4][PF_6]$, at 0.5 $V.s^{-1}$ at room temperature. IUPAC plotting convention.

Oxamic acid **1a** exhibits a chemically non-reversible oxidation at E_{pa}=1.54 vs Fc^+/Fc

NMR Spectra

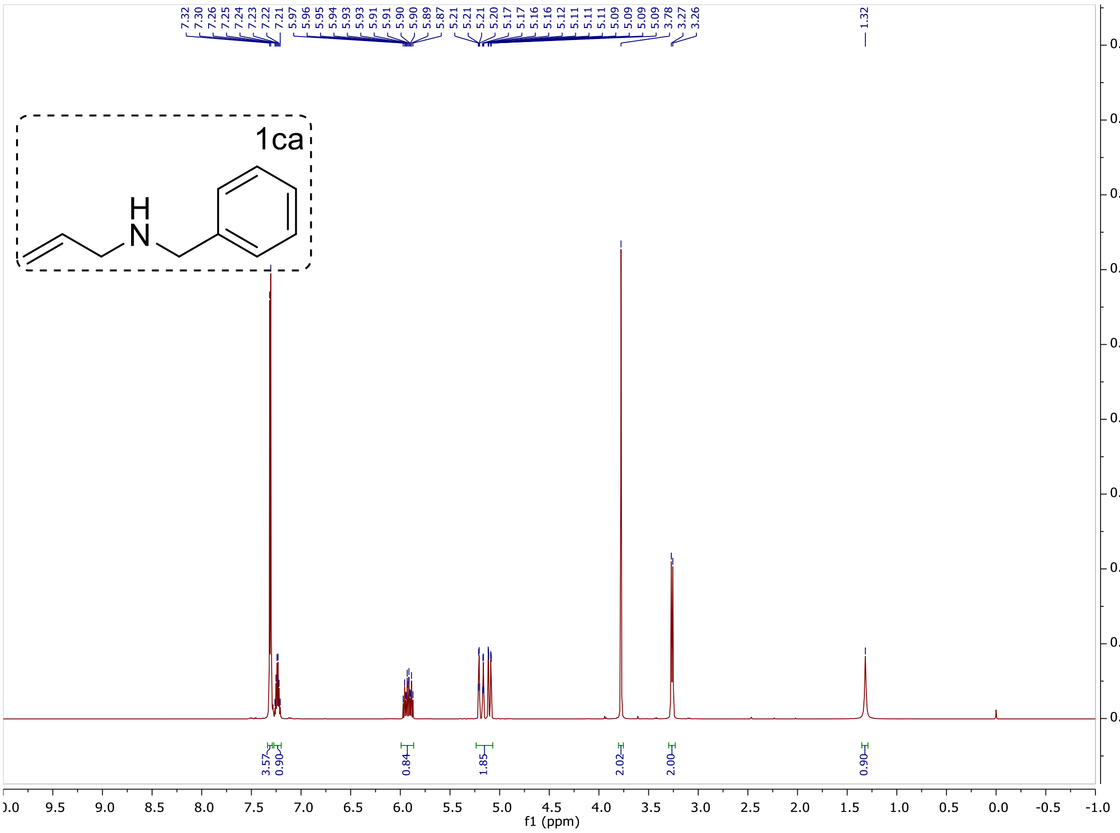

^{1}H NMR of 1ca in $CDCl_3$ (400 MHz)

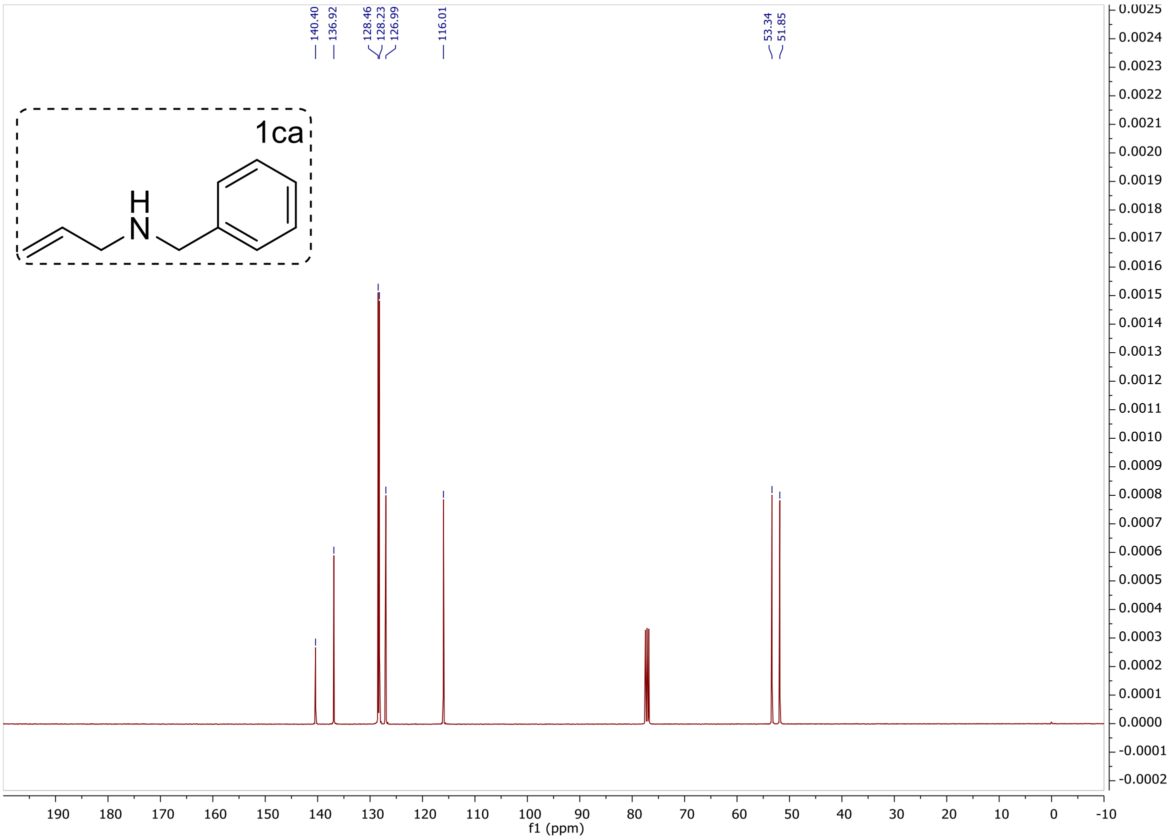

^{13}C NMR of 1ca in $CDCl_3$ (101 MHz)

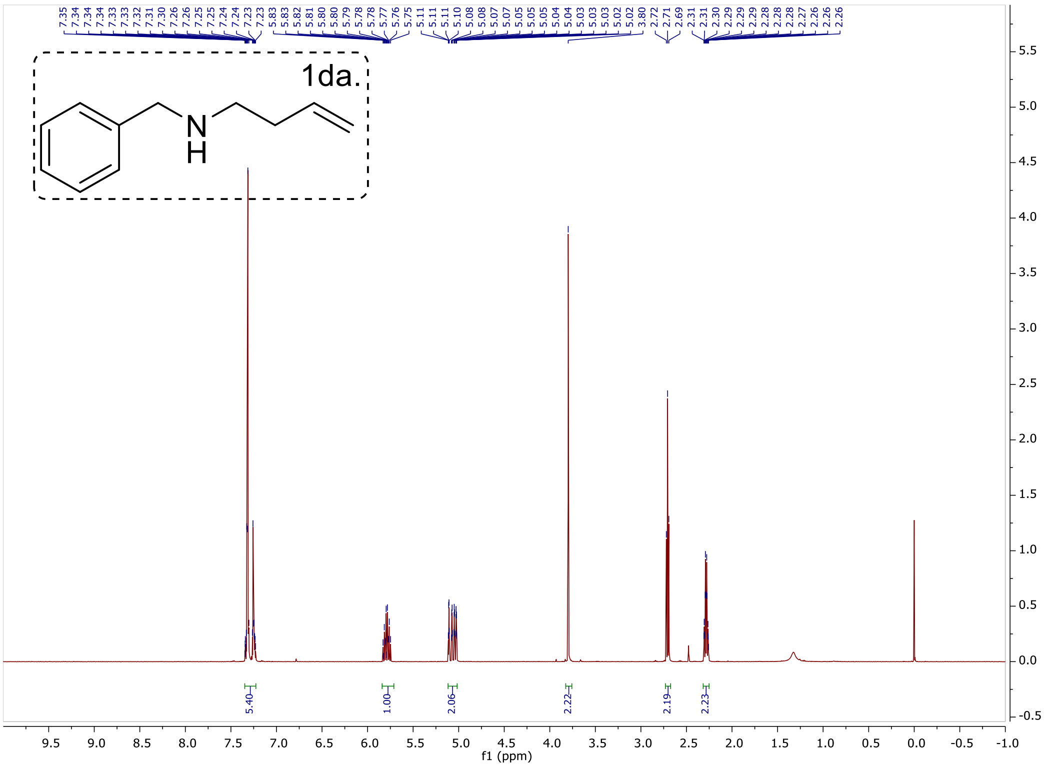

^{1}H NMR of 1da in $CDCl_3$ (500 MHz)

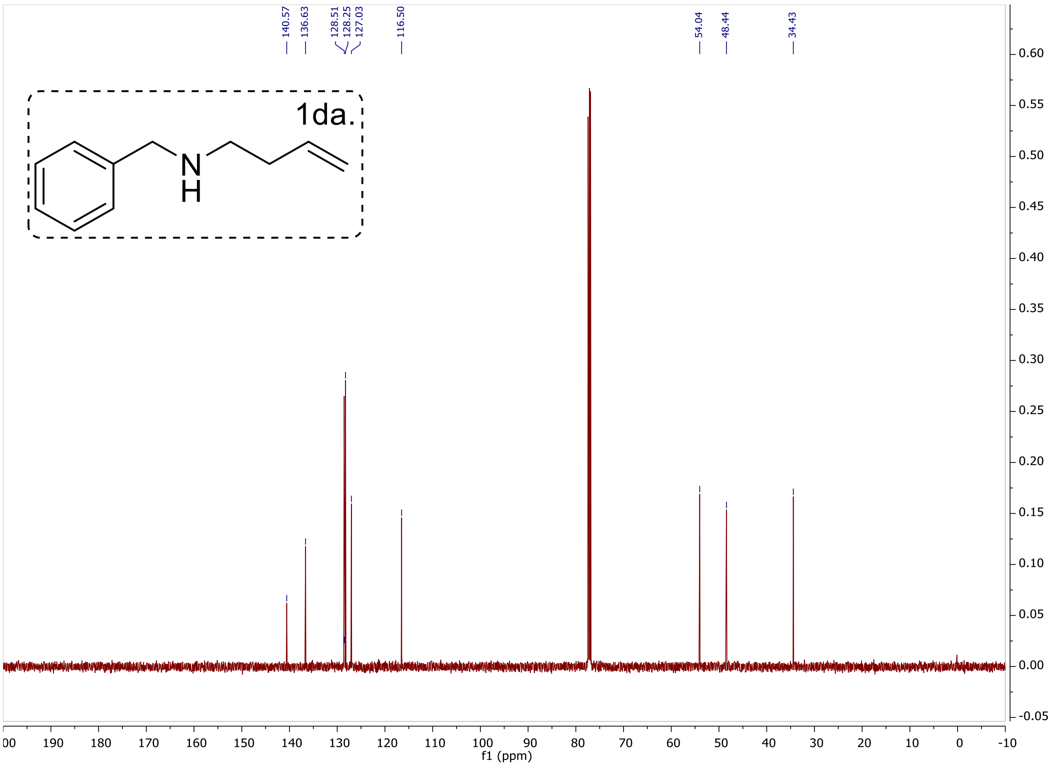

^{13}C NMR of 1da in $CDCl_3$ (126 MHz)

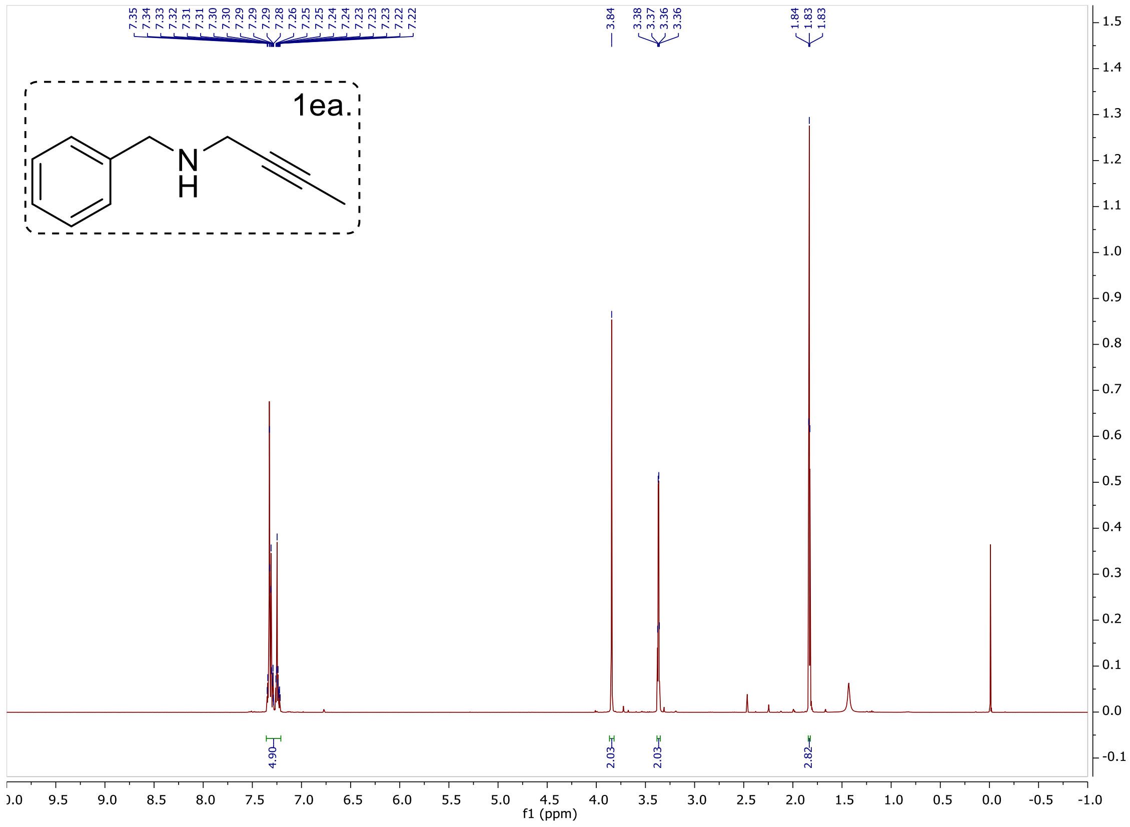

^{1}H NMR of 1ea in $CDCl_3$ (400 MHz)

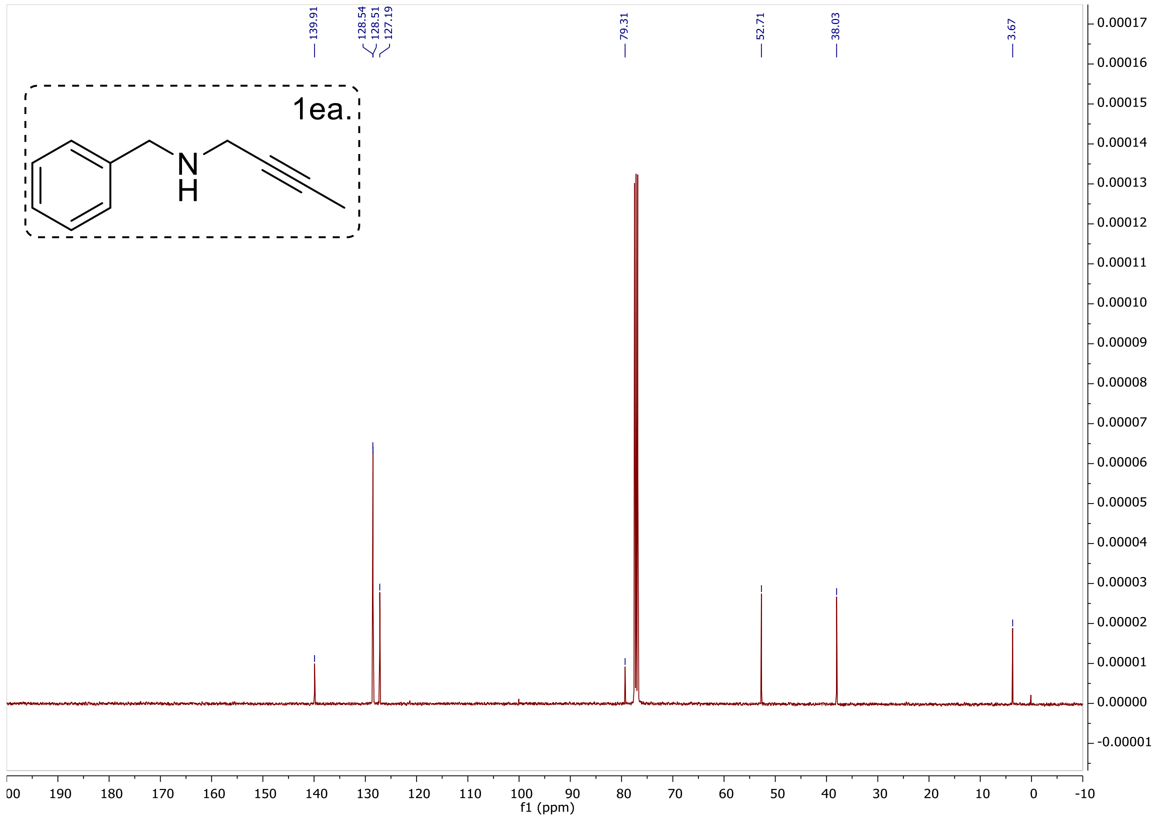

^{13}C NMR of 1ea in $CDCl_3$ (101 MHz)

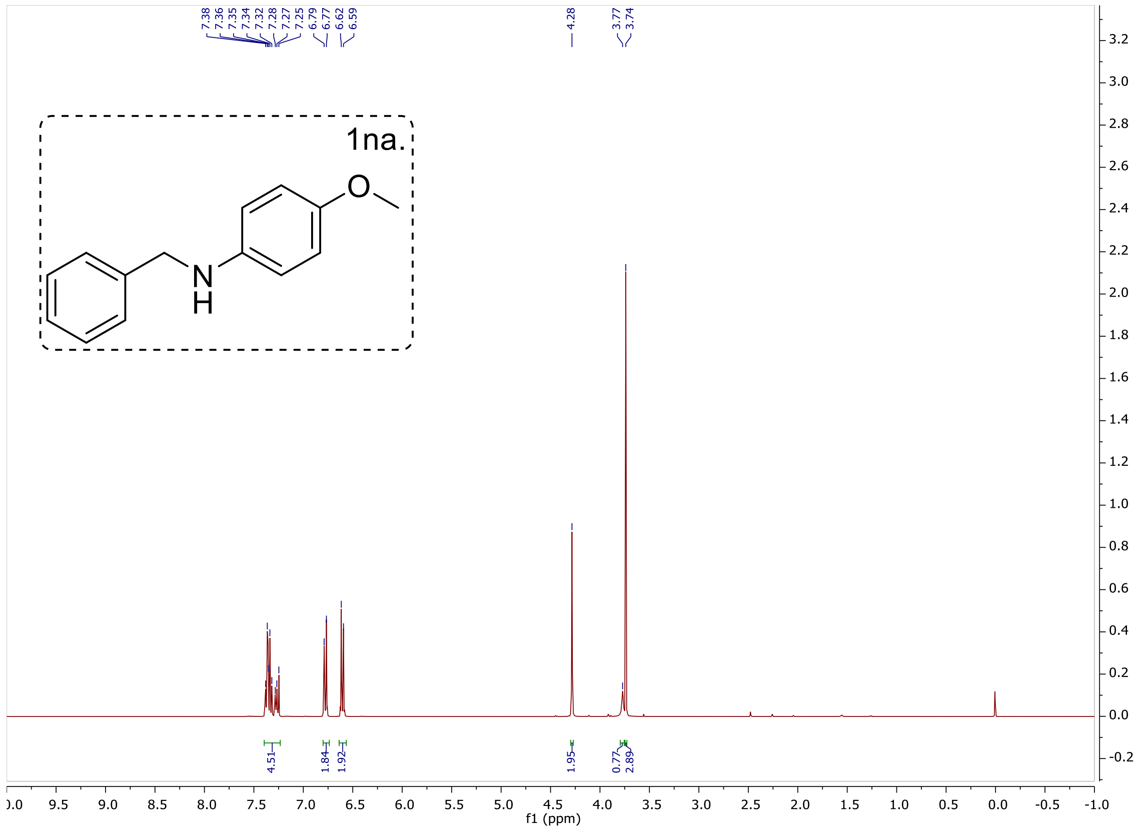

^{1}H NMR of 1na in $CDCl_3$ (400 MHz)

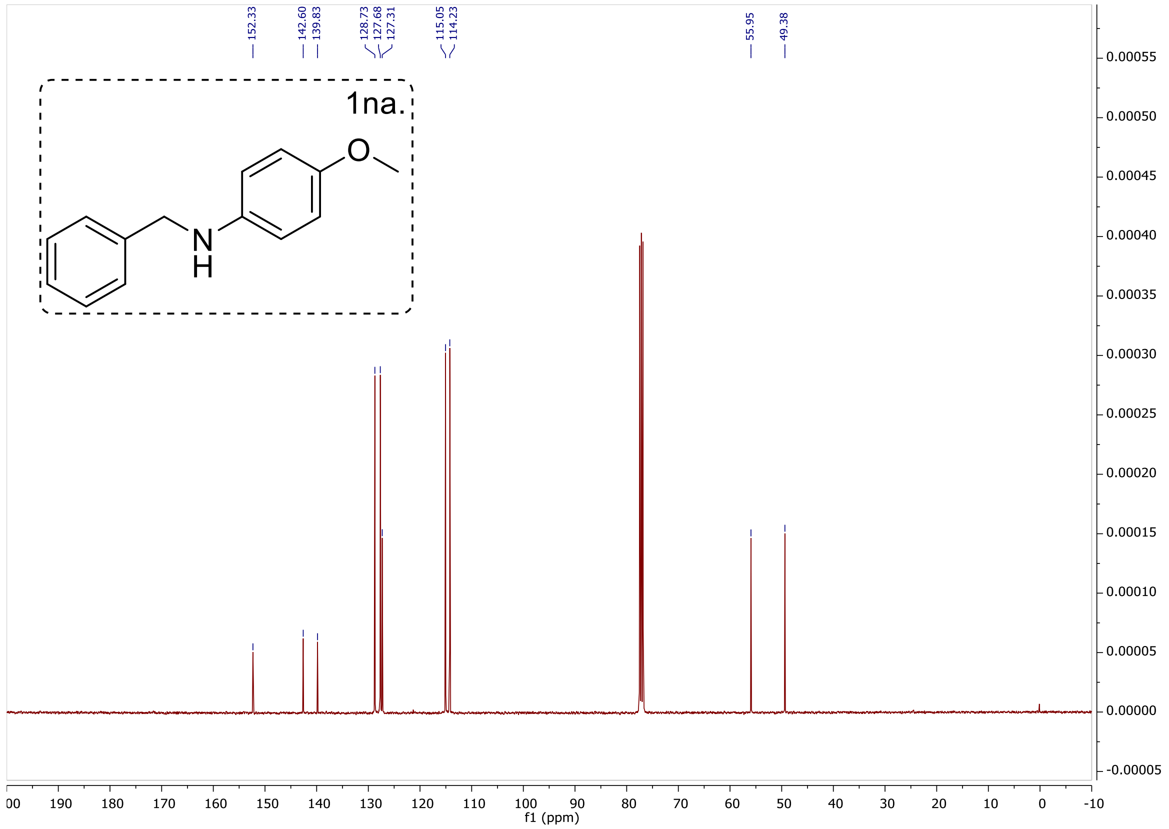

^{13}C NMR of 1na in $CDCl_3$ (101 MHz)

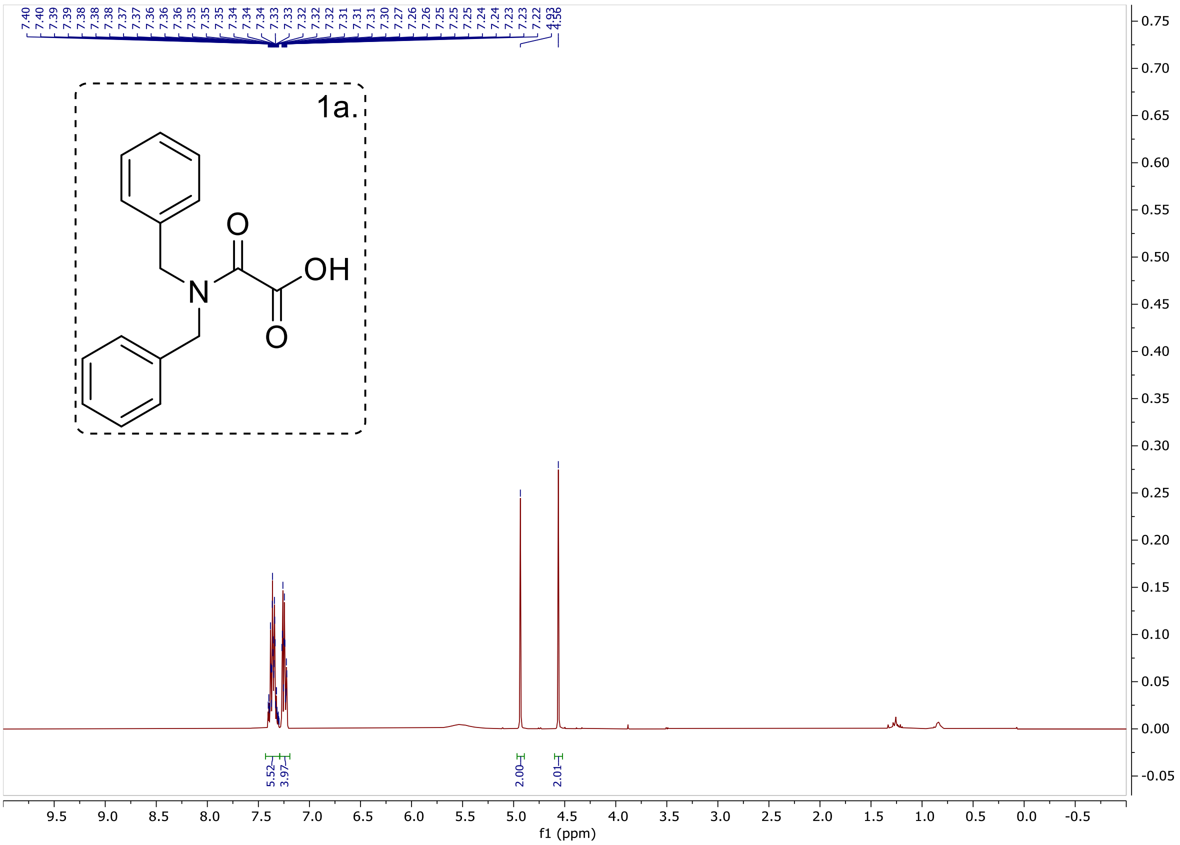

^{1}H NMR of 1a in $CDCl_3$ (400 MHz)

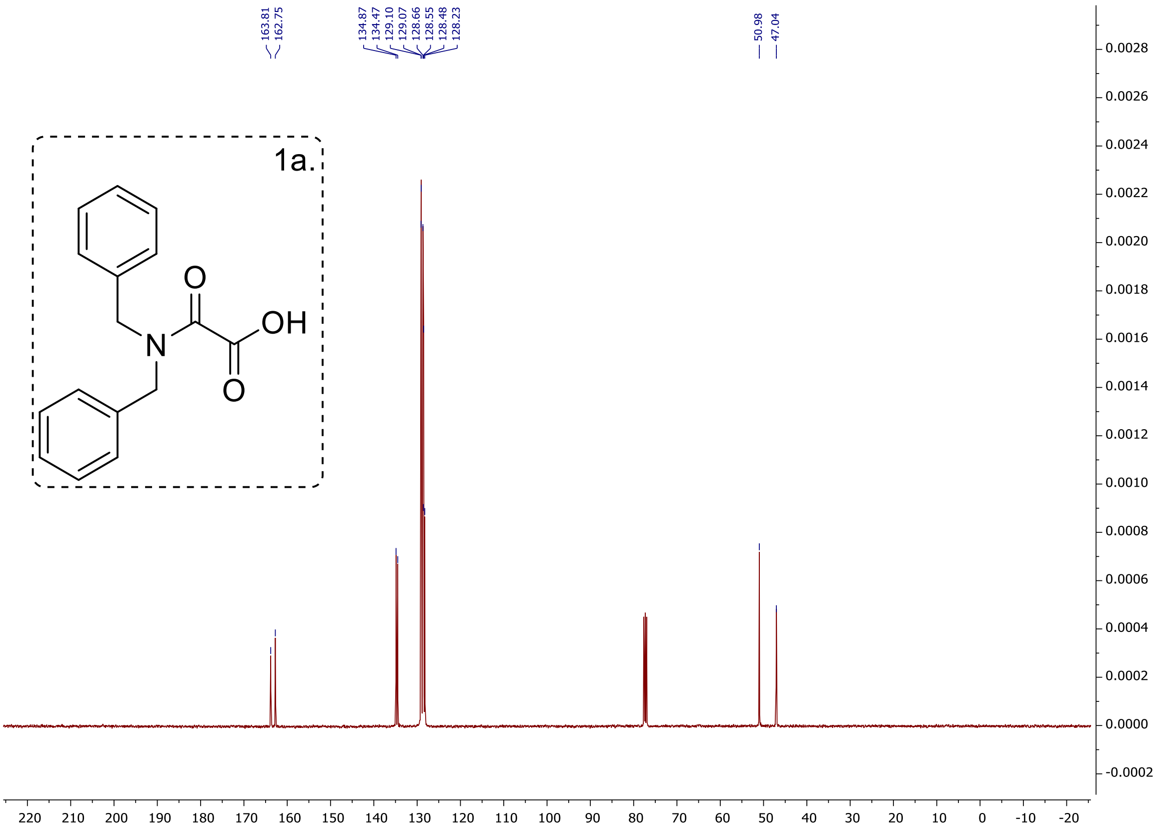

^{13}C NMR of 1a in $CDCl_3$ (101 MHz)

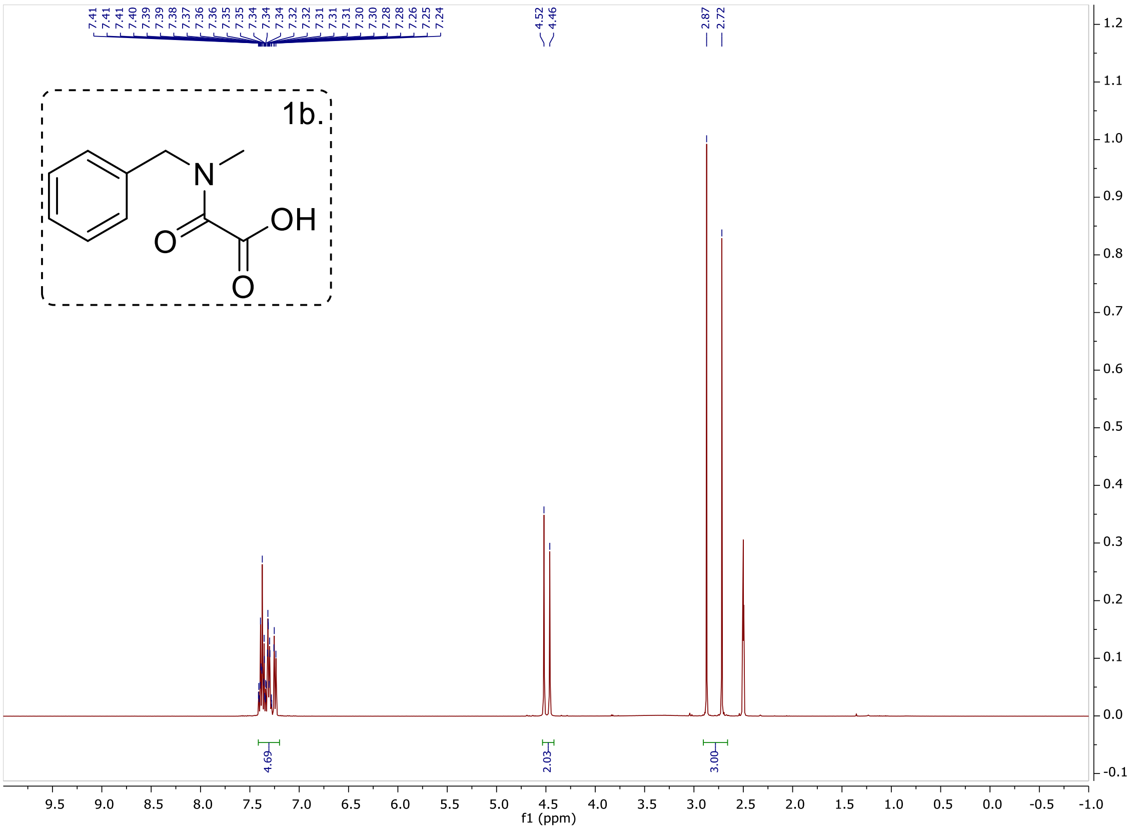

^{1}H NMR of 1b in DMSO (400 MHz)

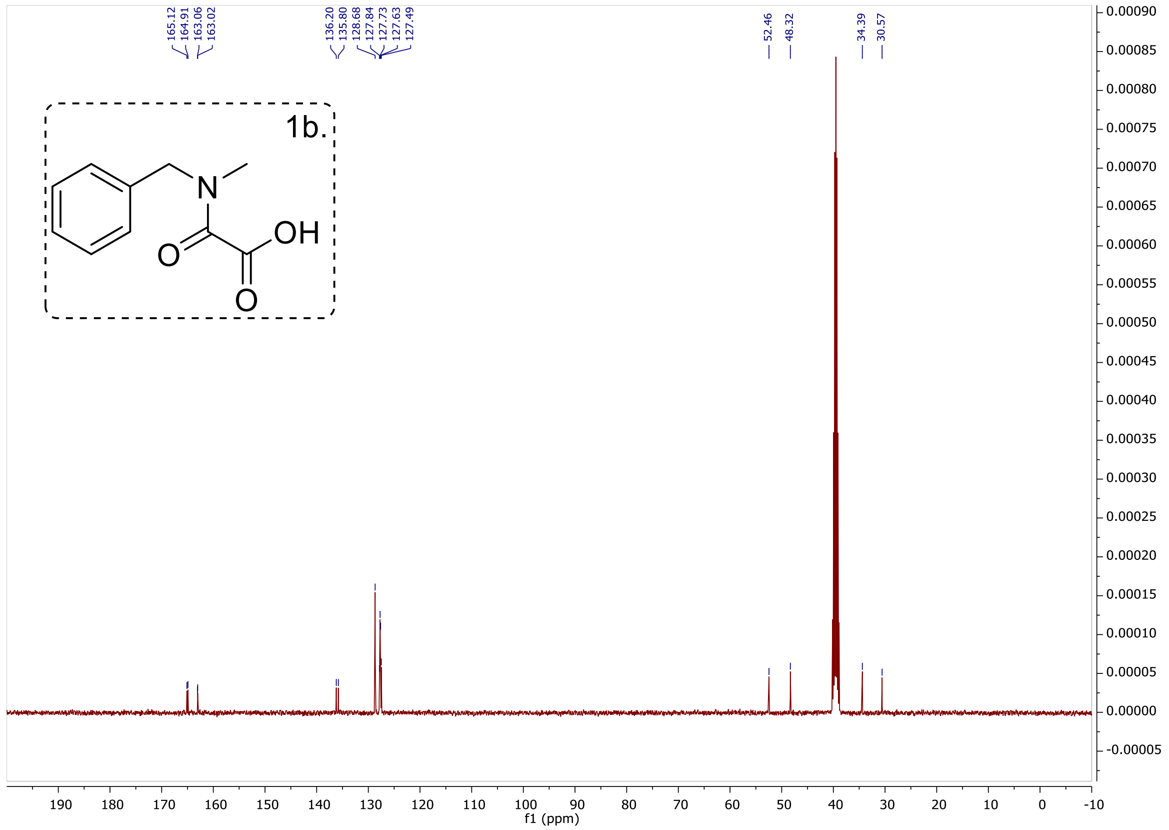

^{13}C NMR of 1b in DMSO (101 MHz)

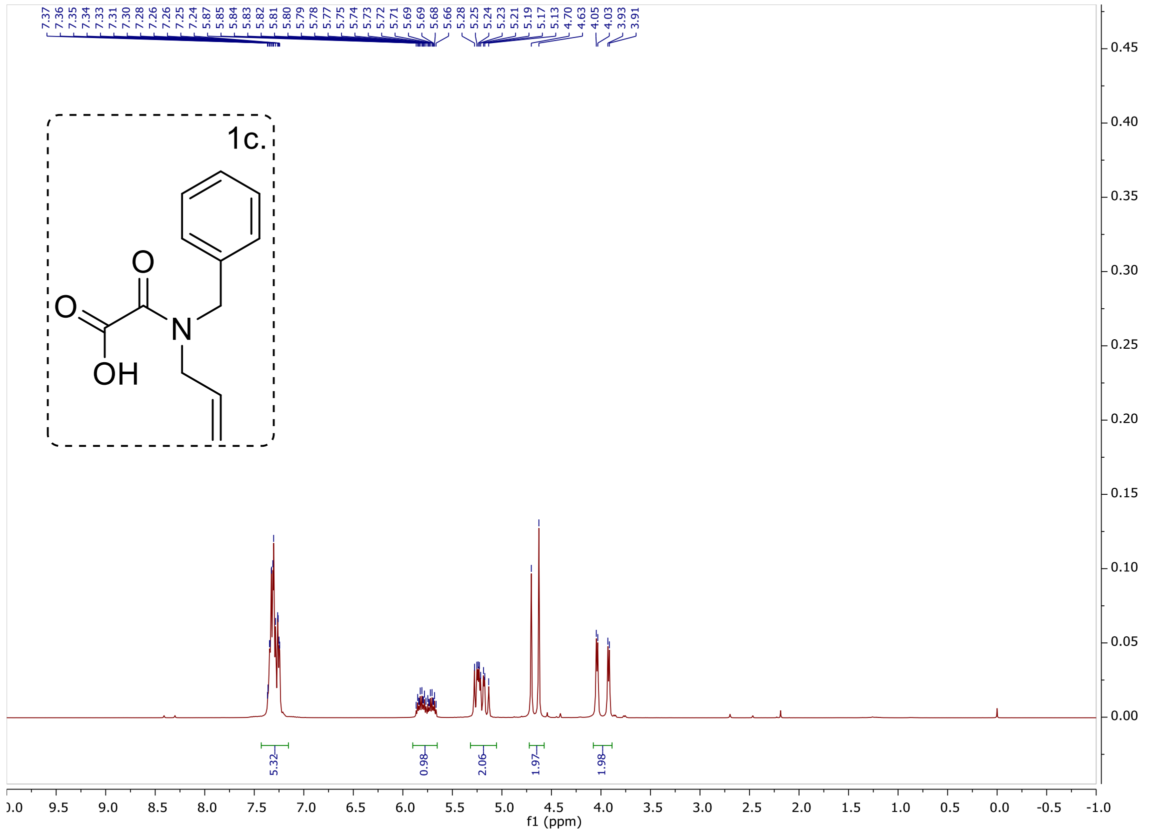

^{1}H NMR of 1c in $CDCl_3$ (400 MHz)

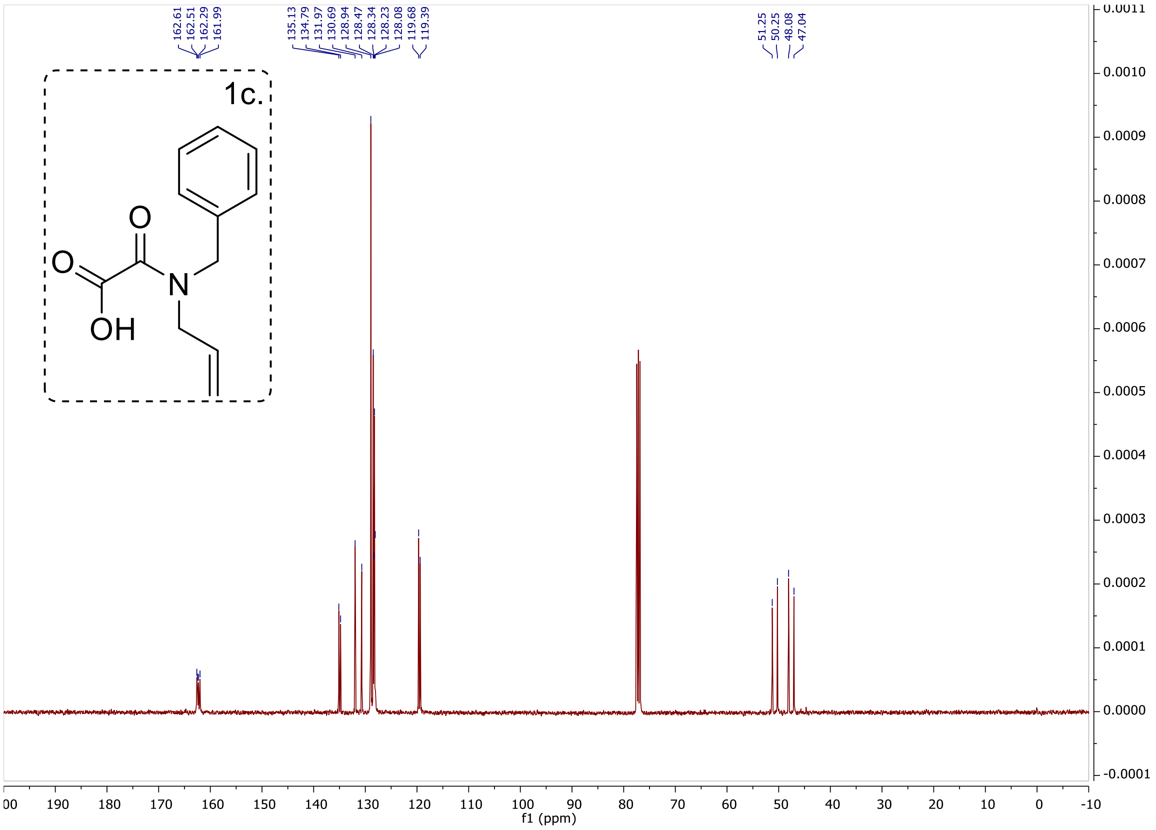

^{13}C NMR of 1c in $CDCl_3$ (101 MHz)

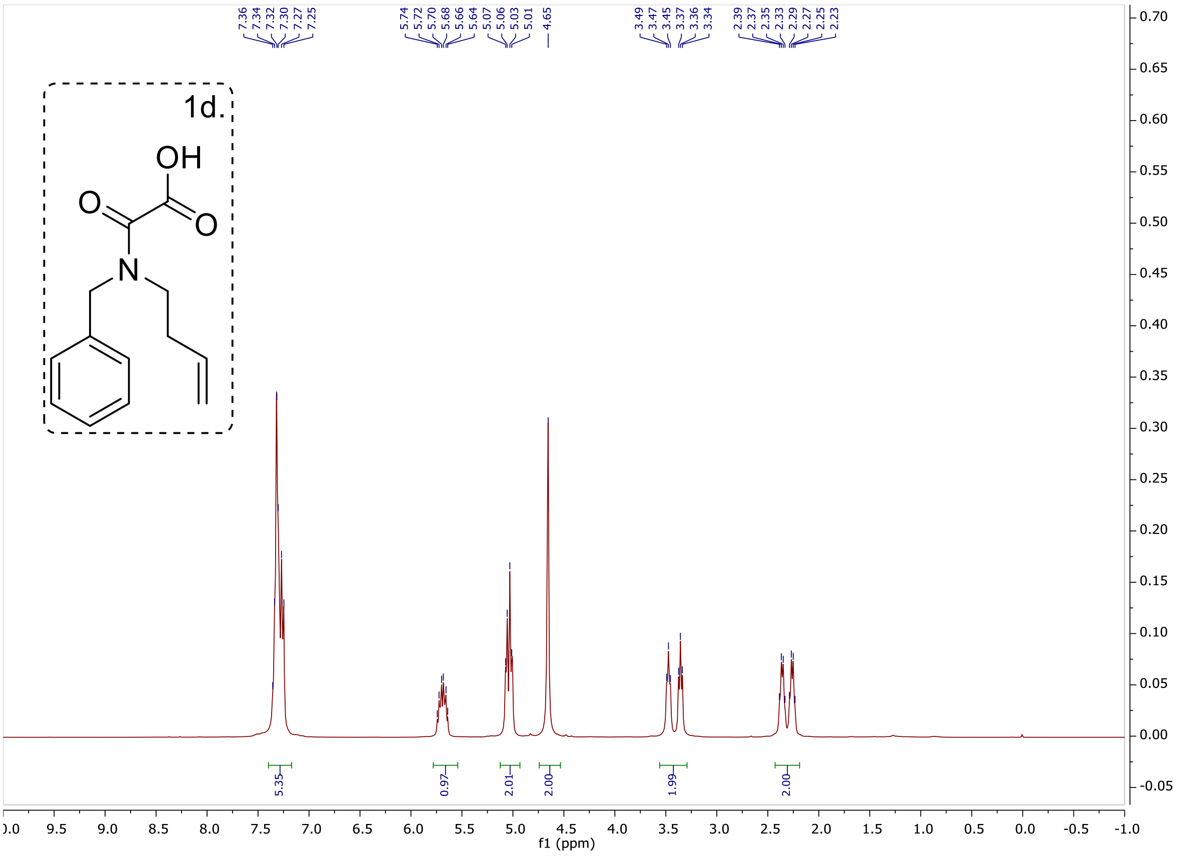

^{1}H NMR of 1d in $CDCl_3$ (400 MHz)

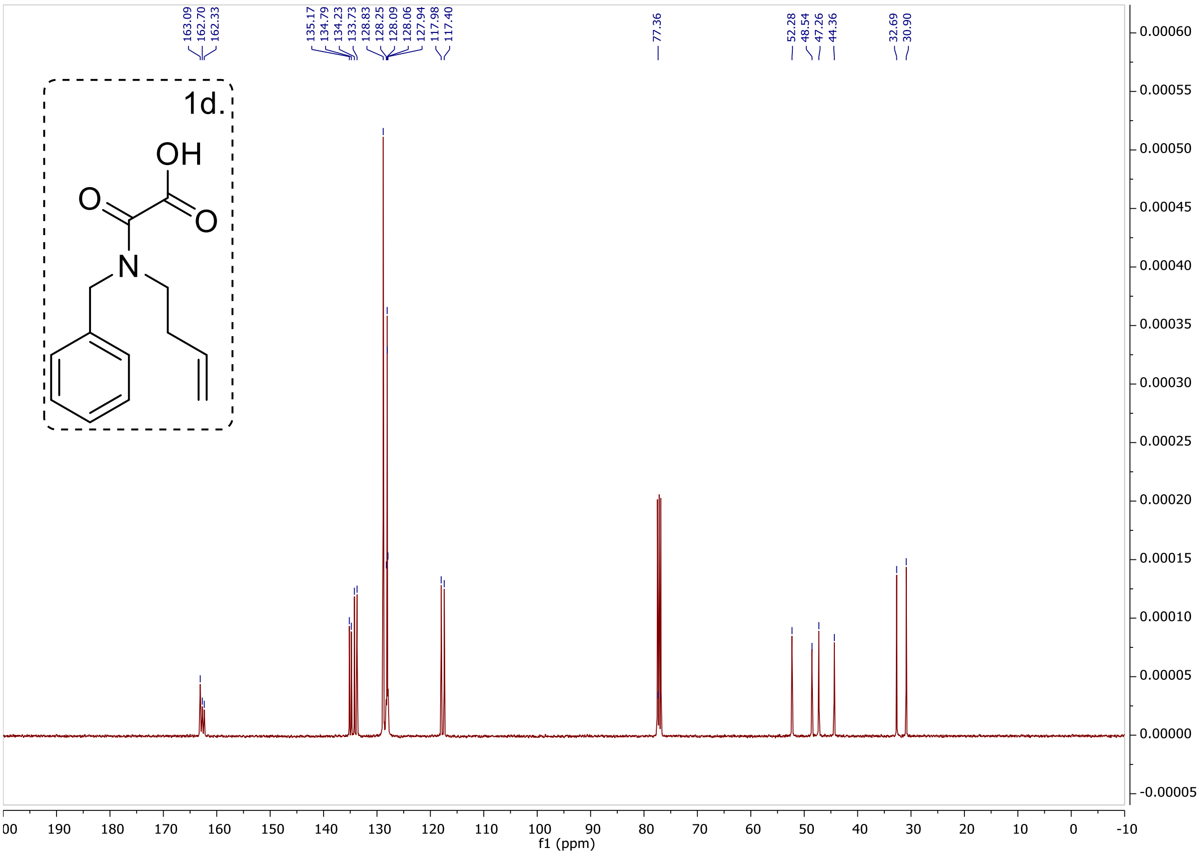

^{13}C NMR of 1d in $CDCl_3$ (101 MHz)

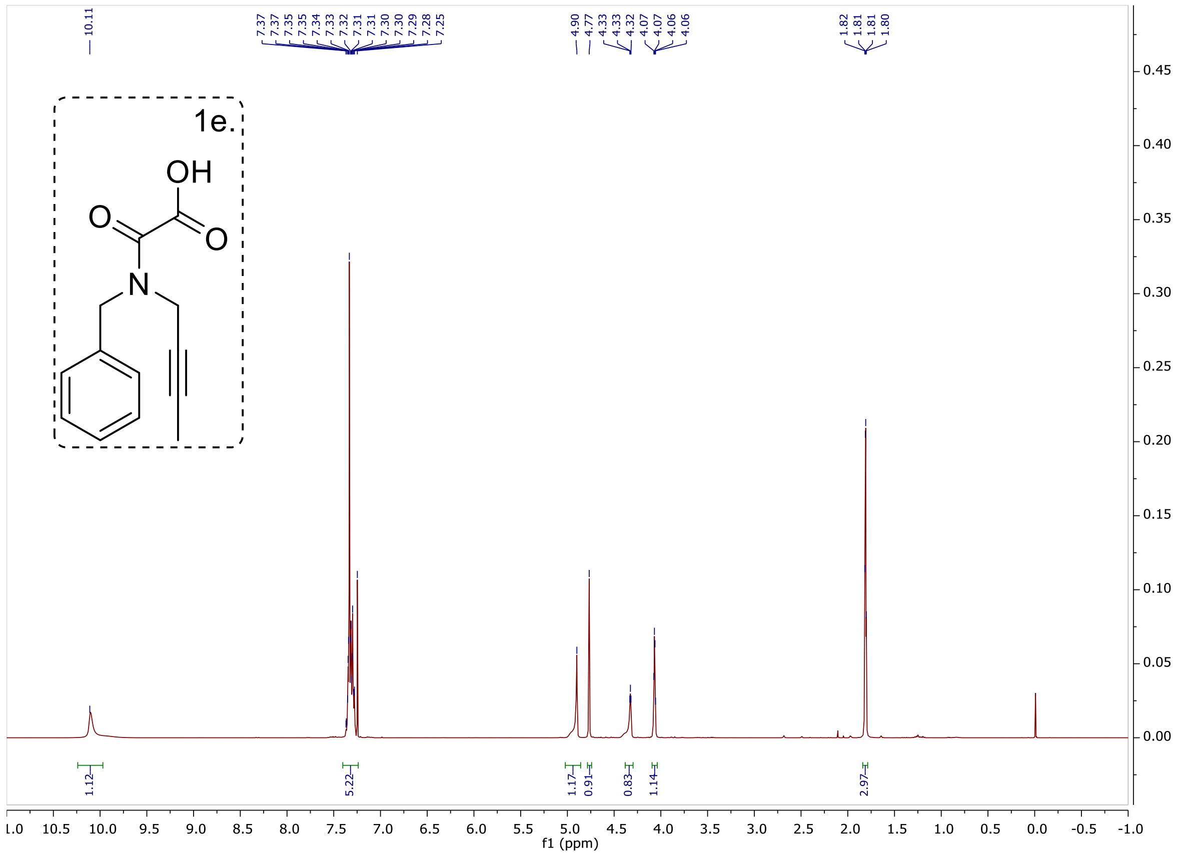

^{1}H NMR of 1e in $CDCl_3$ (400 MHz)

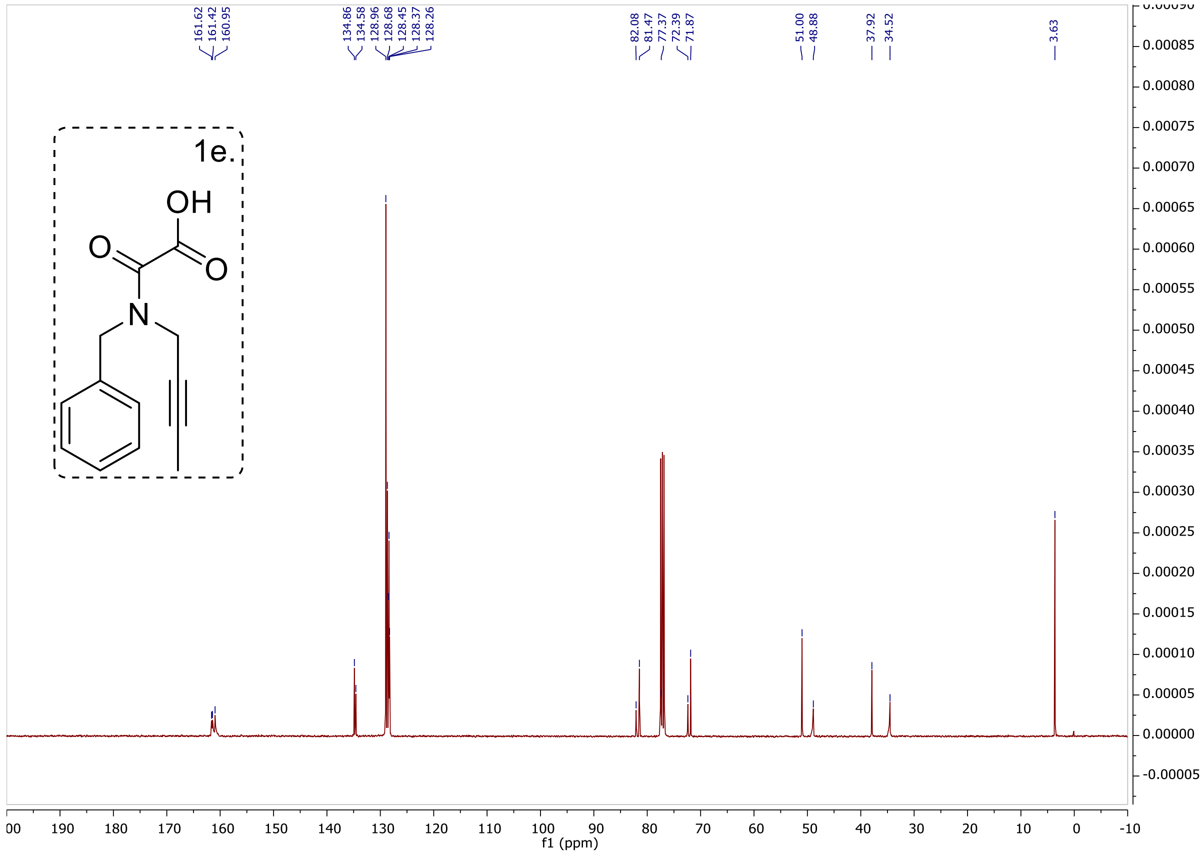

^{13}C NMR of 1e in $CDCl_3$ (101 MHz)

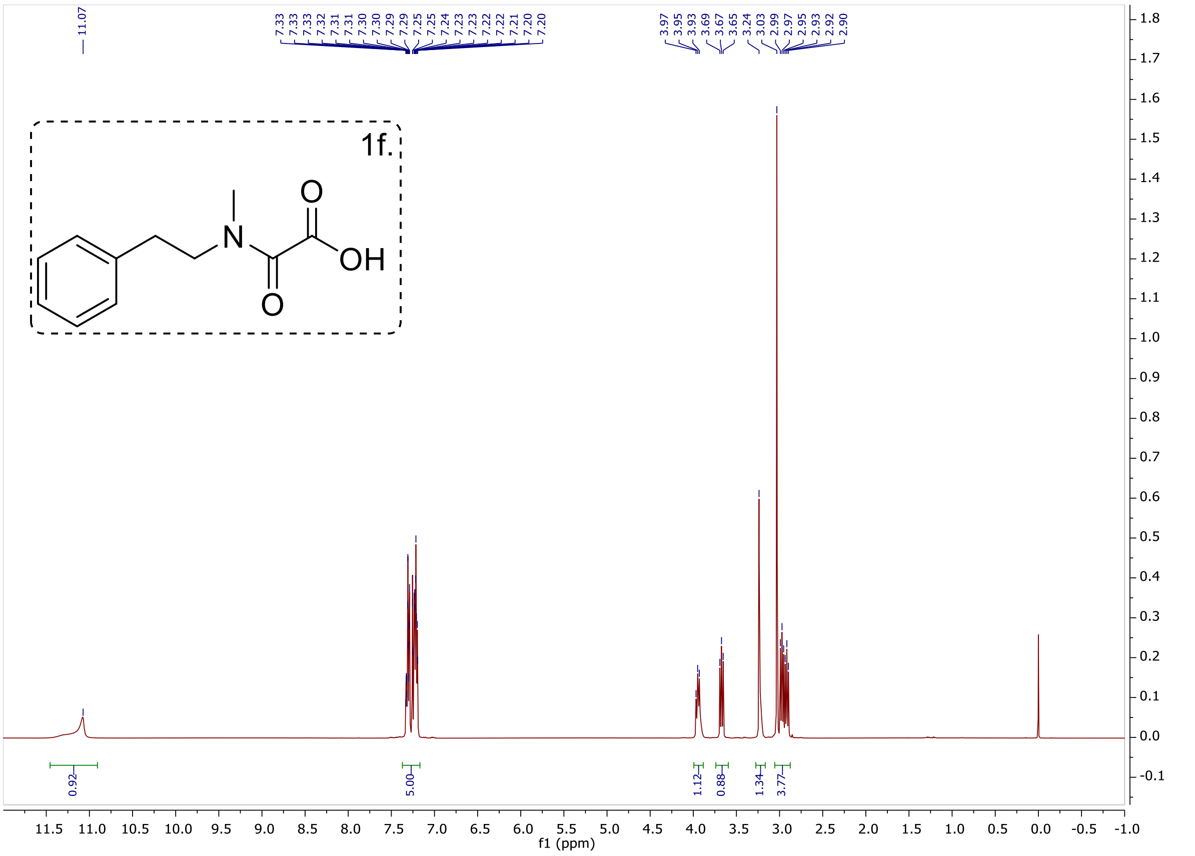

^{1}H NMR of 1f in $CDCl_3$ (400 MHz)

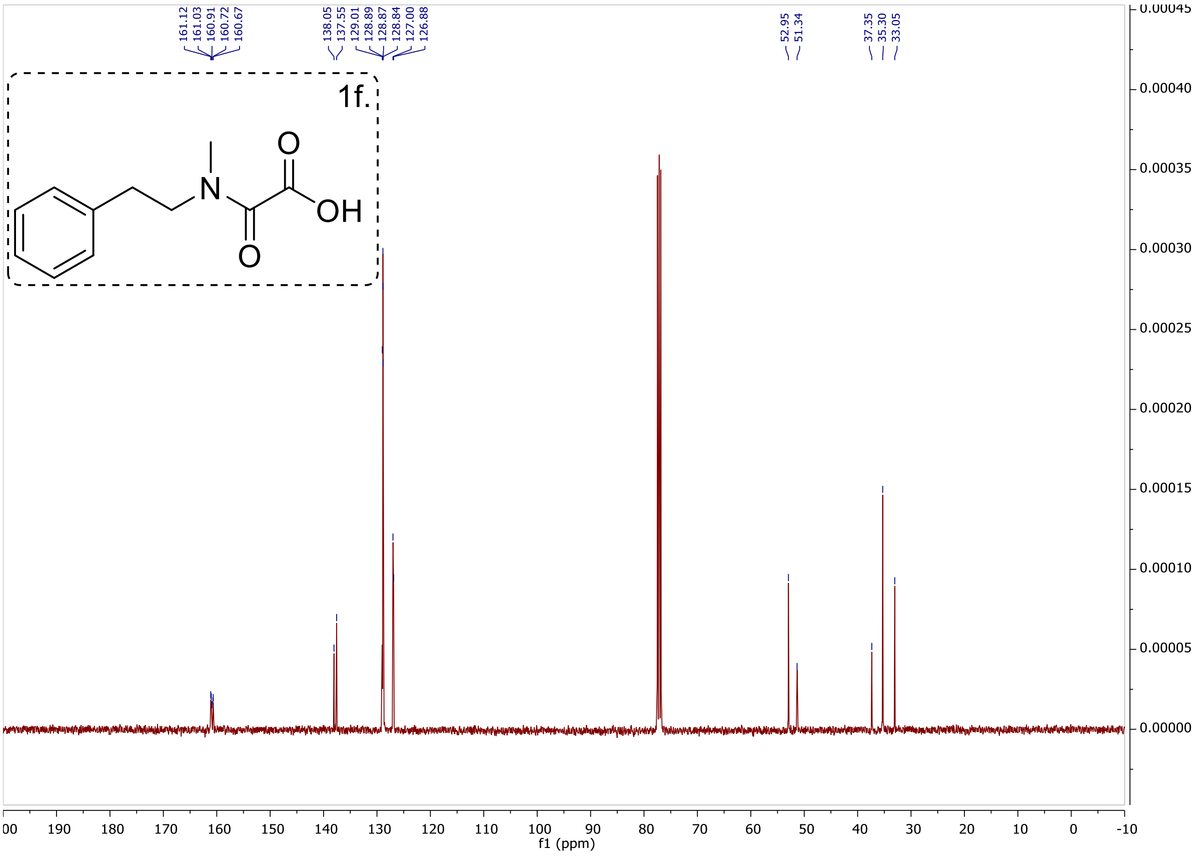

^{13}C NMR of 1f in $CDCl_3$ (101 MHz)

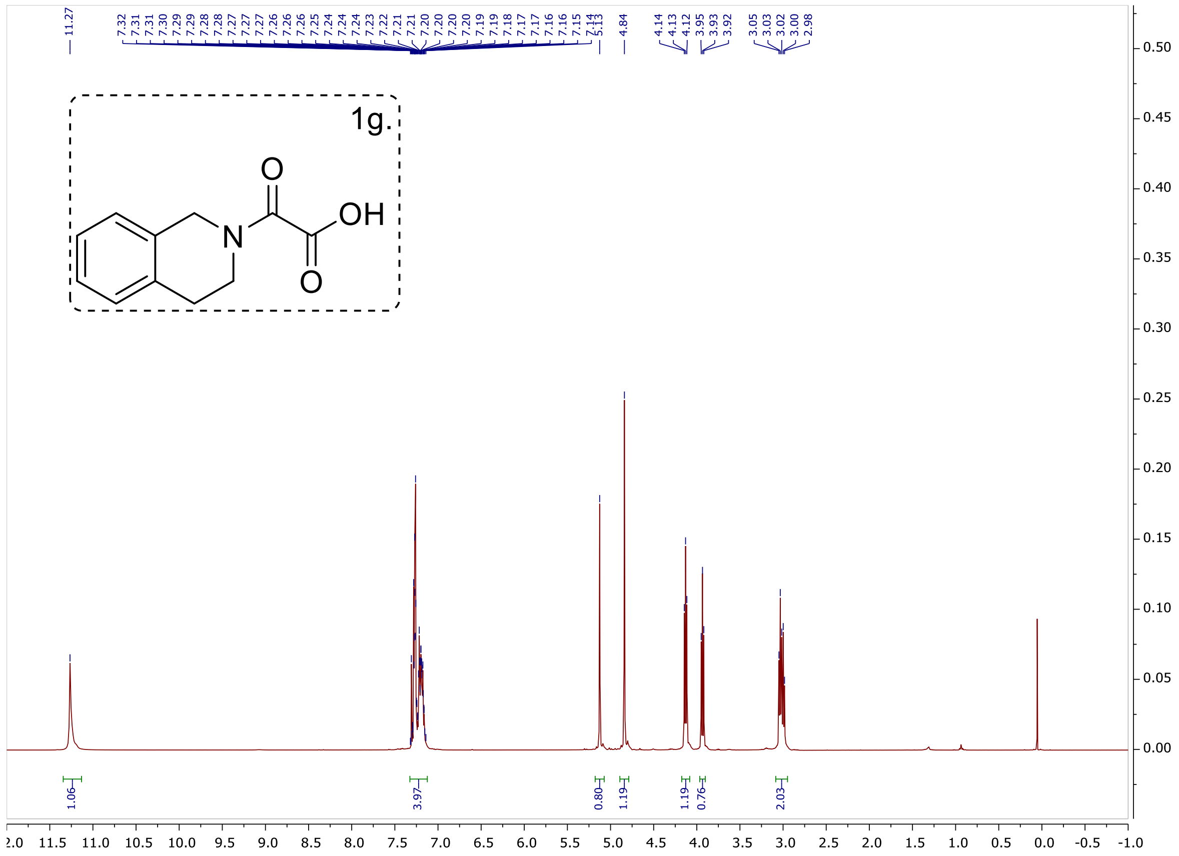

^{1}H NMR of 1g in $CDCl_3$ (400 MHz)

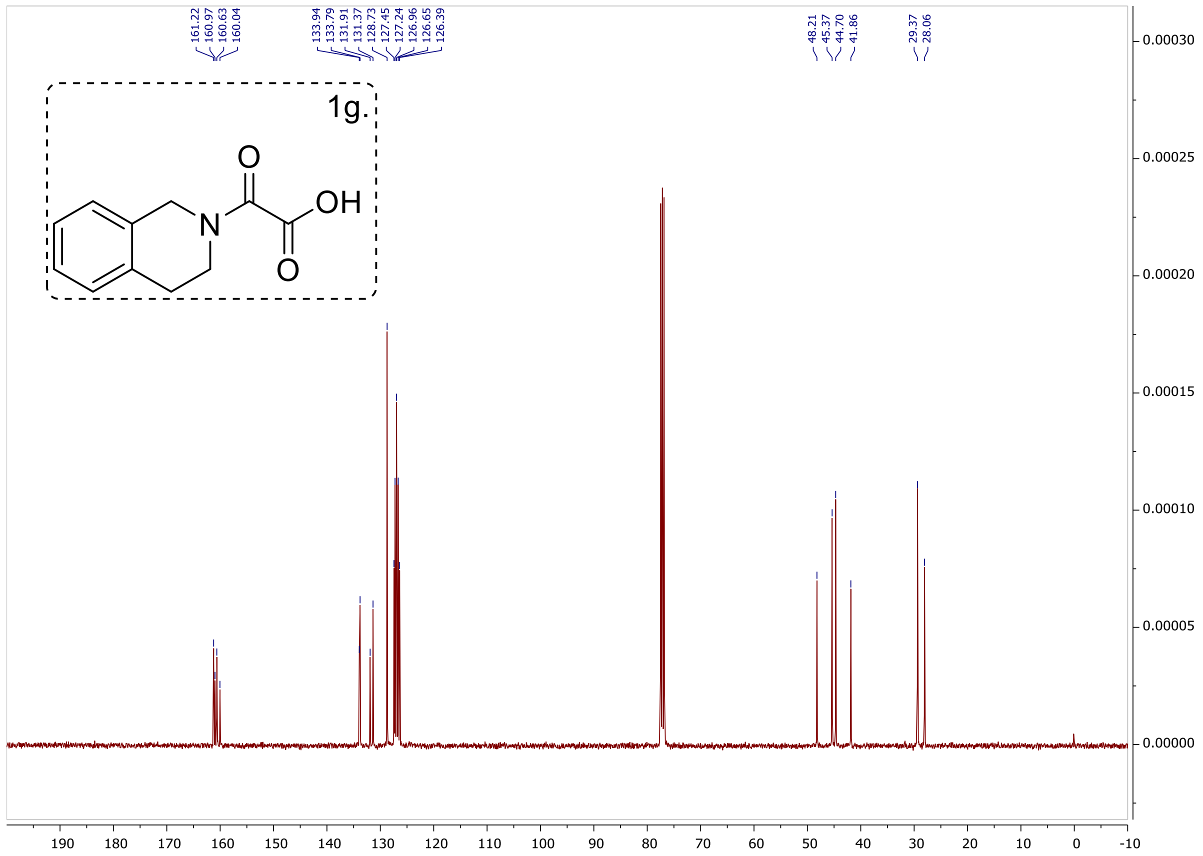

^{13}C NMR of 1g in $CDCl_3$ (101 MHz)

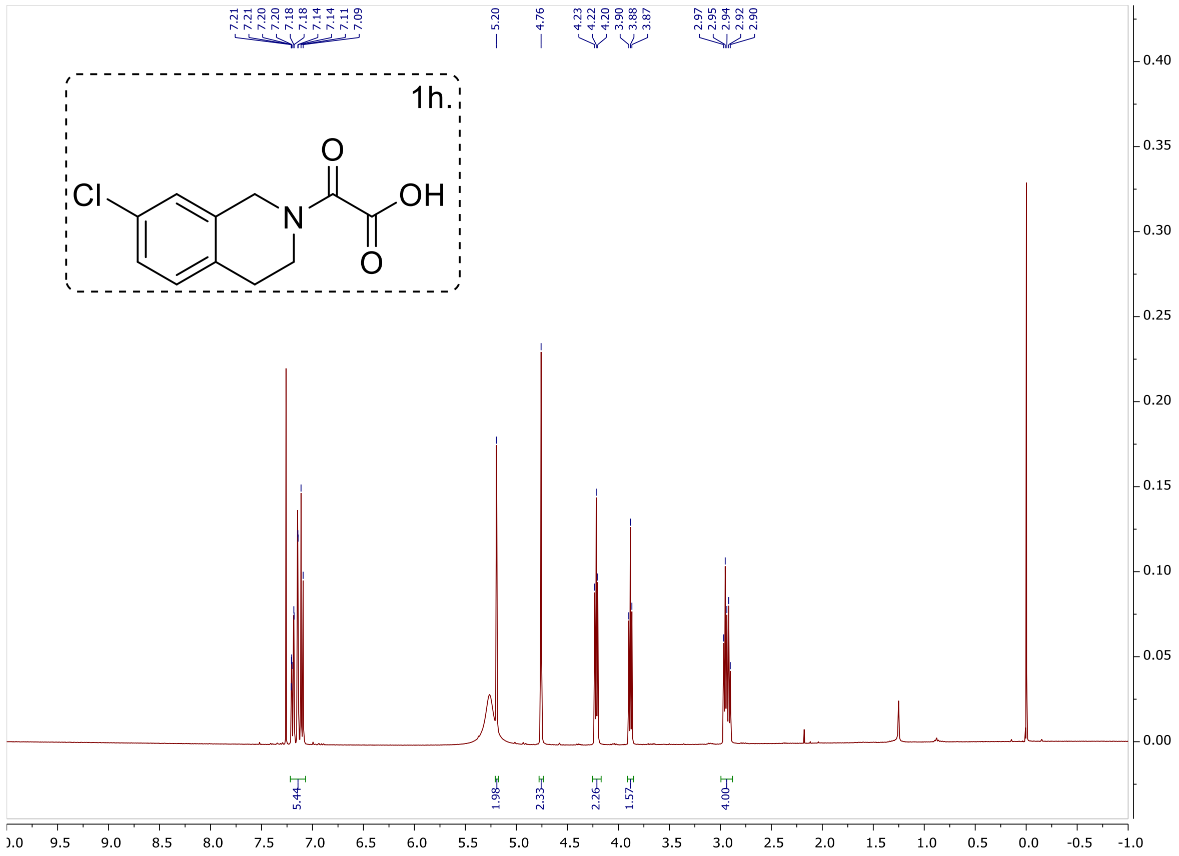

^{1}H NMR of 1h in $CDCl_3$ (400 MHz)

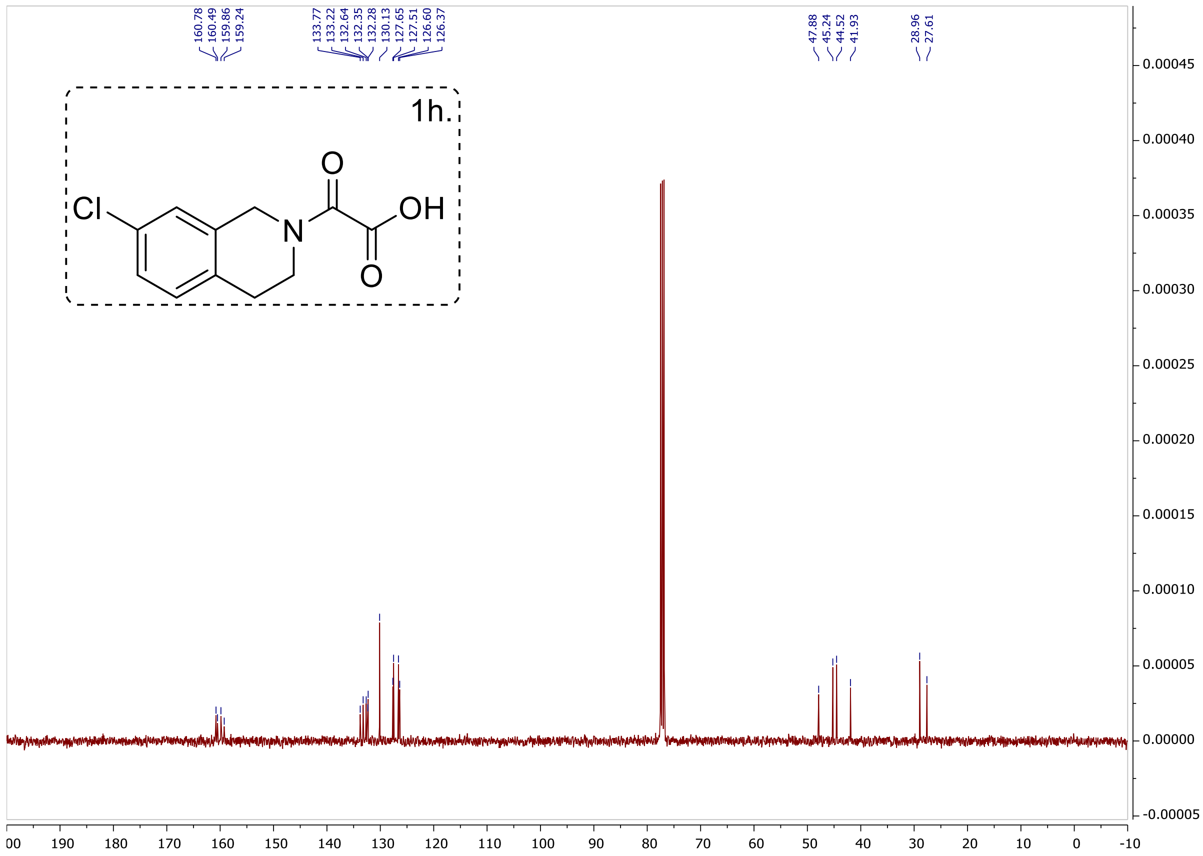

^{13}C NMR of 1h in $CDCl_3$ (101 MHz)

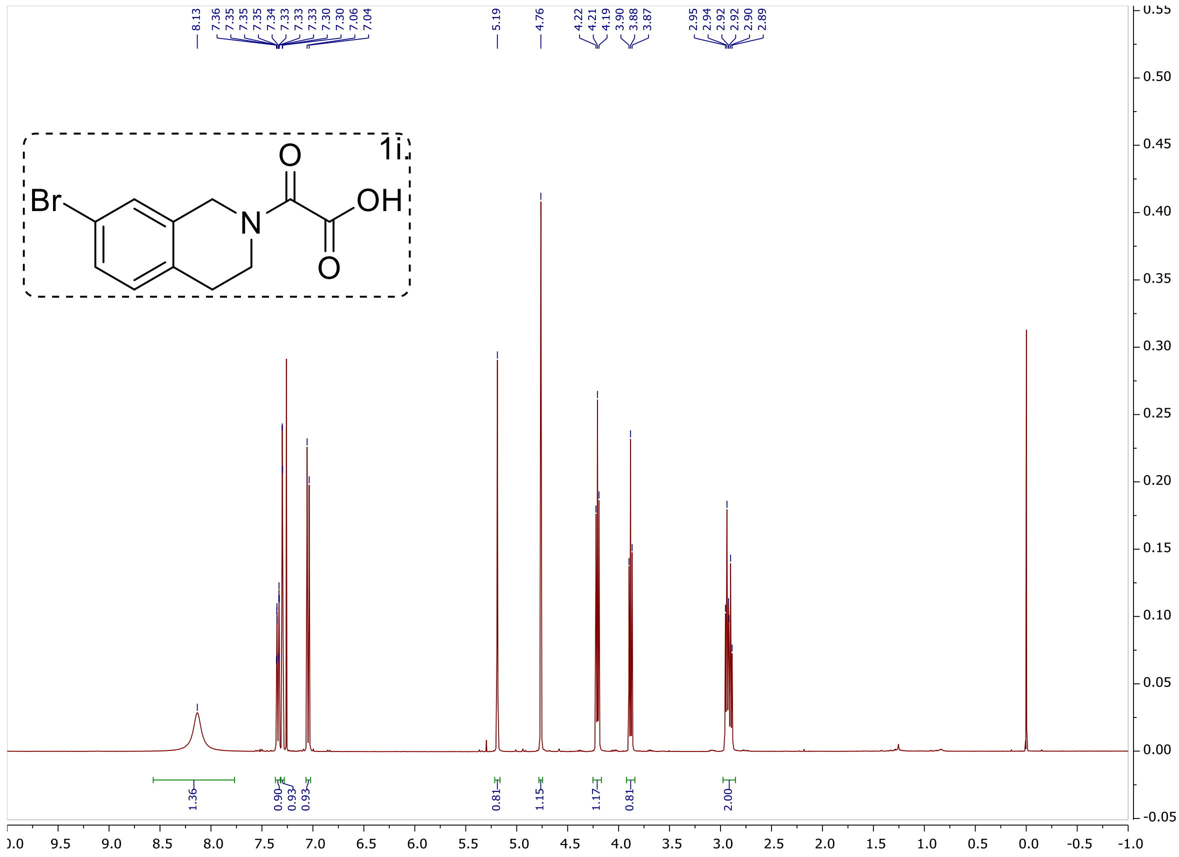

^{1}H NMR of 1i in $CDCl_3$ (400 MHz)

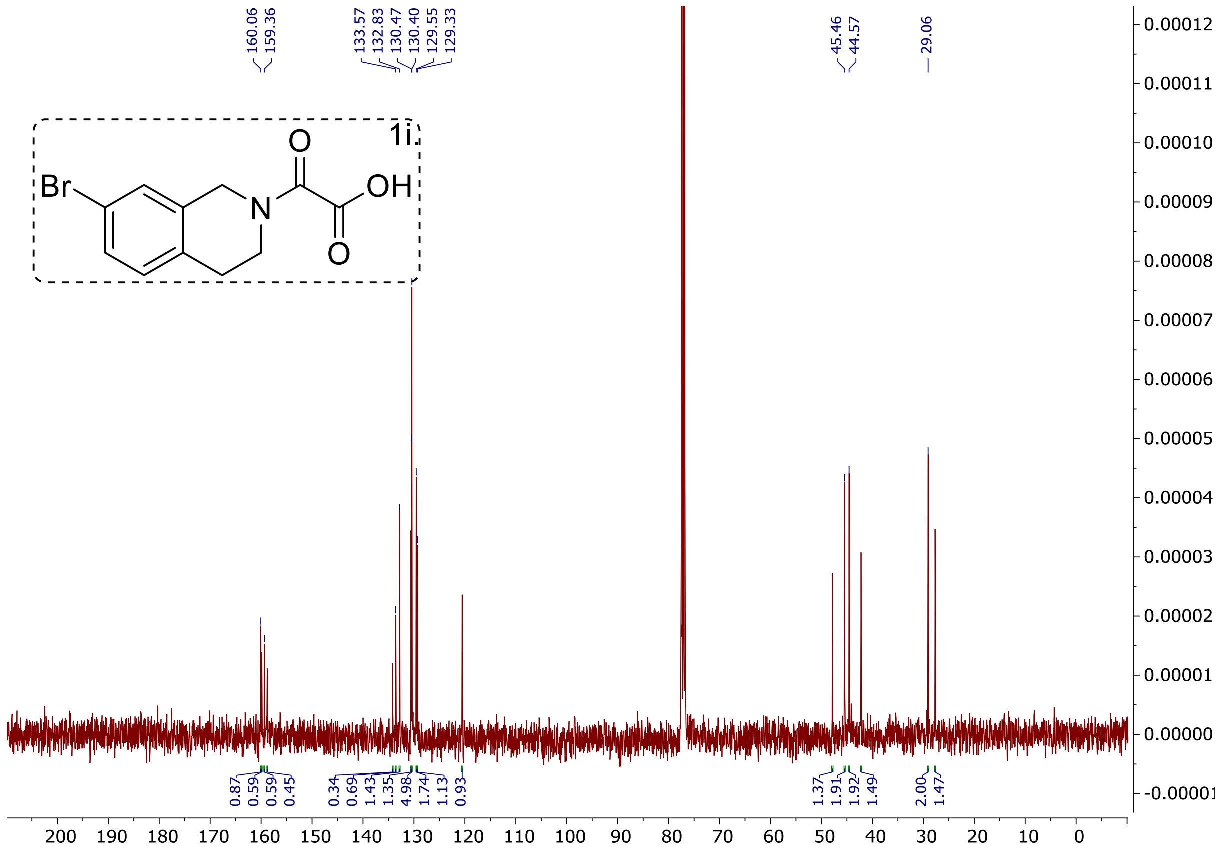

^{13}C NMR of 1i in $CDCl_3$ (101 MHz)

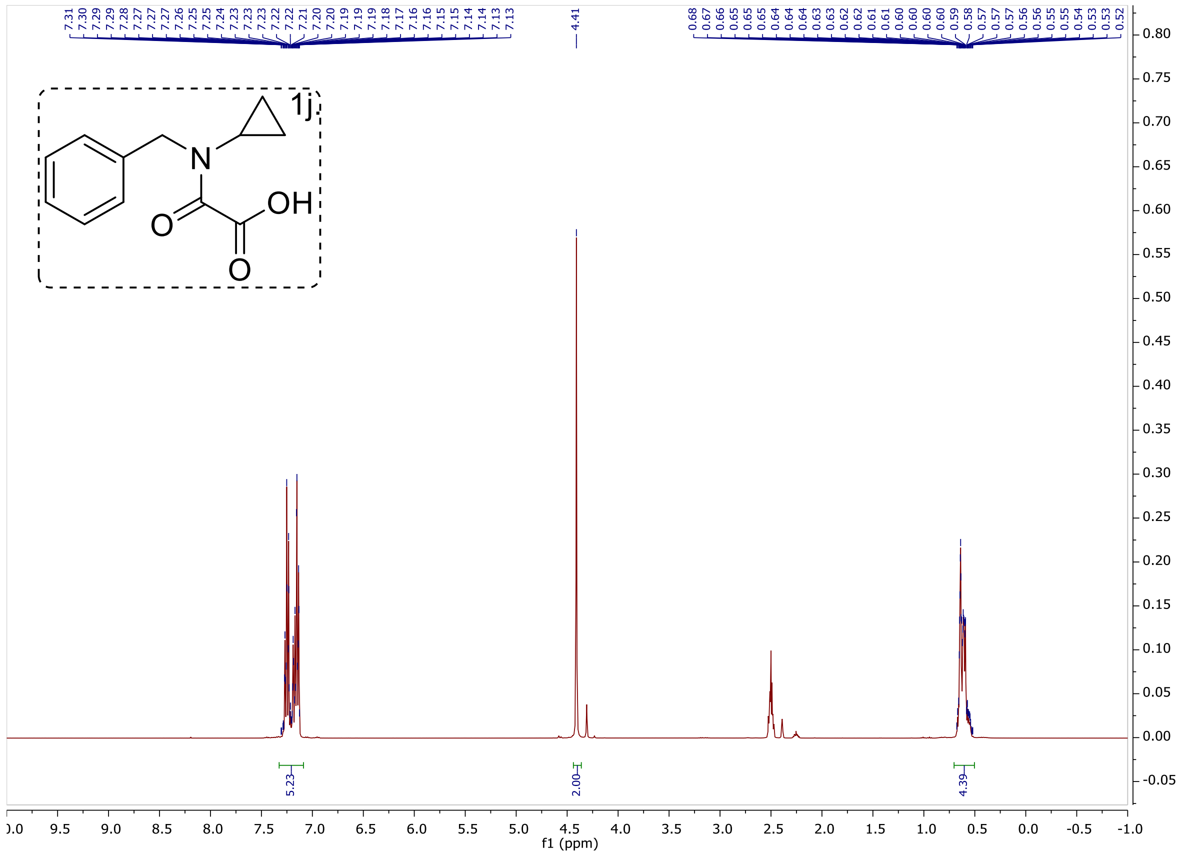

^{1}H NMR of 1j in DMSO (400 MHz)

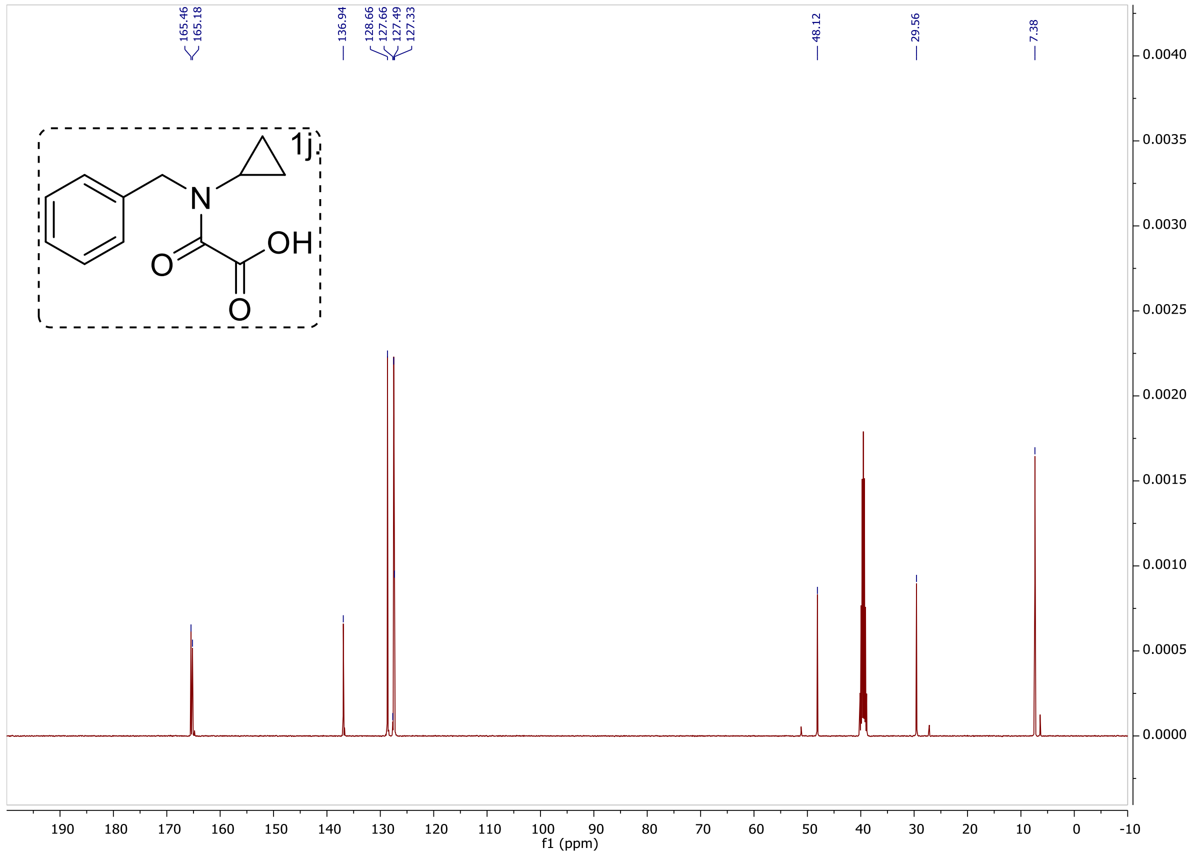

^{13}C NMR of 1j in DMSO (101 MHz)

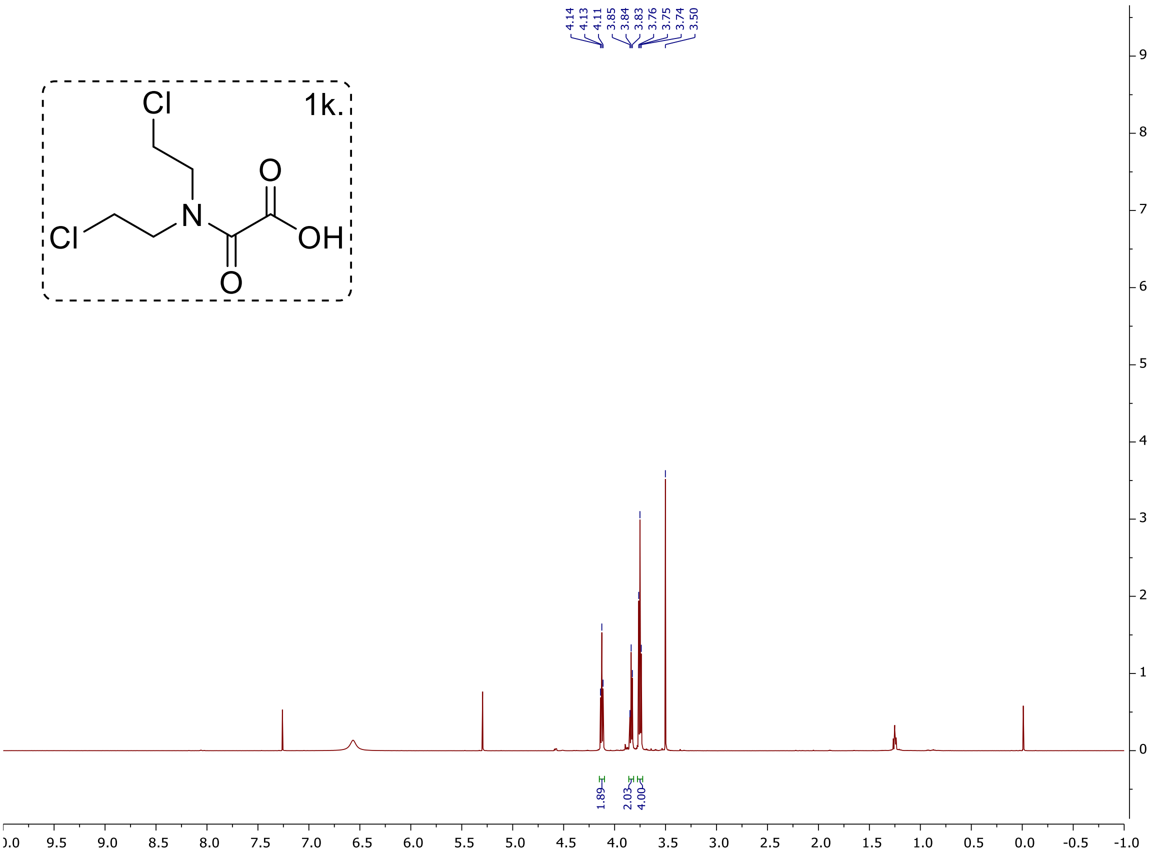

^{1}H NMR of 1k in $CDCl_3$ (500 MHz)

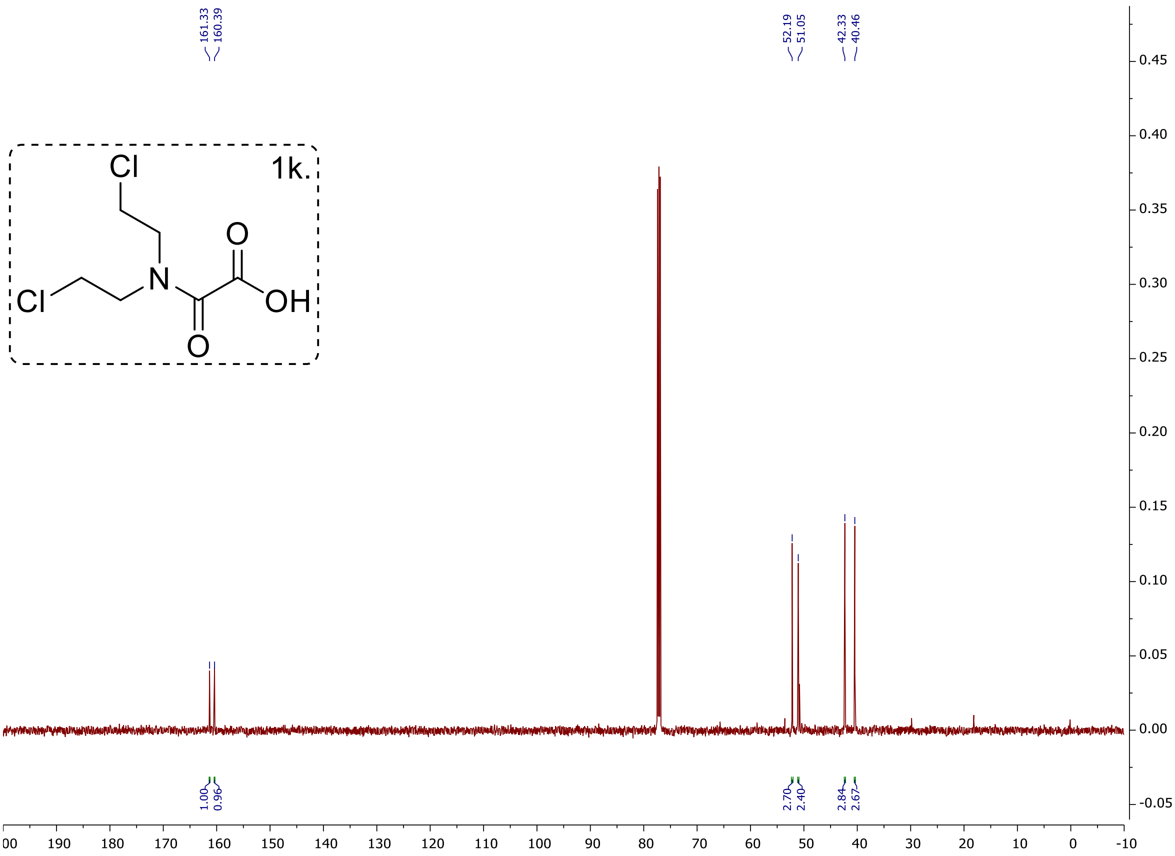

^{13}C NMR of 1k in $CDCl_3$ (126 MHz)

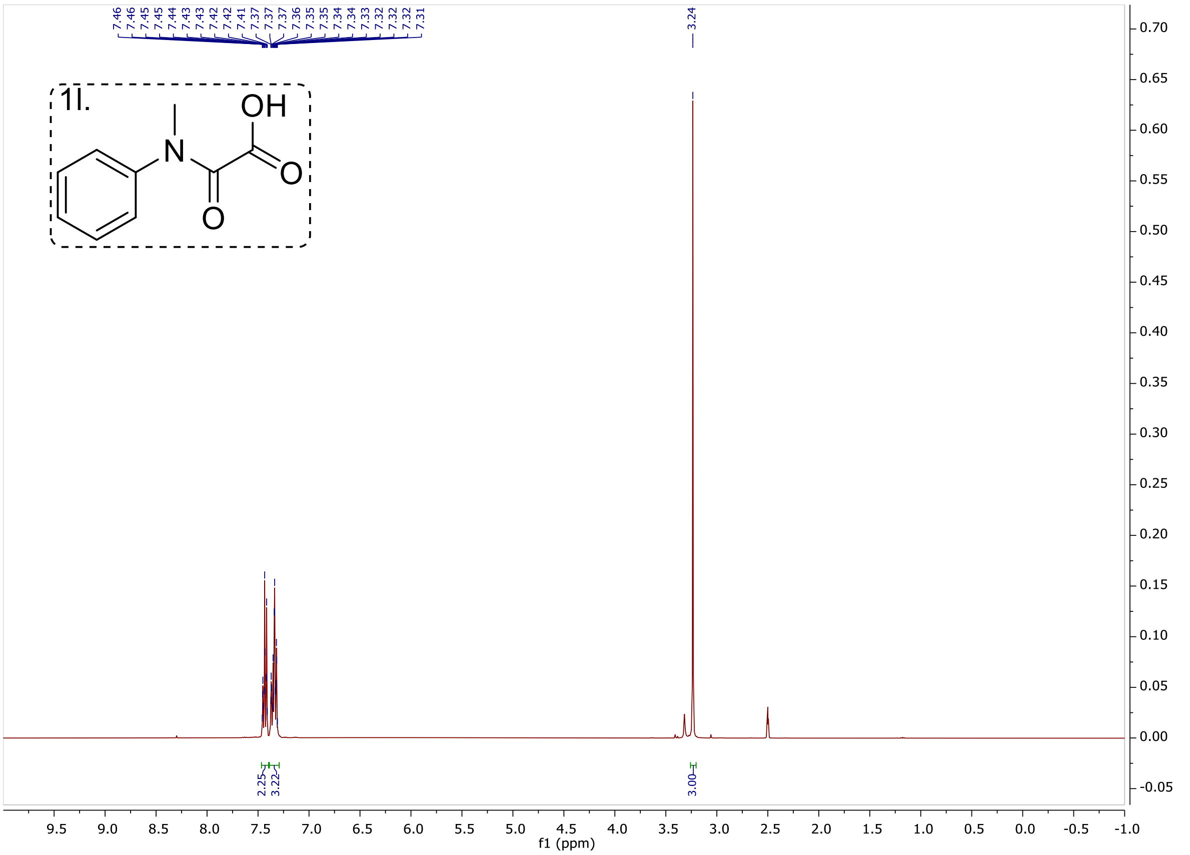

^{1}H NMR of 1l in DMSO-d_6 (400 MHz)

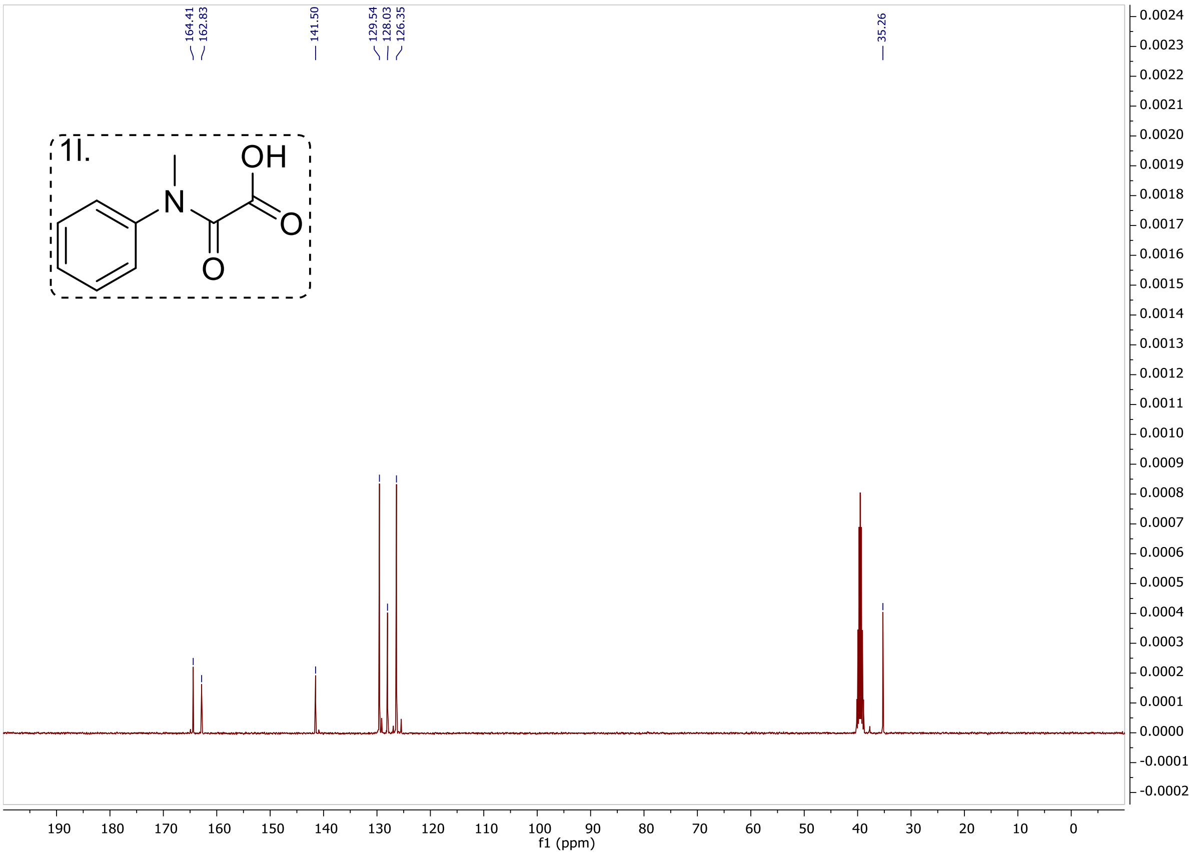

^{13}C NMR of 1l in DMSO-d_6 (101 MHz)

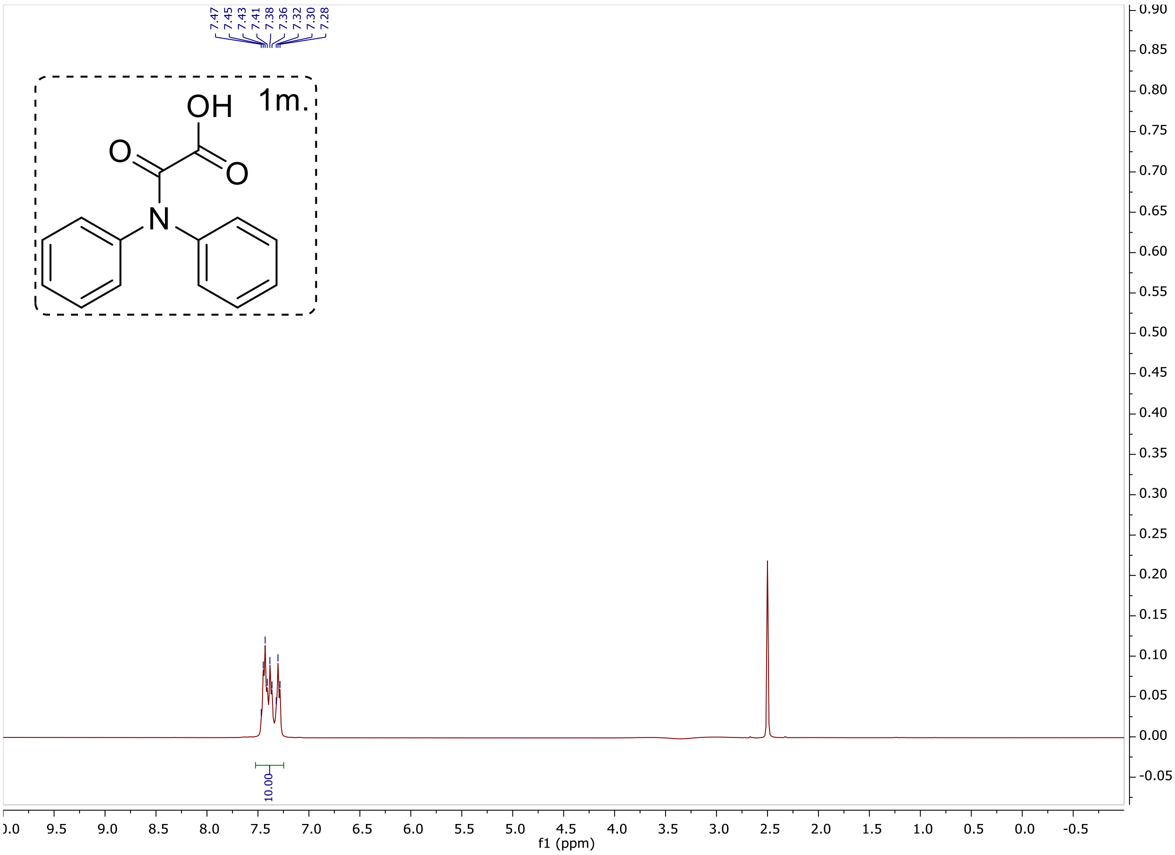

^{1}H NMR of 1m in DMSO-d_6 (400 MHz)

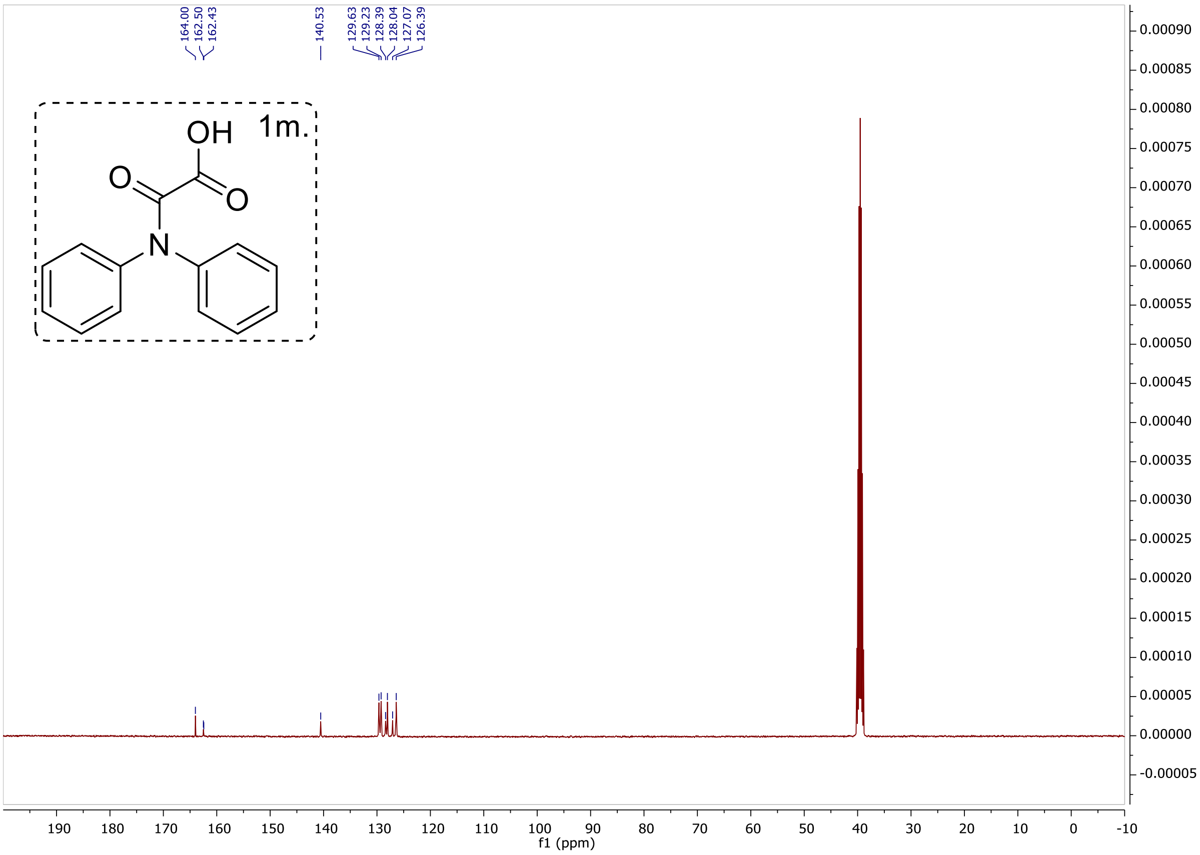

^{13}C NMR of 1m in DMSO-d_6 (101 MHz)

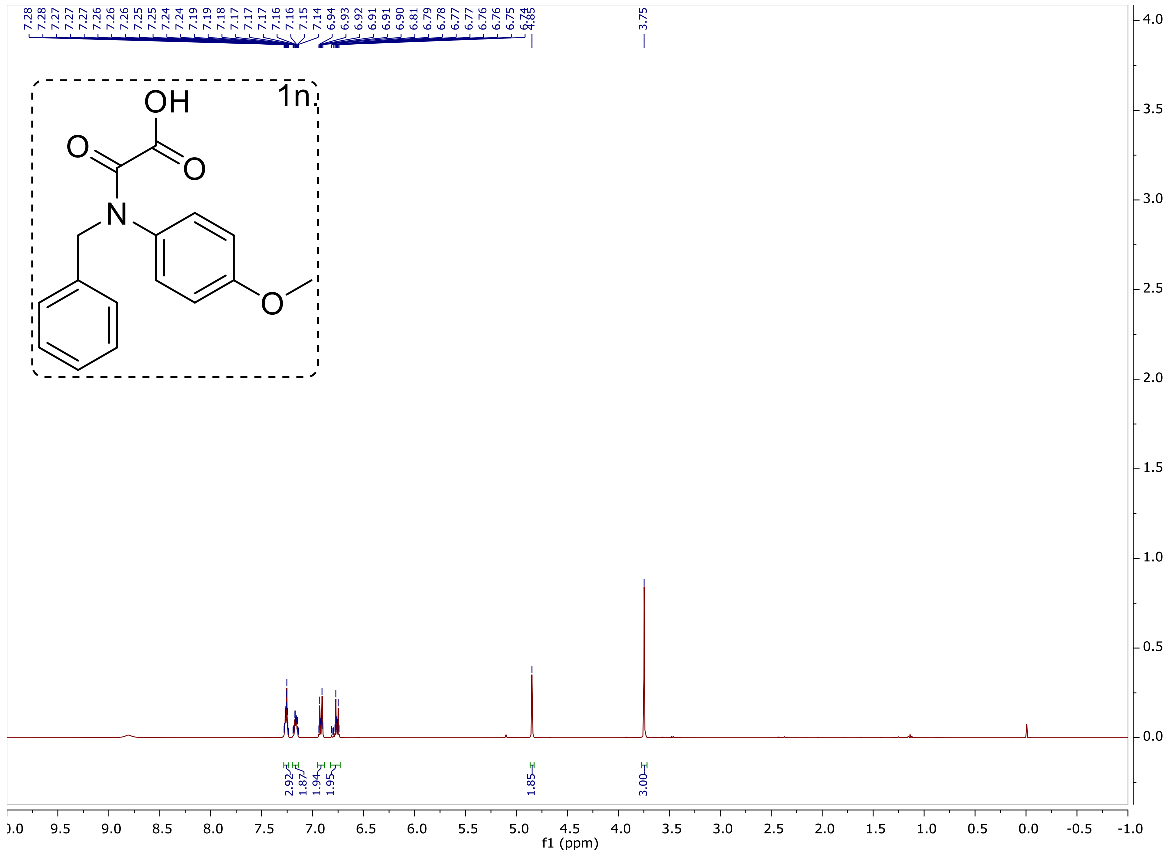

^{1}H NMR of 1n in $CDCl_3$ (400 MHz)

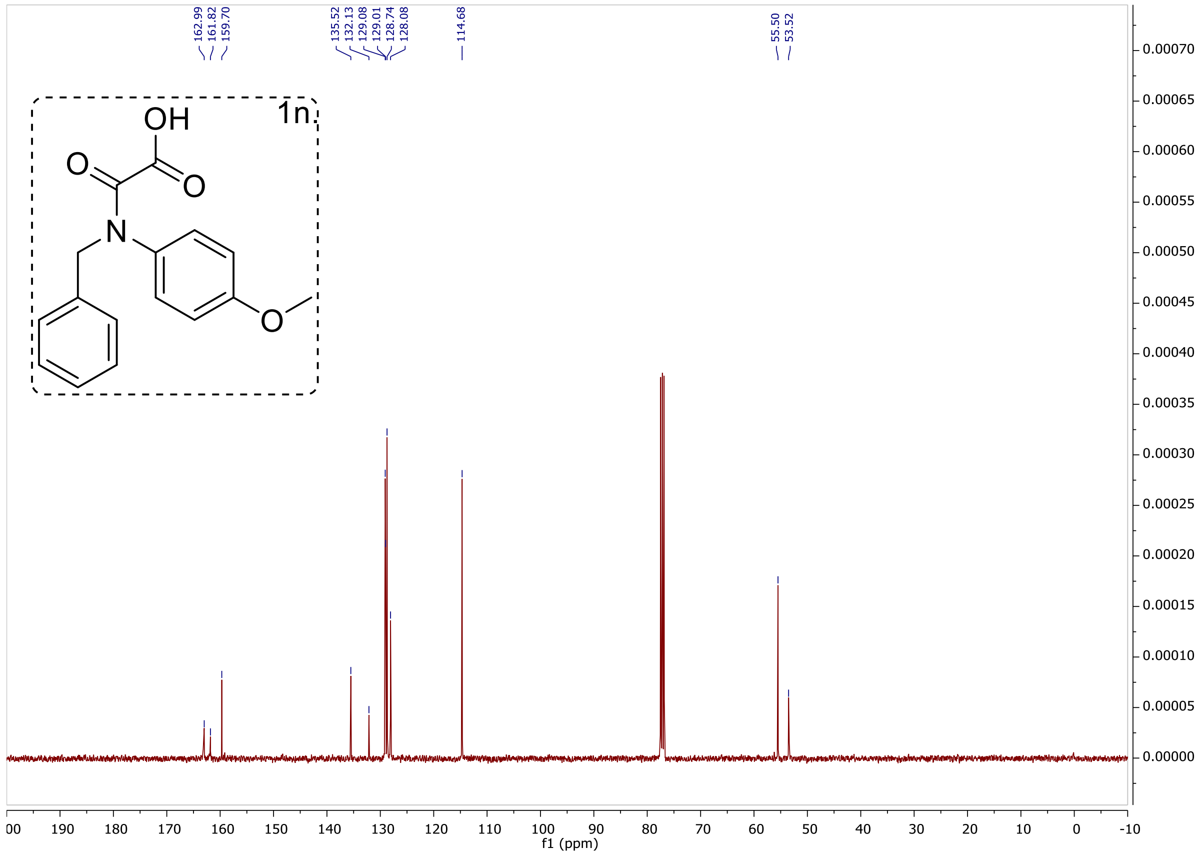

^{13}C NMR of 1n in $CDCl_3$ (101 MHz)

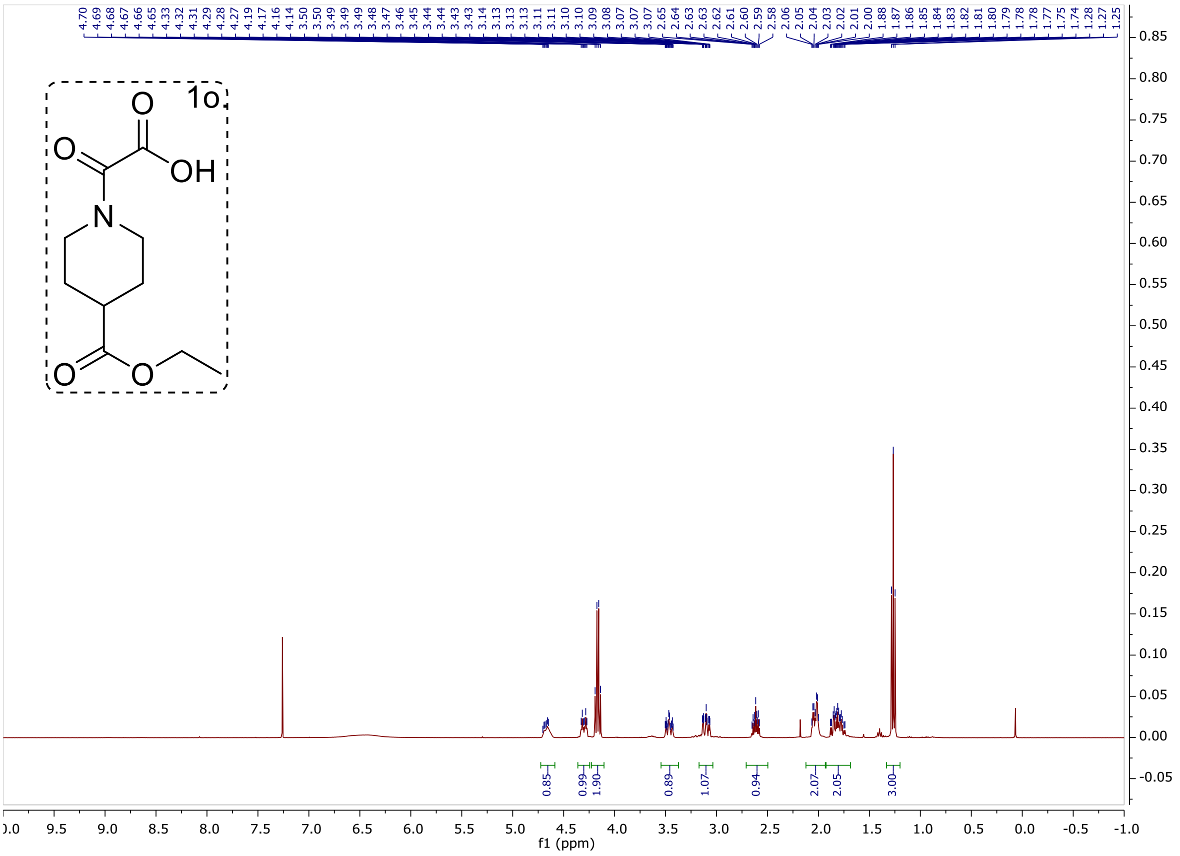

^{1}H NMR of 1o in $CDCl_3$ (400 MHz)

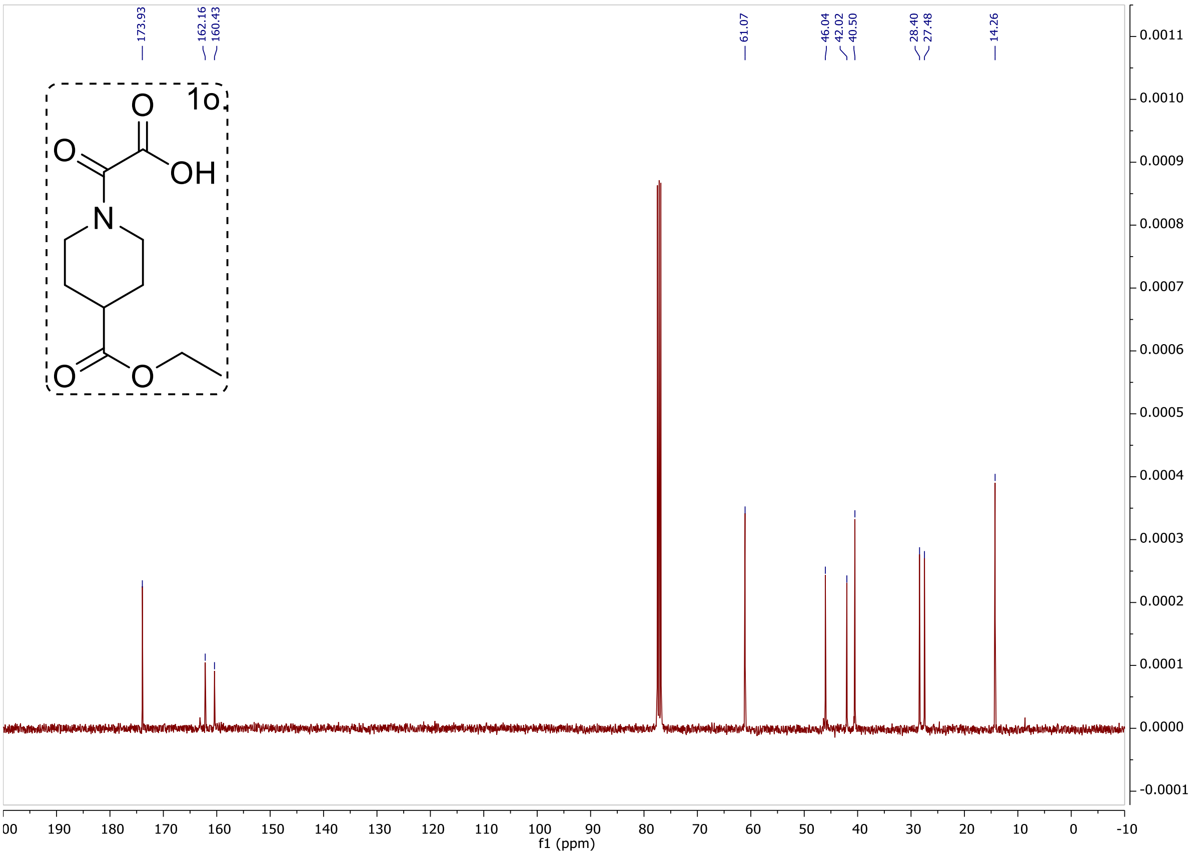

^{13}C NMR of 1o in $CDCl_3$ (101 MHz)

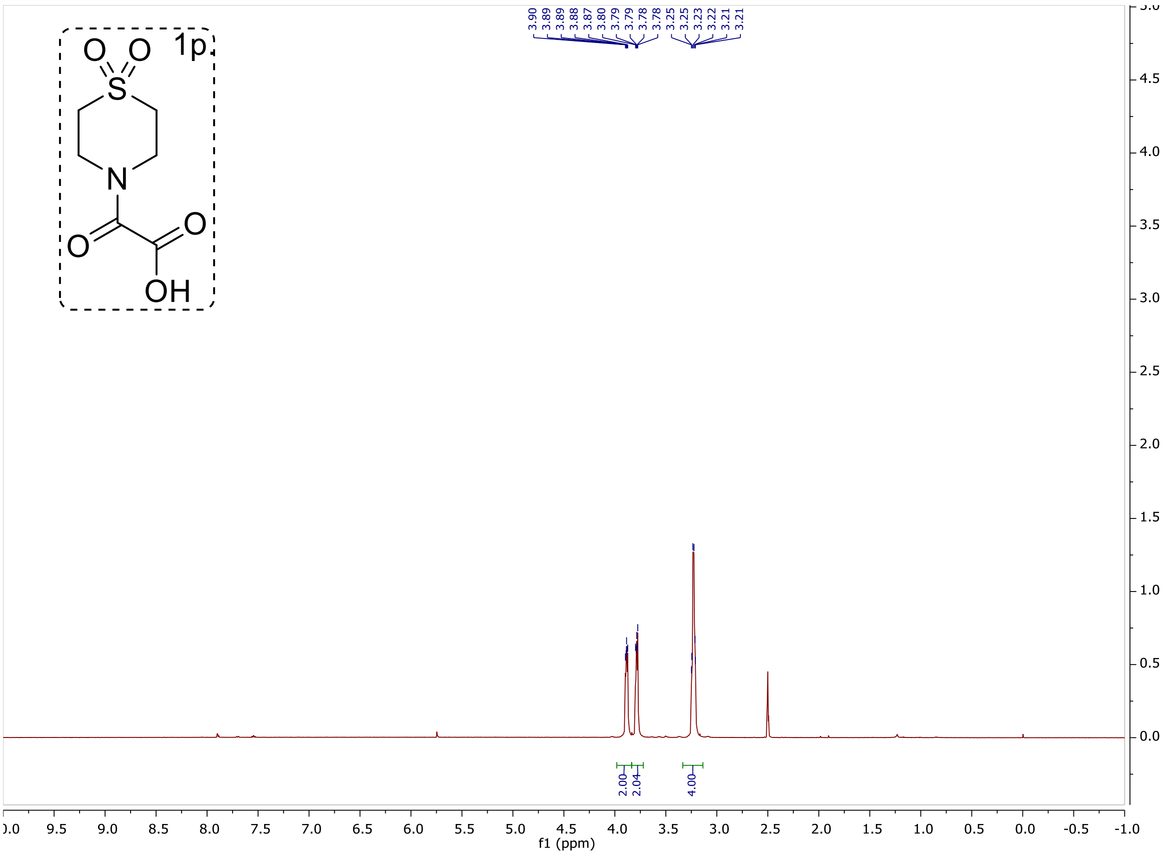

^{1}H NMR of 1p in DMSO-d_6 (500 MHz)

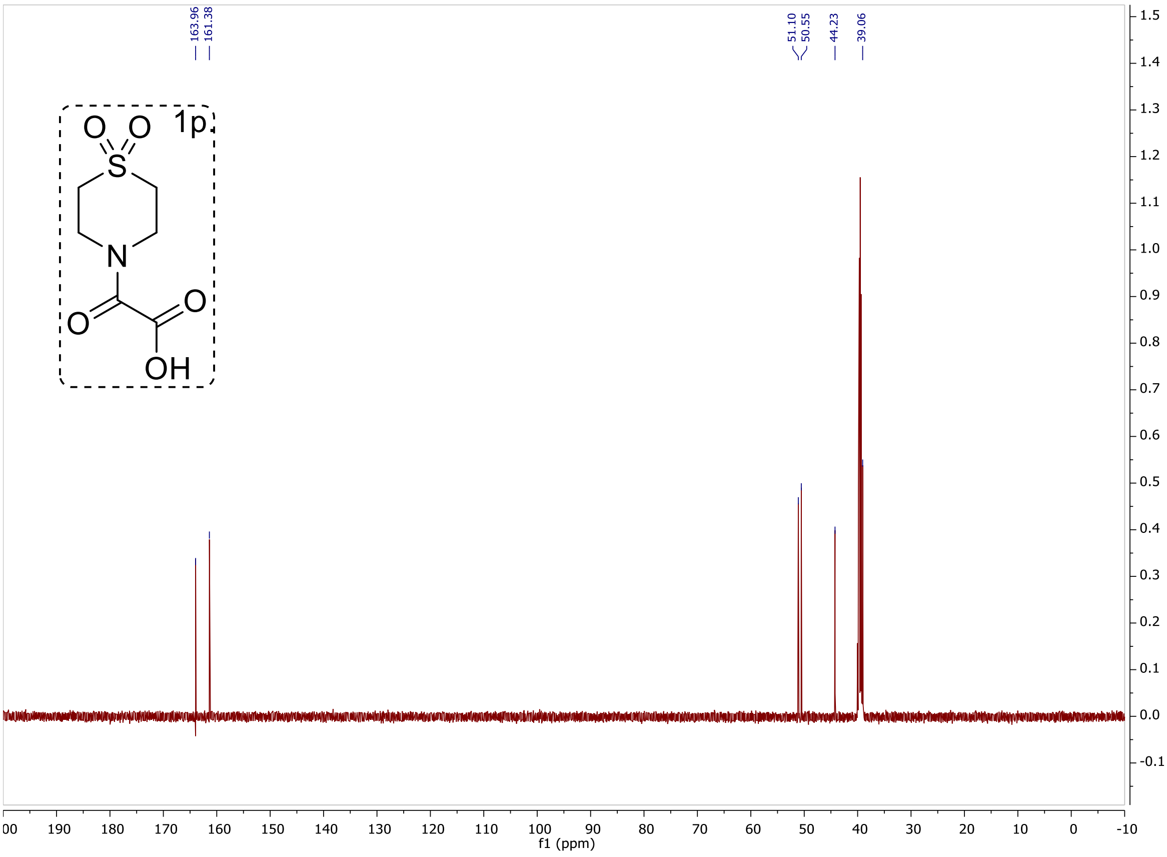

^{13}C NMR of 1p in DMSO-d_6 (126 MHz)

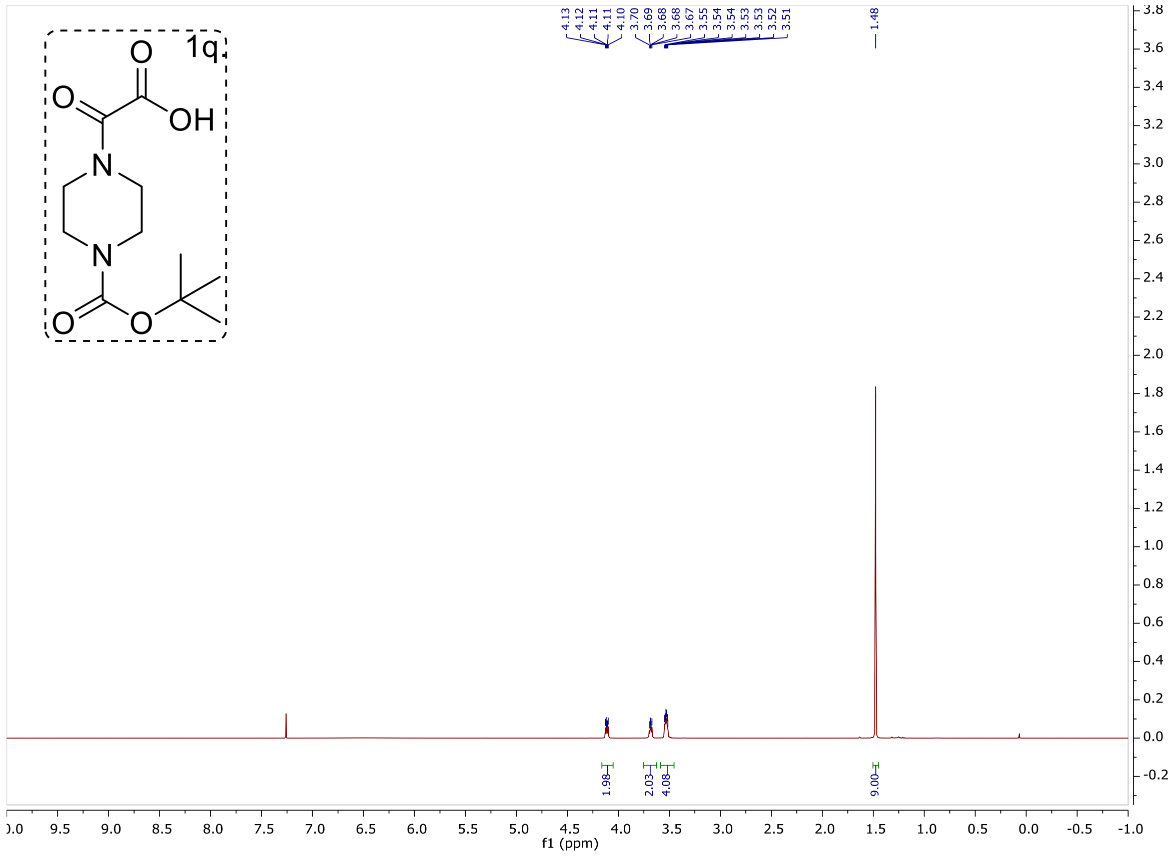

^{1}H NMR of 1q in $CDCl_3$ (400 MHz)

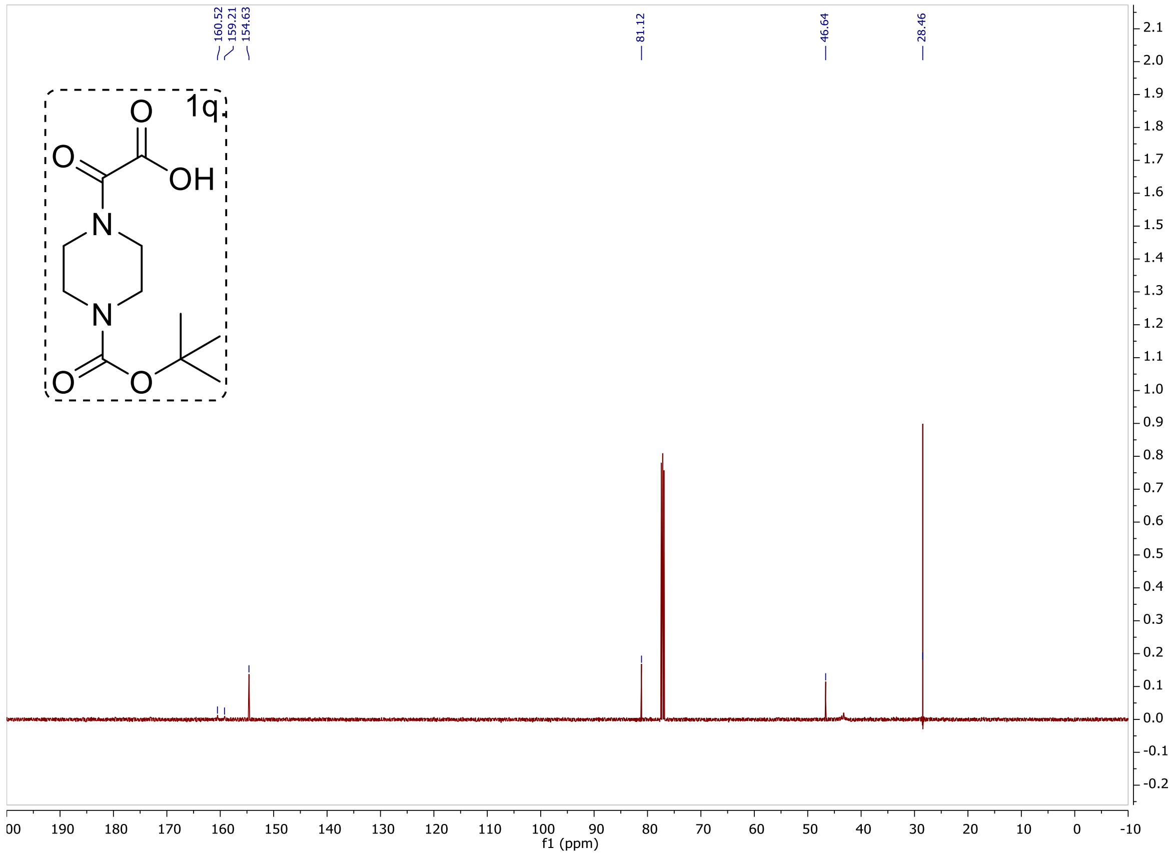

^{13}C NMR of 1q in $CDCl_3$ (126 MHz)

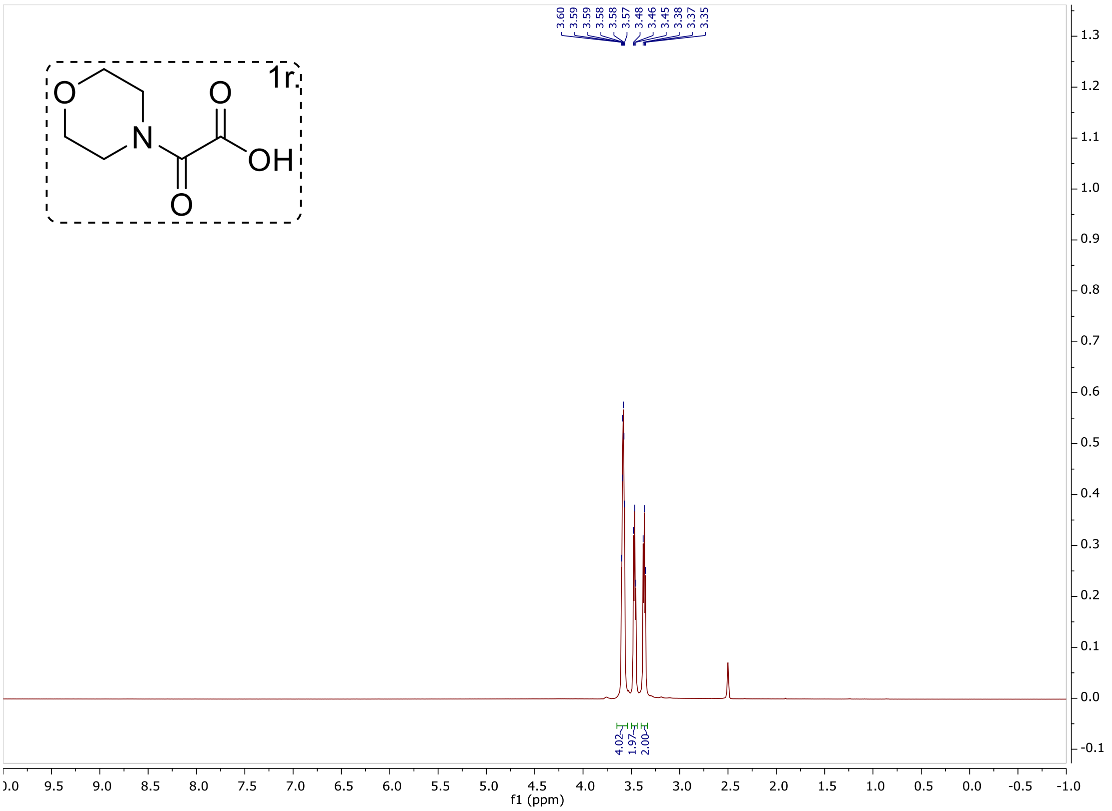

^{1}H NMR of 1r in $CDCl_3$ (400 MHz)

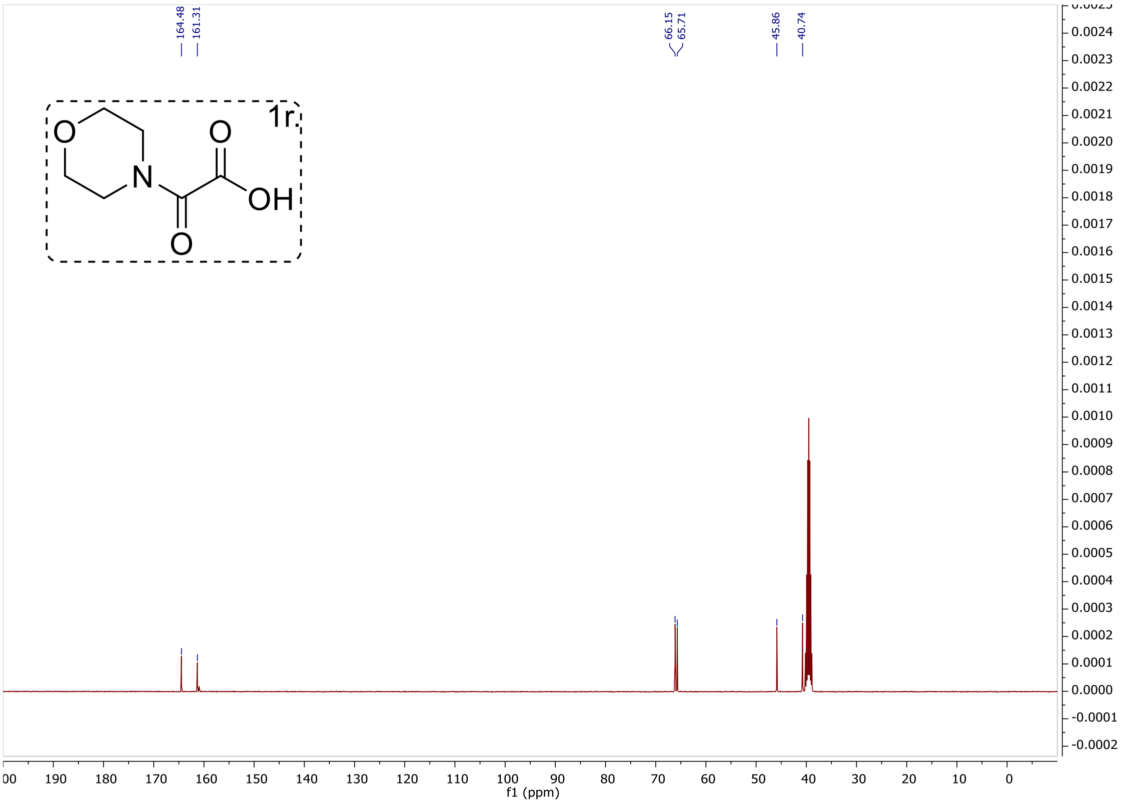

^{13}C NMR of 1r in $CDCl_3$ (101 MHz)

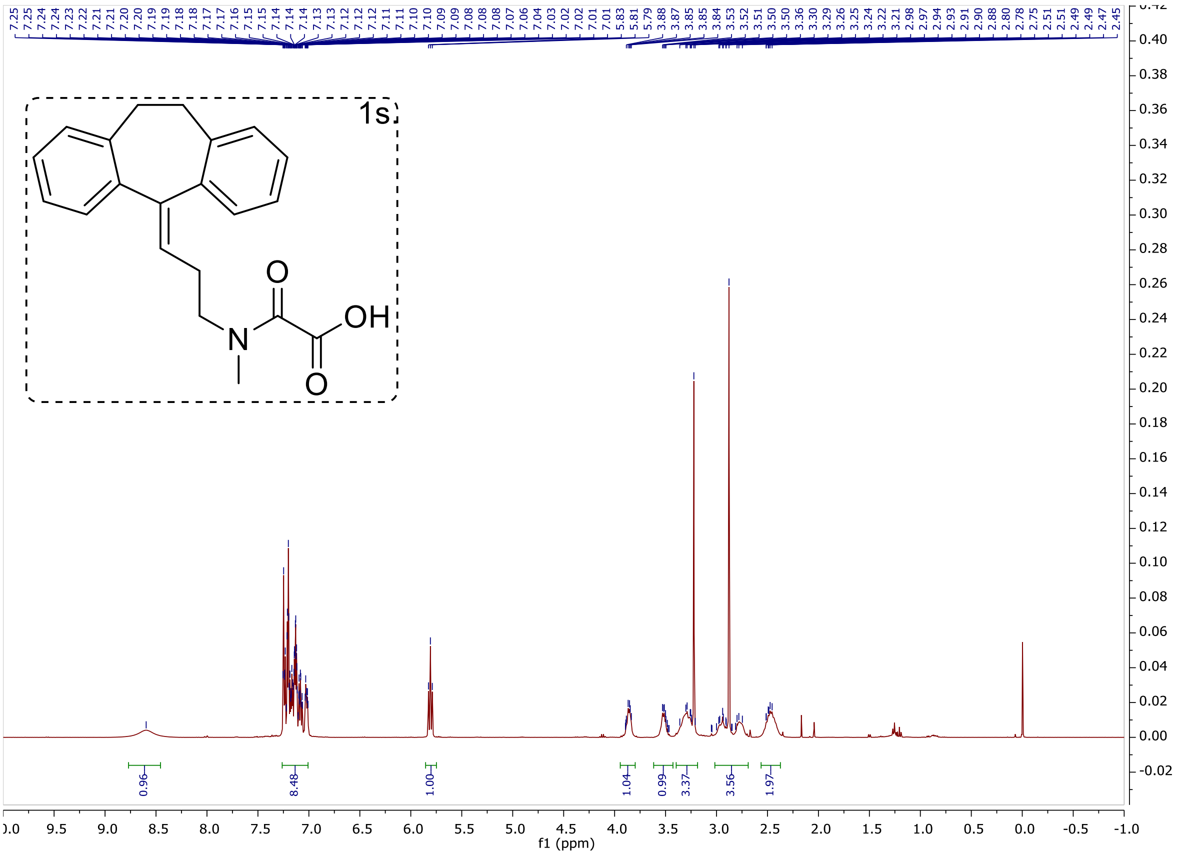

^{1}H NMR of 1s in $CDCl_3$ (400 MHz)

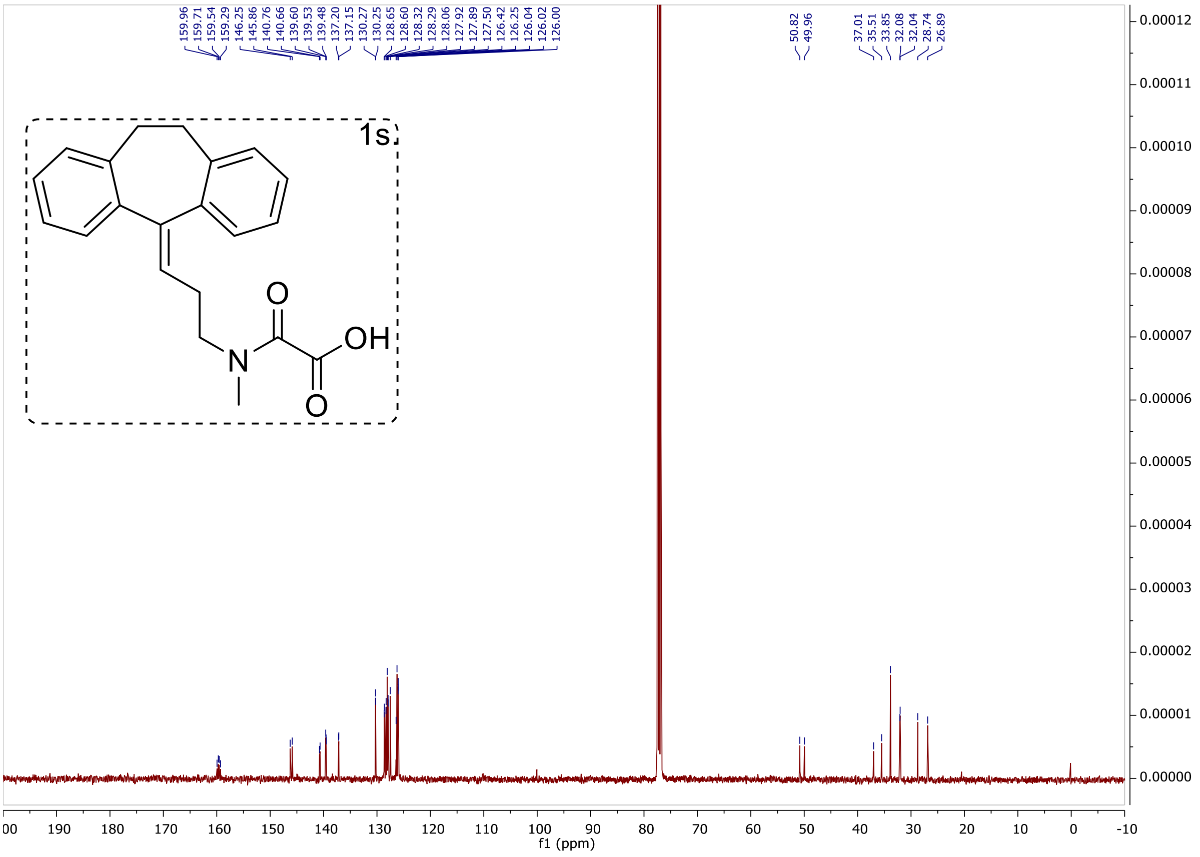

^{13}C NMR of 1s in $CDCl_3$ (101 MHz)

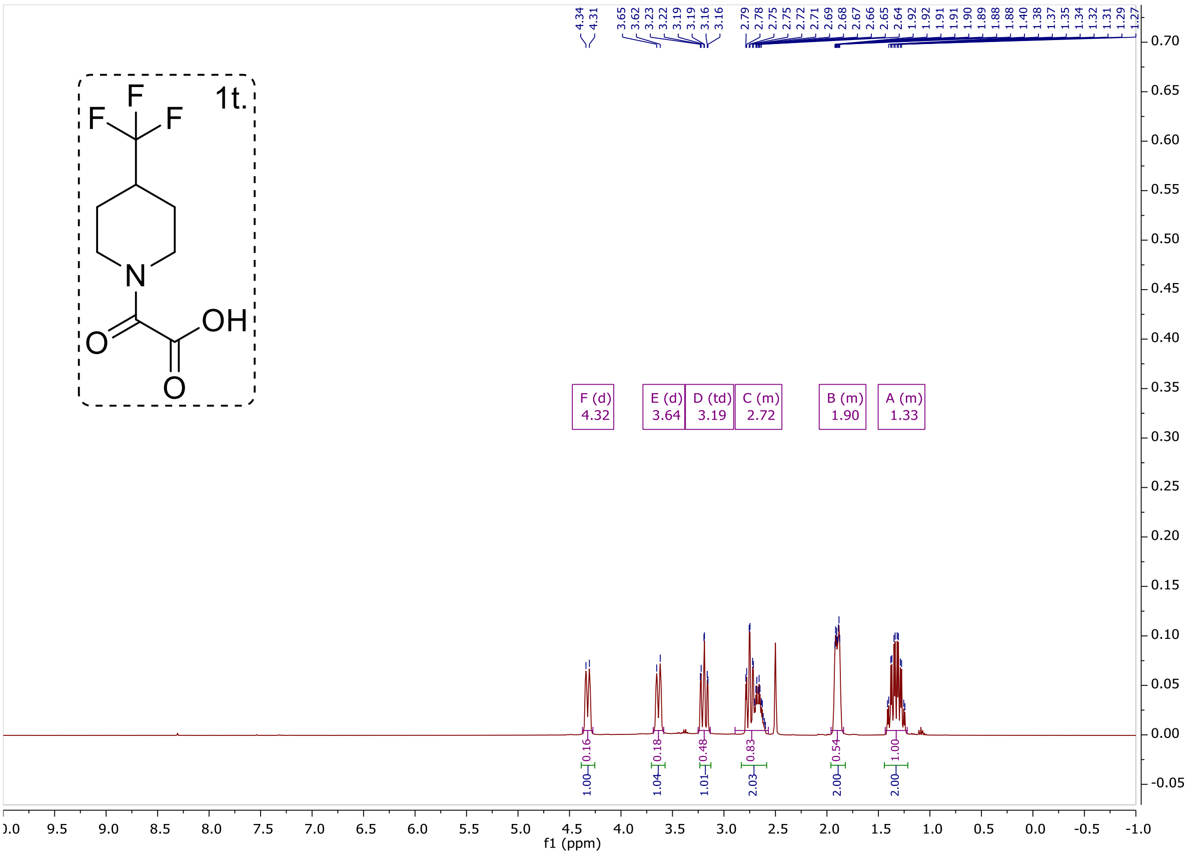

^{1}H NMR of 1t in DMSO-d_6 (400 MHz)

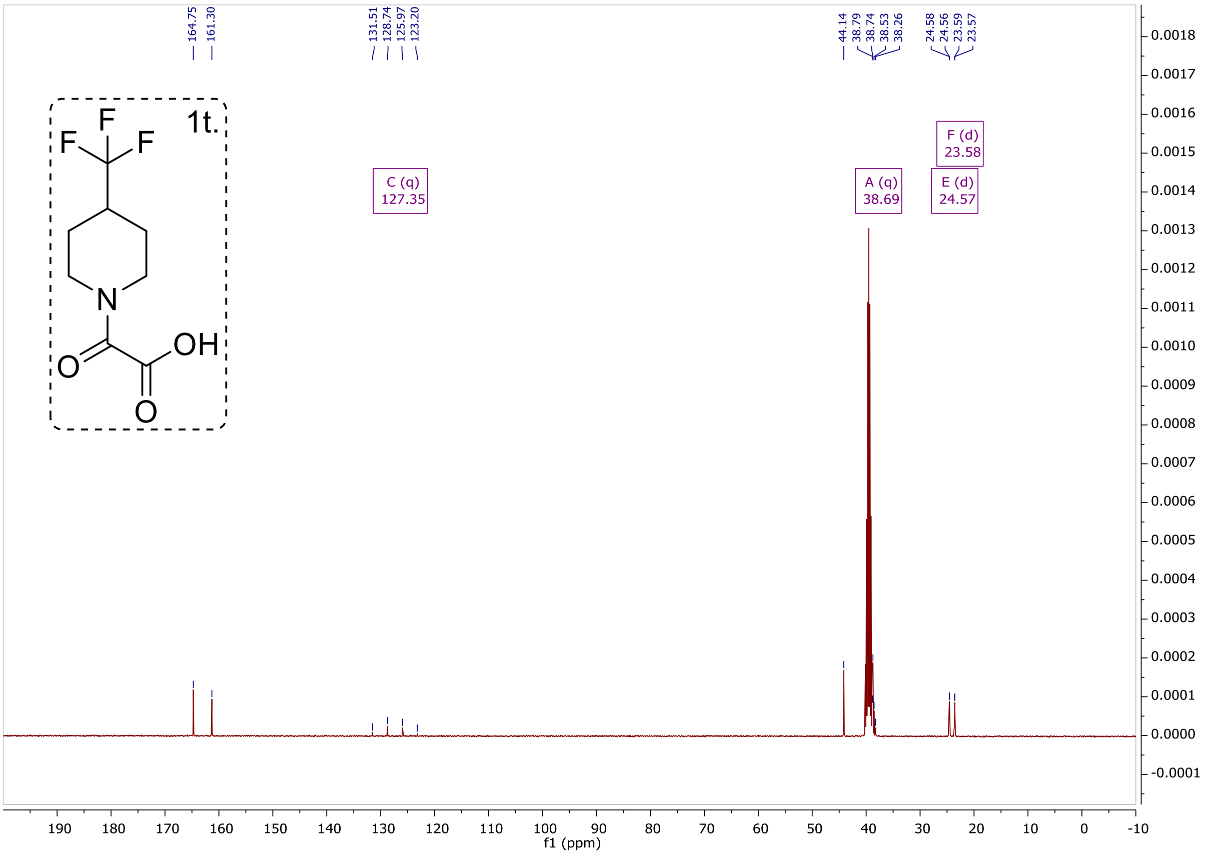

^{13}C NMR of 1t in DMSO-d_6 (101 MHz)

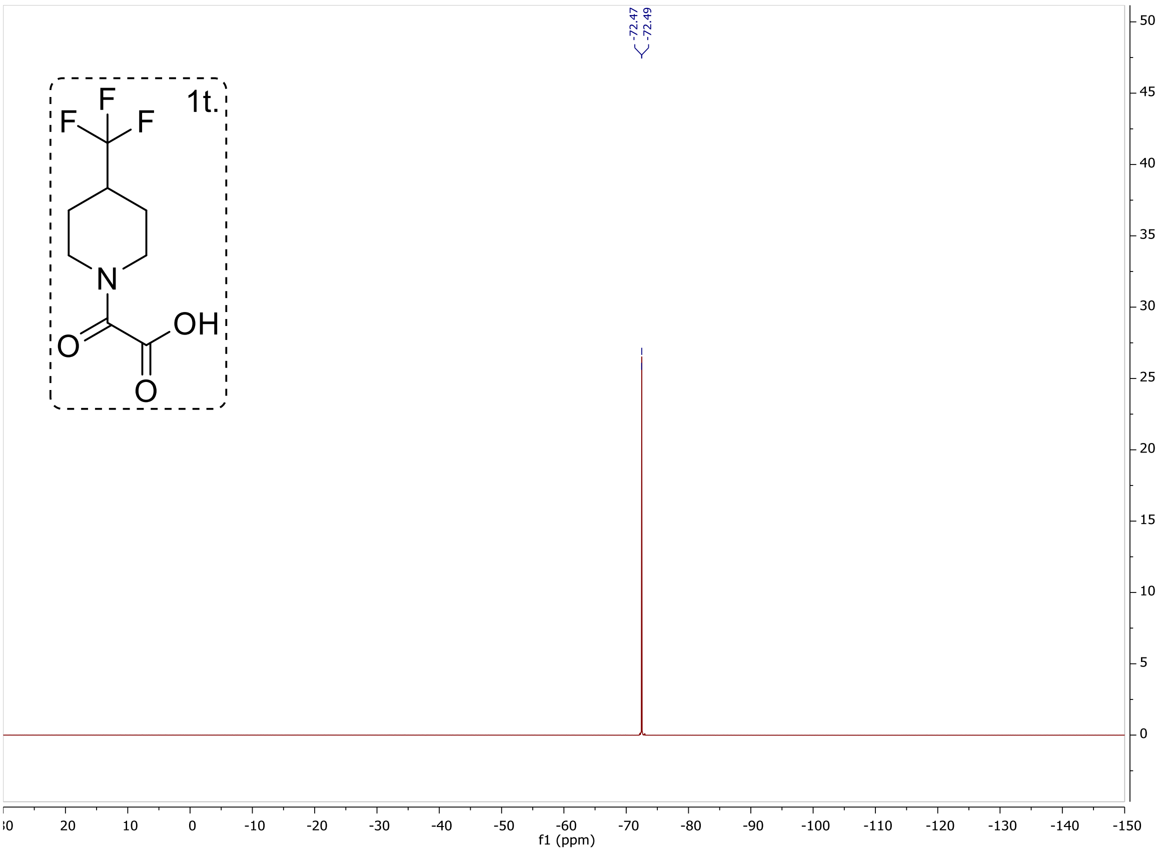

^{19}F NMR of 1t in DMSO-d_6 (376 MHz)

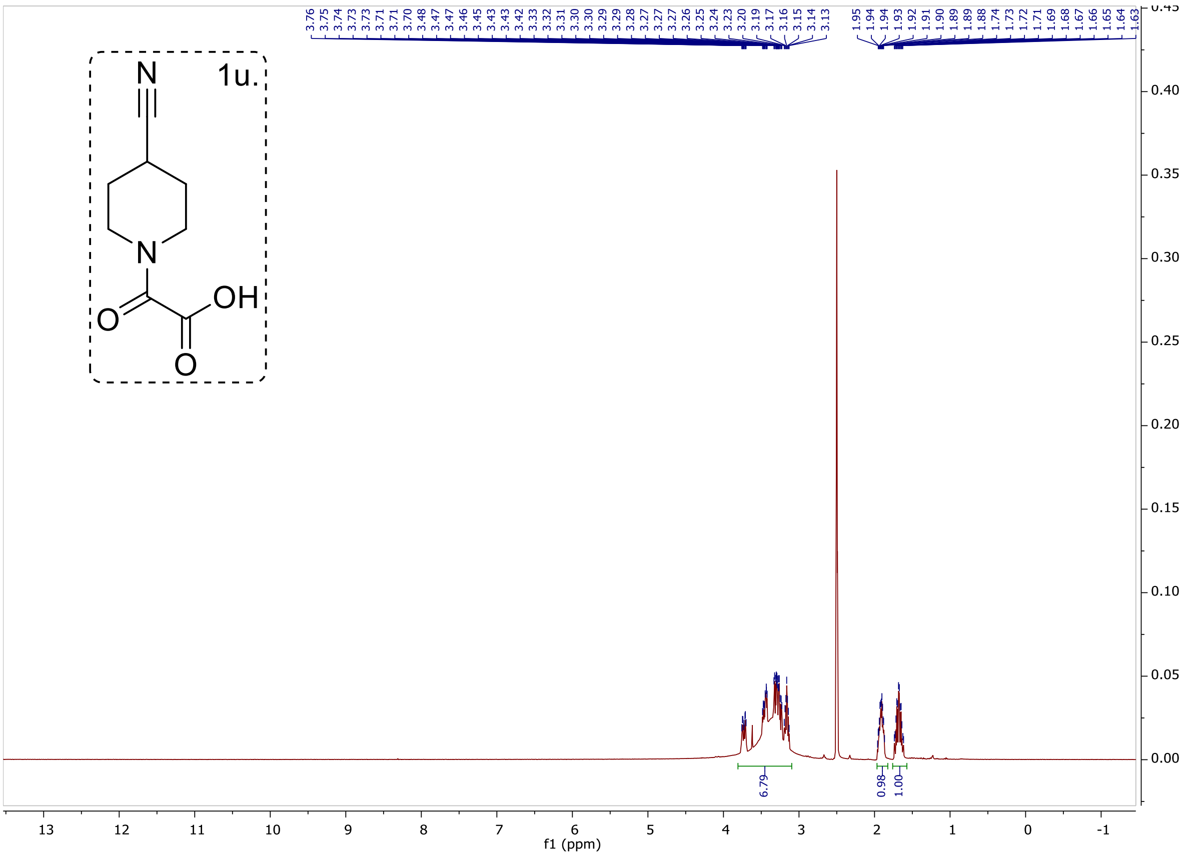

^{1}H NMR of 1u in DMSO-d_6 (400 MHz)

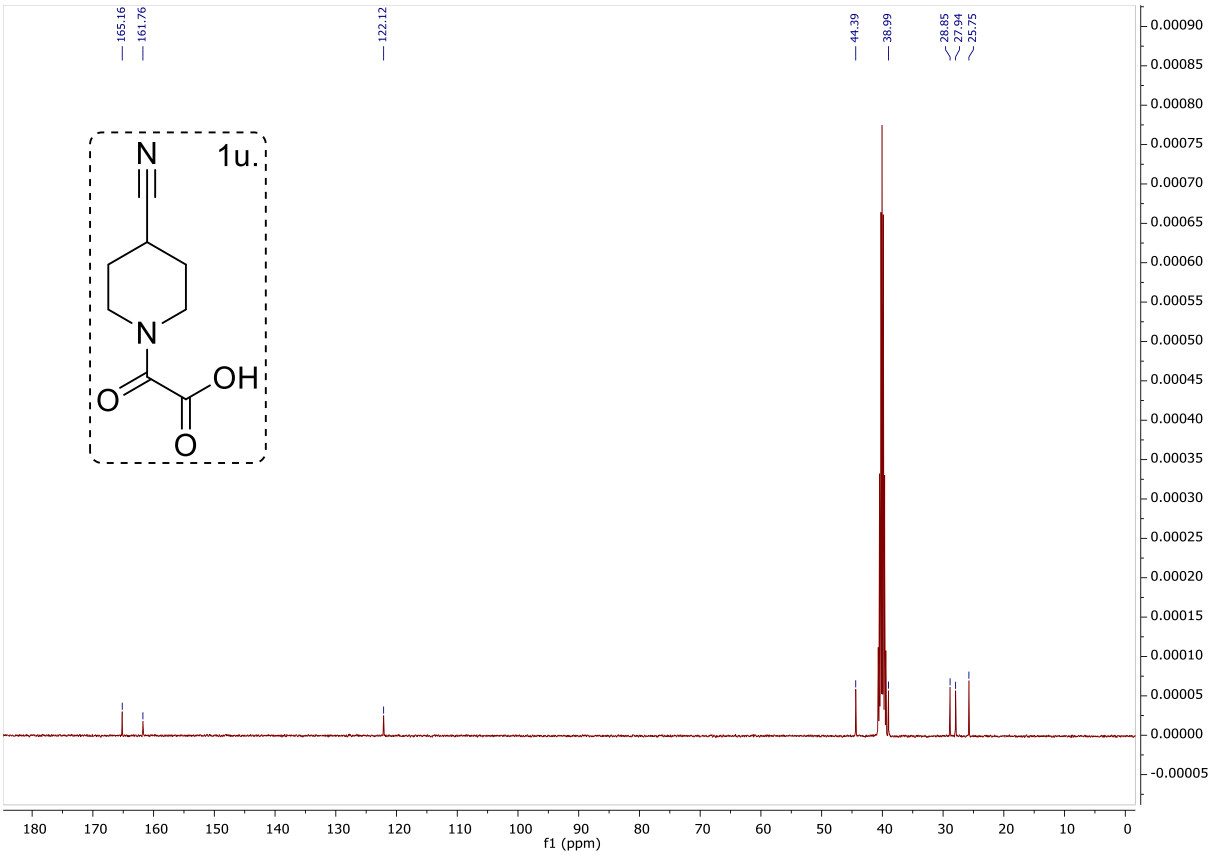

^{13}C NMR of 1u in DMSO-d_6 (101 MHz)

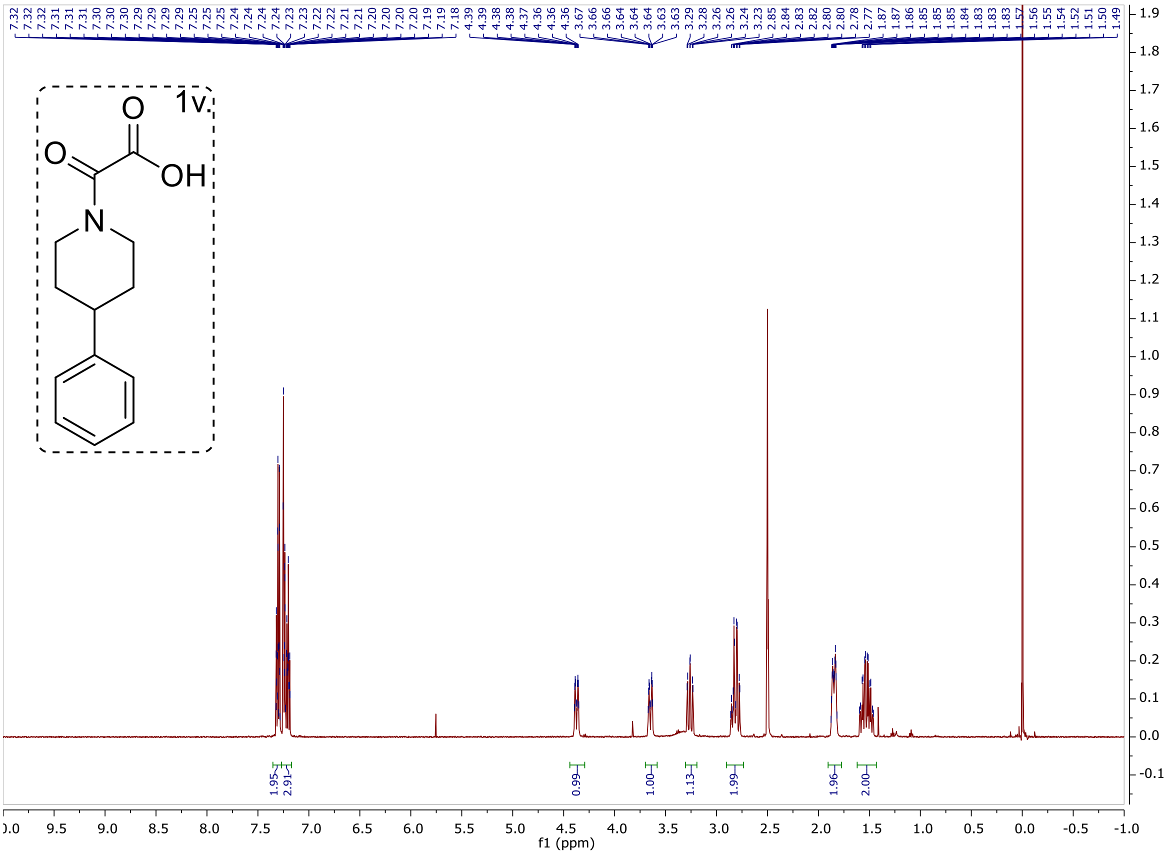

^{1}H NMR of 1v in DMSO-d_6 (500 MHz)

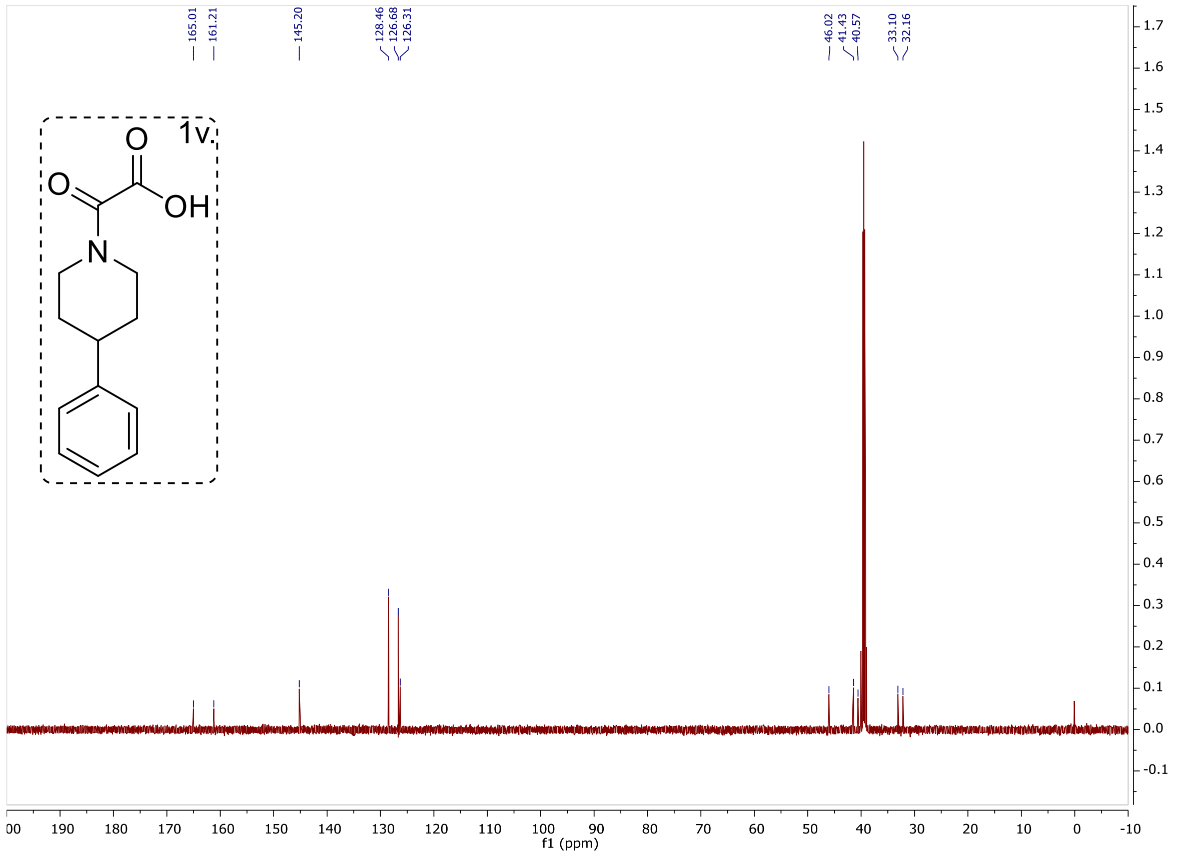

^{13}C NMR of 1v in DMSO-d_6 (126 MHz)

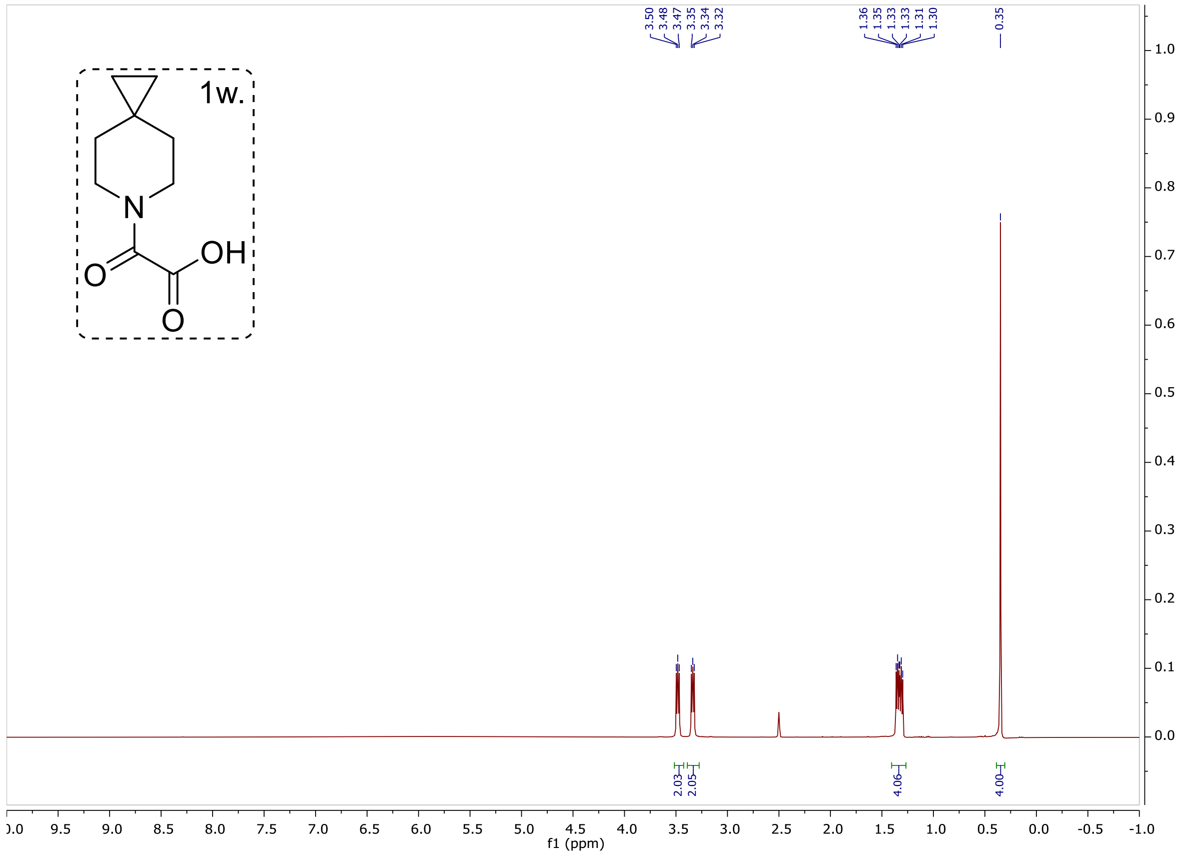

^{1}H NMR of 1w in DMSO-d_6 (400 MHz)

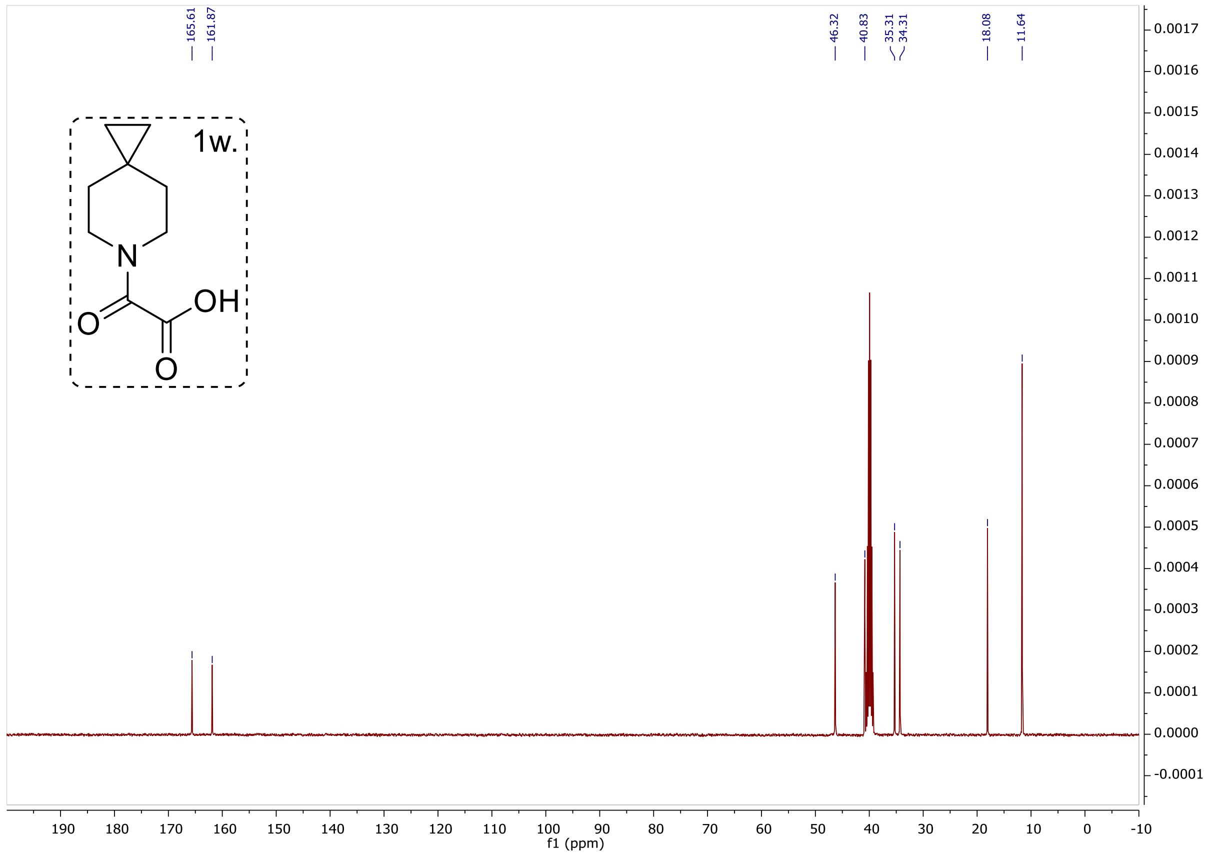

^{13}C NMR of 1w in DMSO-d_6 (101 MHz)

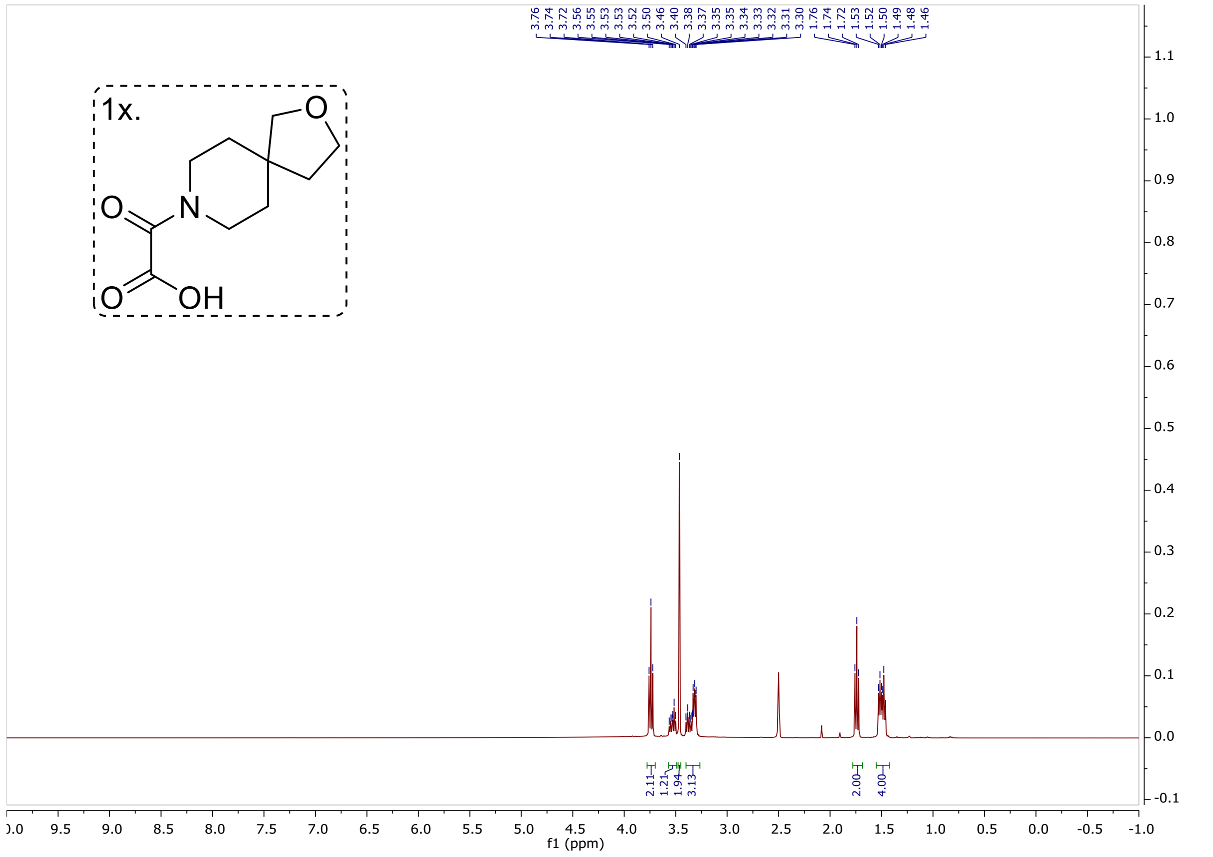

^{1}H NMR of 1x in DMSO-d_6 (400 MHz)

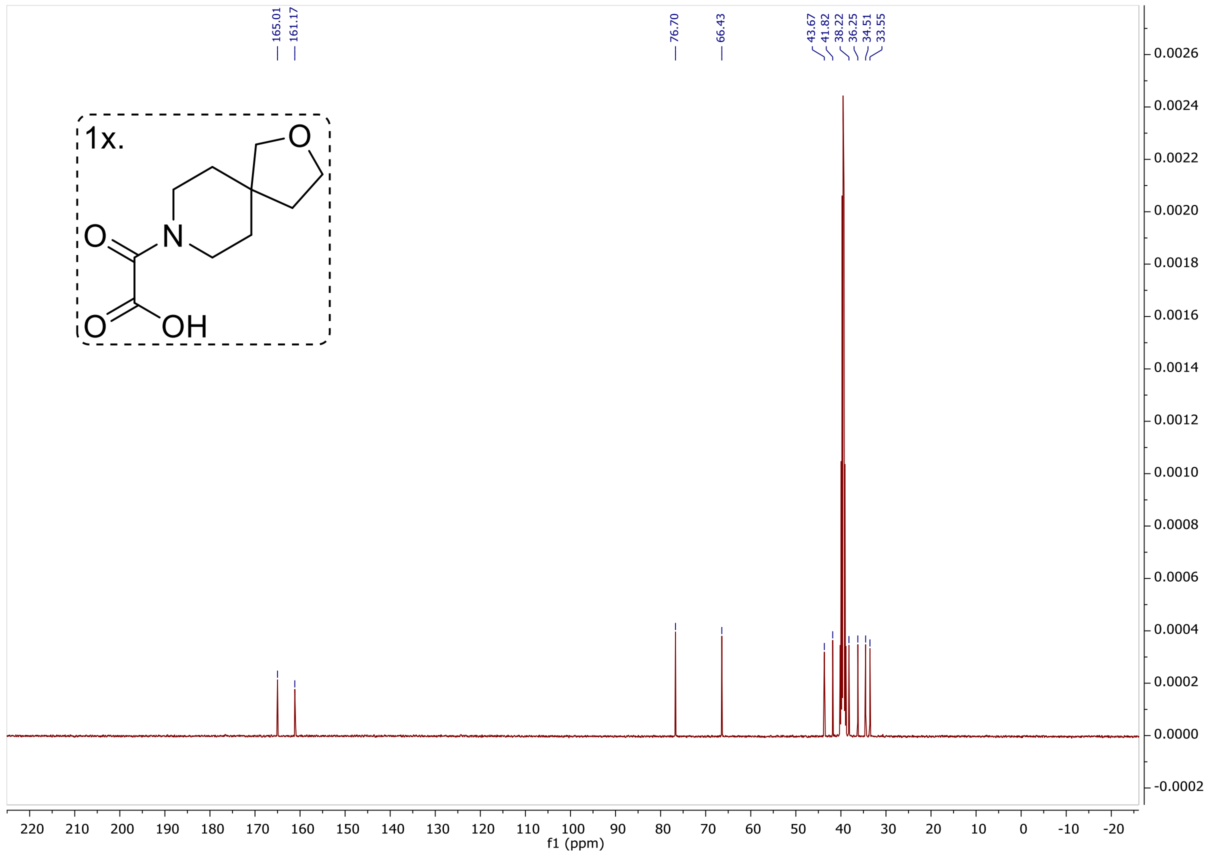

^{13}C NMR of 1x in DMSO-d_6 (101 MHz)

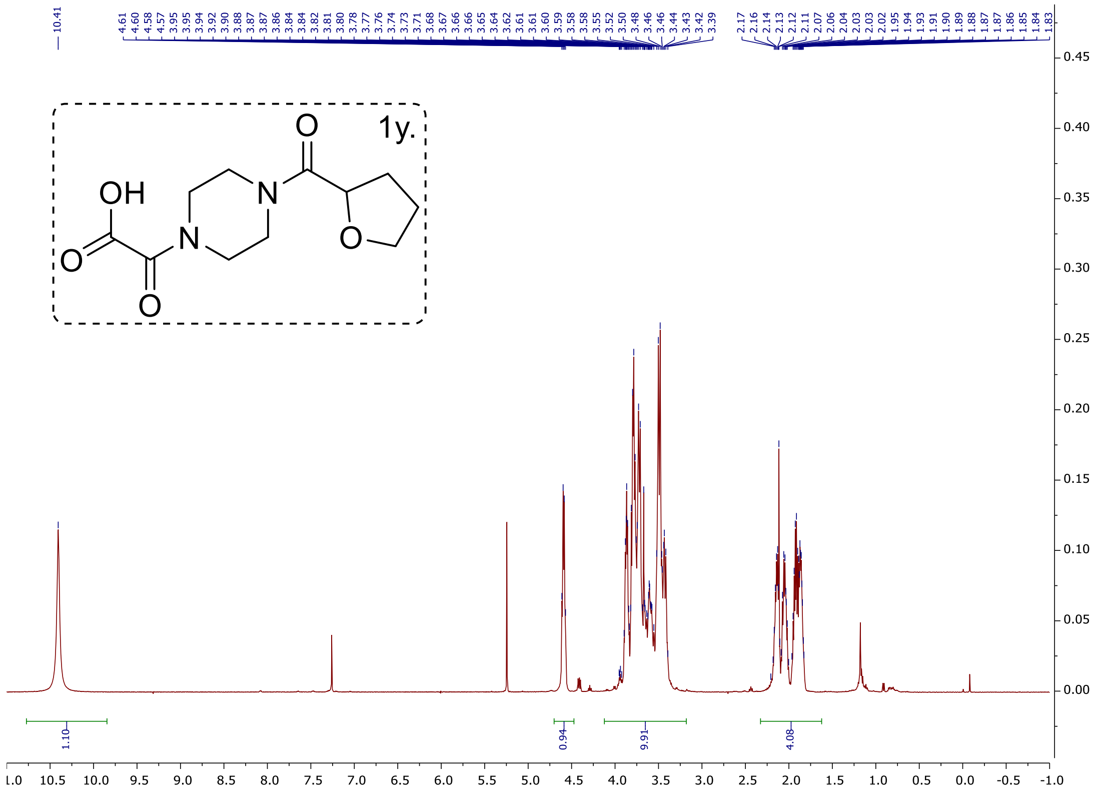

^1H NMR of 1y in $CDCl_3$ (500 MHz)

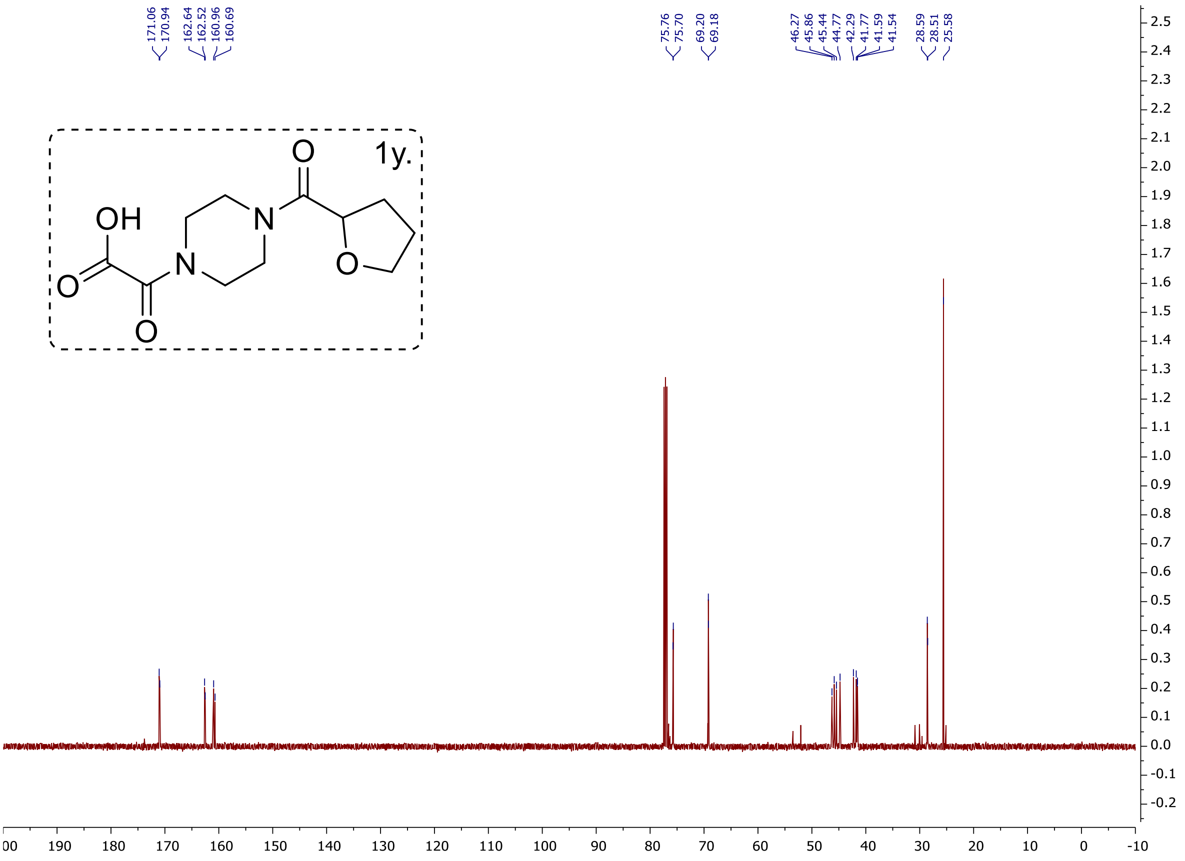

^{13}C NMR of 1y in $CDCl_3$ (126 MHz)

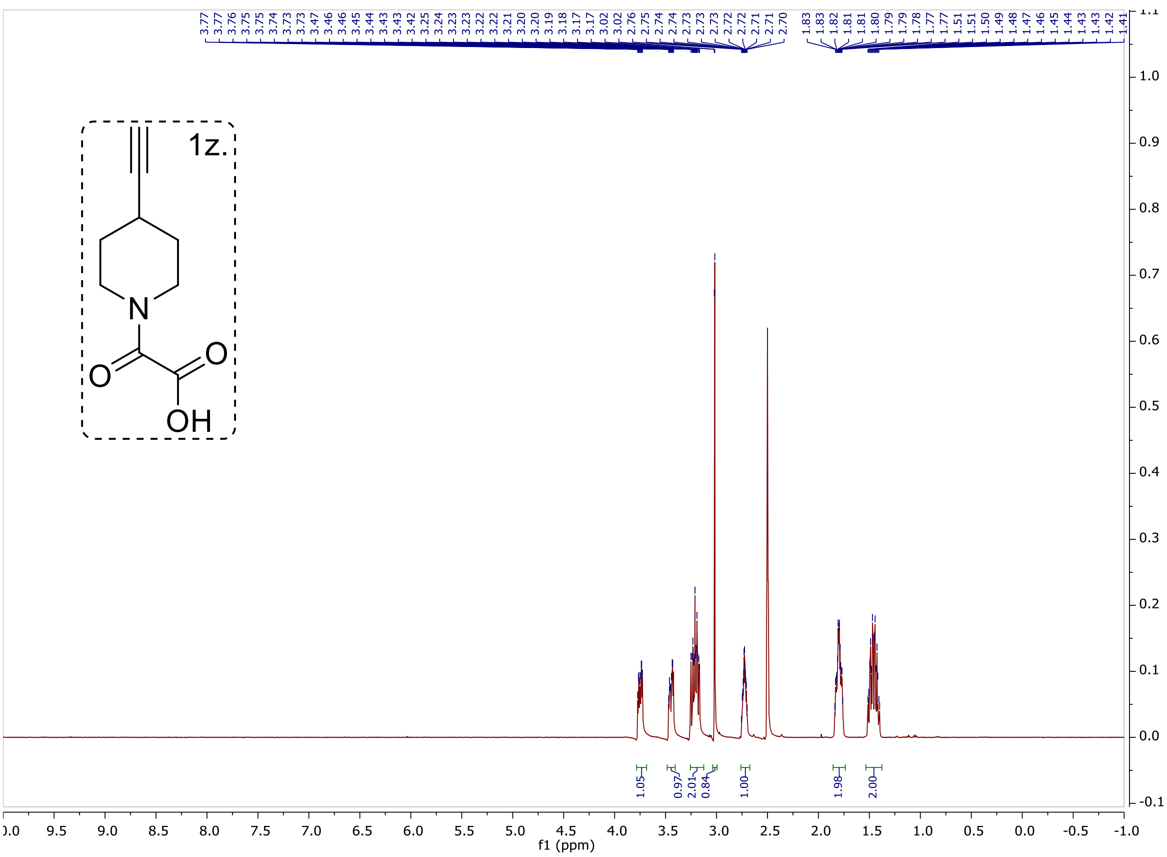

^{1}H NMR of 1z in DMSO-d_6 (500 MHz)

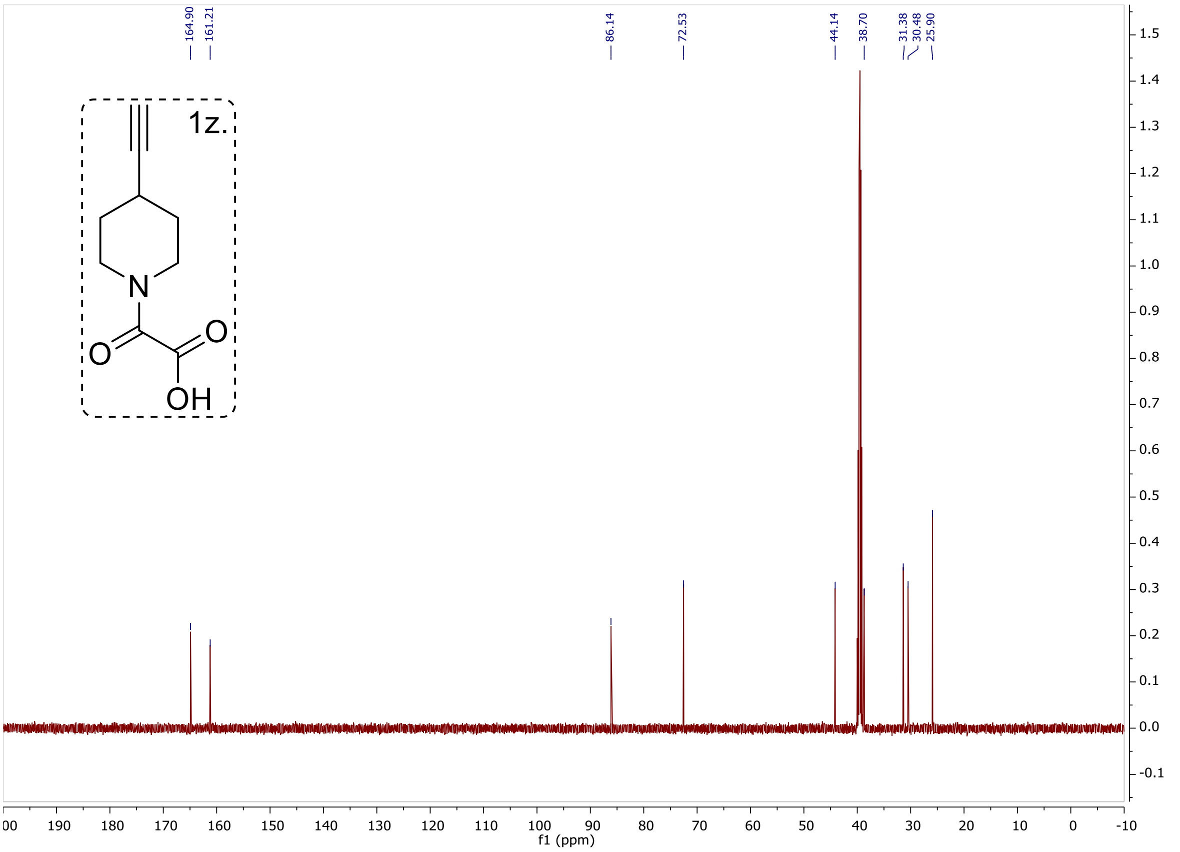

^{13}C NMR of 1z in DMSO-d_6 (126 MHz)

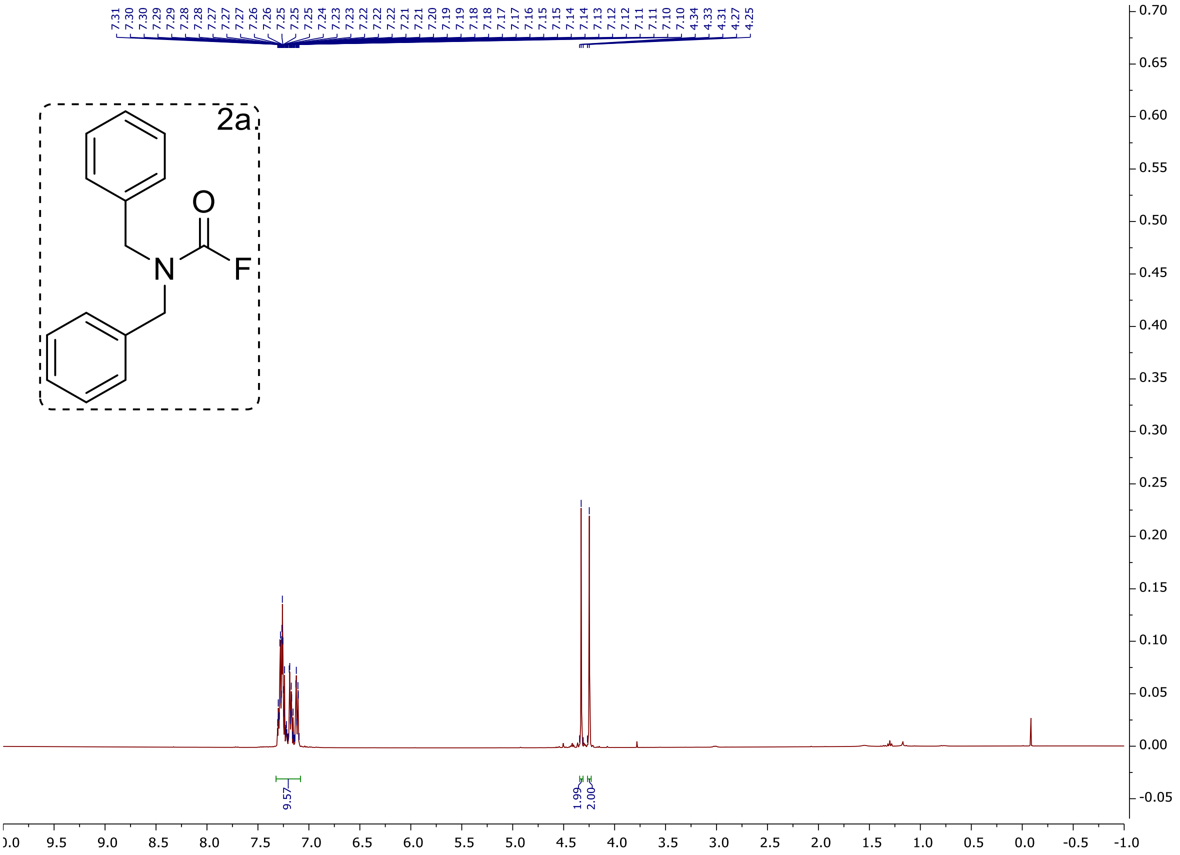

^{1}H NMR of 2a in $CDCl_3$ (400 MHz)

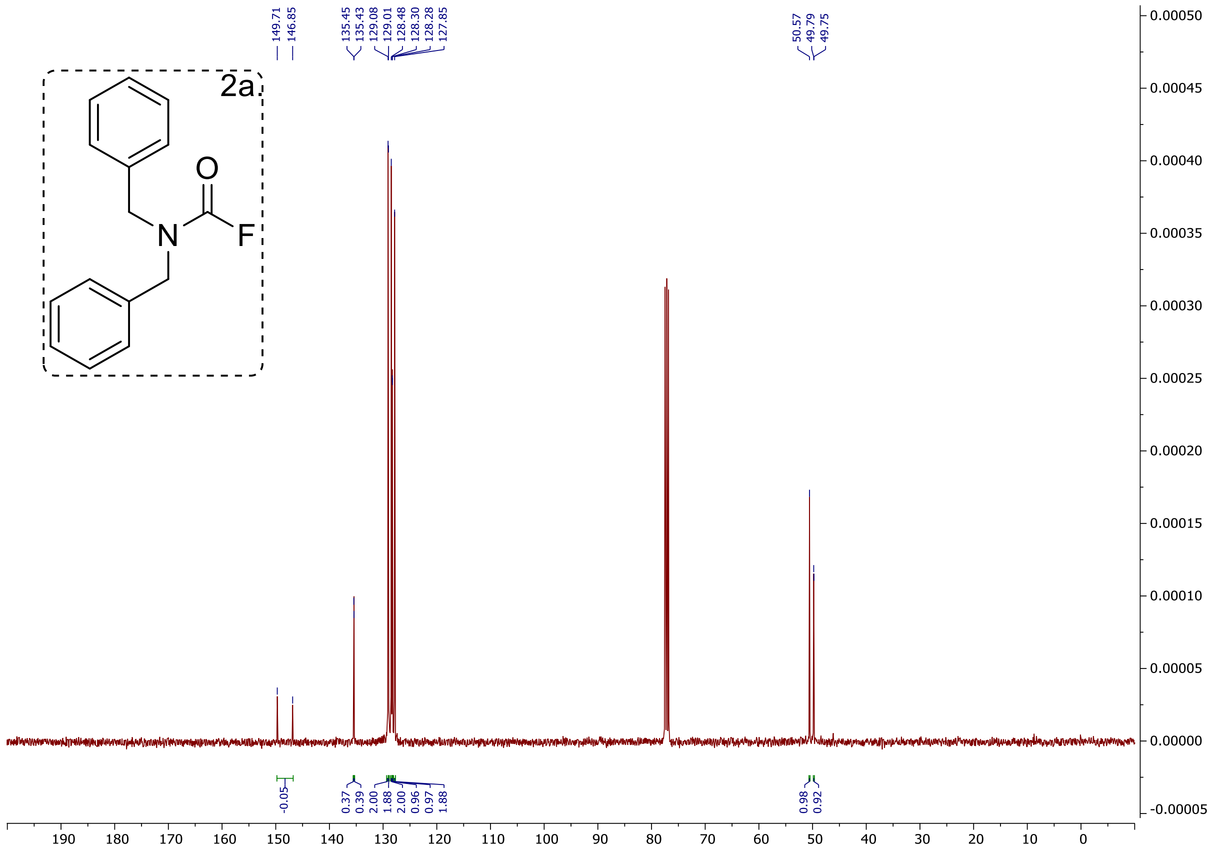

^{13}C NMR of 2a in $CDCl_3$ (101 MHz)

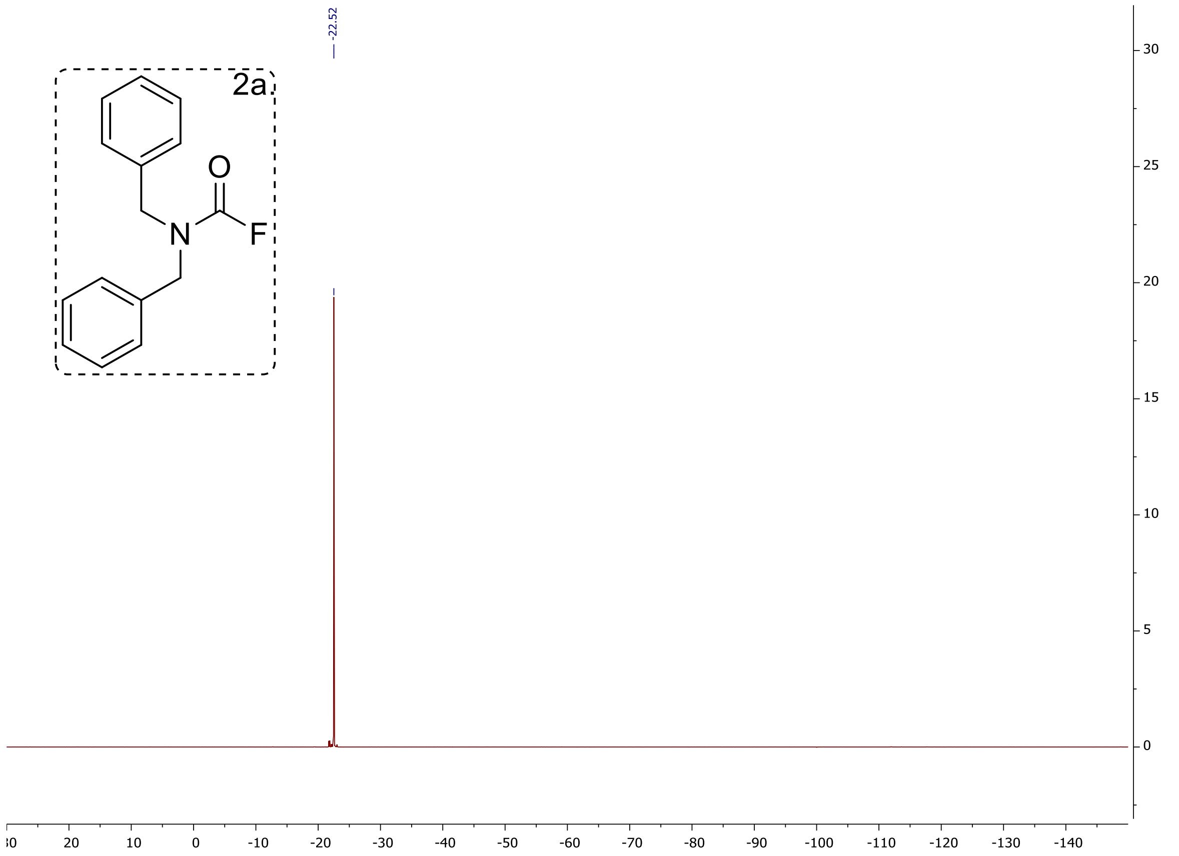

^{19}F NMR of 2b in $CDCl_3$ (376 MHz)

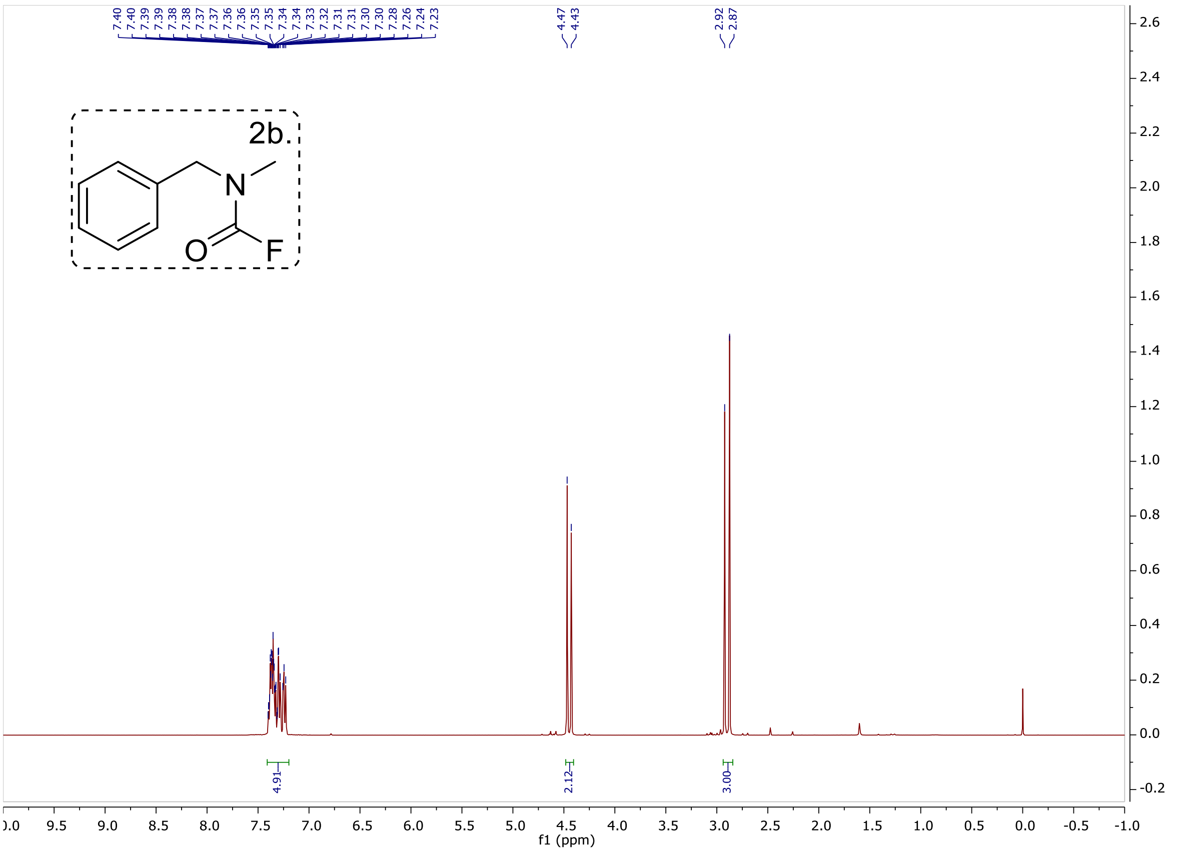

^{1}H NMR of 2b in $CDCl_3$ (400 MHz)

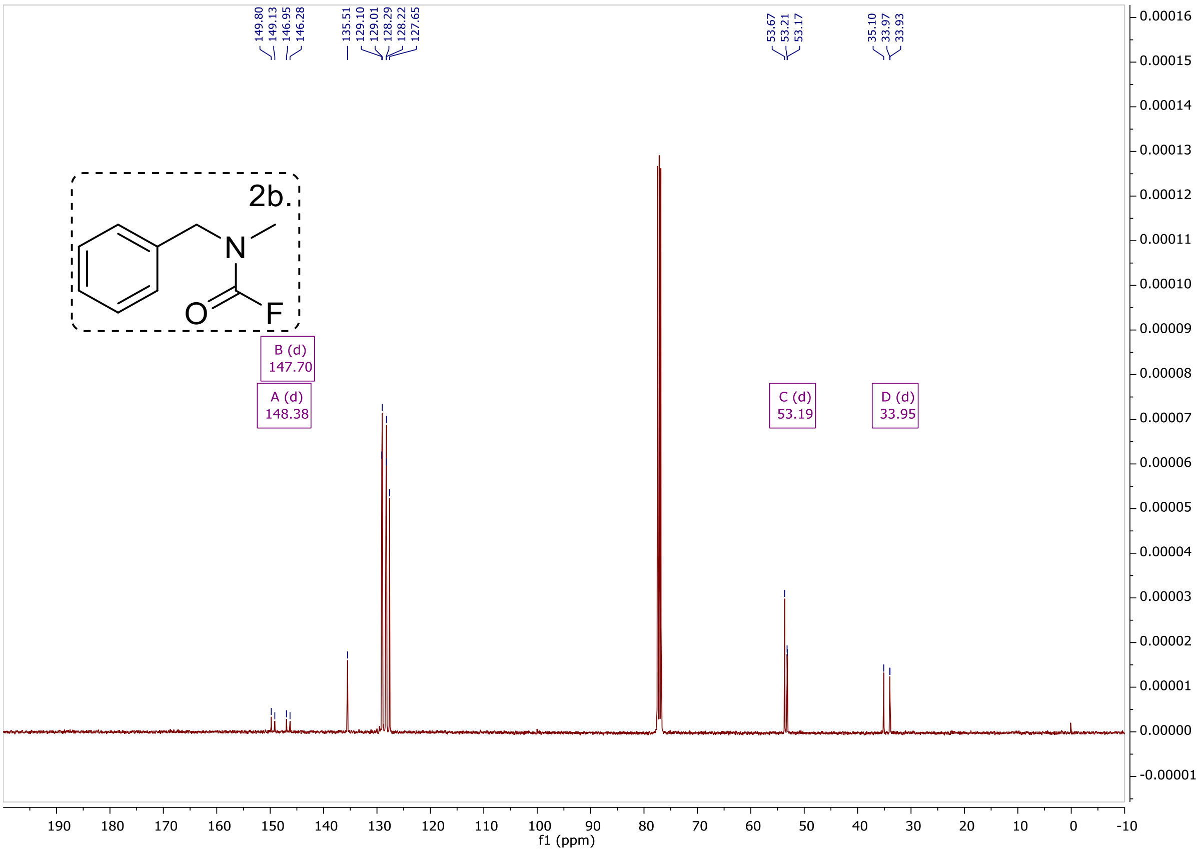

^{13}C NMR of 2b in $CDCl_3$ (101 MHz)

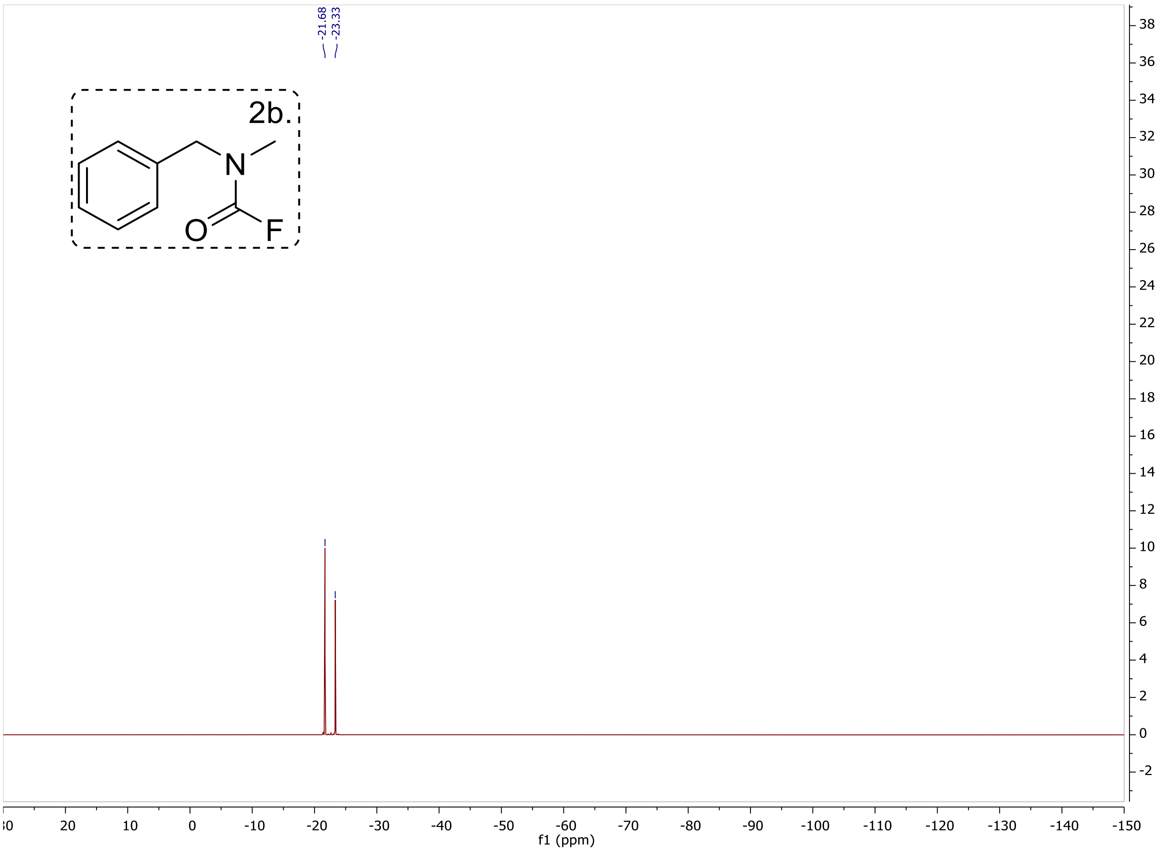

^{19}F NMR of 2b in $CDCl_3$ (376 MHz)

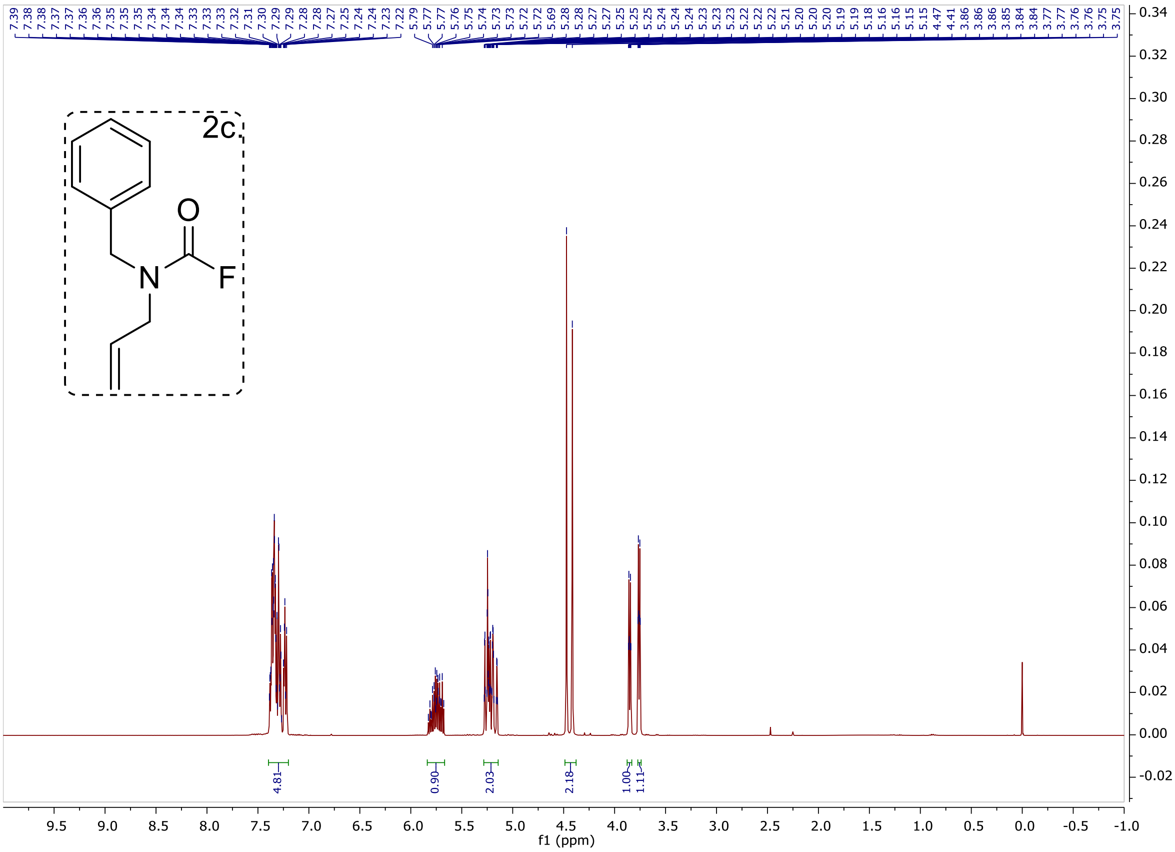

^{1}H NMR of 2c in $CDCl_3$ (400 MHz)

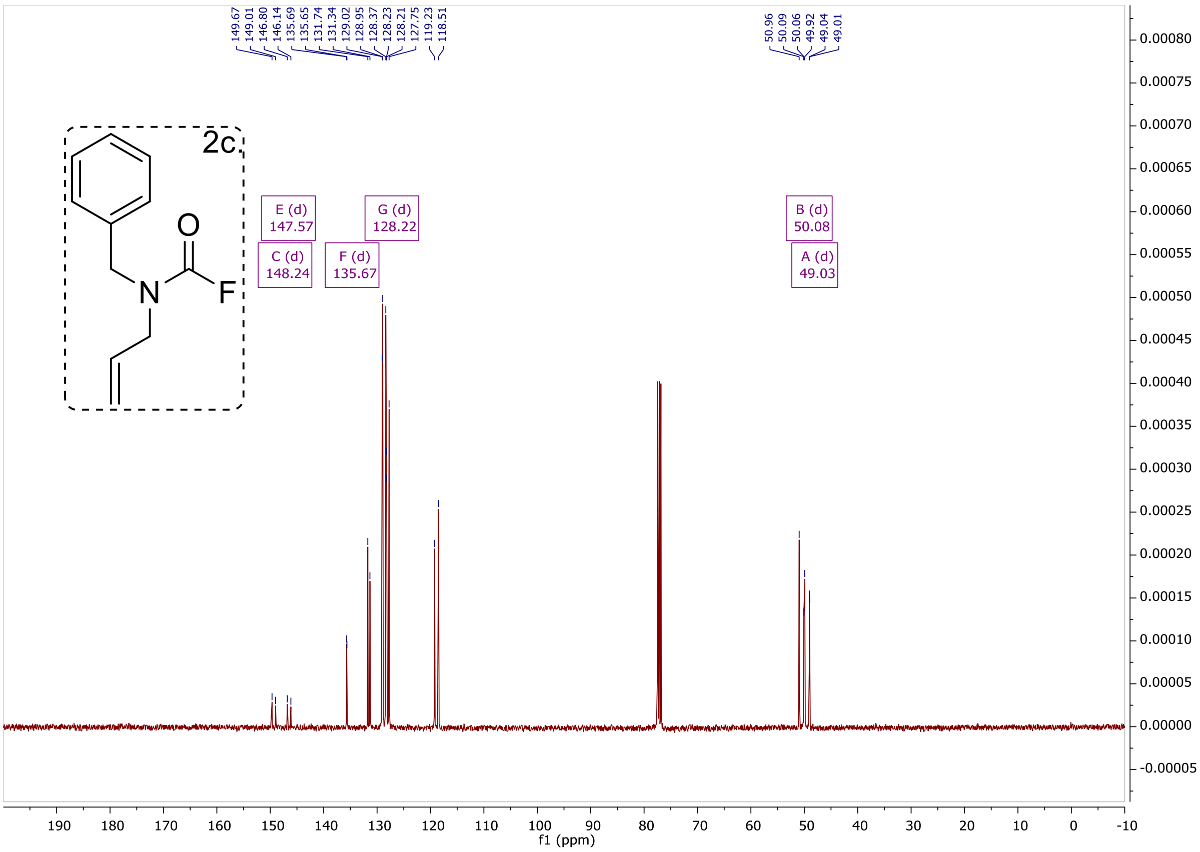

^{13}C NMR of 1c in $CDCl_3$ (101 MHz)

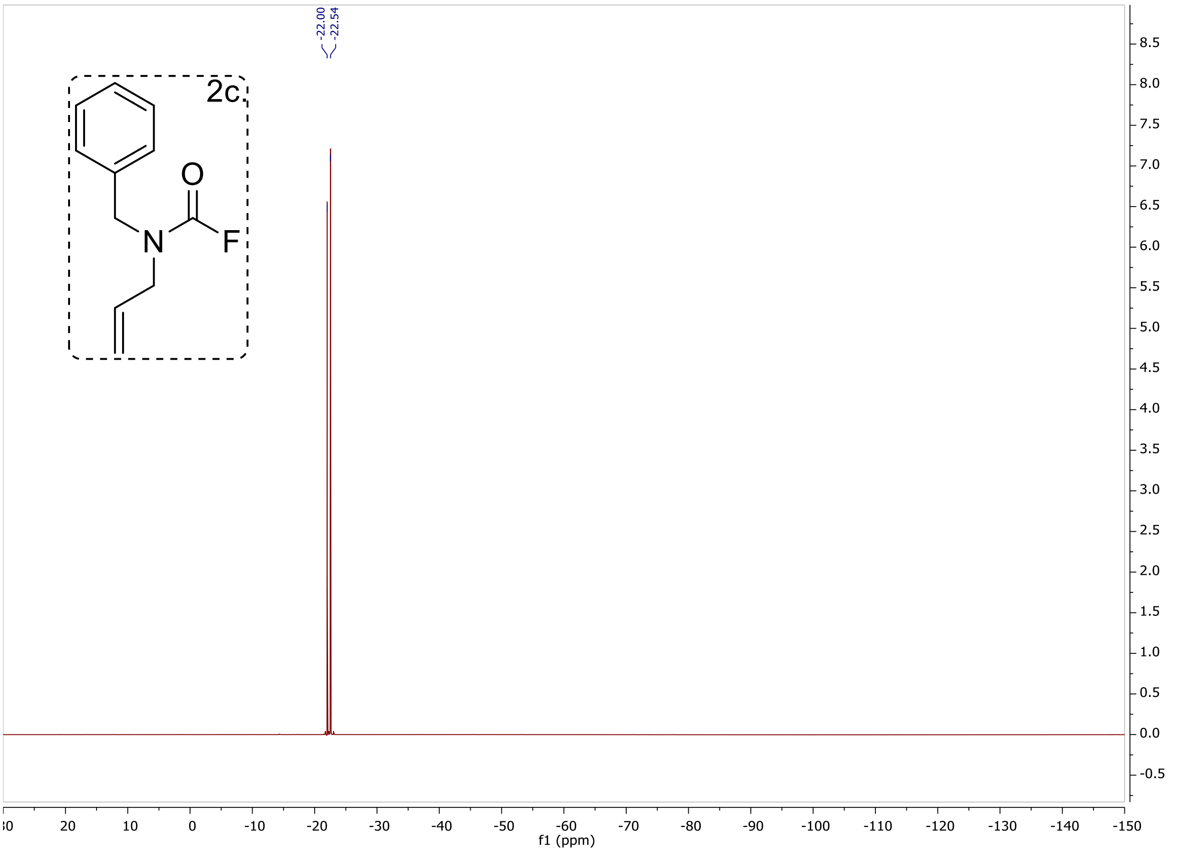

^{19}F NMR of 2c in $CDCl_3$ (376 MHz)

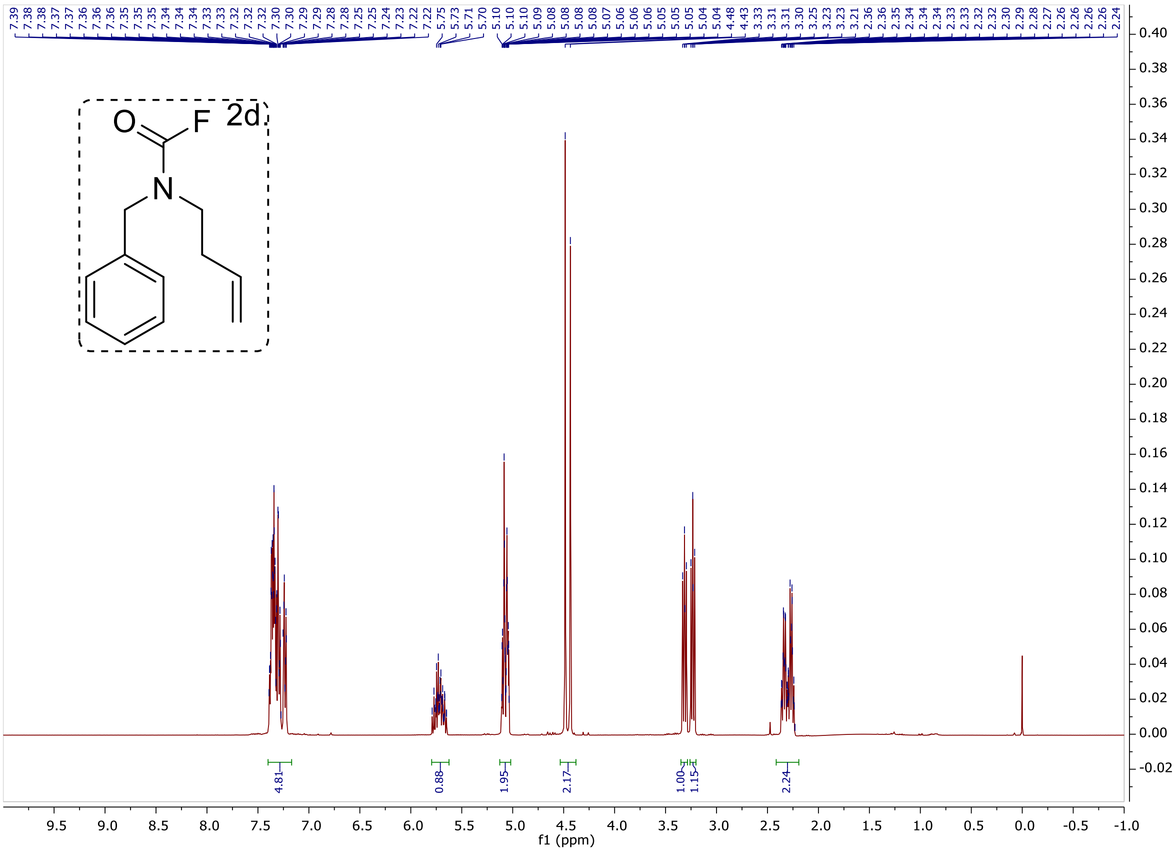

^{1}H NMR of 2d in $CDCl_3$ (400 MHz)

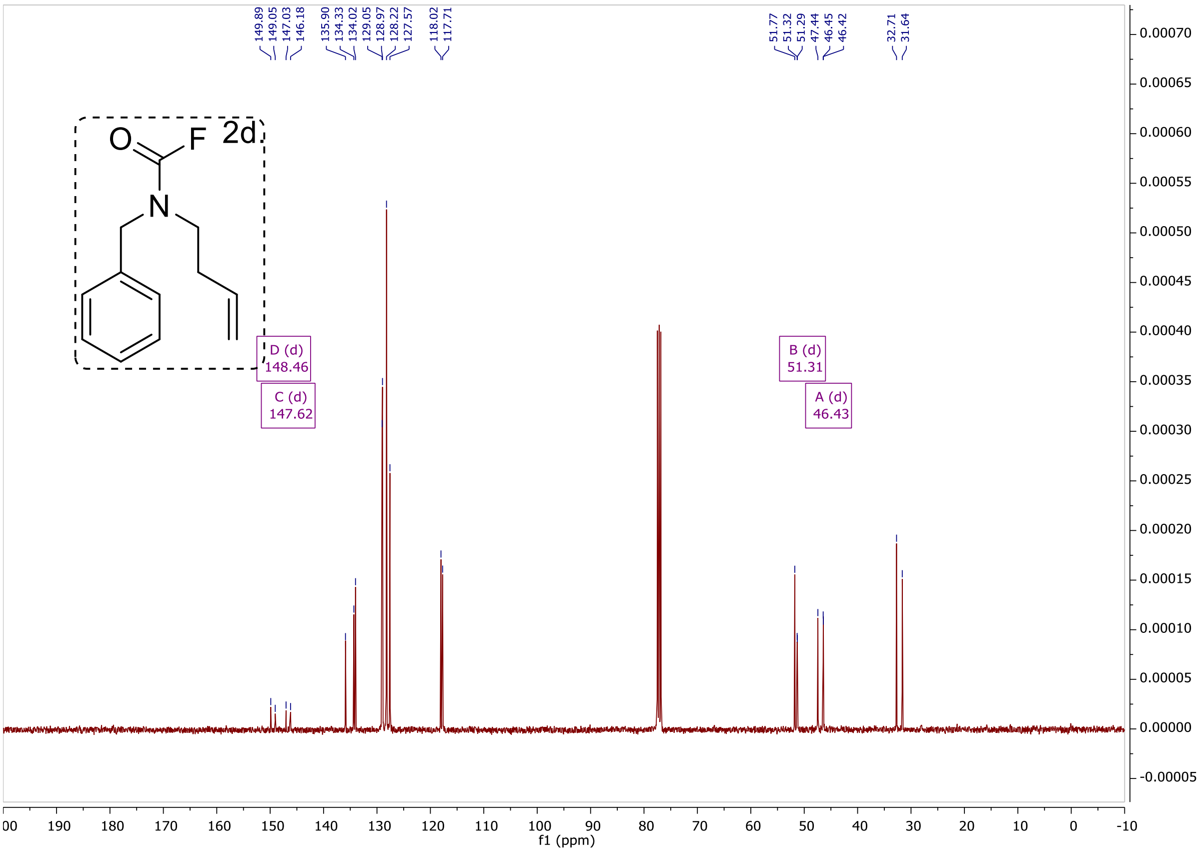

^{13}C NMR of 2d in $CDCl_3$ (101 MHz)

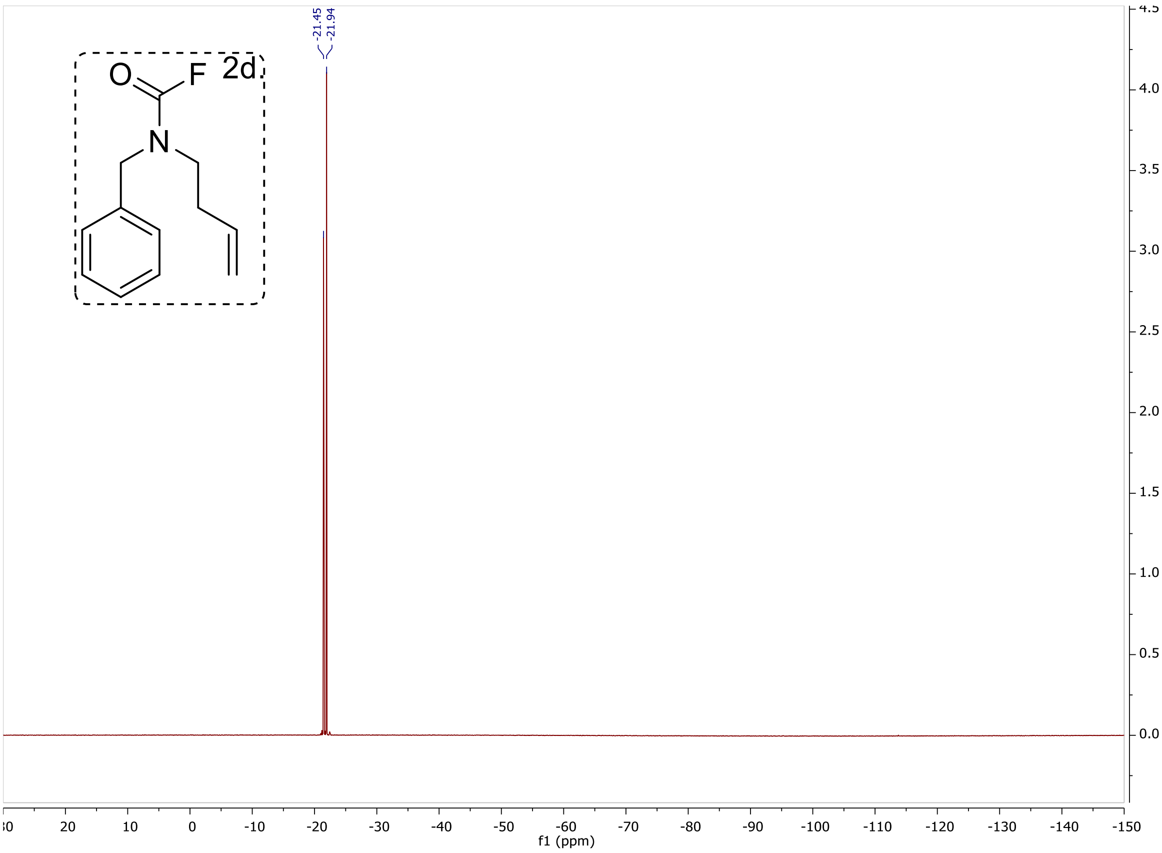

^{19}F NMR of 2d in $CDCl_3$ (376 MHz)

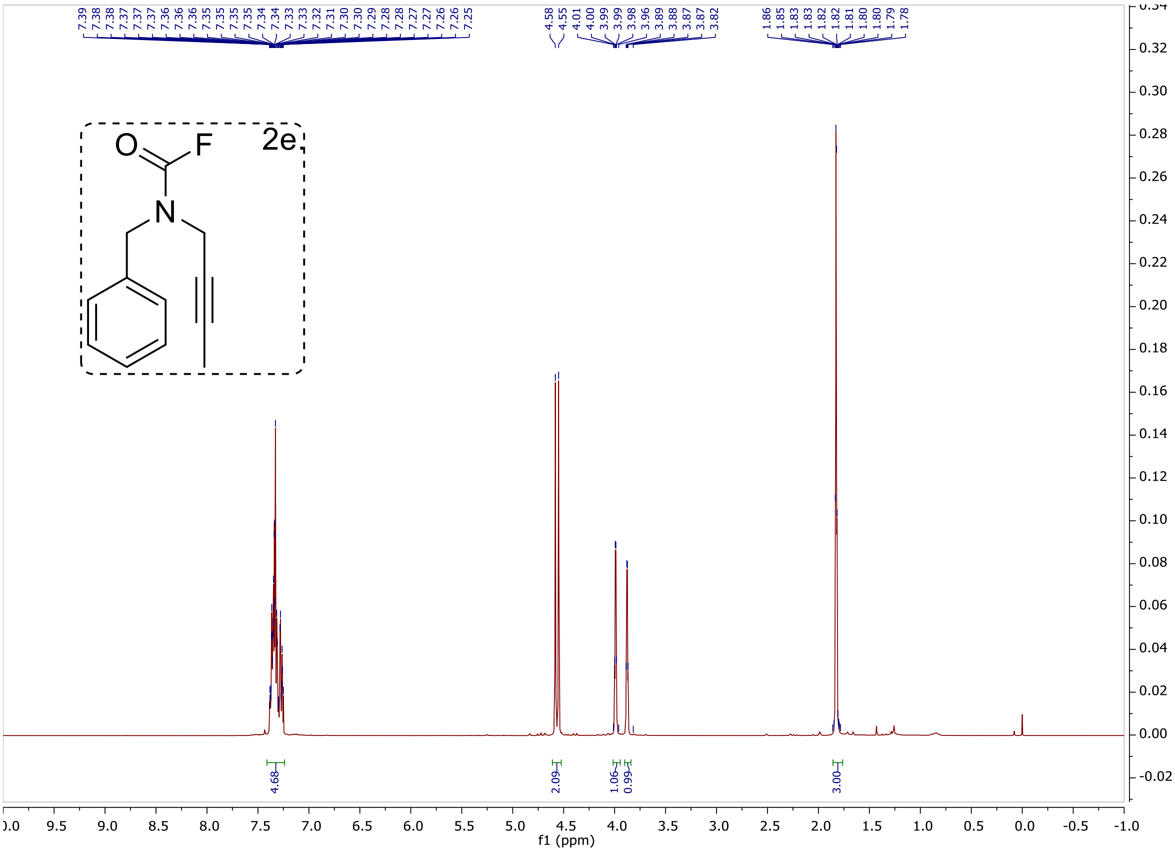

^{1}H NMR of 2e in $CDCl_3$ (400 MHz)

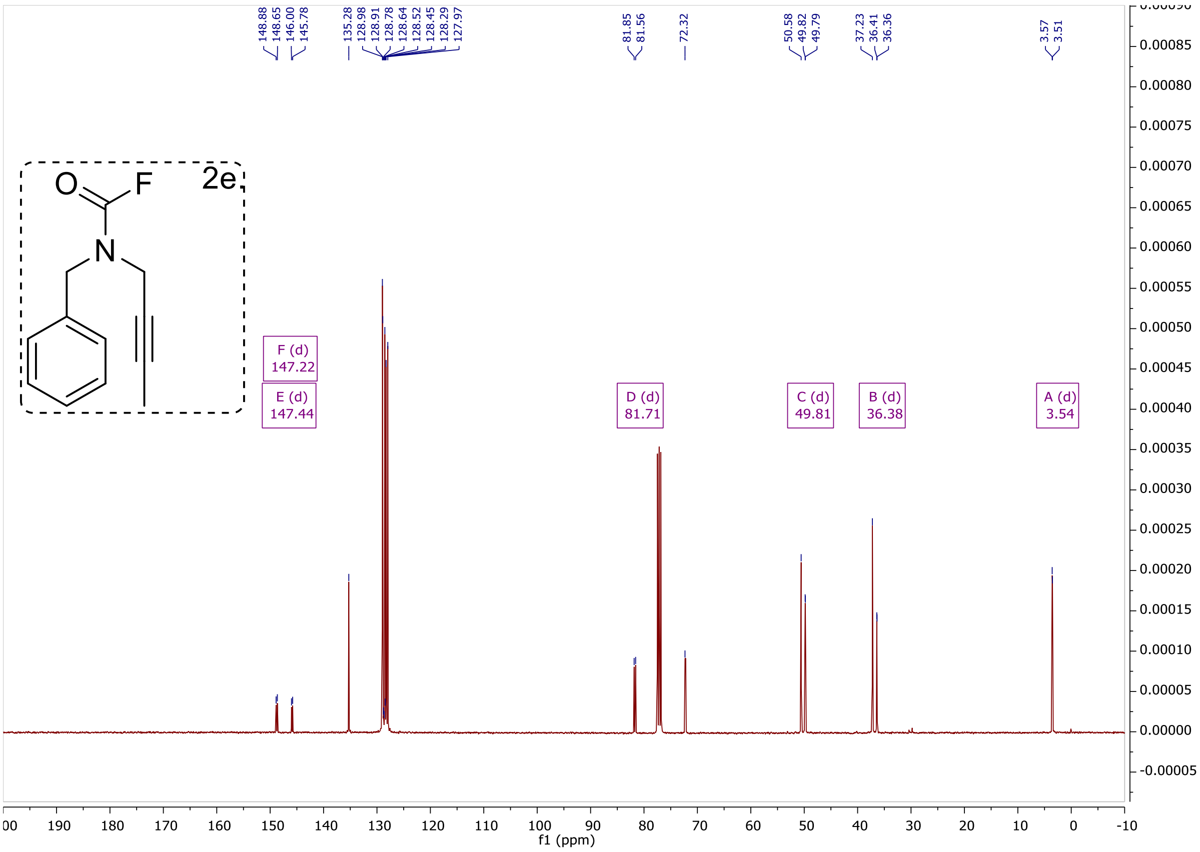

^{13}C NMR of 2e in $CDCl_3$ (101 MHz)

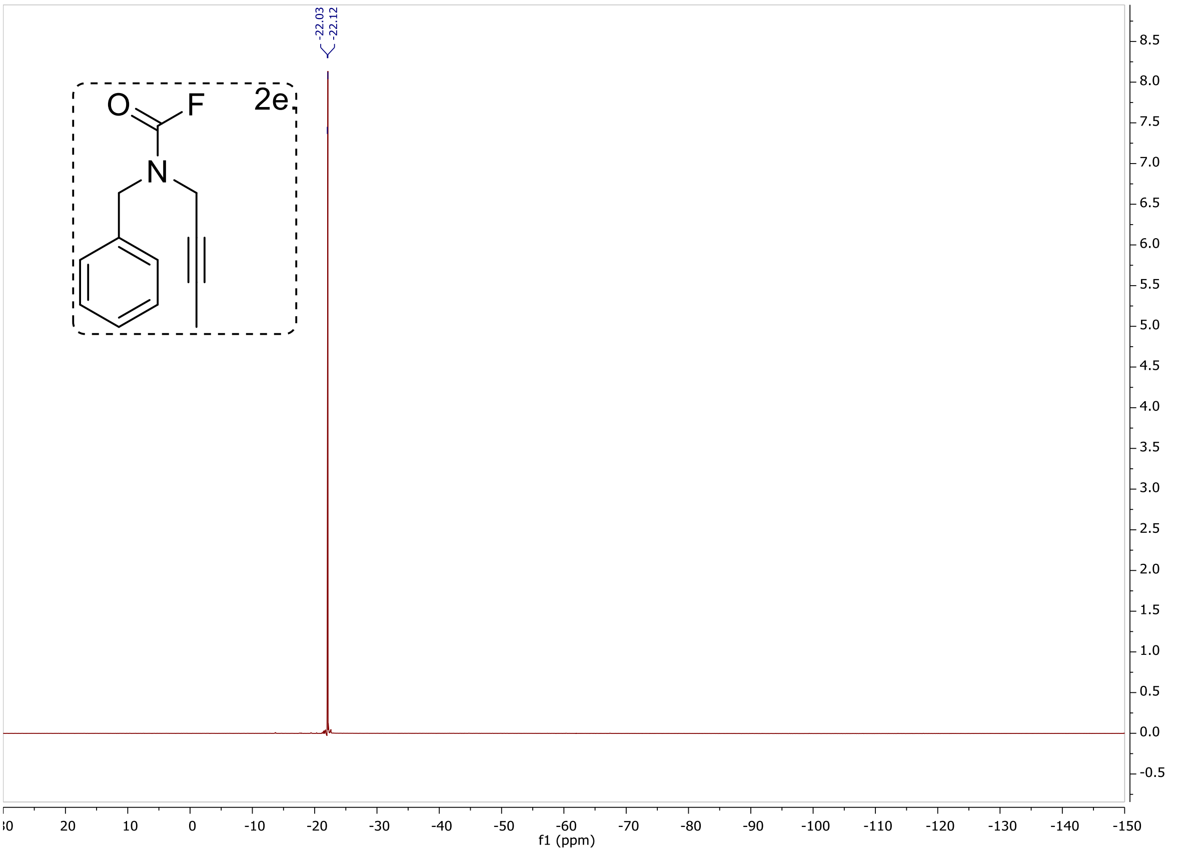

^{19}F NMR of 2e in $CDCl_3$ (376 MHz)

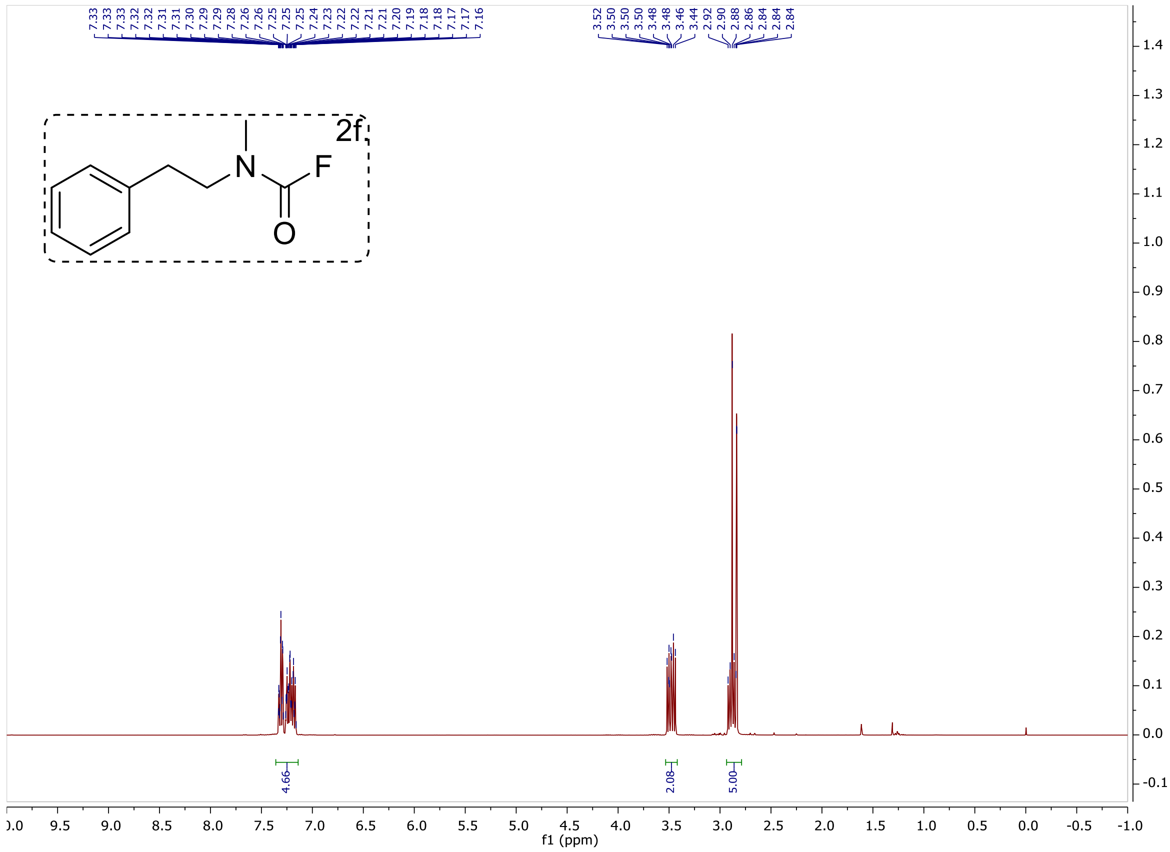

^{1}H NMR of 2f in $CDCl_3$ (400 MHz)

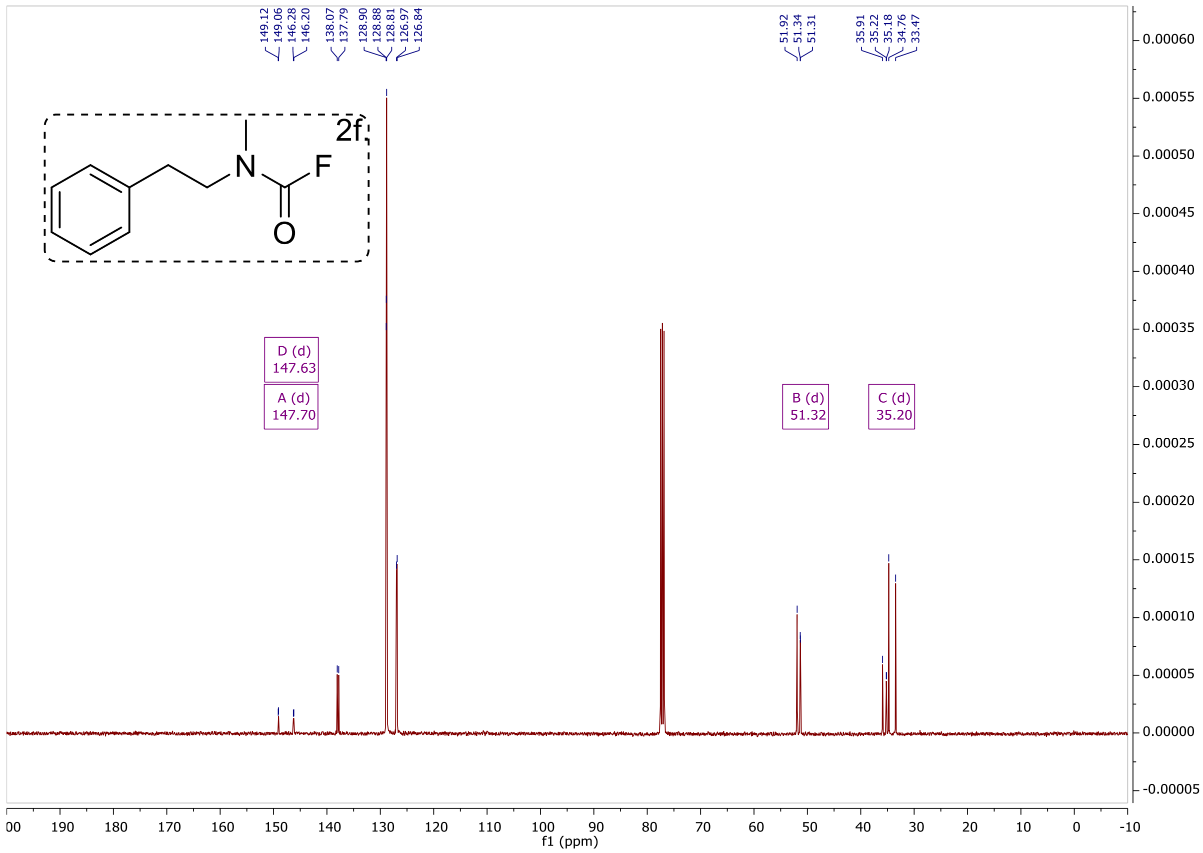

^{13}C NMR of 2f in $CDCl_3$ (101 MHz)

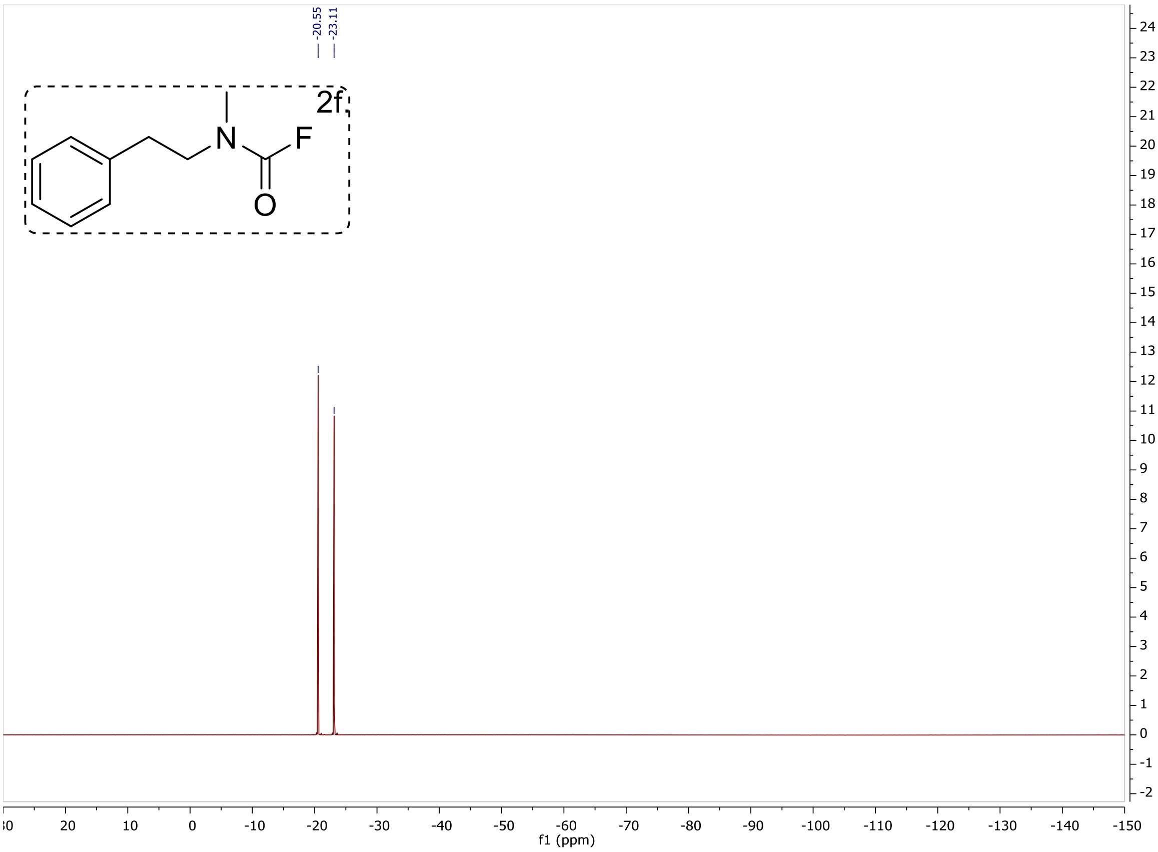

^{19}F NMR of 2f in $CDCl_3$ (376 MHz)

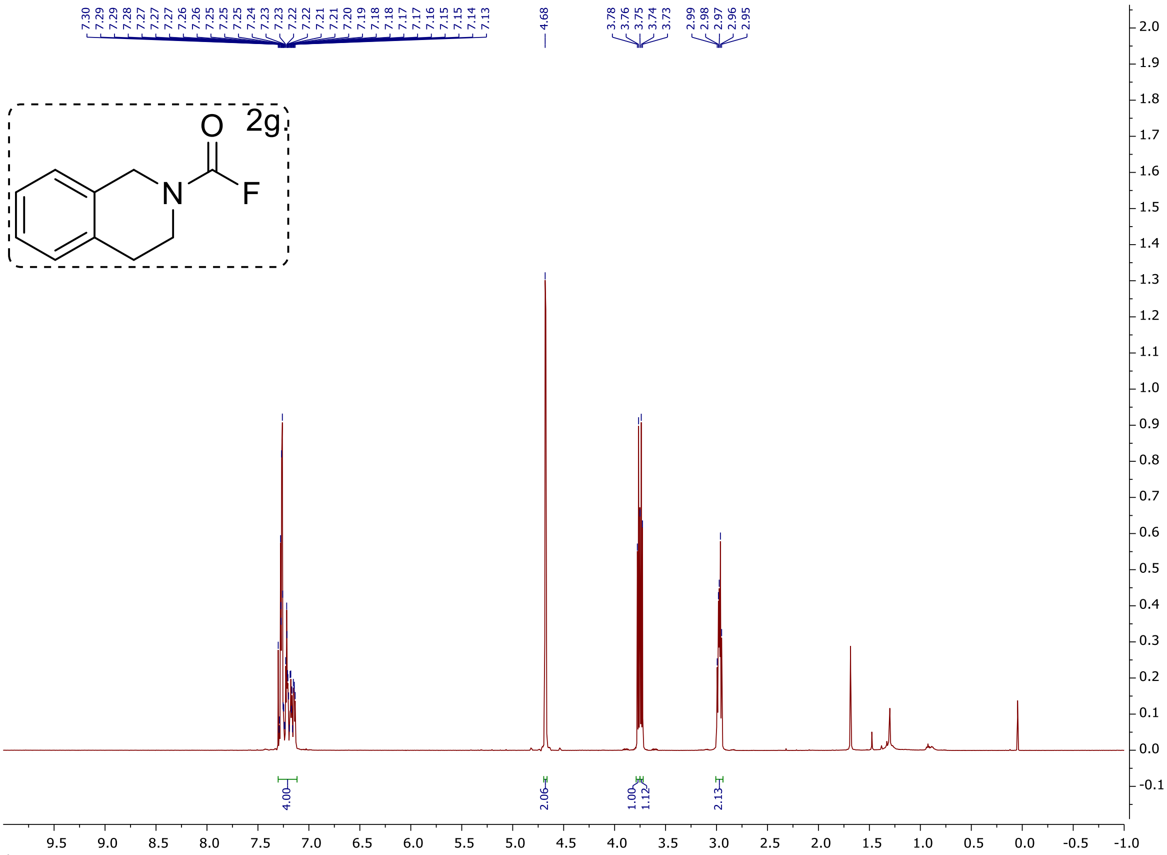

^{1}H NMR of 2g in $CDCl_3$ (500 MHz)

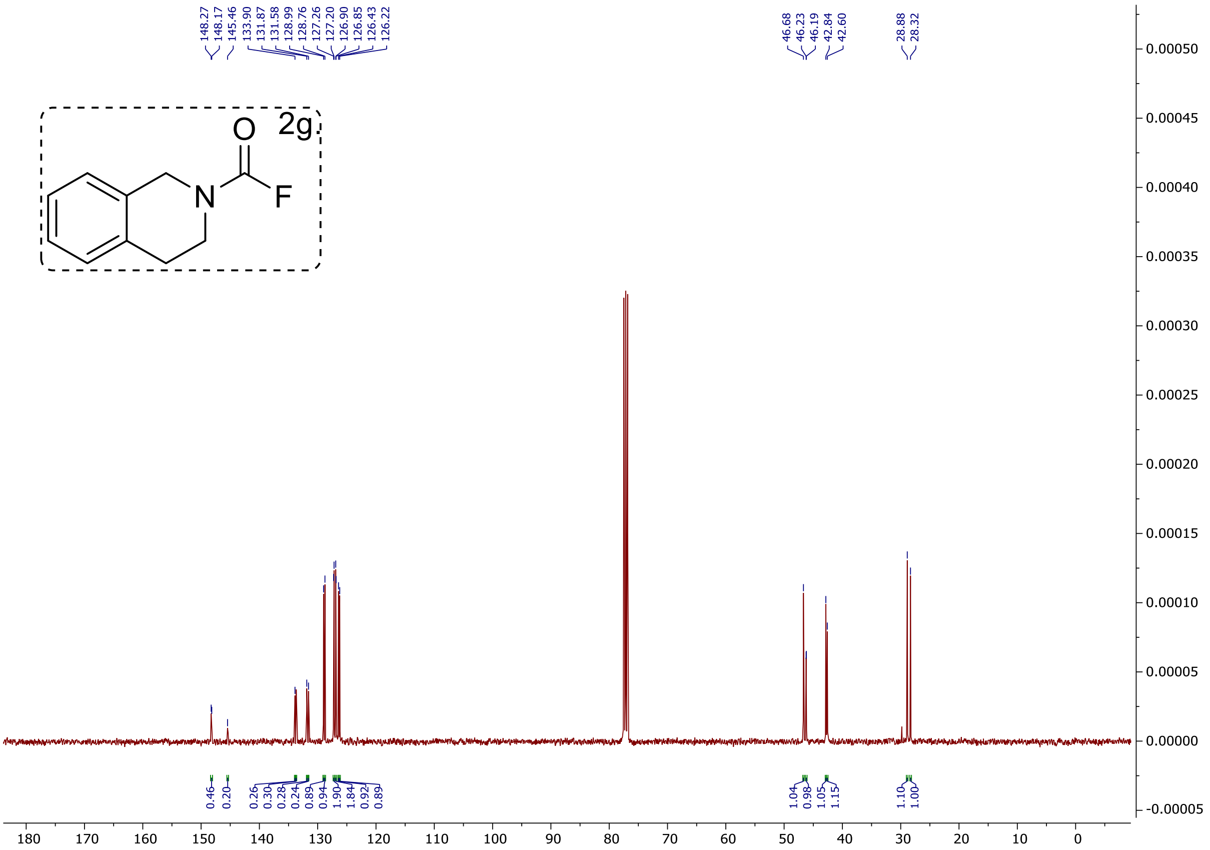

^{13}C NMR of 2g in $CDCl_3$ (101 MHz)

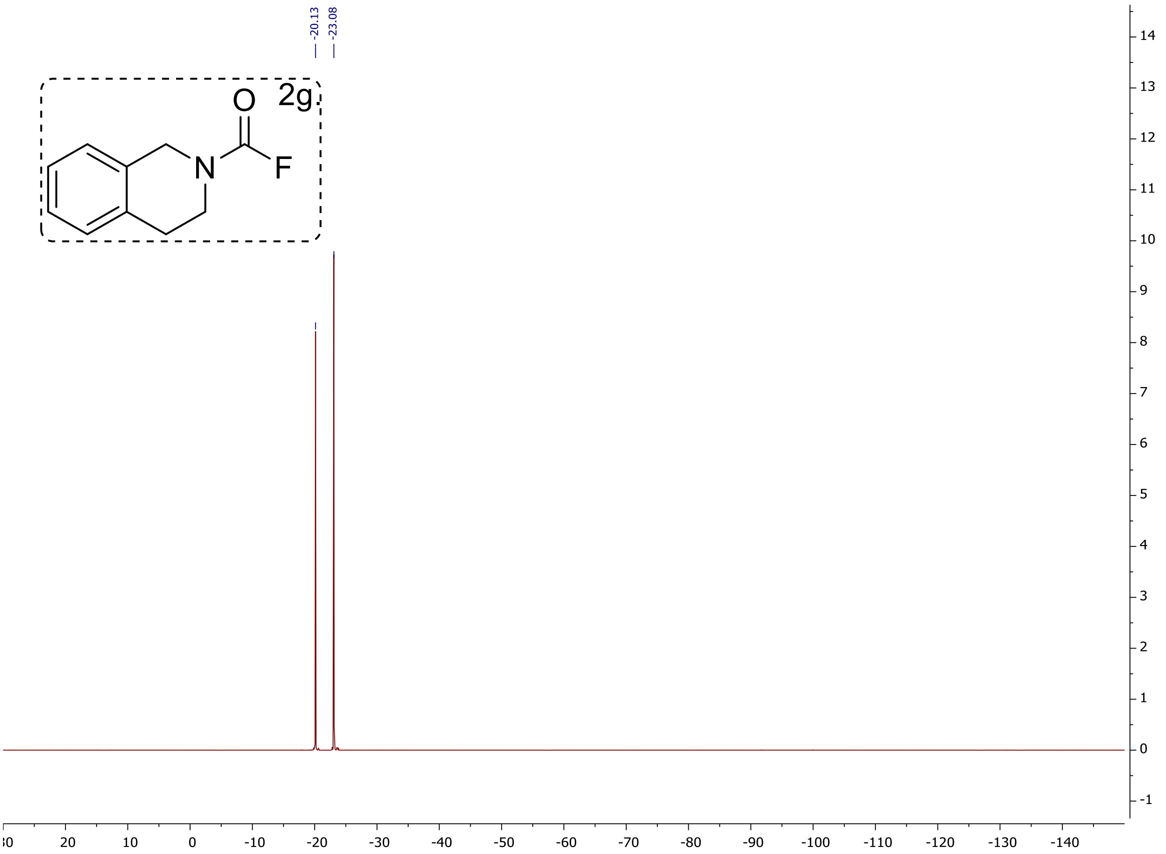

^{19}F NMR of 2g in CDCl$_3$ (376 MHz)

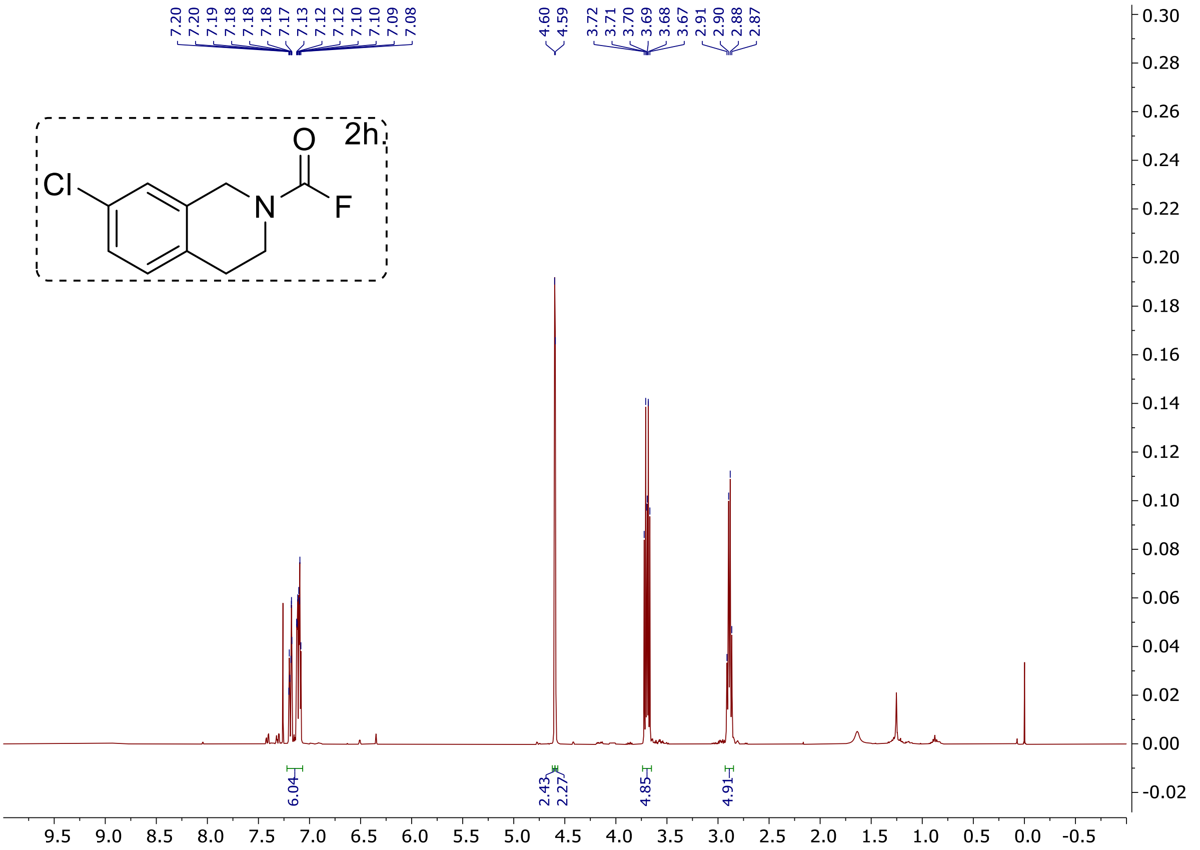

^{1}H NMR of 2h in CDCl$_3$ (400 MHz)

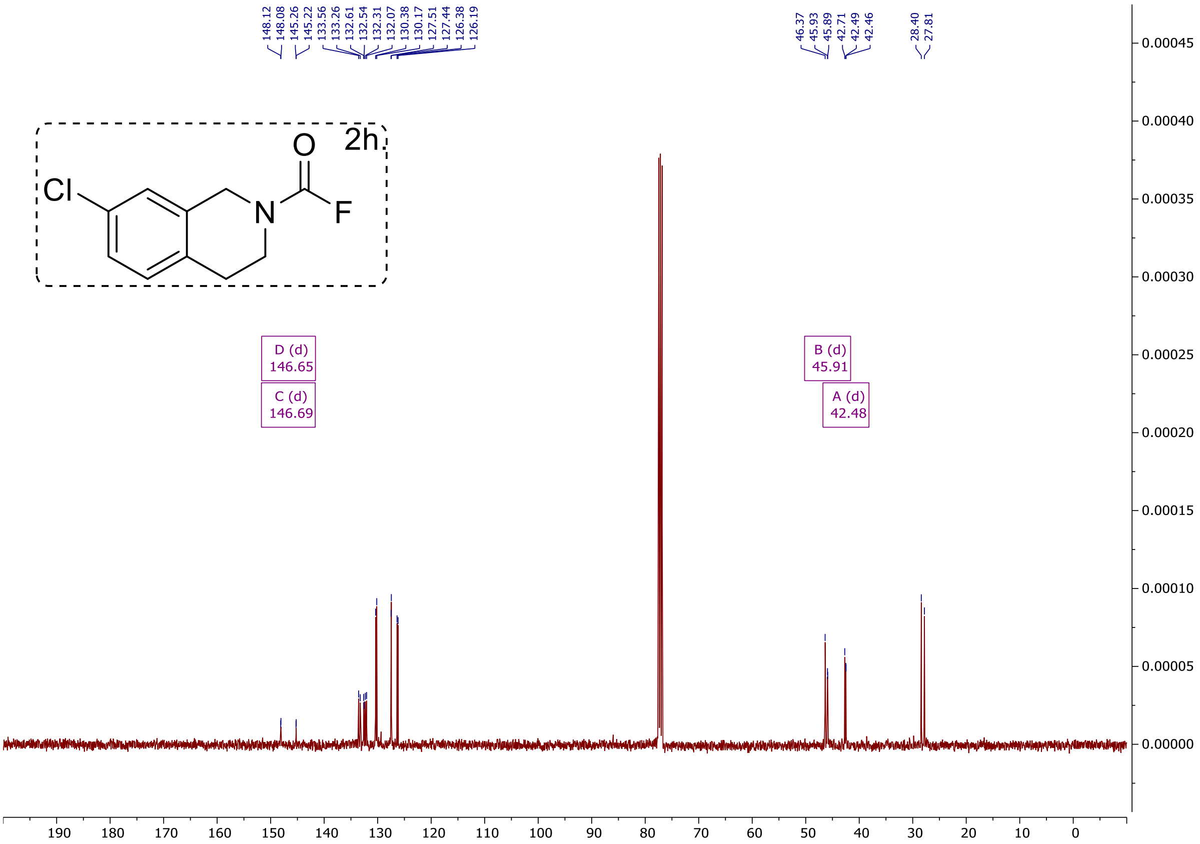

^{13}C NMR of 2h in $CDCl_3$ (101 MHz)

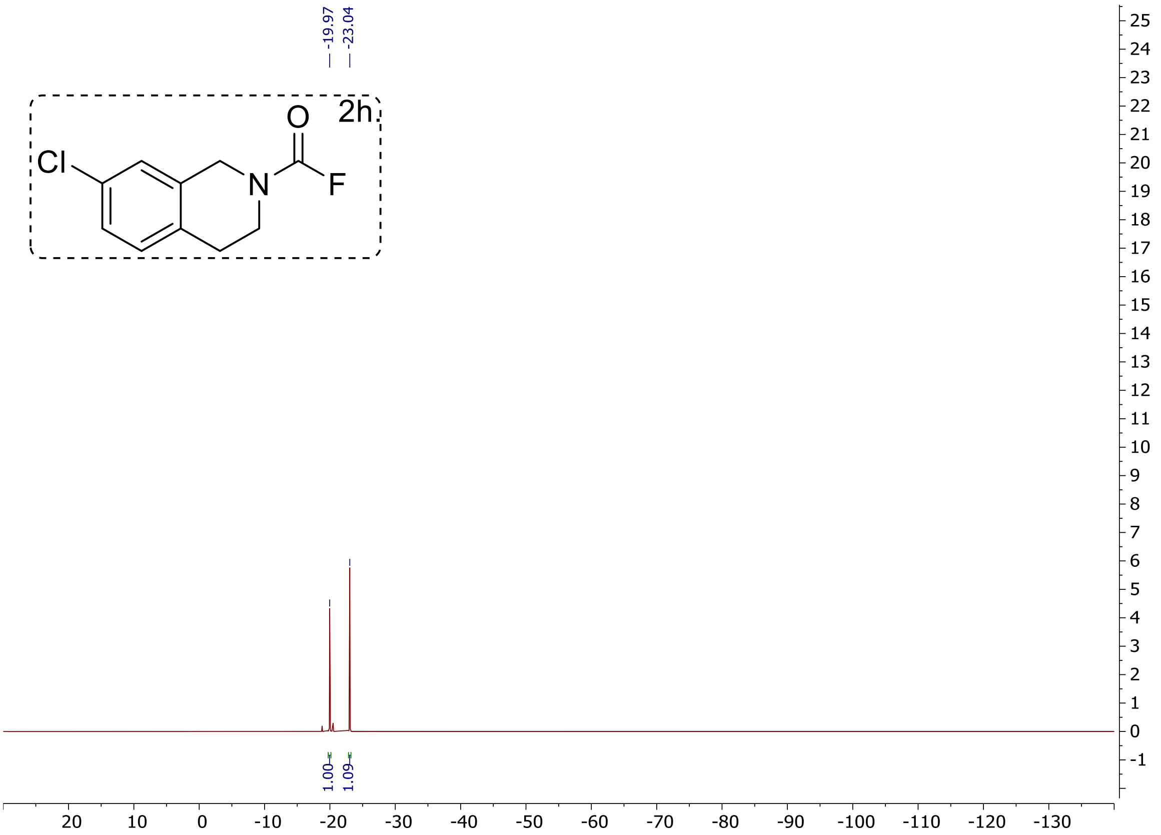

^{19}F NMR of 2h in $CDCl_3$ (376 MHz)

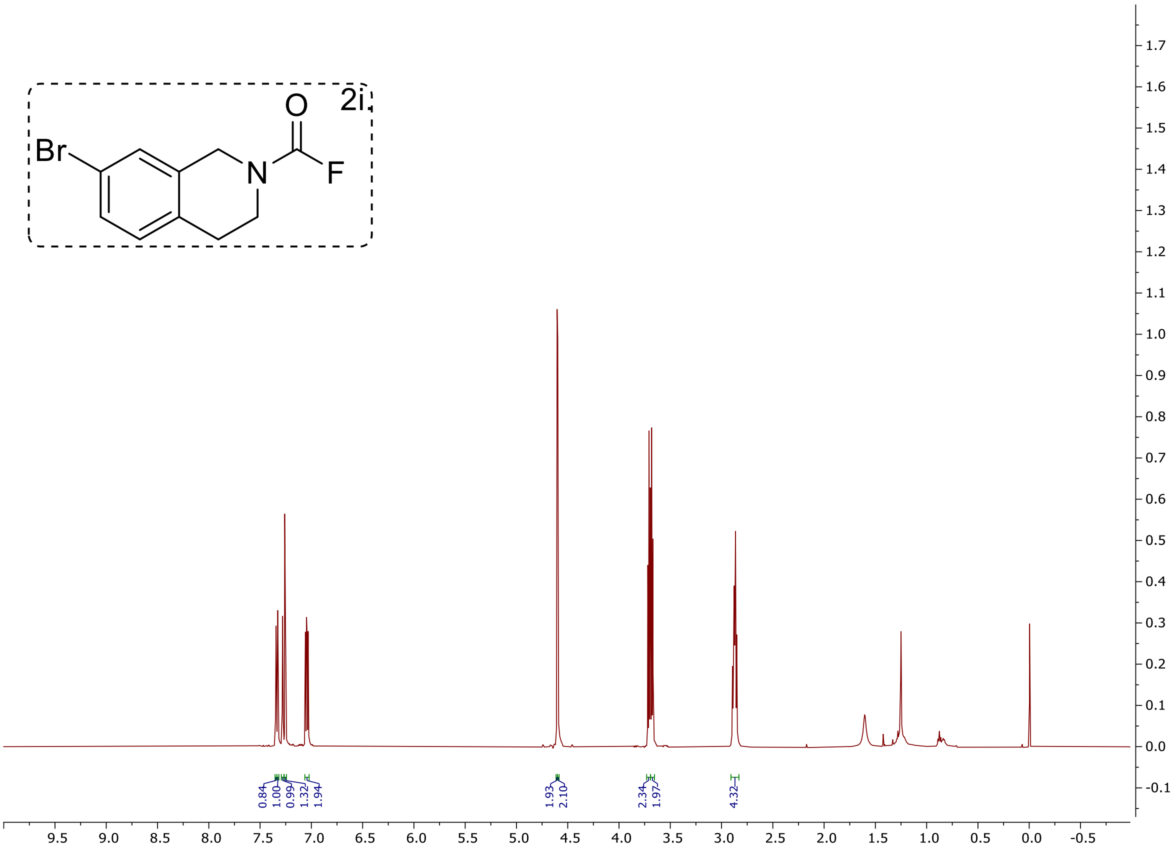

^{1}H NMR of 2i in $CDCl_3$ (500 MHz)

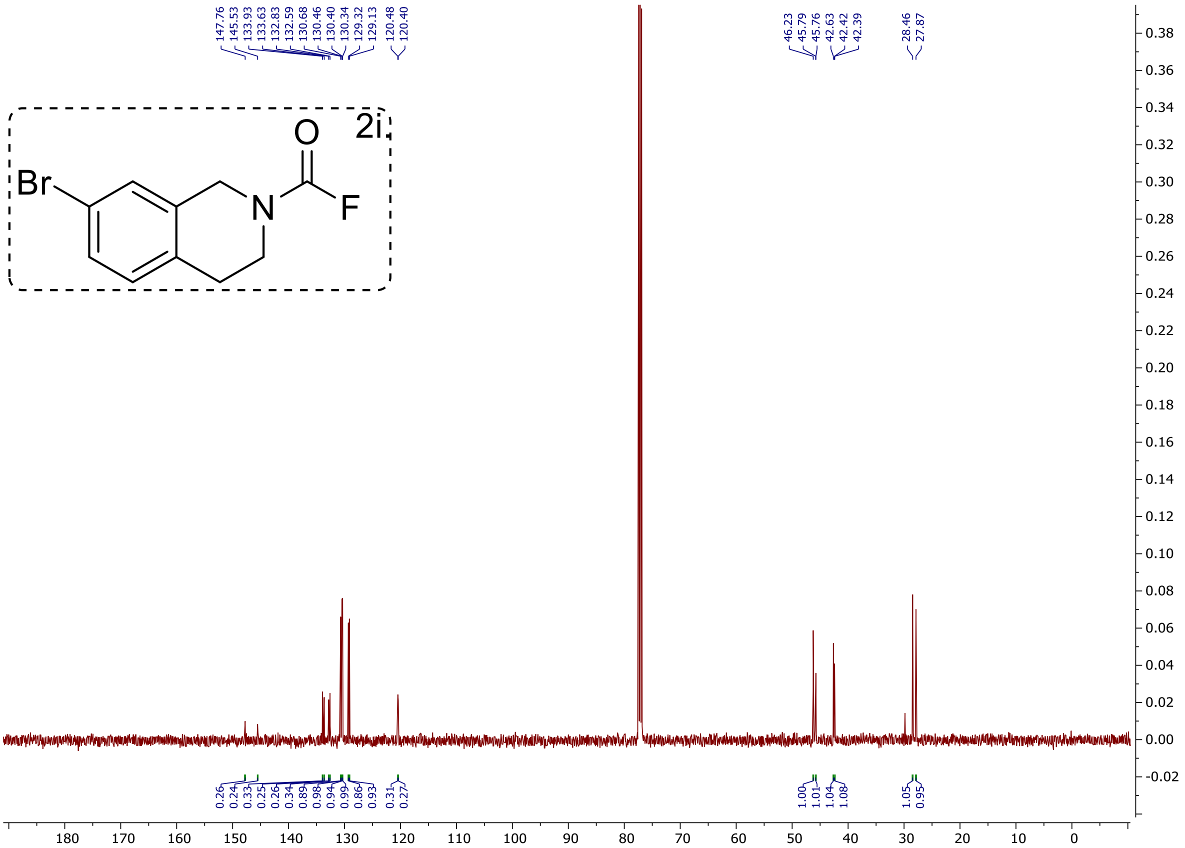

^{13}C NMR of 2i in $CDCl_3$ (126 MHz)

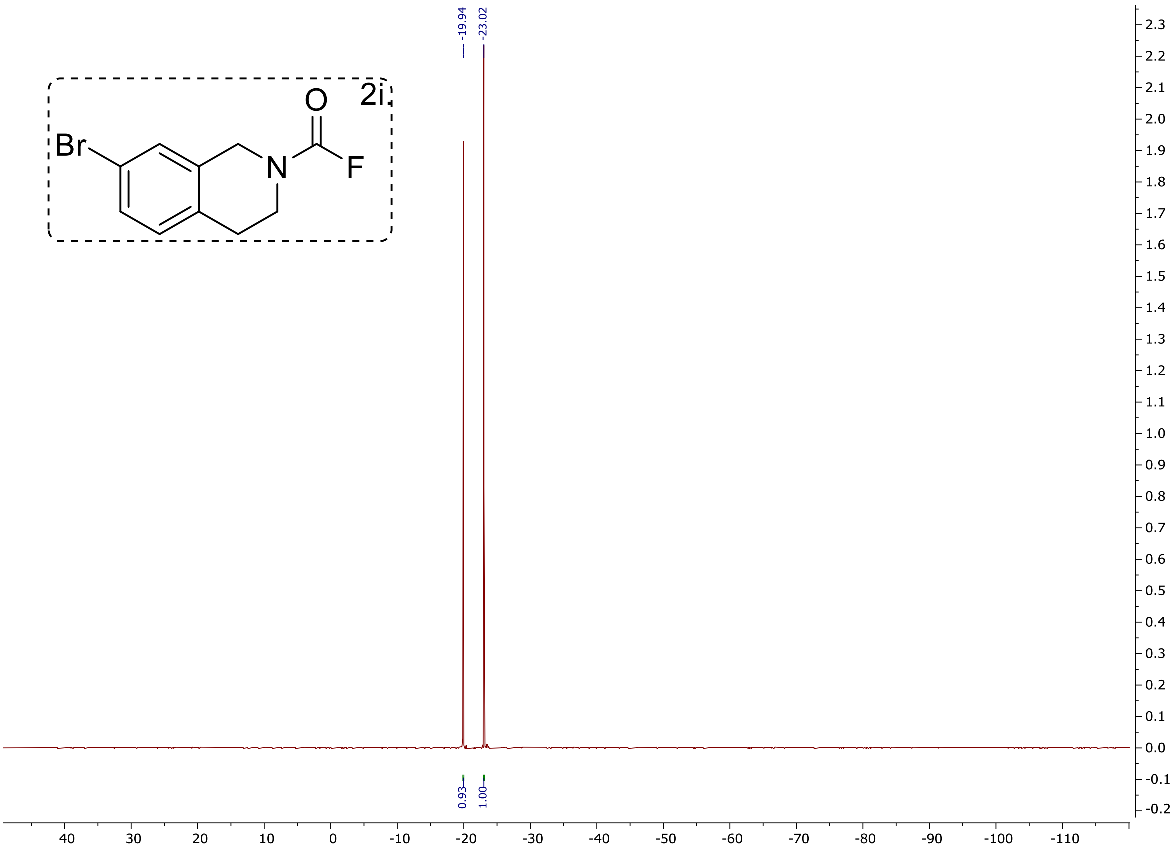

^{19}F NMR of 2i in $CDCl_3$ (471 MHz)

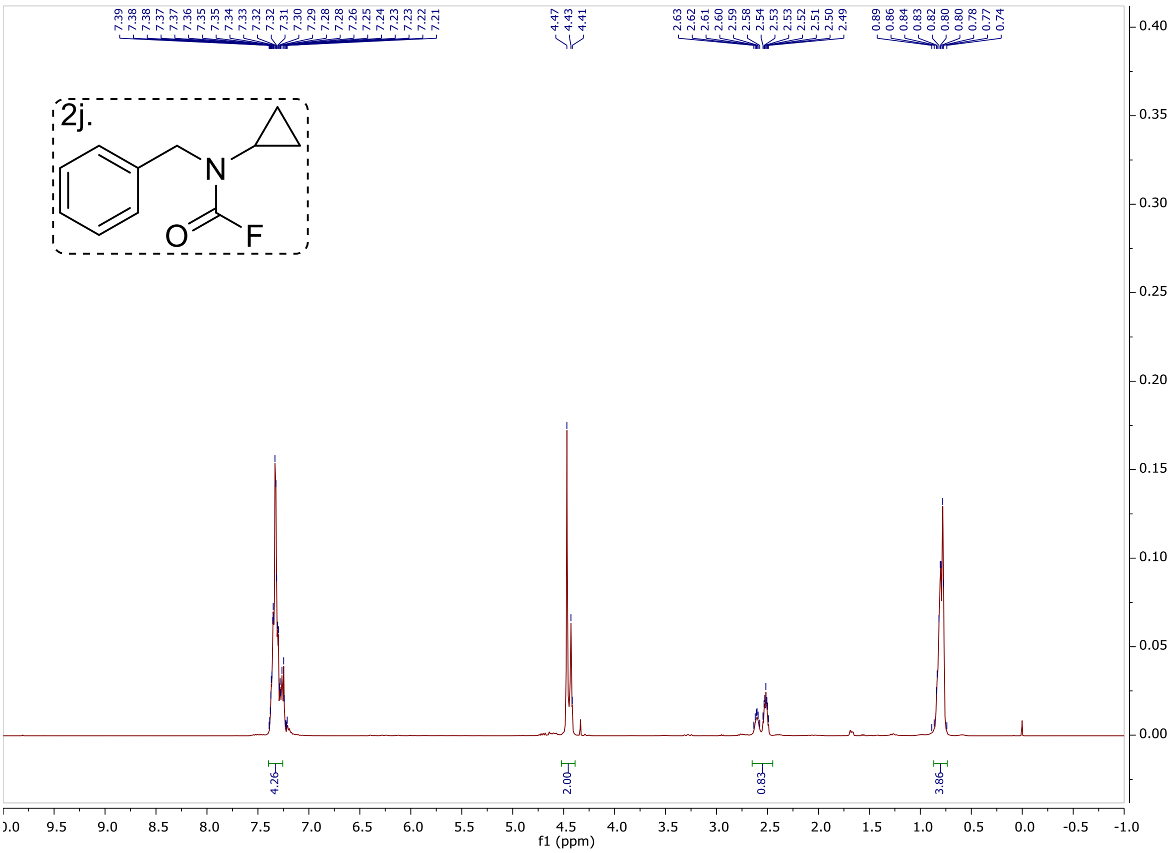

^{1}H NMR of 2j in $CDCl_3$ (400 MHz)

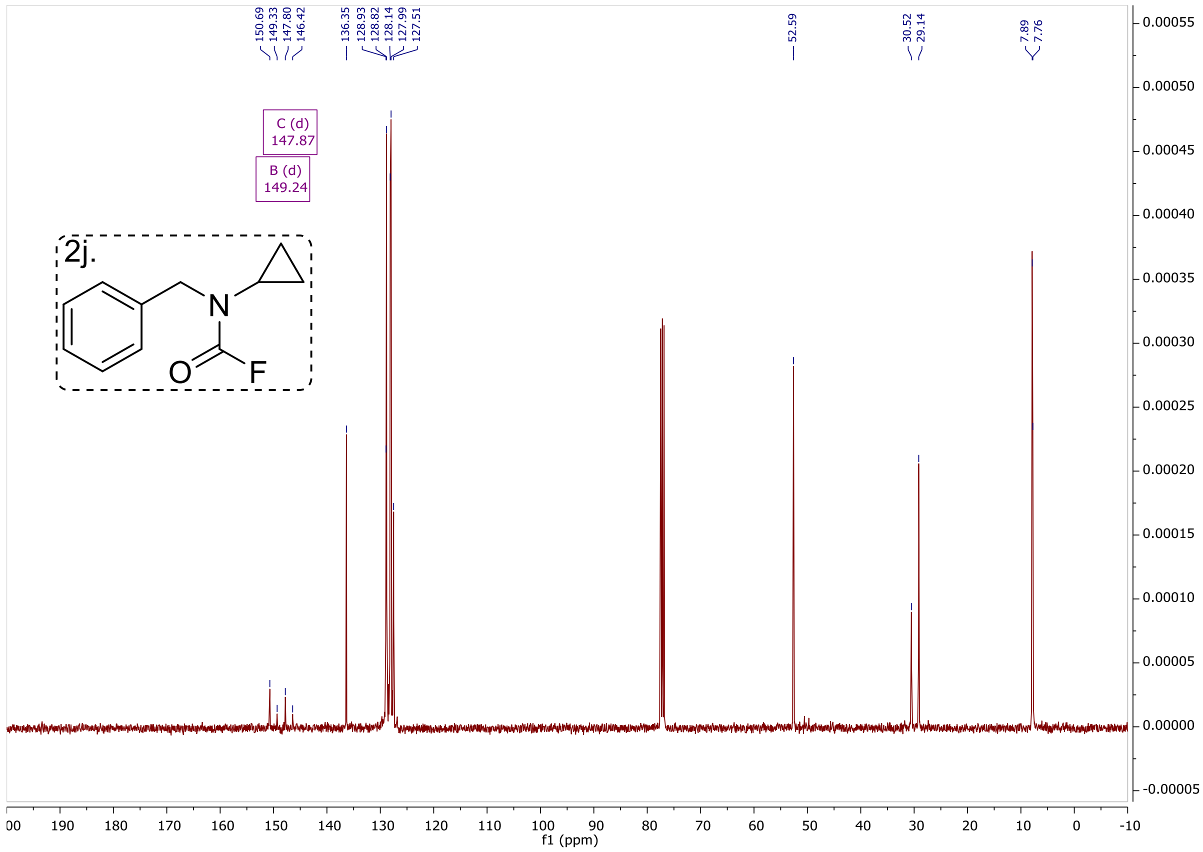

^{13}C NMR of 2j in $CDCl_3$ (101 MHz)

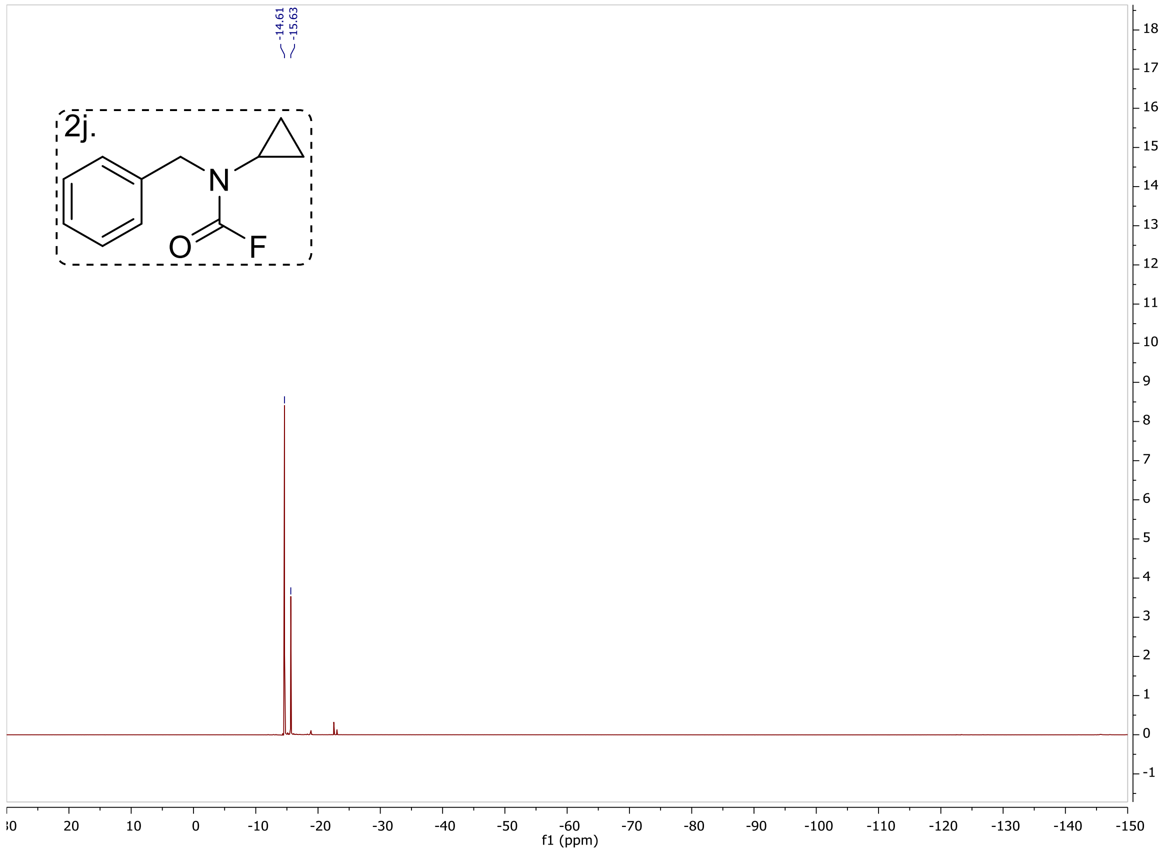

^{19}F NMR of 2j in $CDCl_3$ (376 MHz)

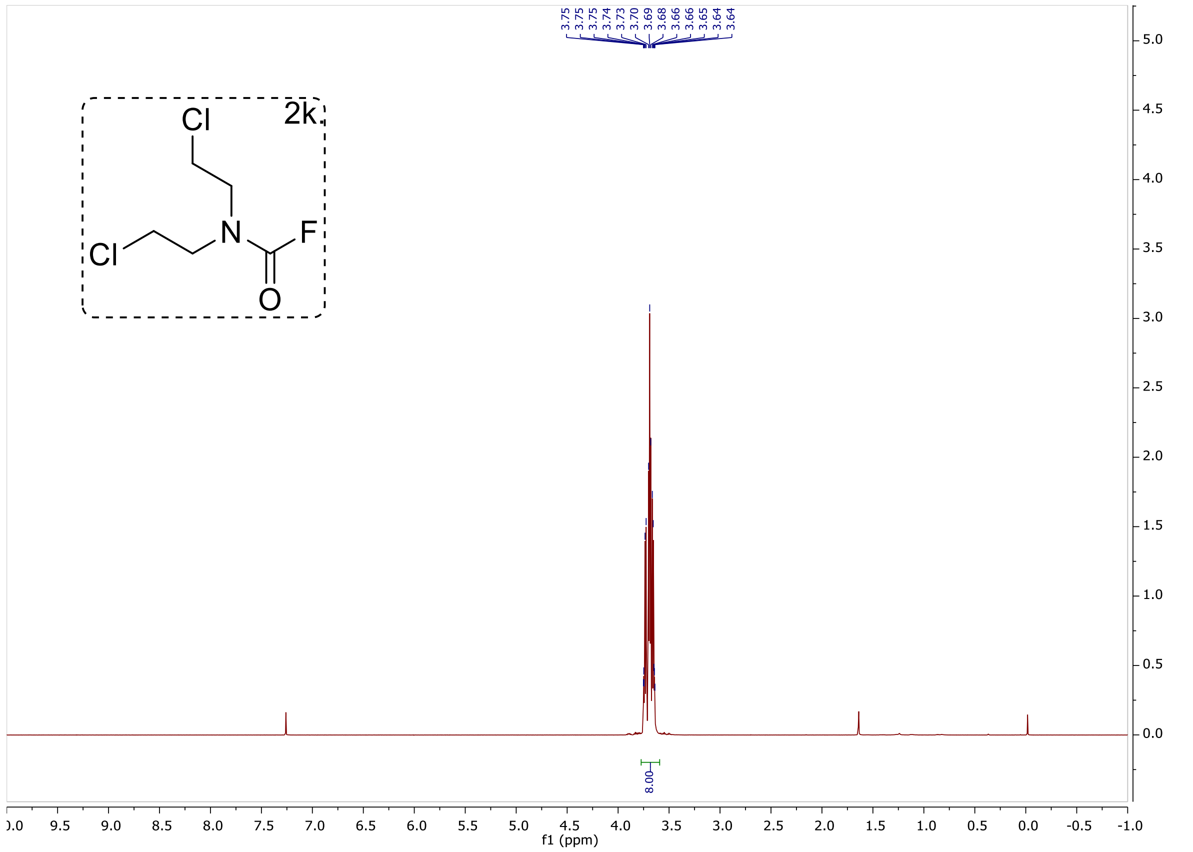

^{1}H NMR of 2k in $CDCl_3$ (500 MHz)

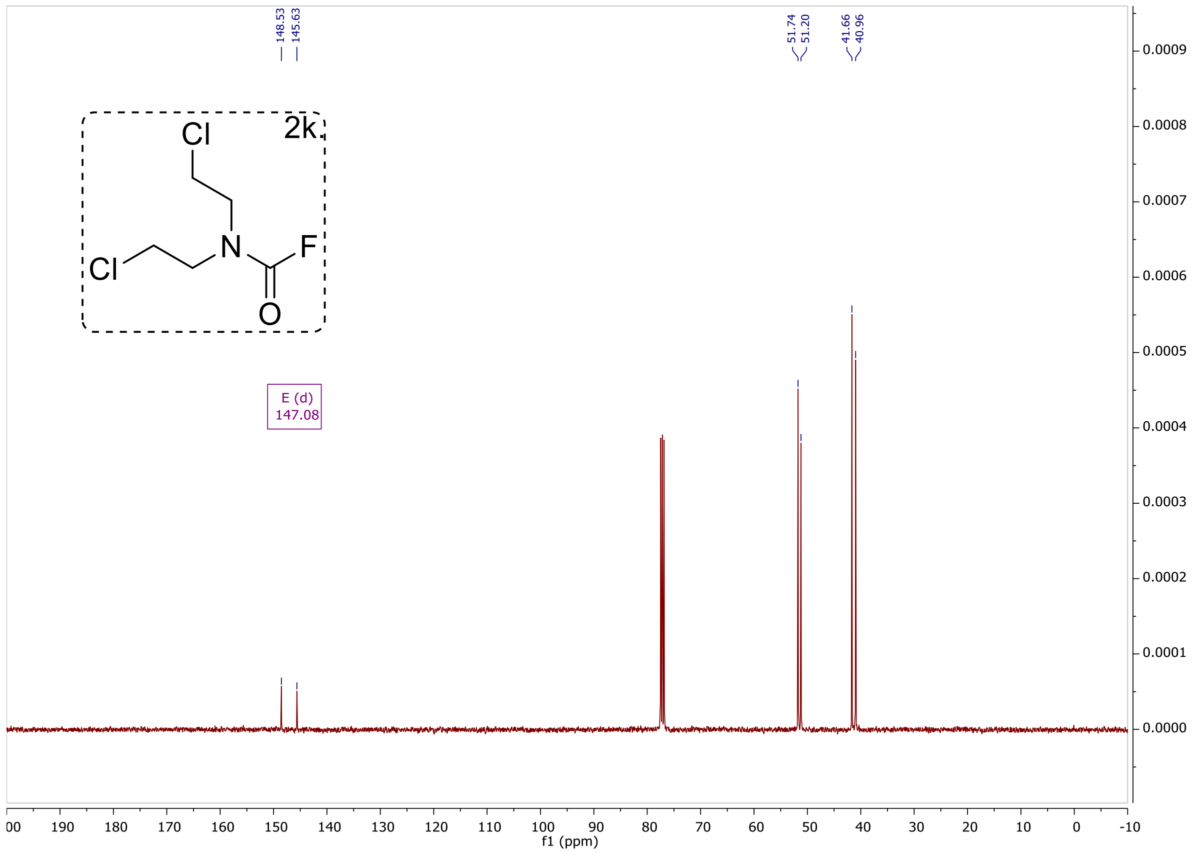

^{13}C NMR of 2k in $CDCl_3$ (101 MHz)

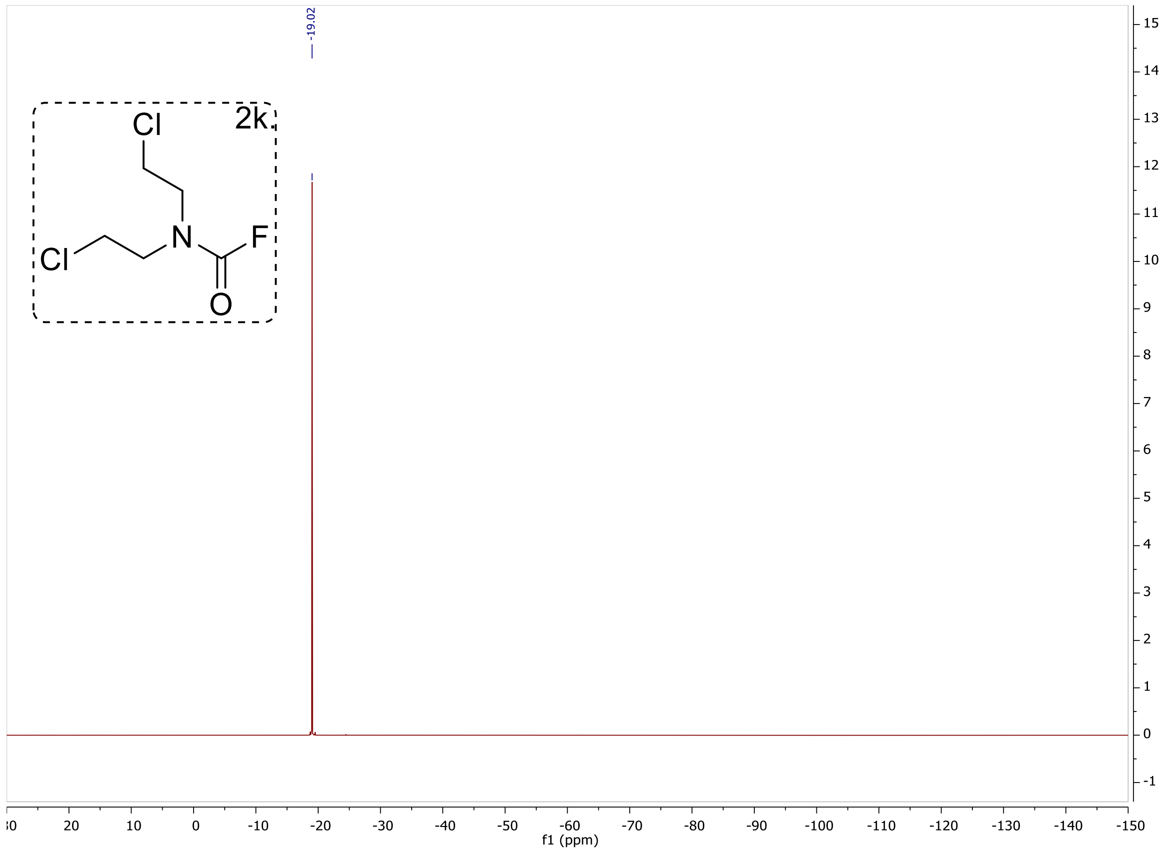

^{19}F NMR of 2k in $CDCl_3$ (376 MHz)

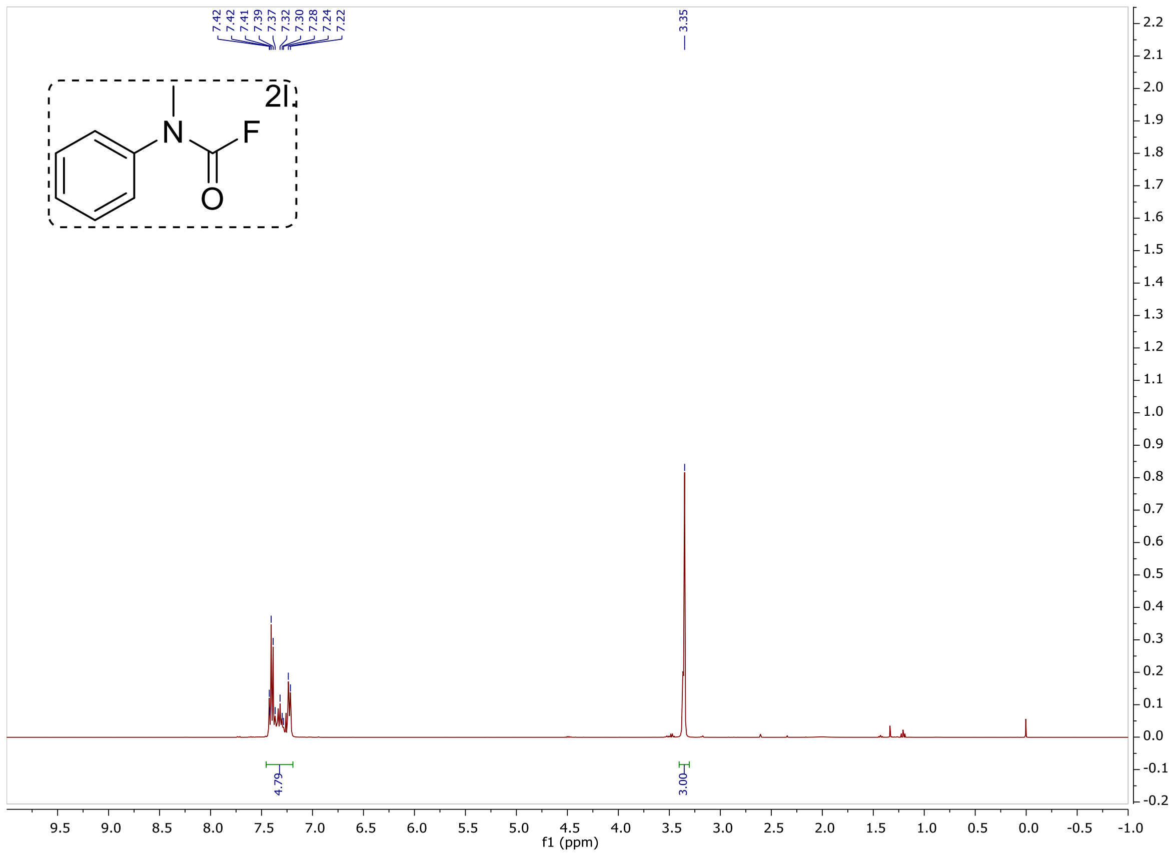

^{1}H NMR of 2l in $CDCl_3$ (400 MHz)

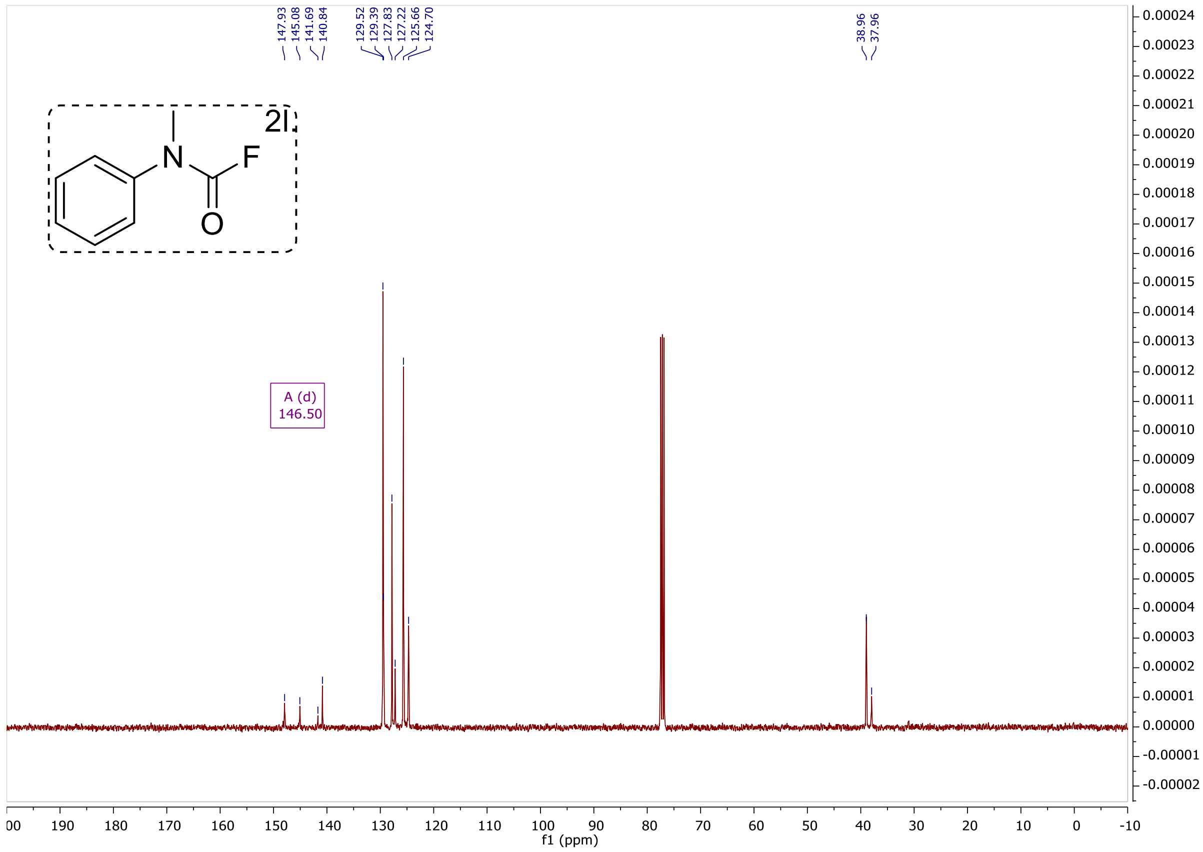

^{13}C NMR of 2l in $CDCl_3$ (101 MHz)

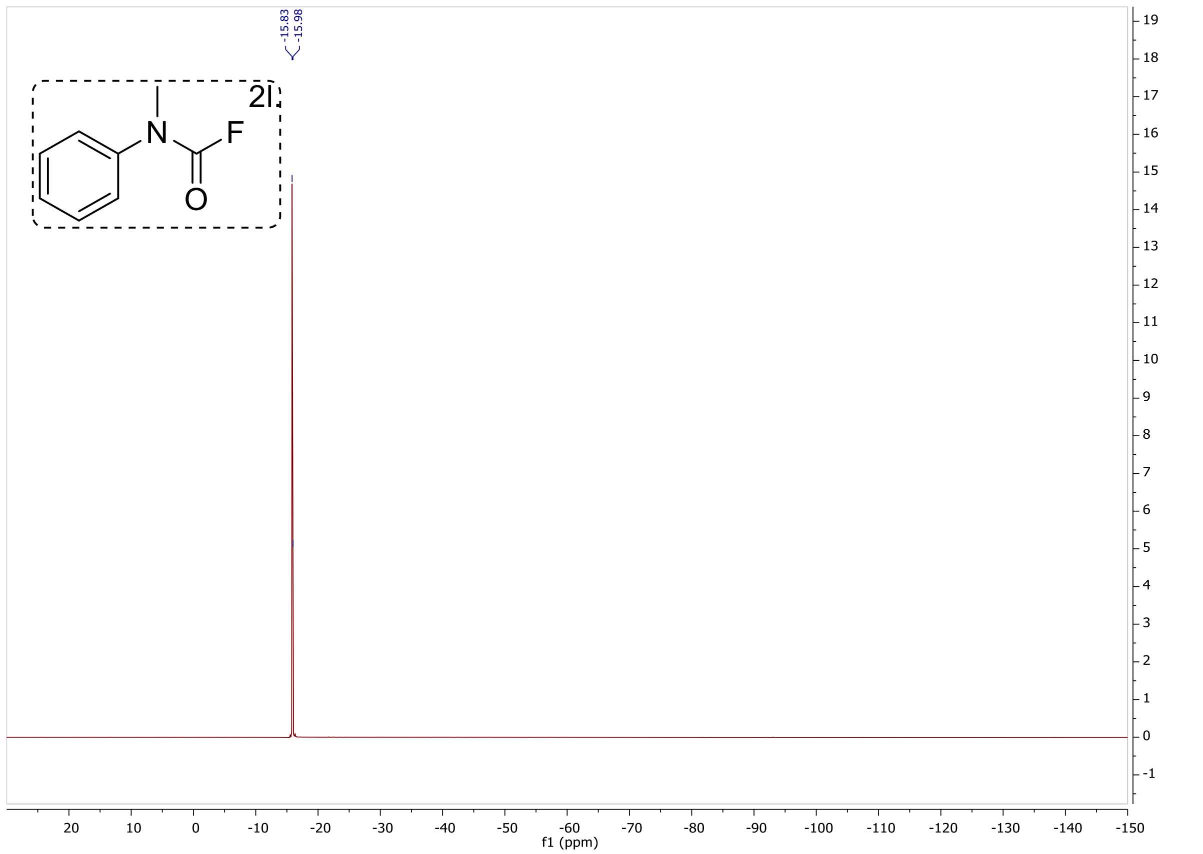

^{19}F NMR of 2l in $CDCl_3$ (376 MHz)

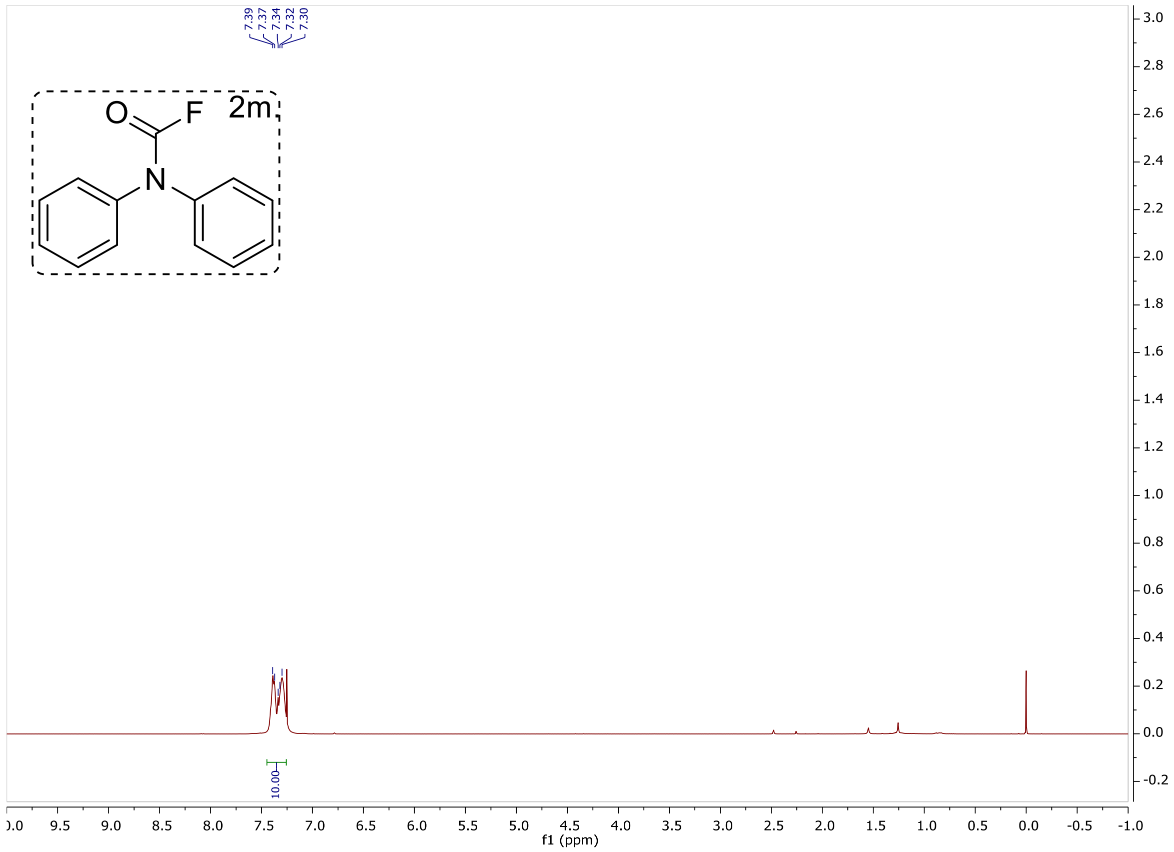

^{1}H NMR of 2m in $CDCl_3$ (400 MHz)

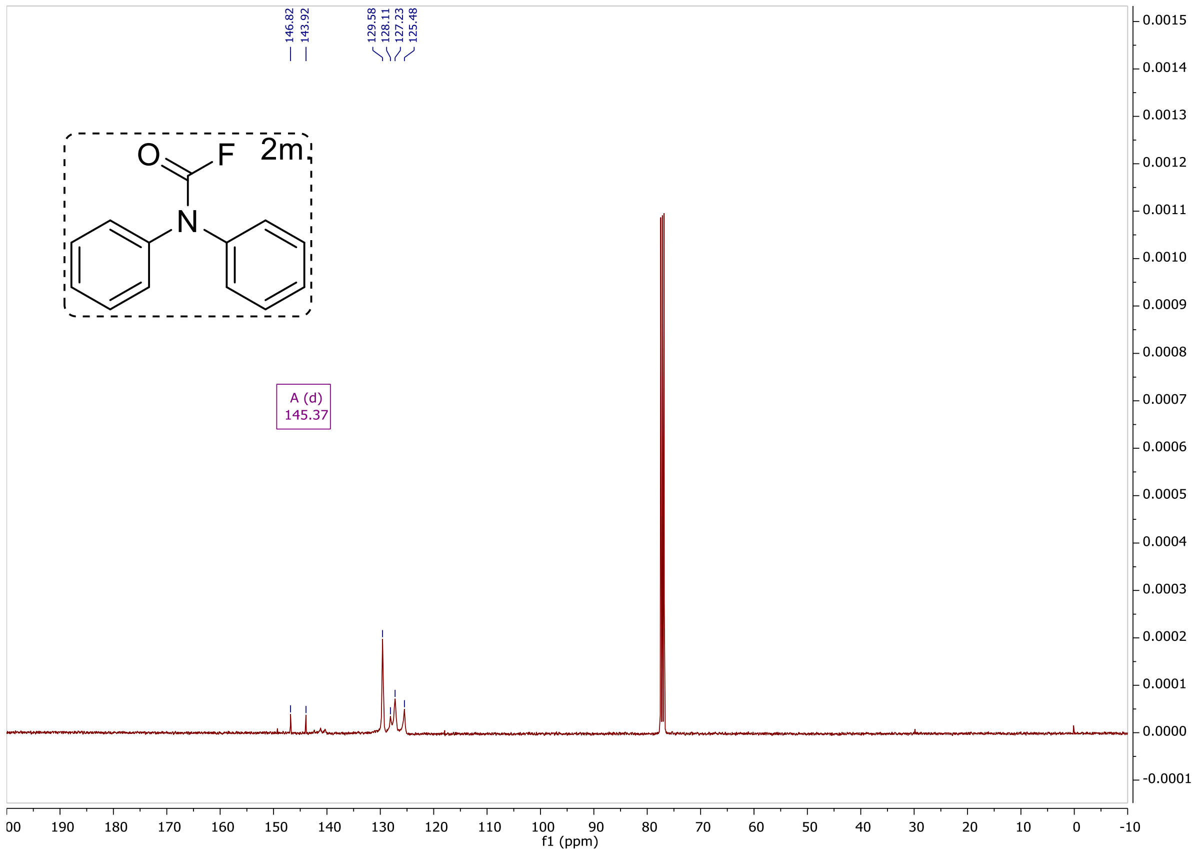

^{13}C NMR of 2m in $CDCl_3$ (101 MHz)

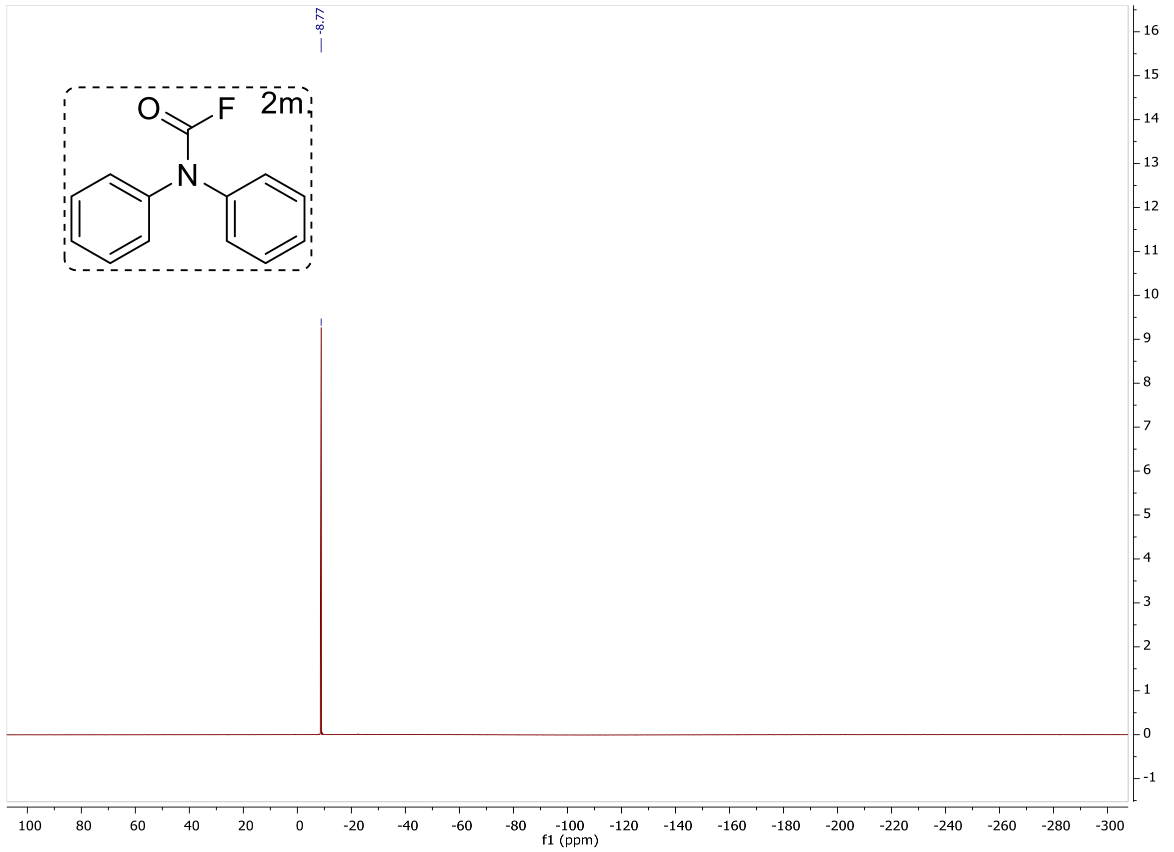

^{19}F NMR of 2m in $CDCl_3$ (376 MHz)

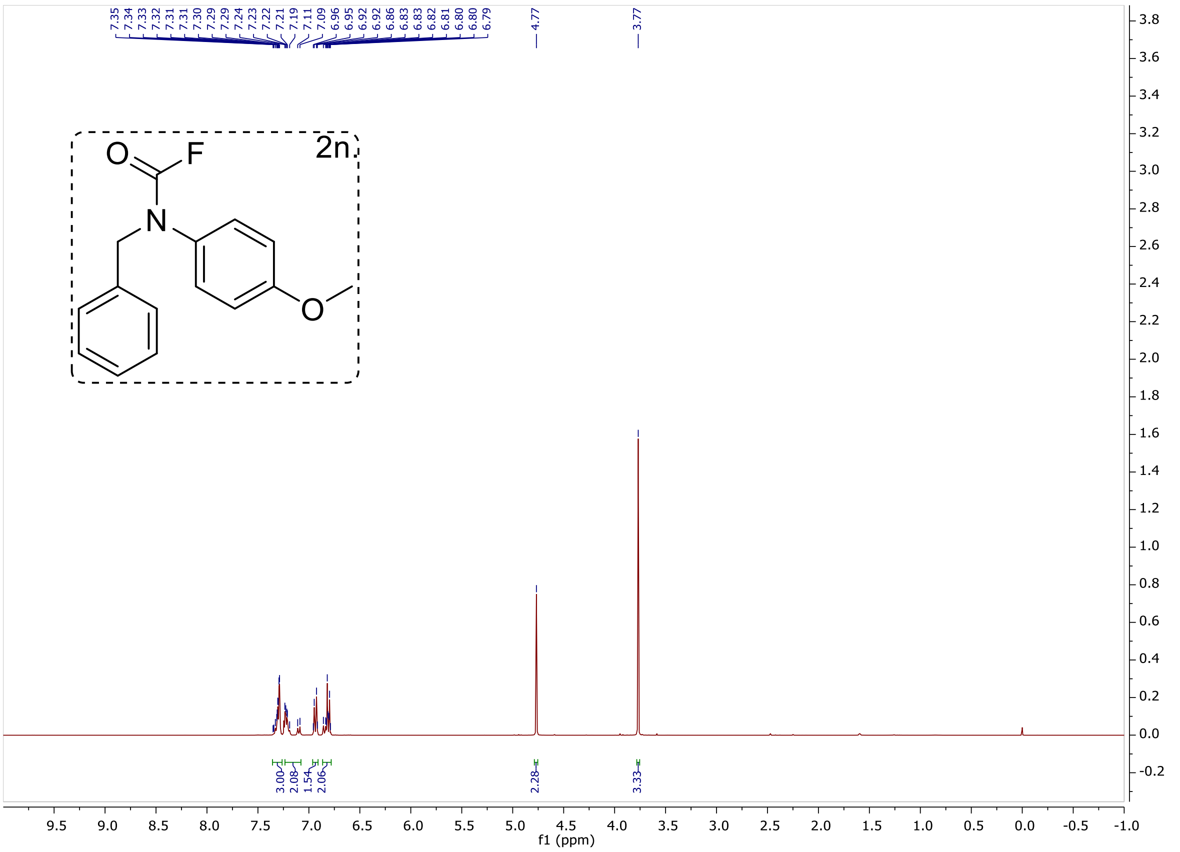

^{1}H NMR of 2n in $CDCl_3$ (400 MHz)

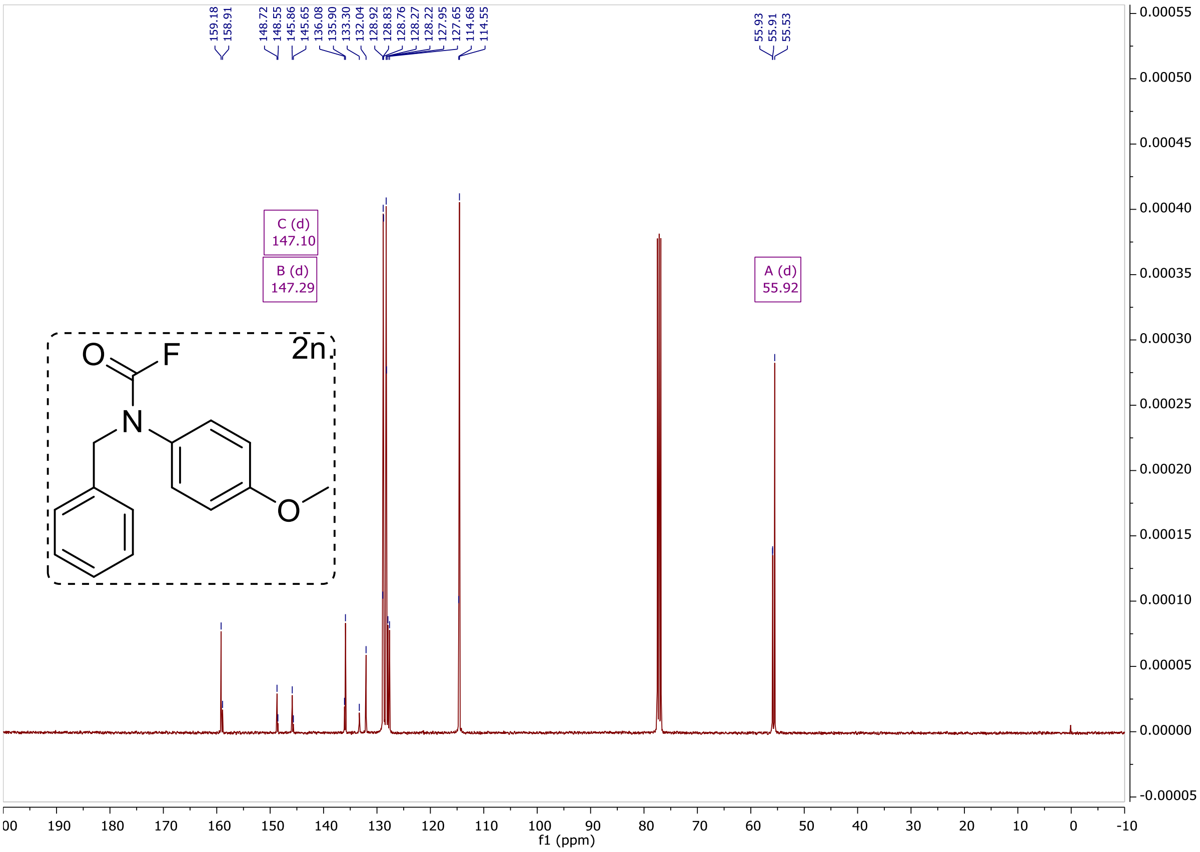

^{13}C NMR of 2n in $CDCl_3$ (101 MHz)

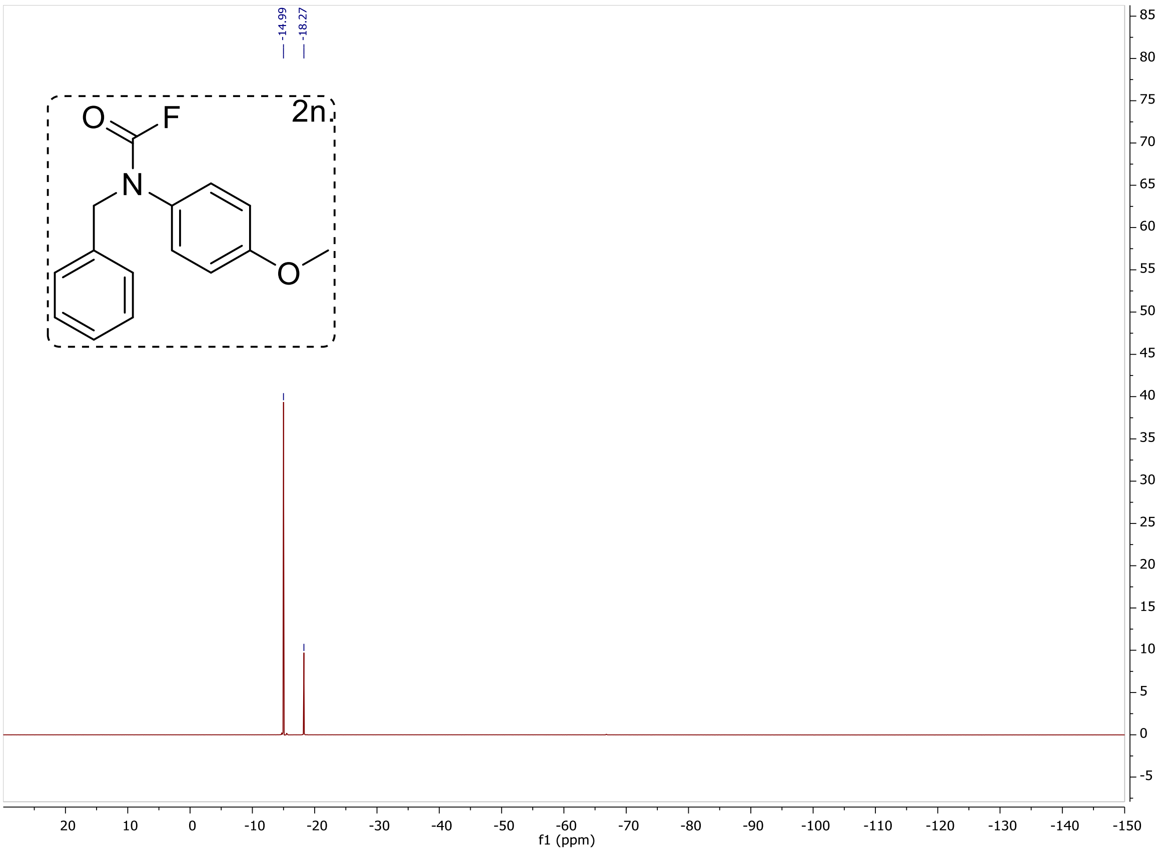

^{19}F NMR of 2n in $CDCl_3$ (376 MHz)

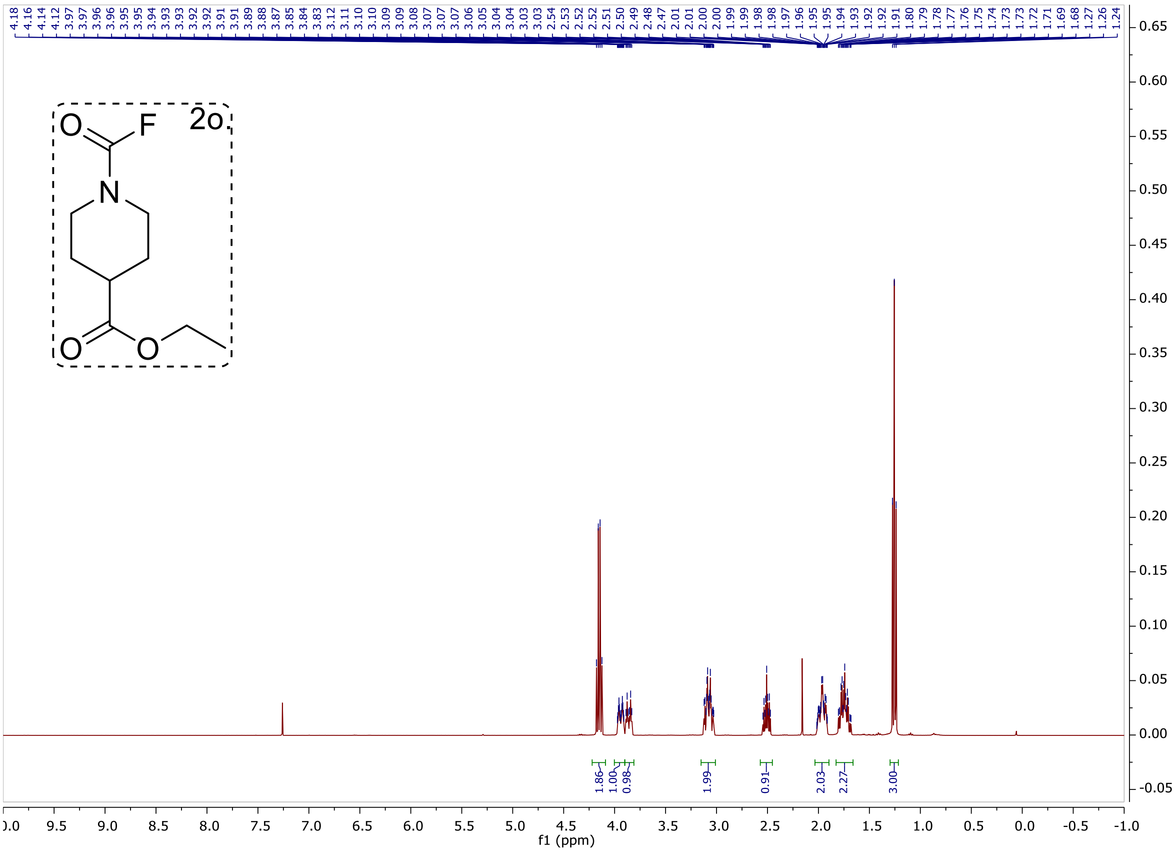

^{1}H NMR of 2o in $CDCl_3$ (400 MHz)

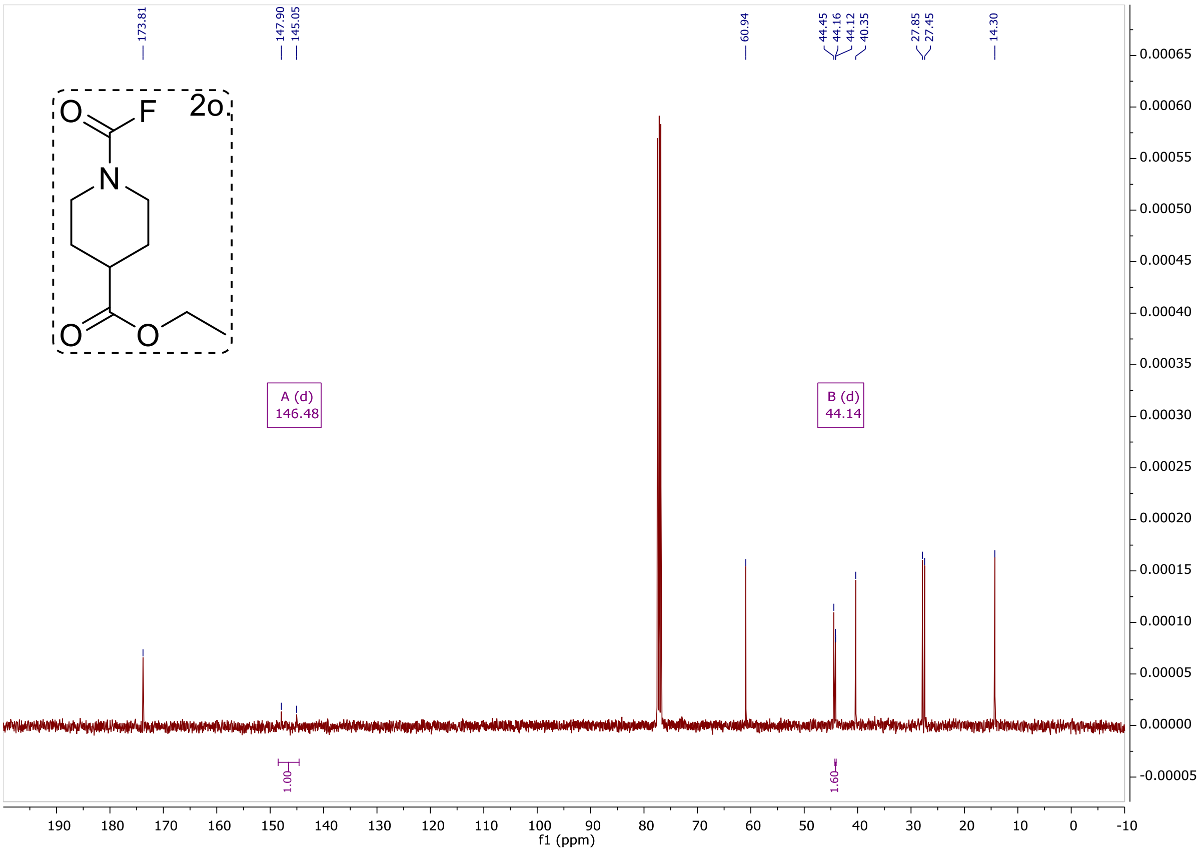

^{13}C NMR of 2o in $CDCl_3$ (101 MHz)

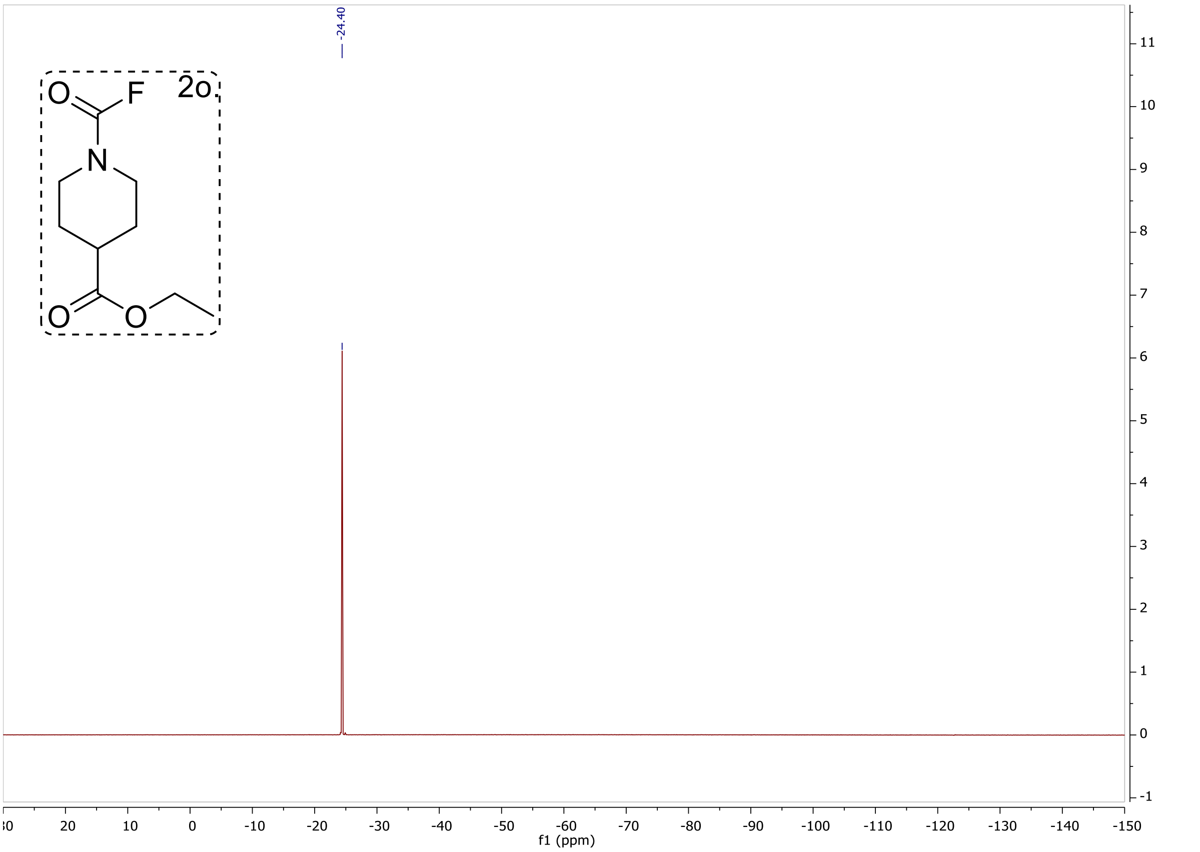

^{19}F NMR of 2o in $CDCl_3$ (376 MHz)

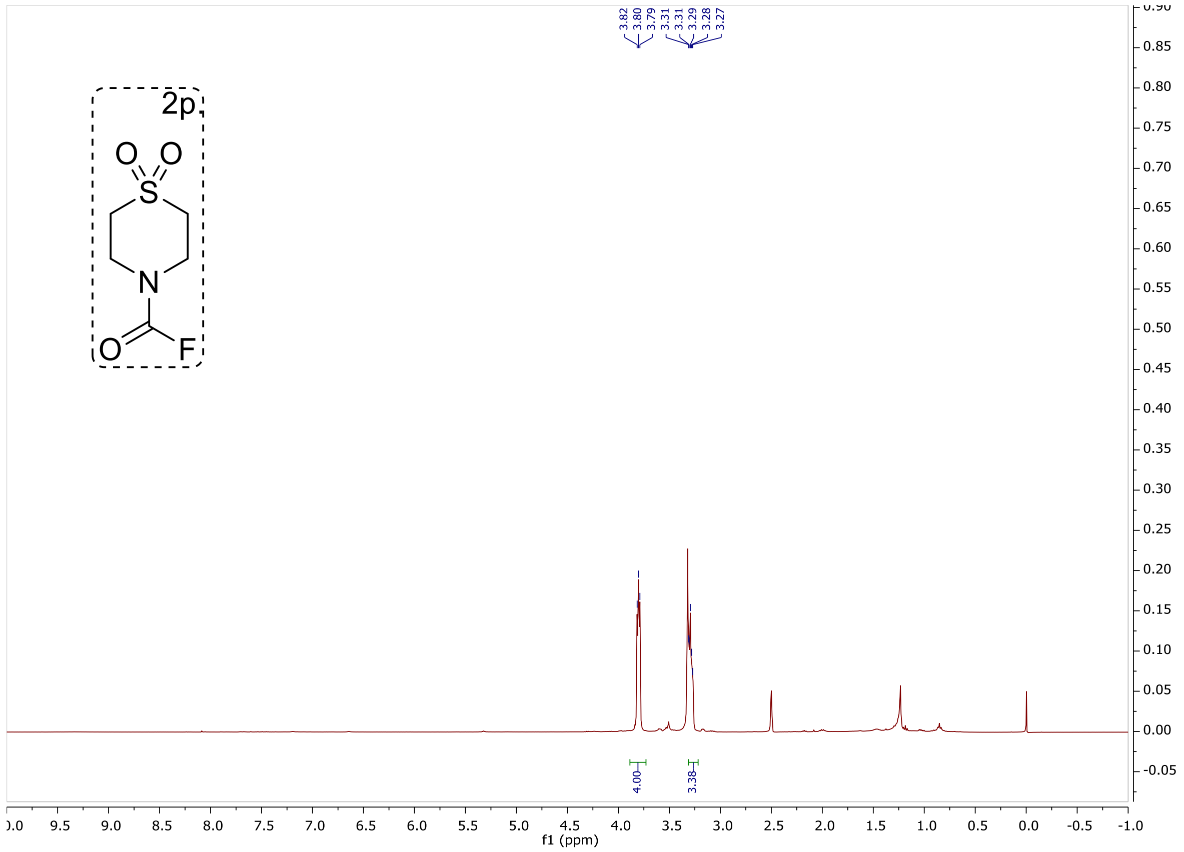

^{1}H NMR of 2p in DMSO-d_6 (400 MHz)

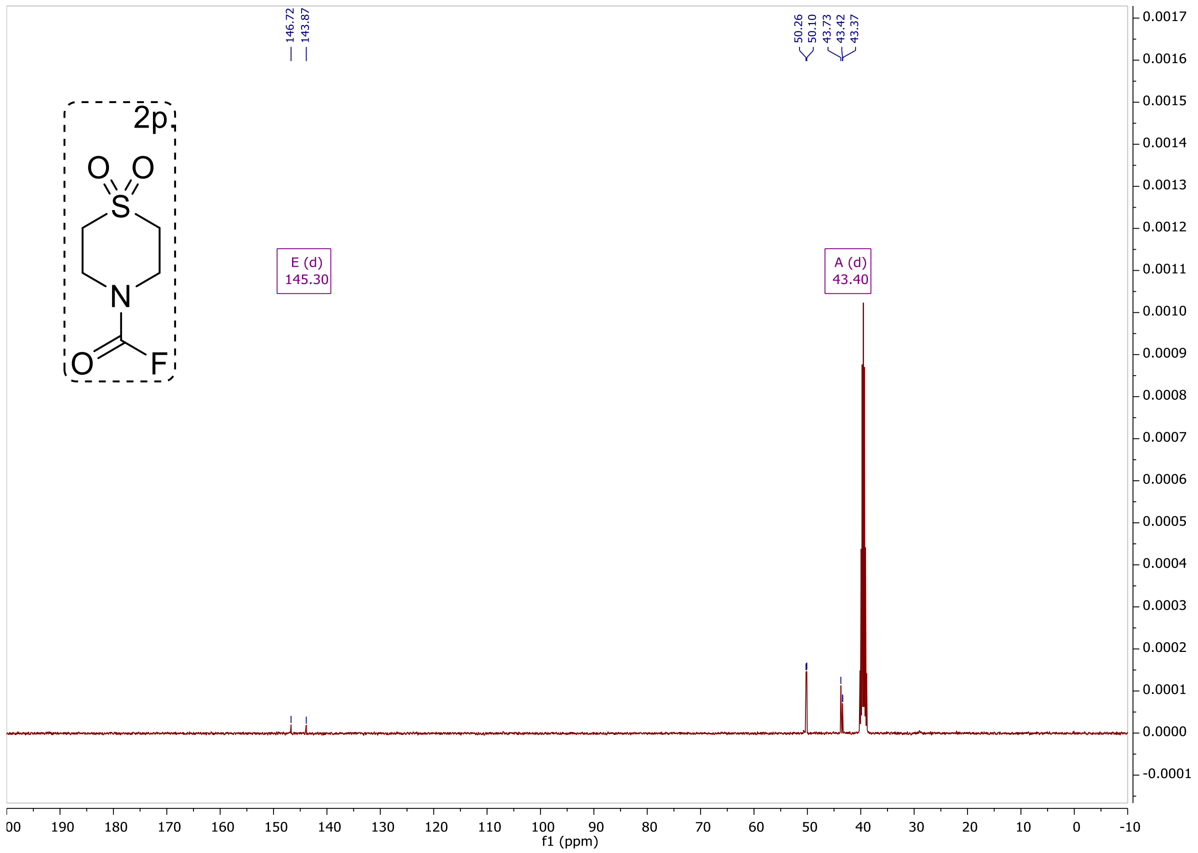

^{13}C NMR of 2p in DMSO-d_6 (101 MHz)

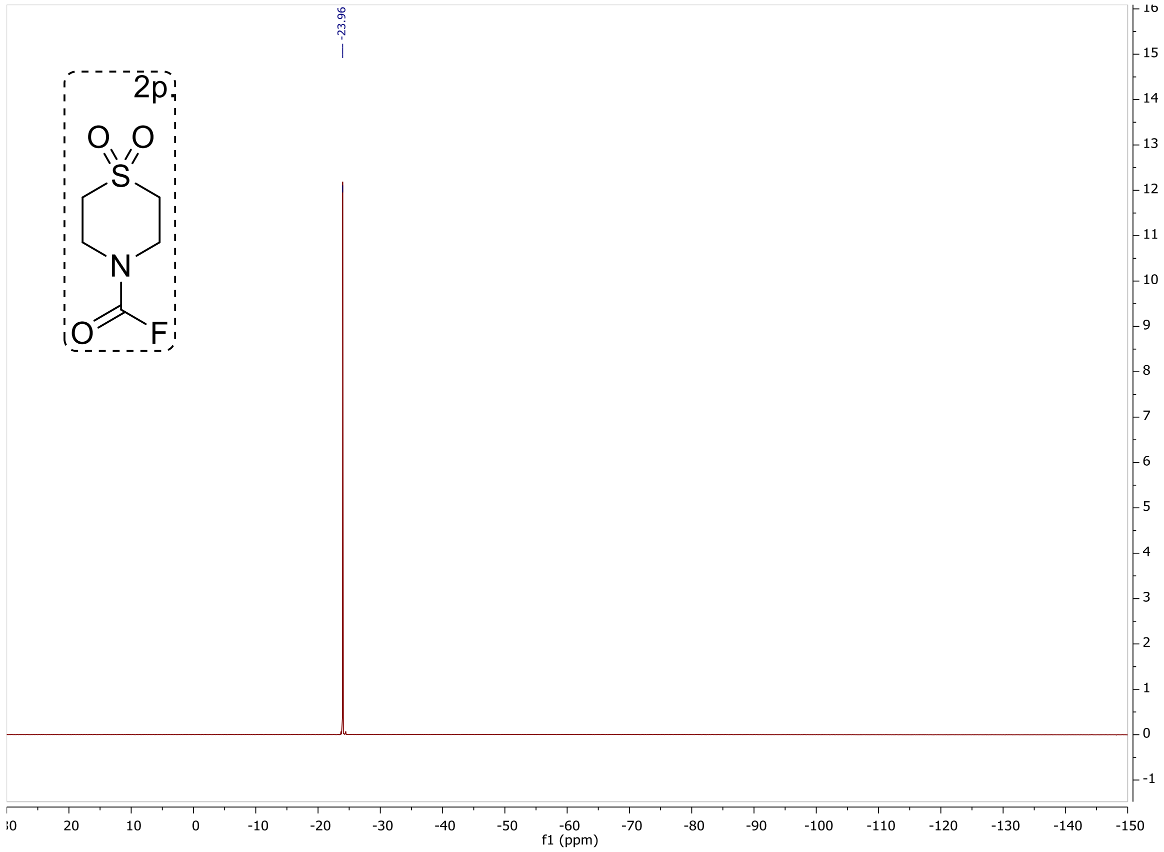

^{19}F NMR of 2p in DMSO-d_6 (376 MHz)

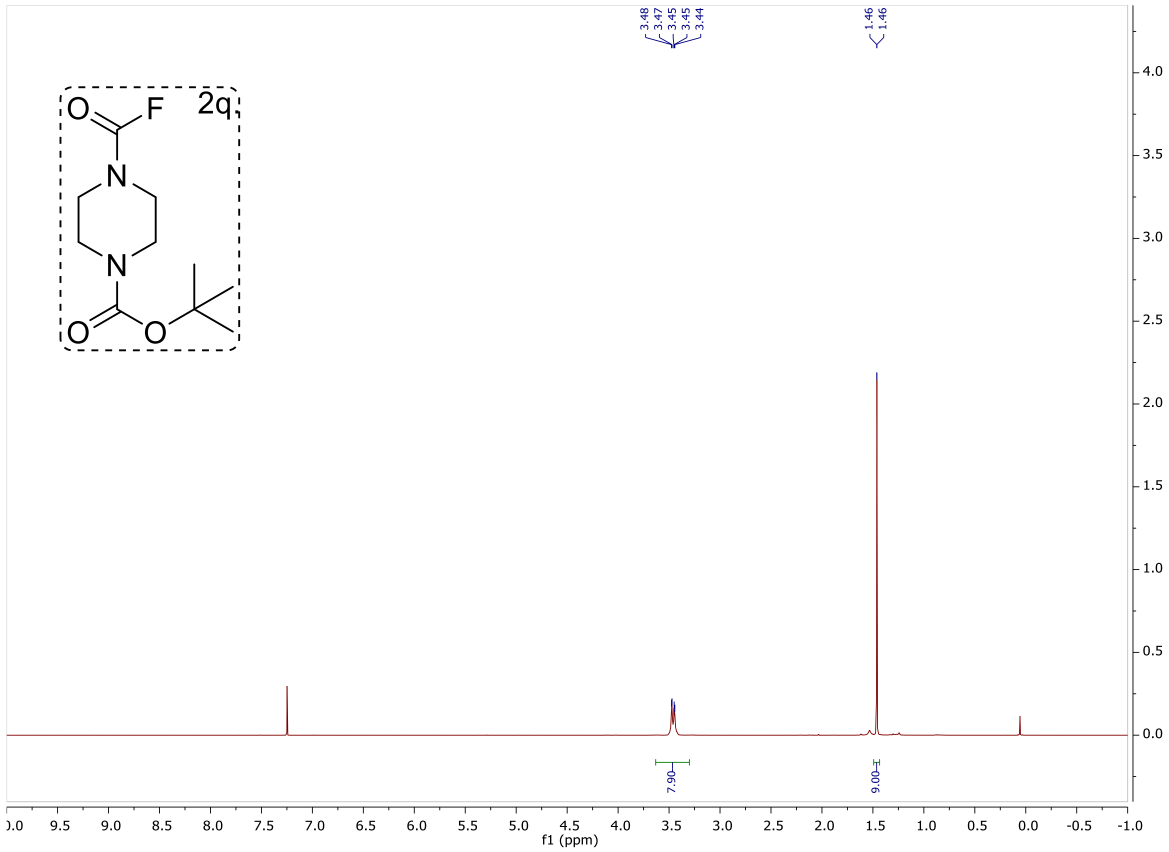

^{1}H NMR of 2q in $CDCl_3$ (400 MHz)

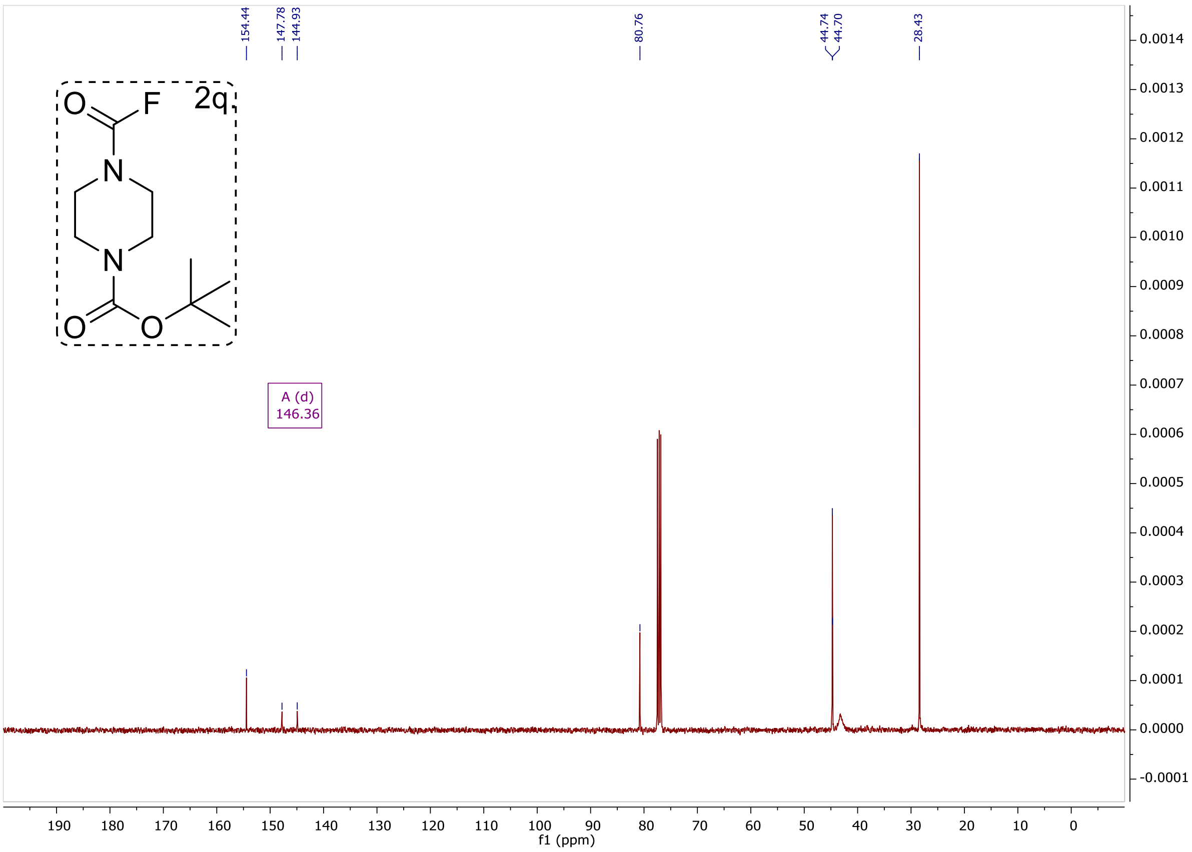

^{13}C NMR of 2q in $CDCl_3$ (101 MHz)

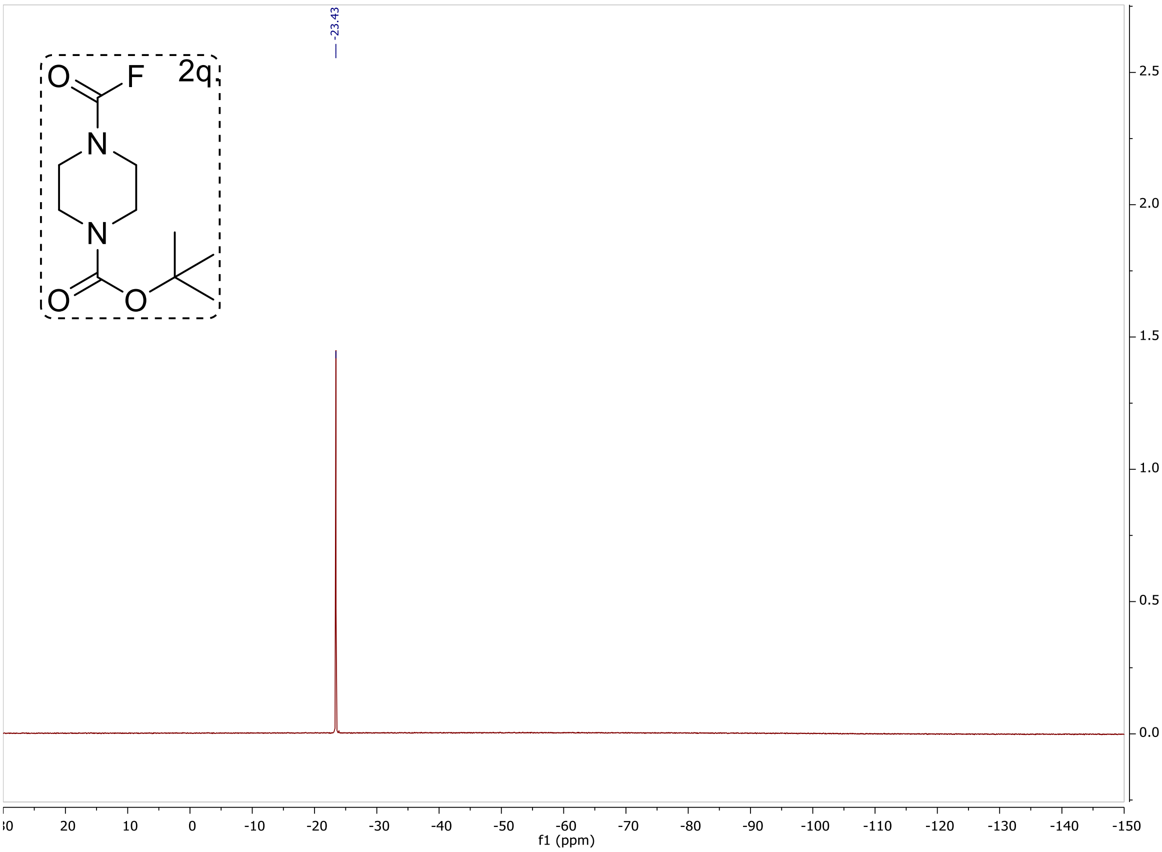

^{19}F NMR of 2q in $CDCl_3$ (376 MHz)

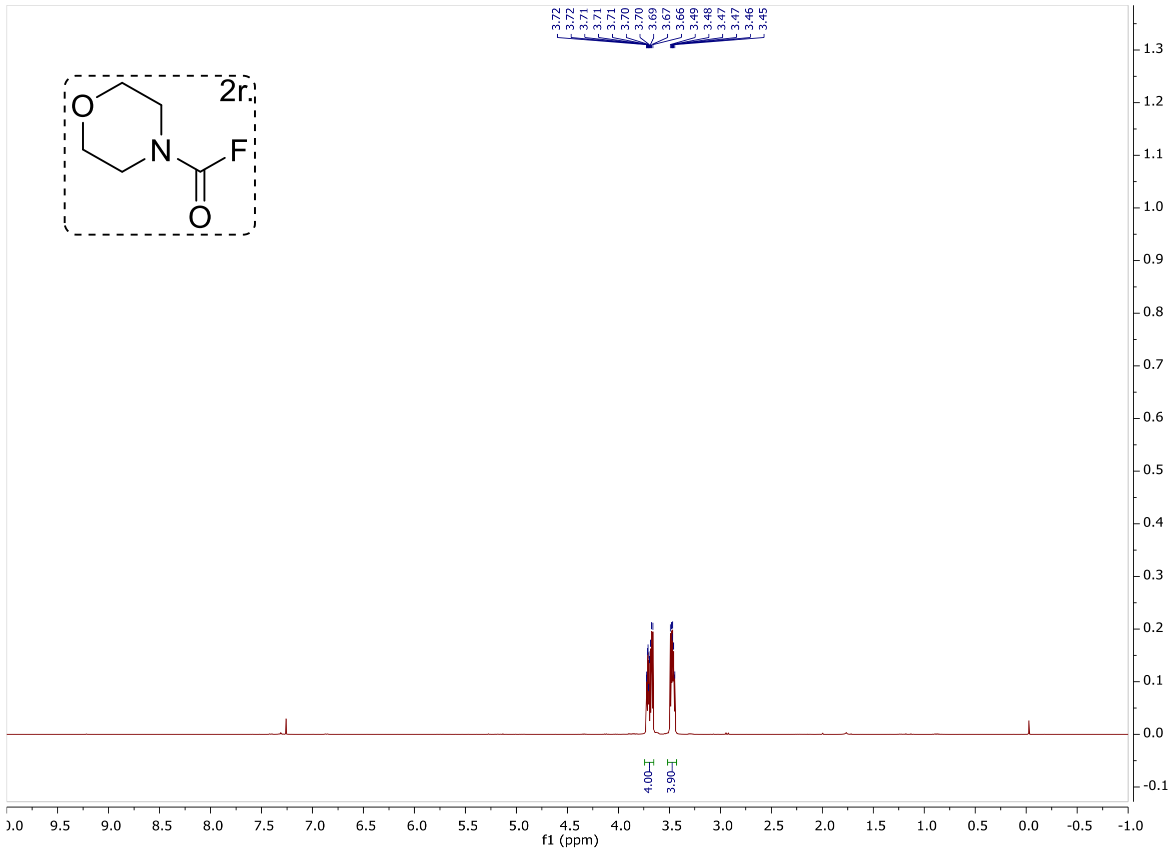

^{1}H NMR of 2r in $CDCl_3$ (400 MHz)

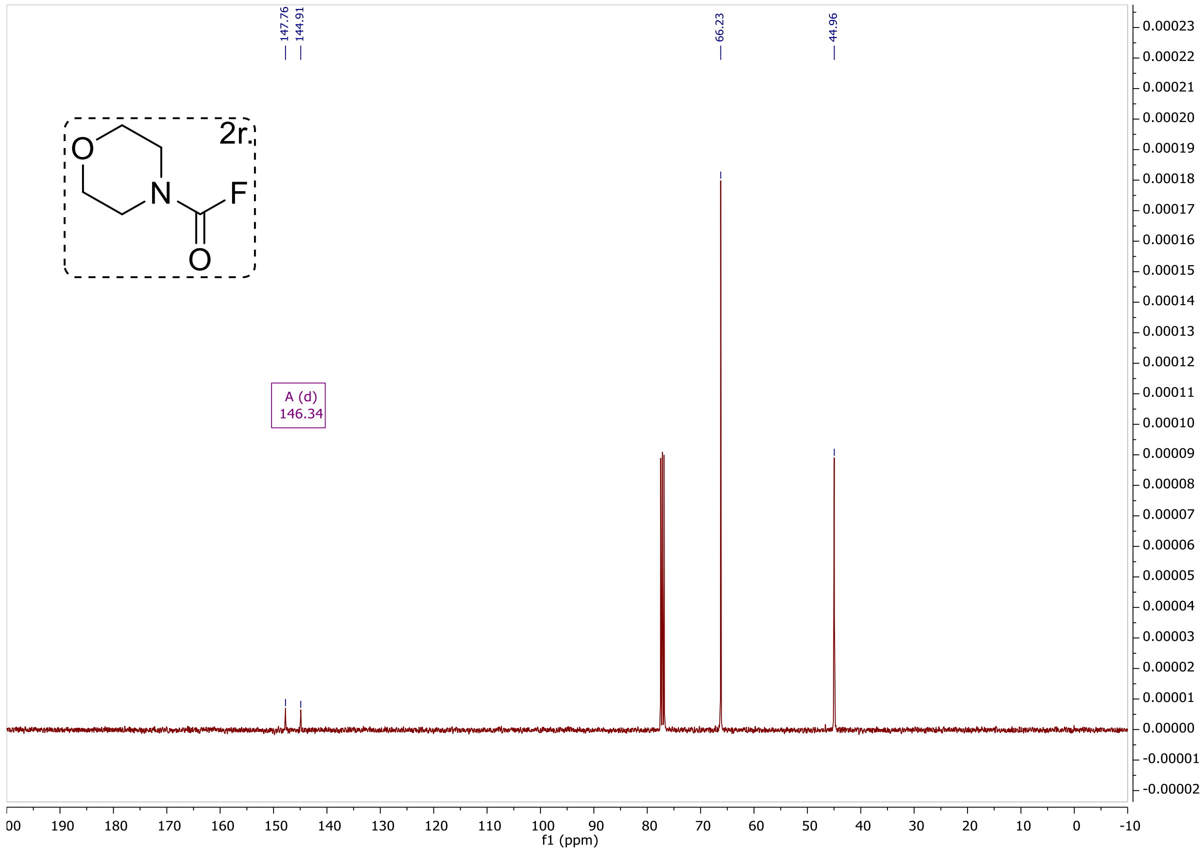

^{13}C NMR of 2r in $CDCl_3$ (101 MHz)

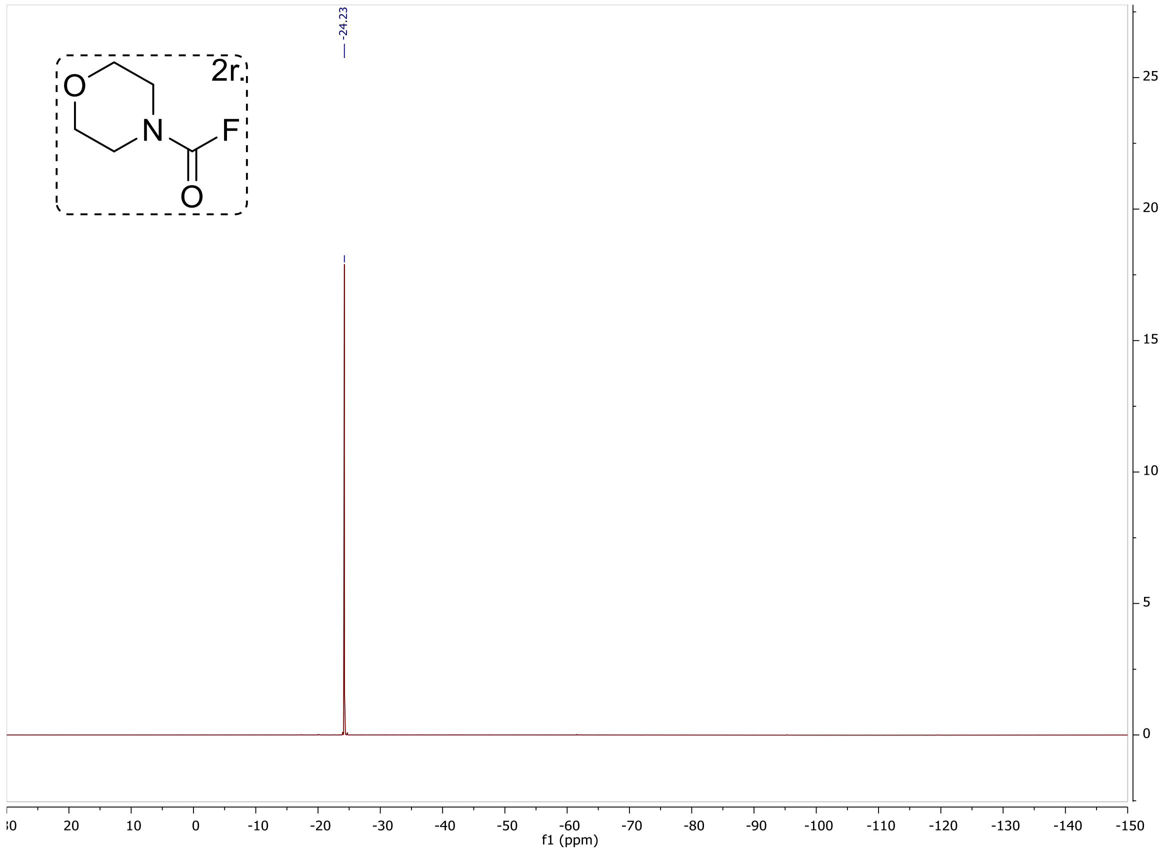

^{19}F NMR of 2r in $CDCl_3$ (376 MHz)

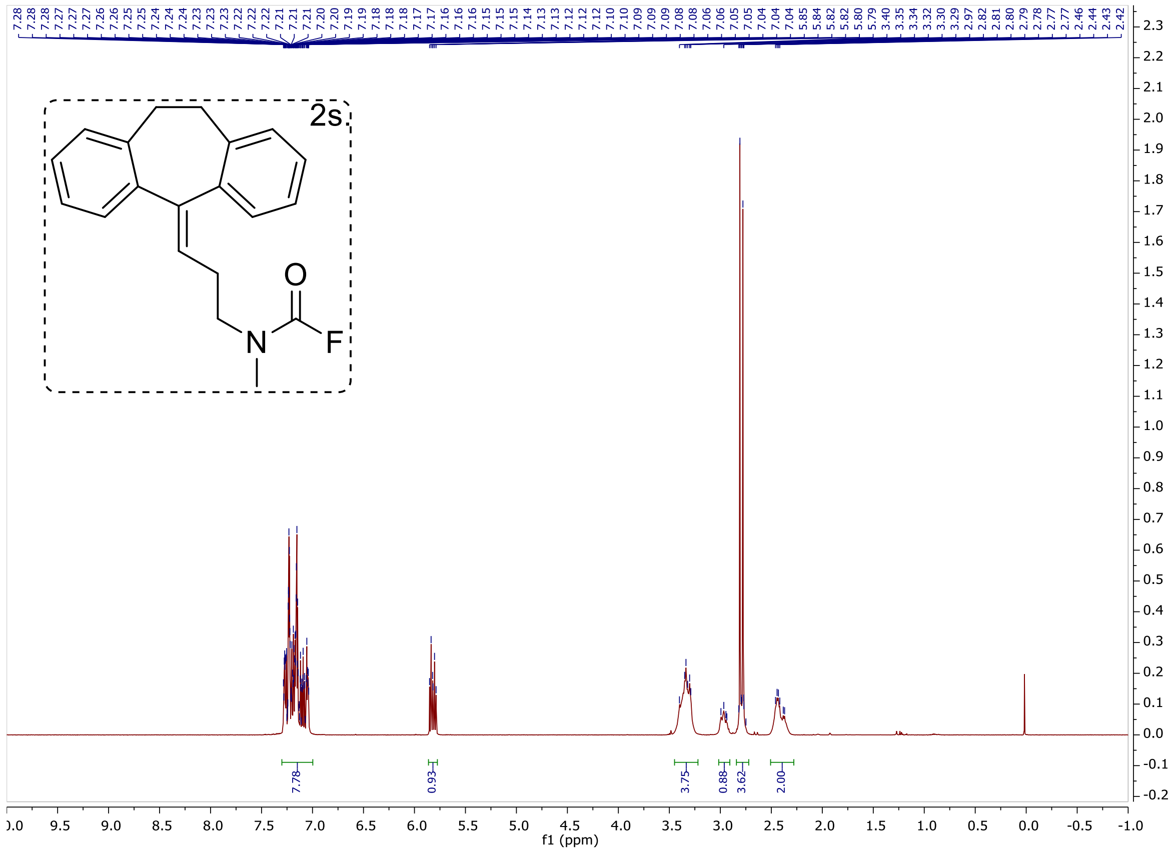

^{1}H NMR of 2s in $CDCl_3$ (500 MHz)

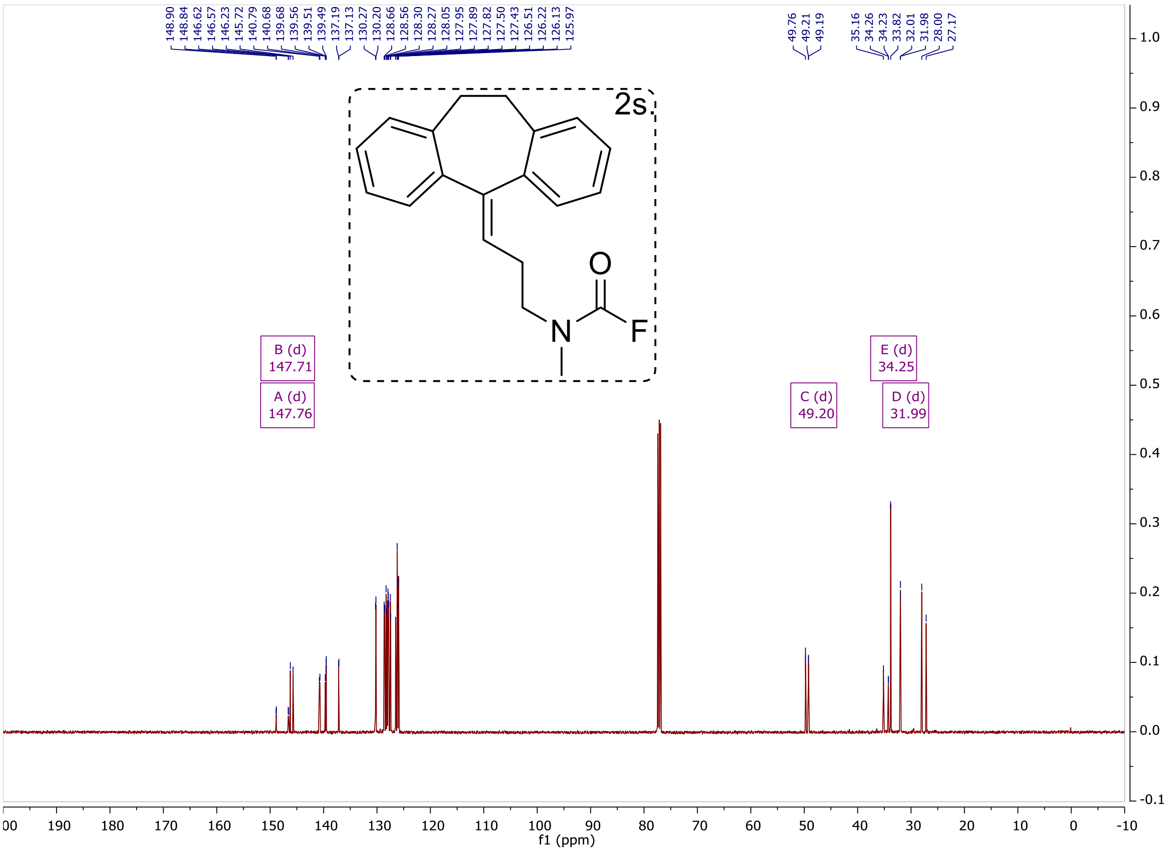

^{13}C NMR of 2s in $CDCl_3$ (126 MHz)

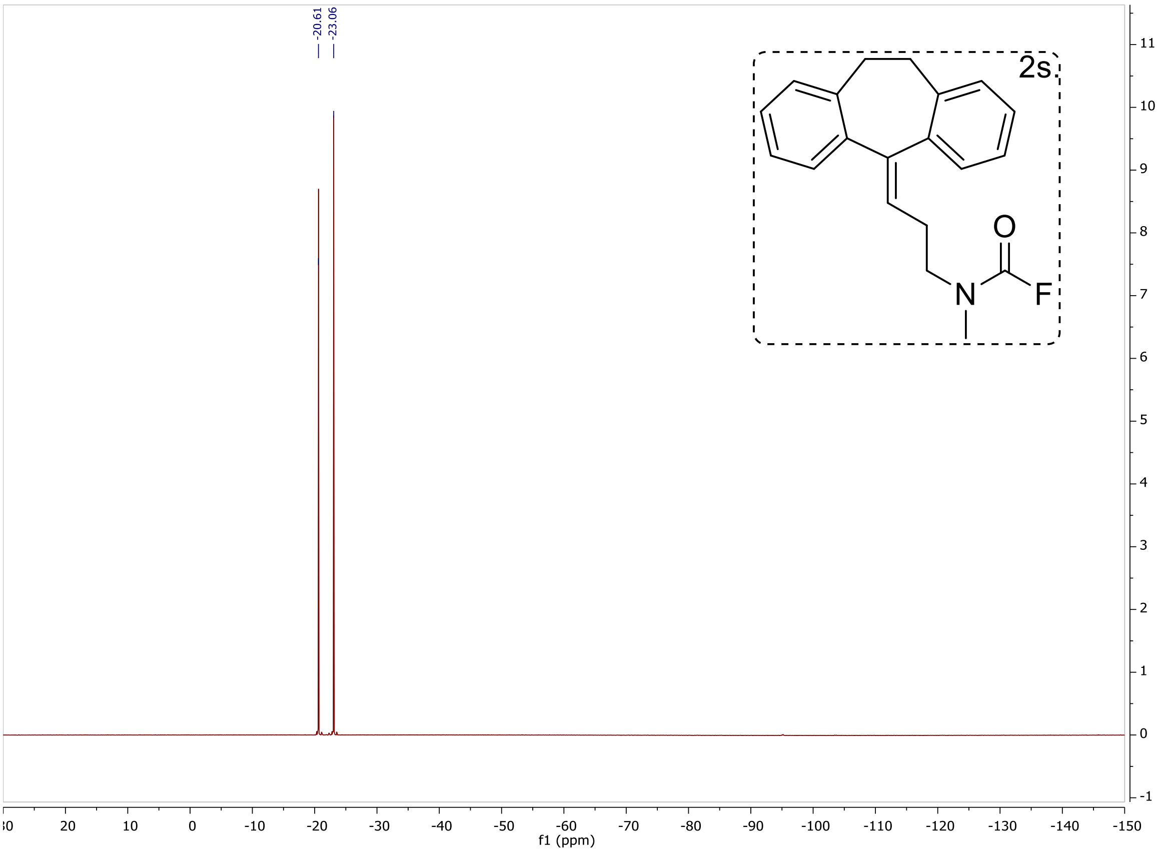

^{19}F NMR of 2s in $CDCl_3$ (376 MHz)

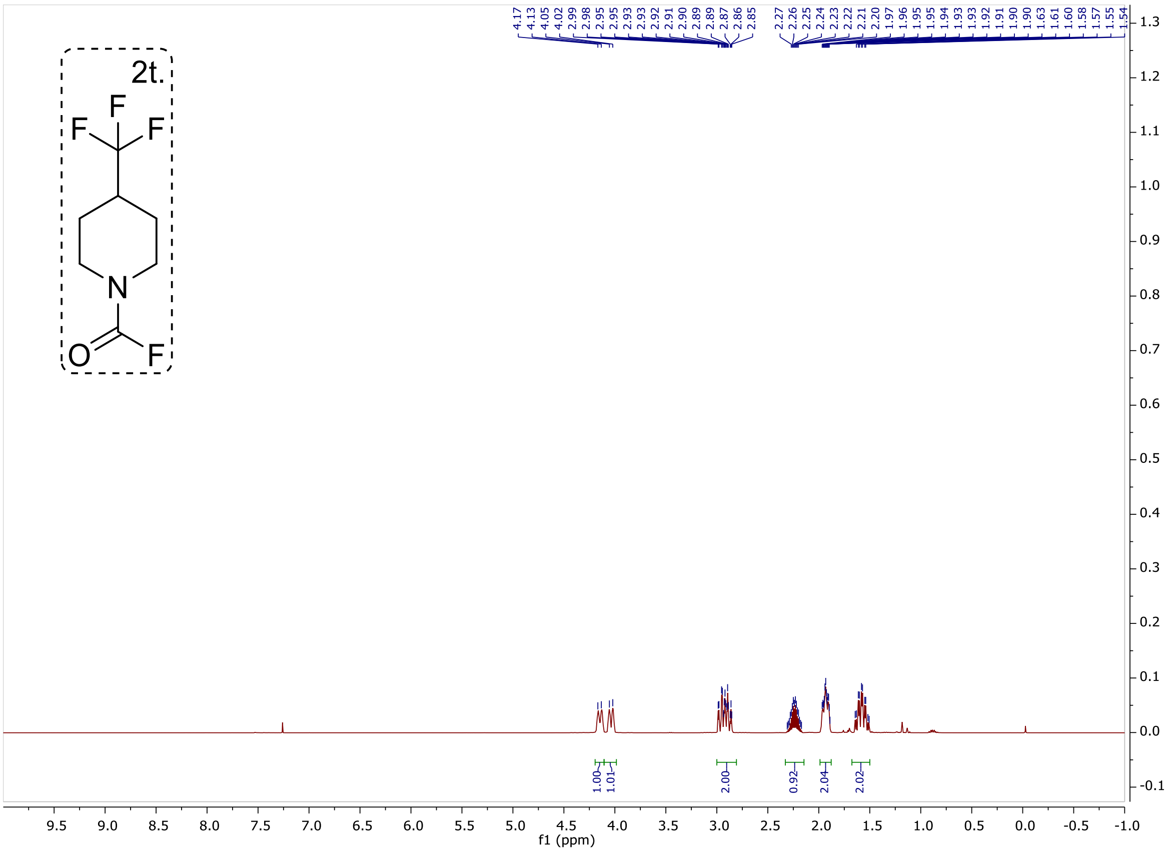

^{1}H NMR of 2t in $CDCl_3$ (400 MHz)

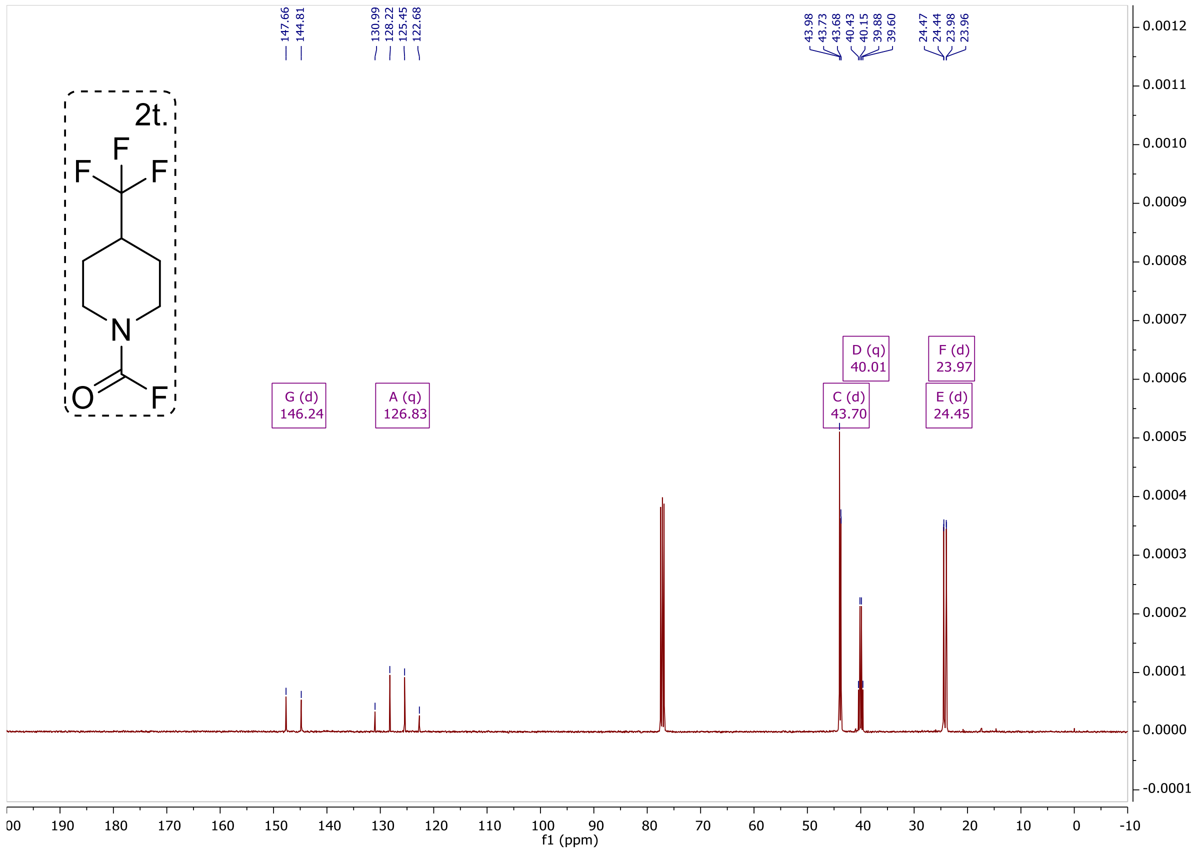

^{13}C NMR of 2t in $CDCl_3$ (101 MHz)

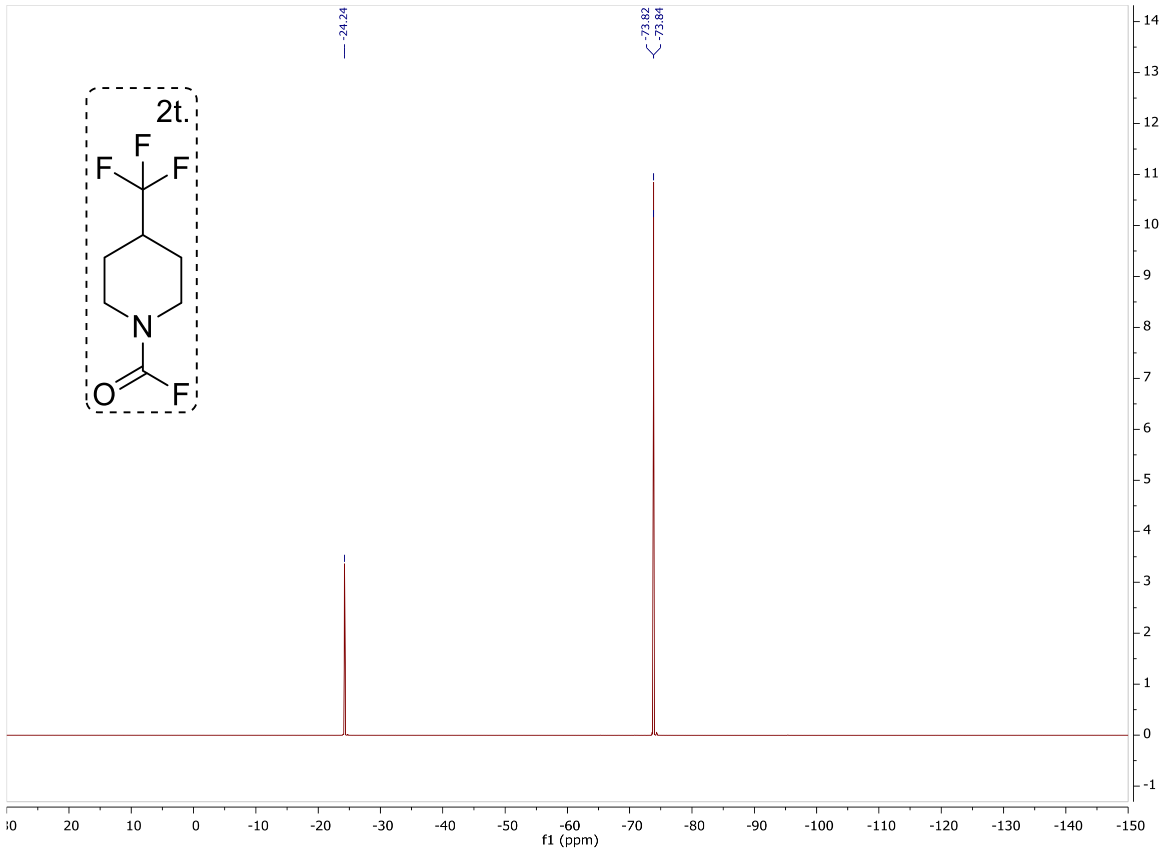

^{19}F NMR of 2t in $CDCl_3$ (376 MHz)

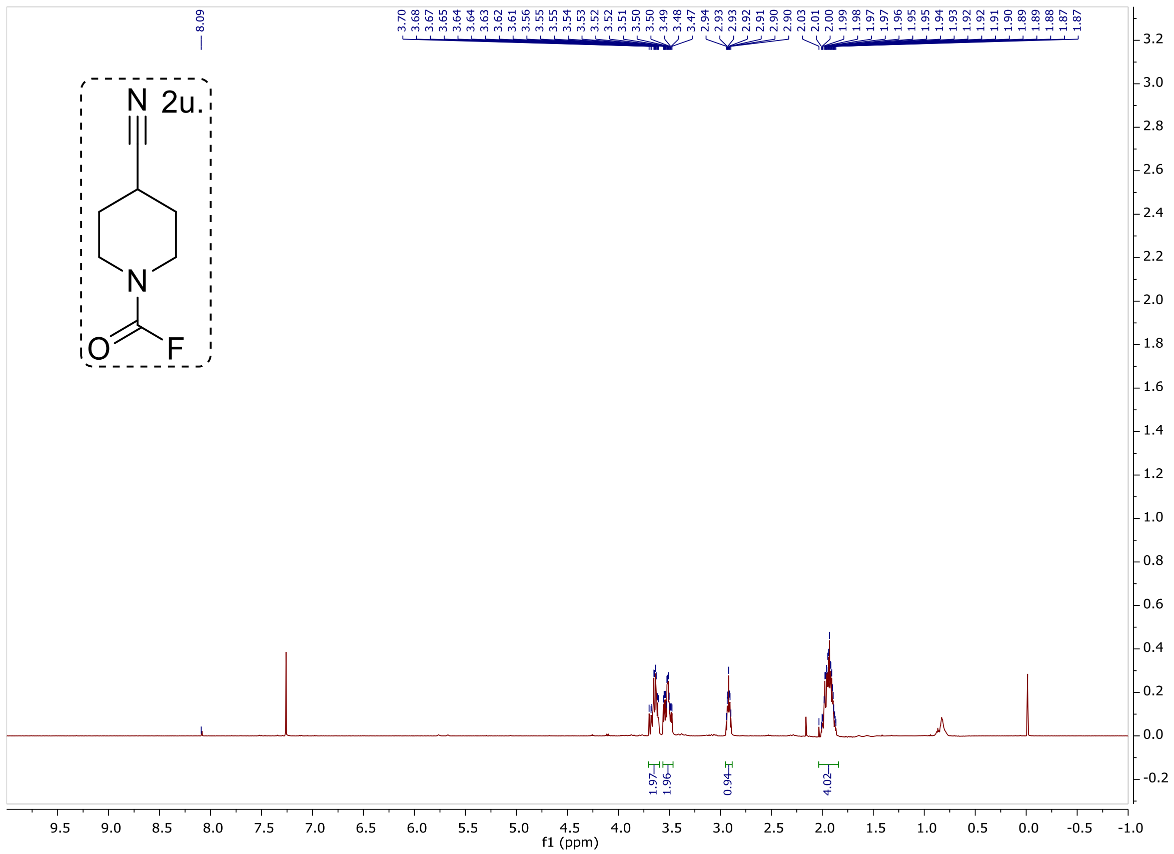

^{1}H NMR of 2u in $CDCl_3$ (500 MHz)

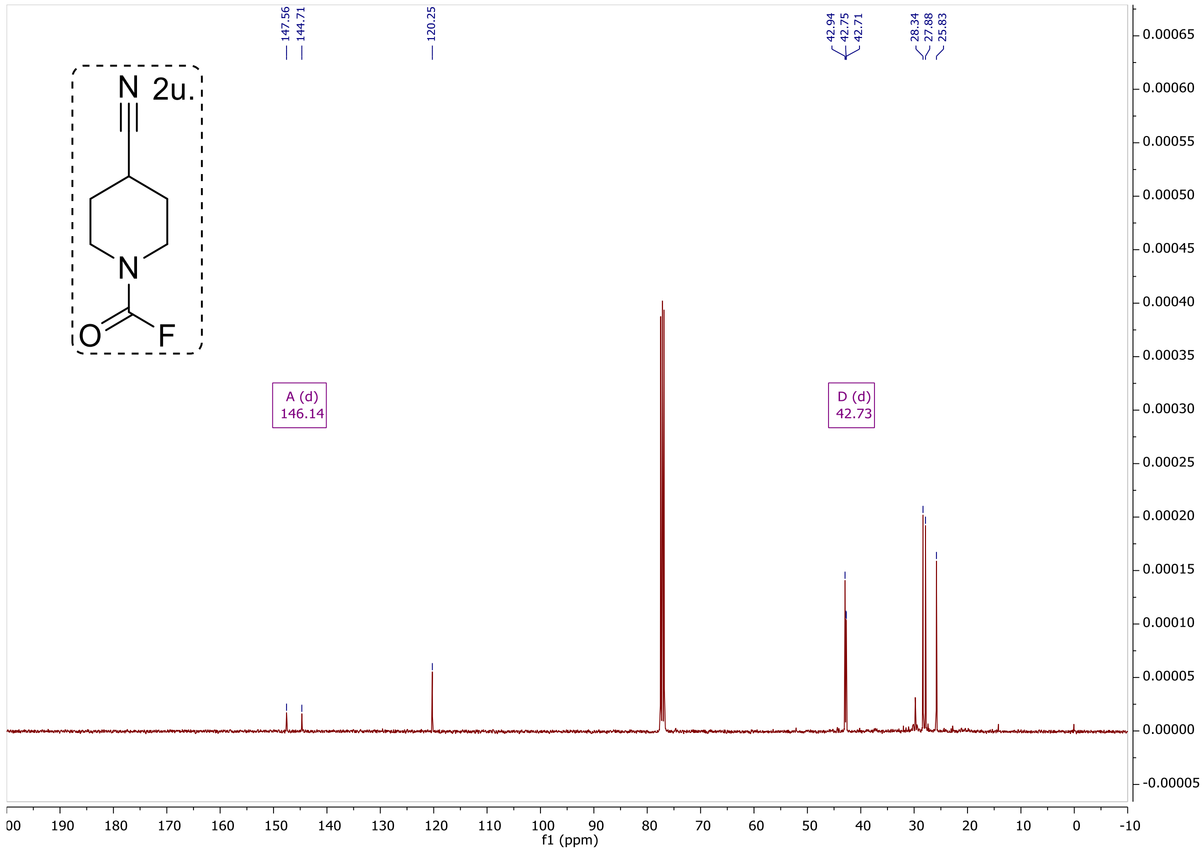

^{13}C NMR of 2u in $CDCl_3$ (101 MHz)

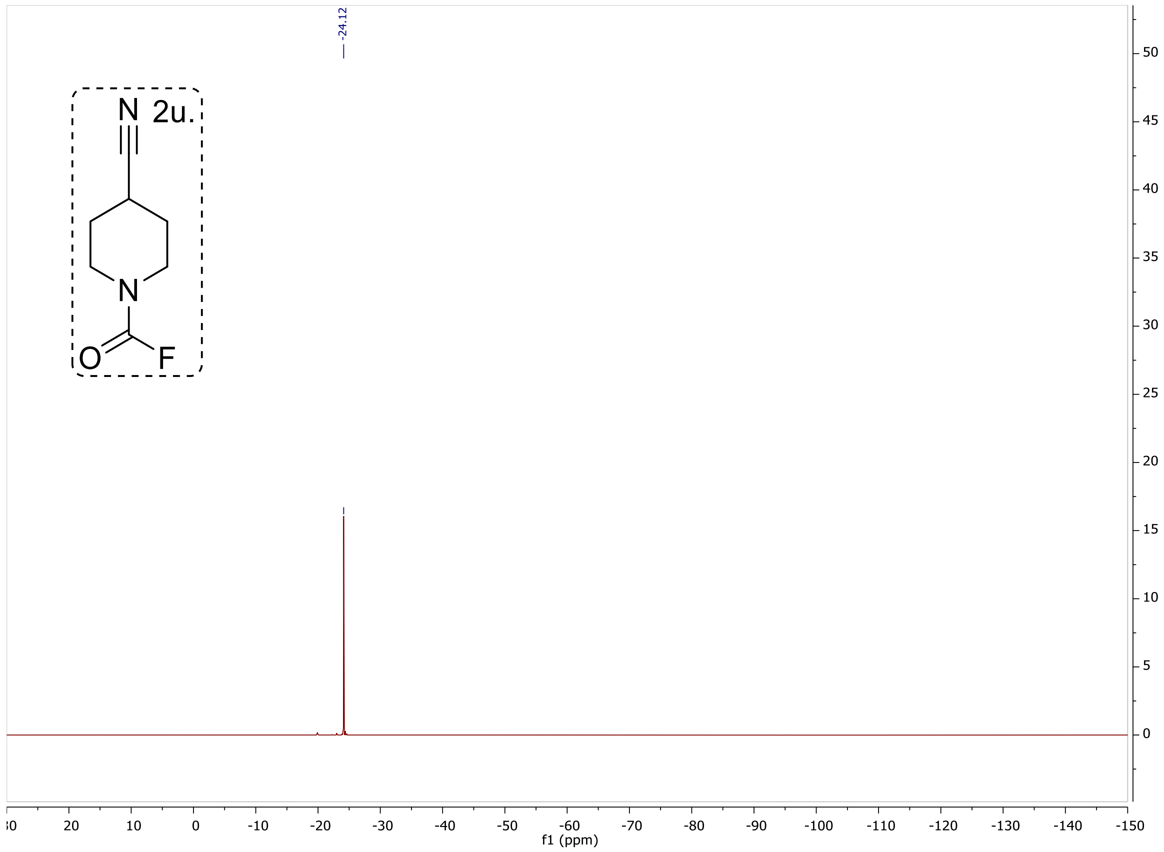

^{19}F NMR of 2u in $CDCl_3$ (376 MHz)

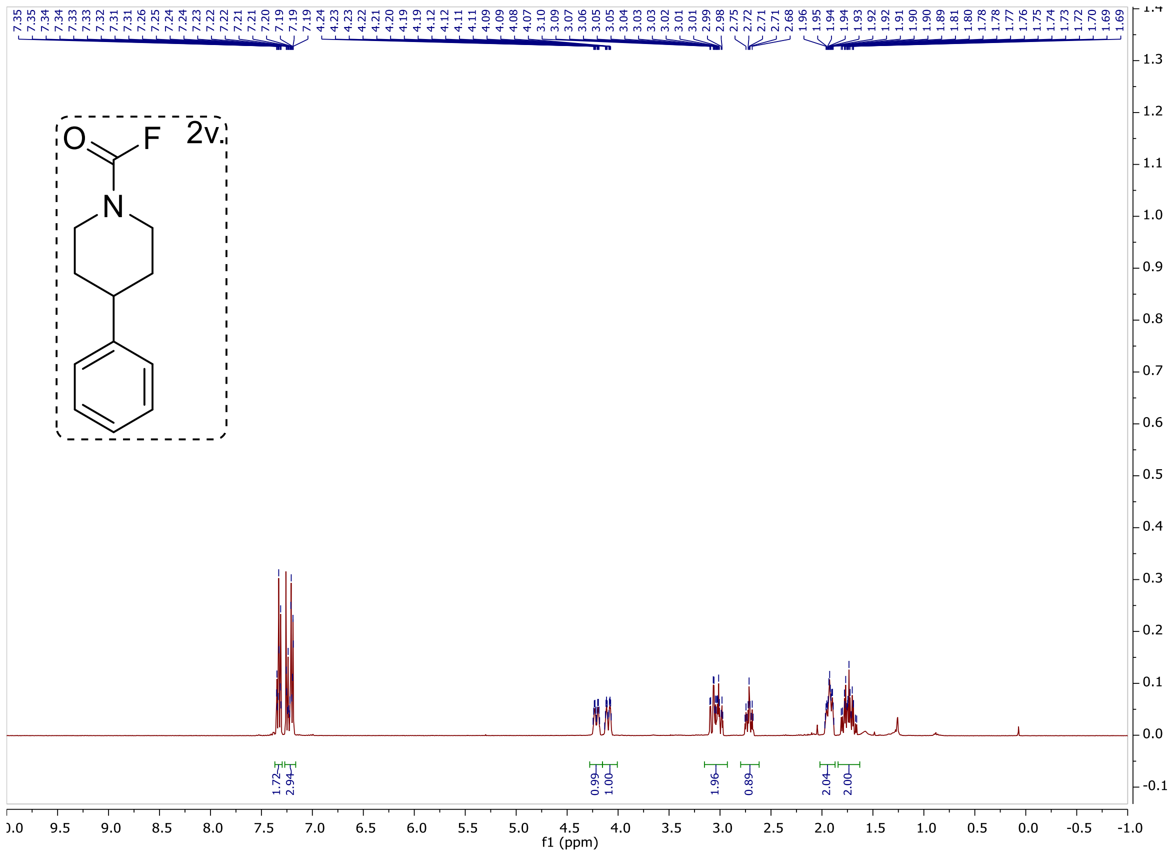

^{1}H NMR of 2v in $CDCl_3$ (400 MHz)

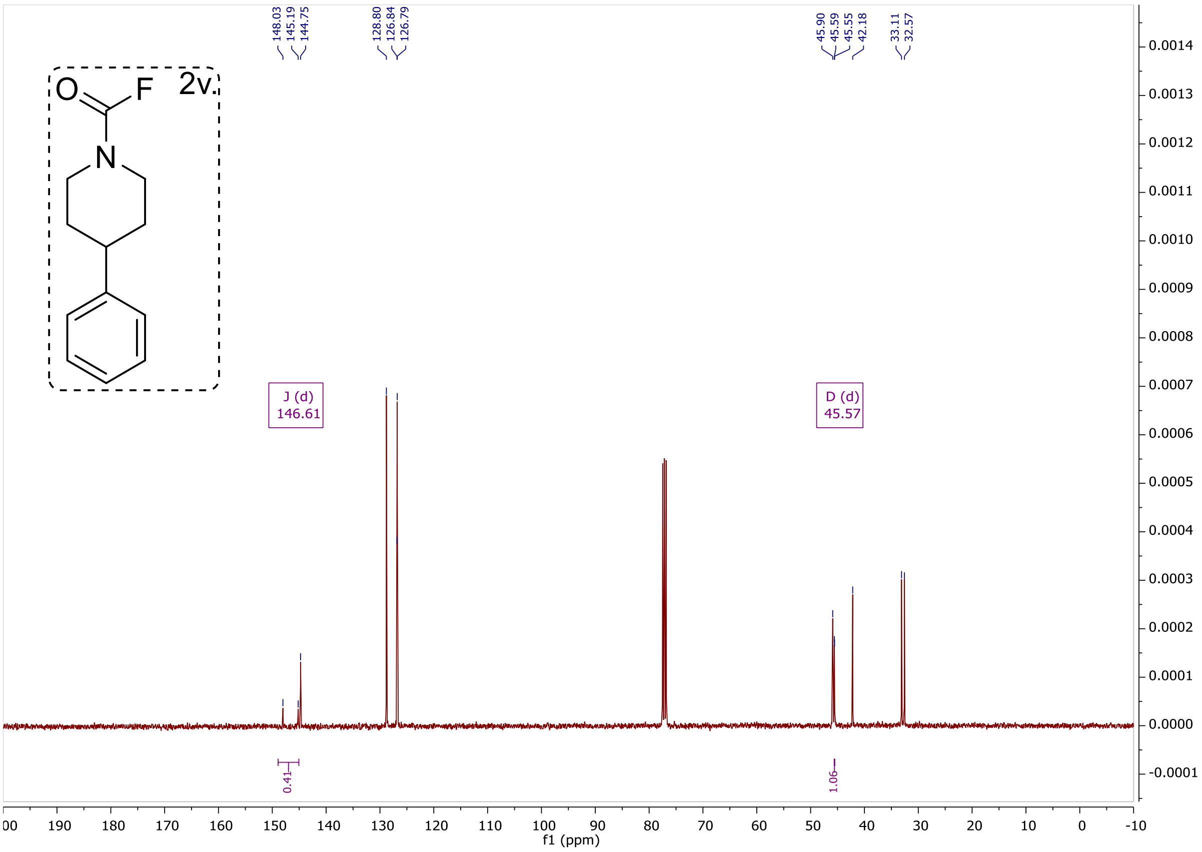

^{13}C NMR of 2v in $CDCl_3$ (101 MHz)

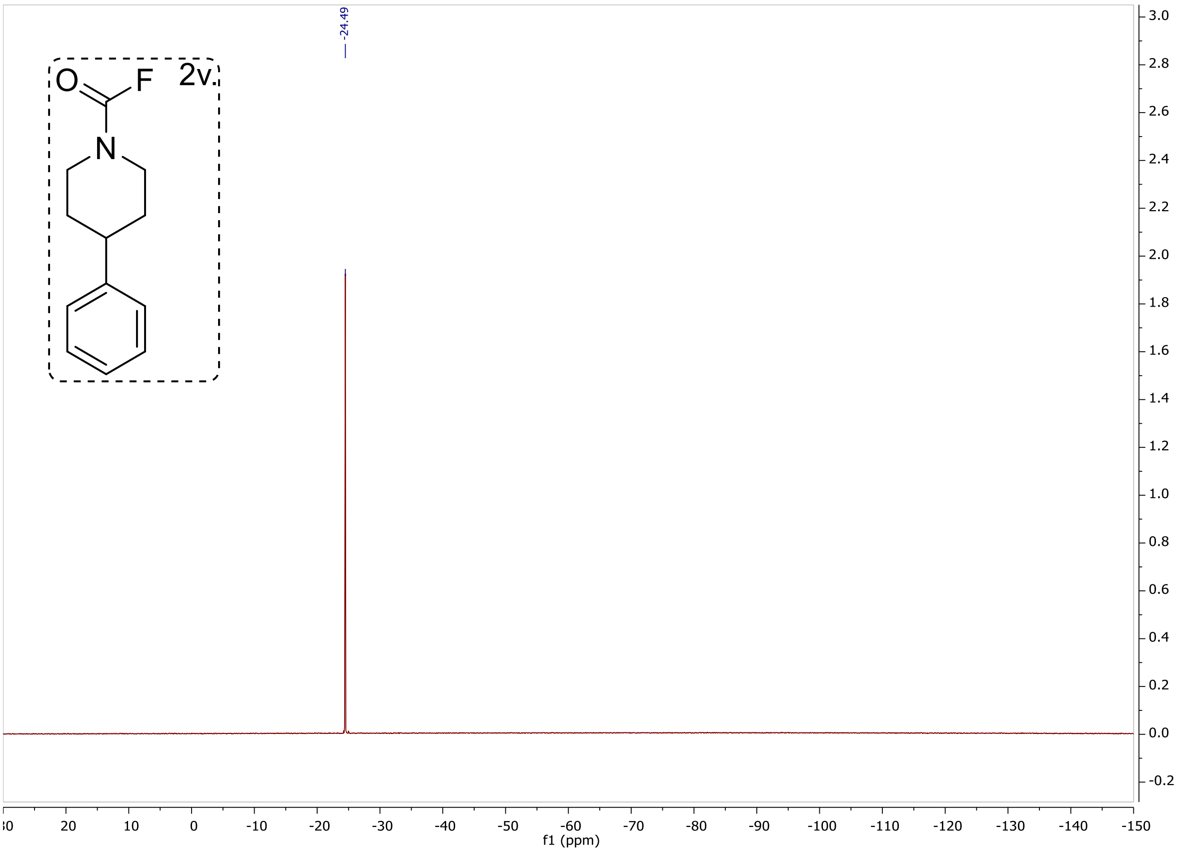

^{19}F NMR of 2v in $CDCl_3$ (376 MHz)

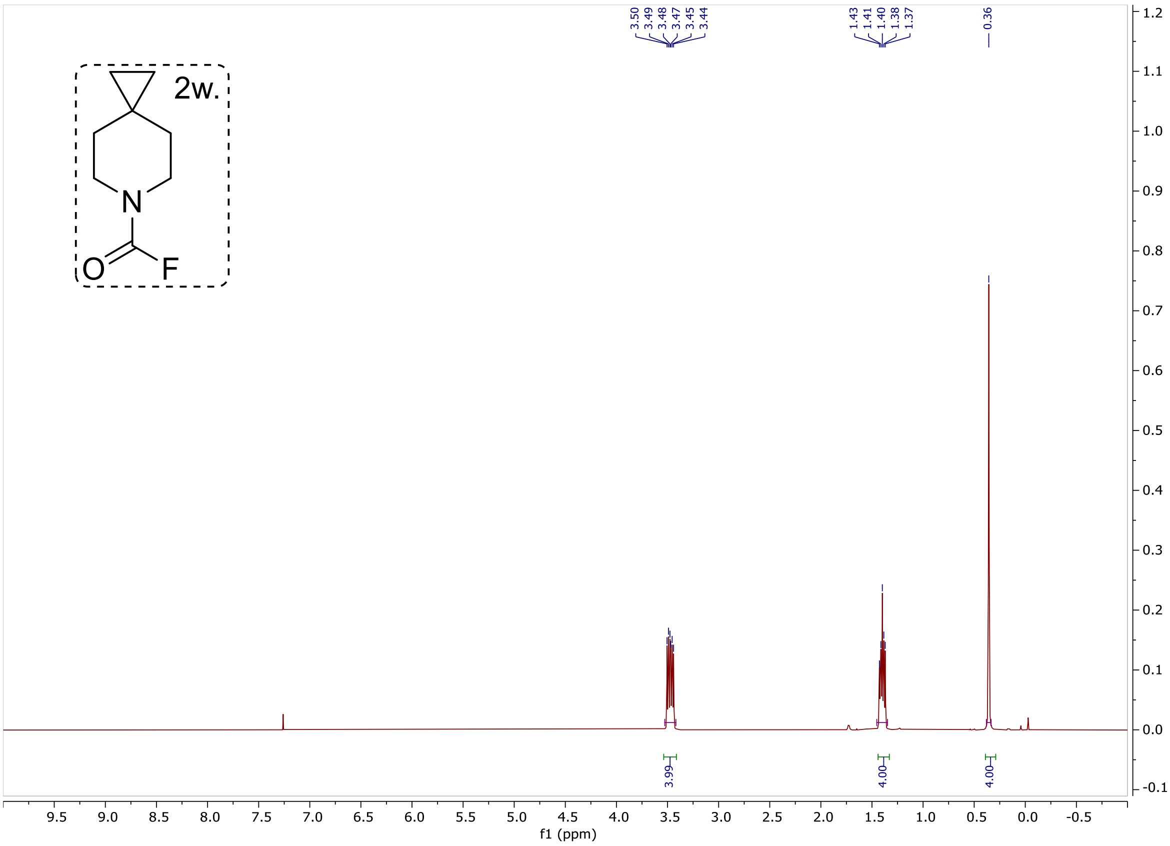

^{1}H NMR of 2w in $CDCl_3$ (400 MHz)

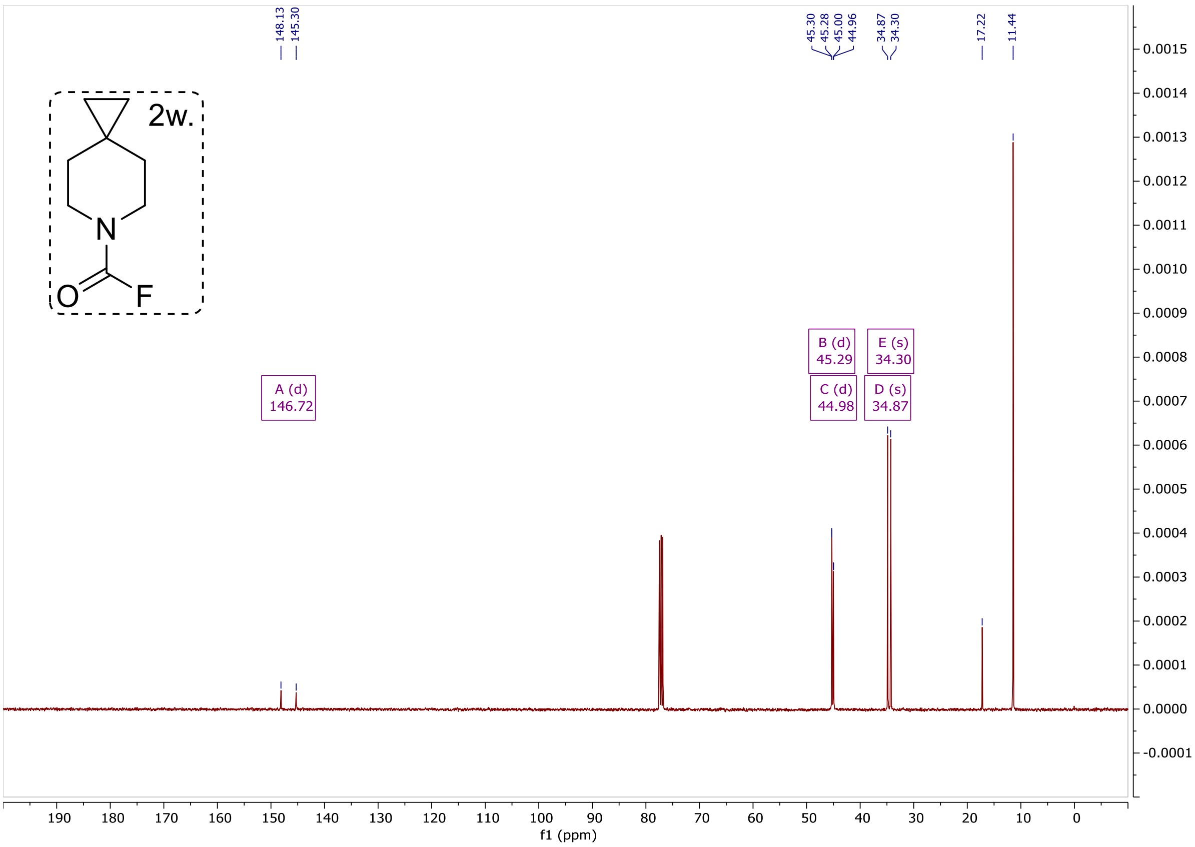

^{13}C NMR of 2w in $CDCl_3$ (101 MHz)

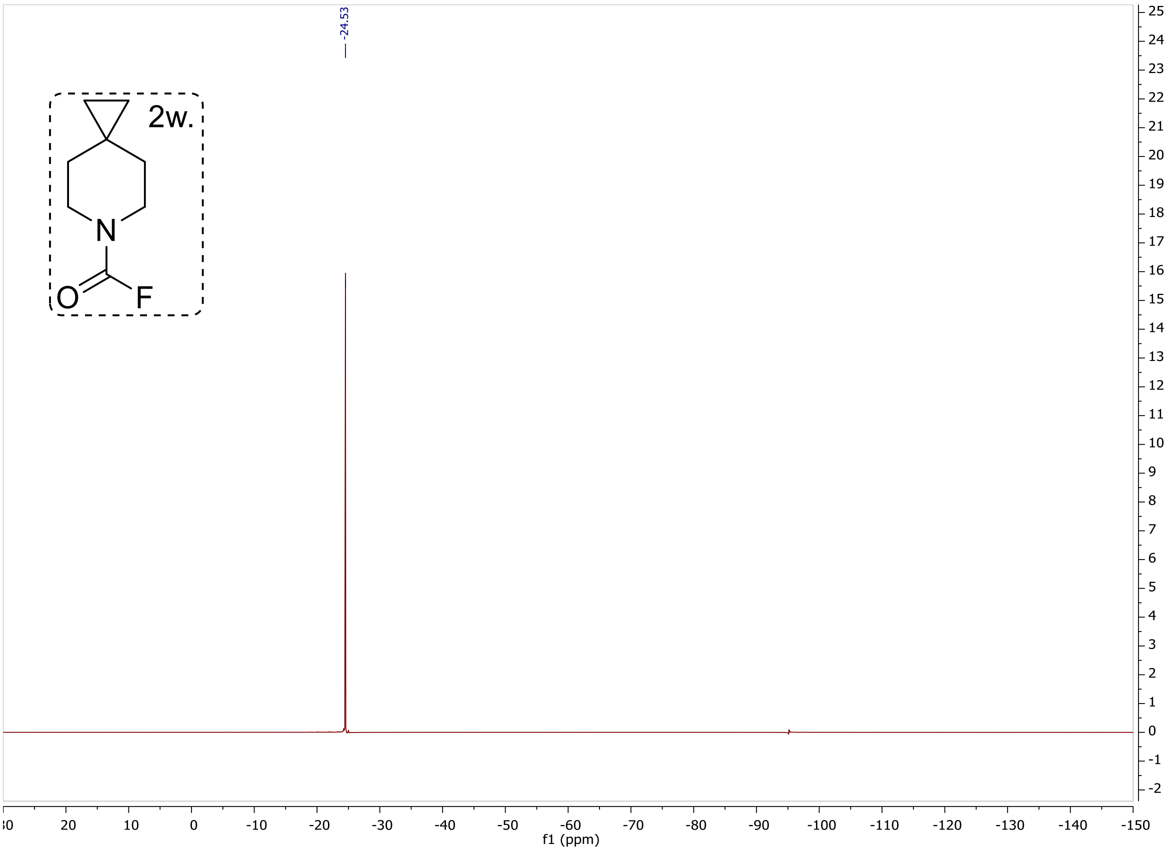

^{19}F NMR of 2w in $CDCl_3$ (376 MHz)

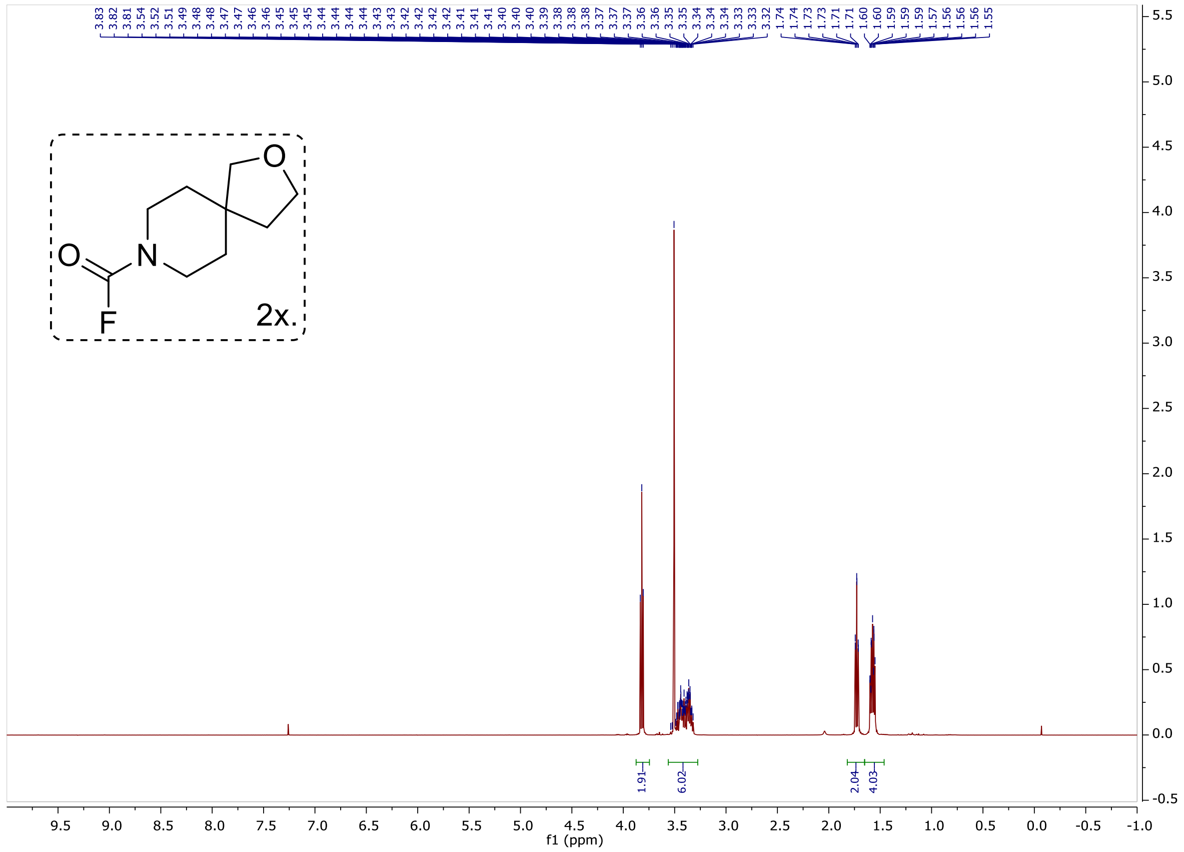

^{1}H NMR of 2x in $CDCl_3$ (400 MHz)

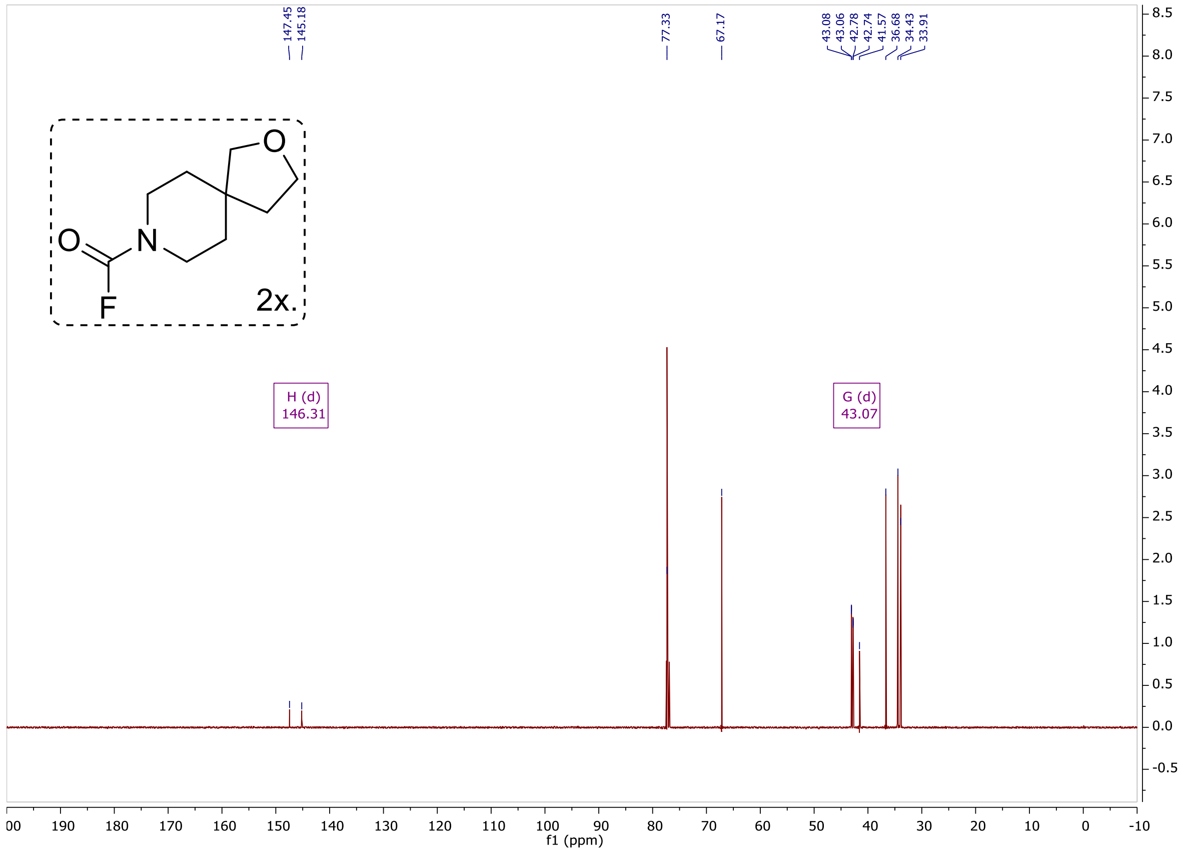

^{13}C NMR of 2x in $CDCl_3$ (126 MHz)

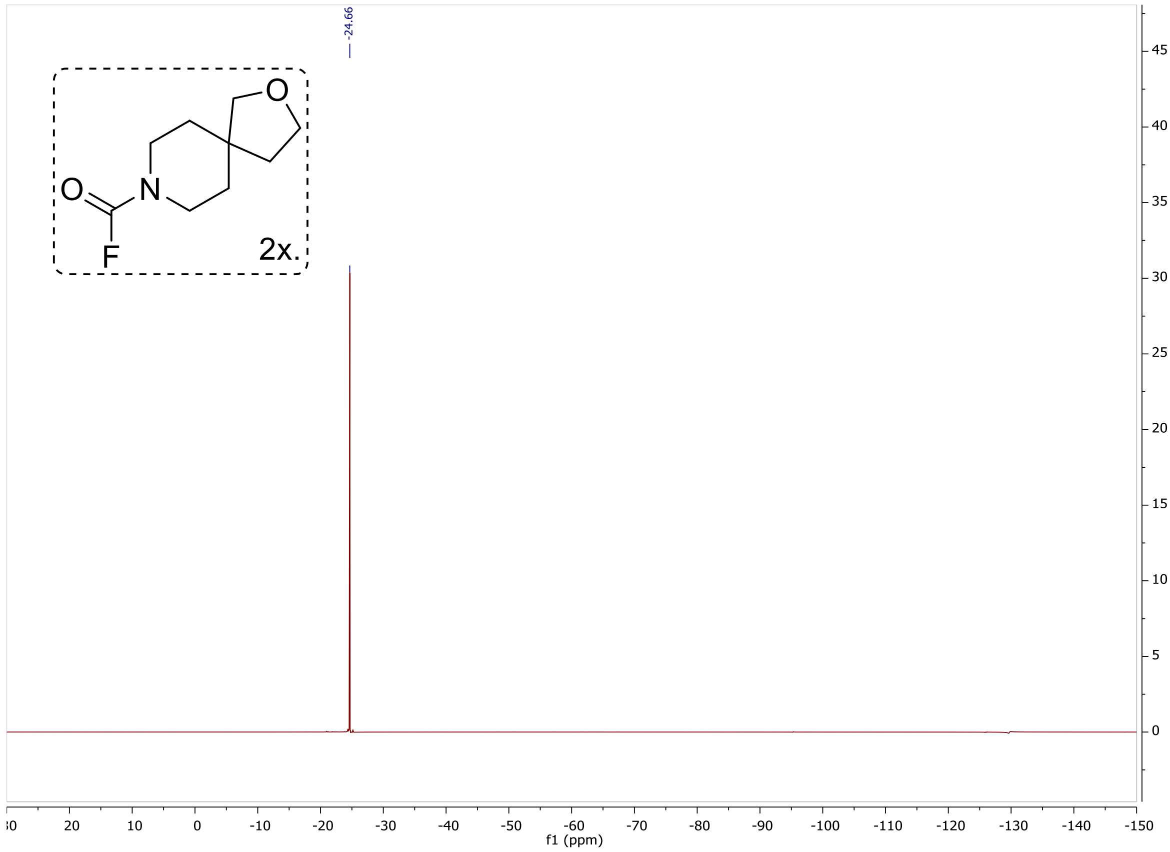

^{19}F NMR of 2x in $CDCl_3$ (376 MHz)

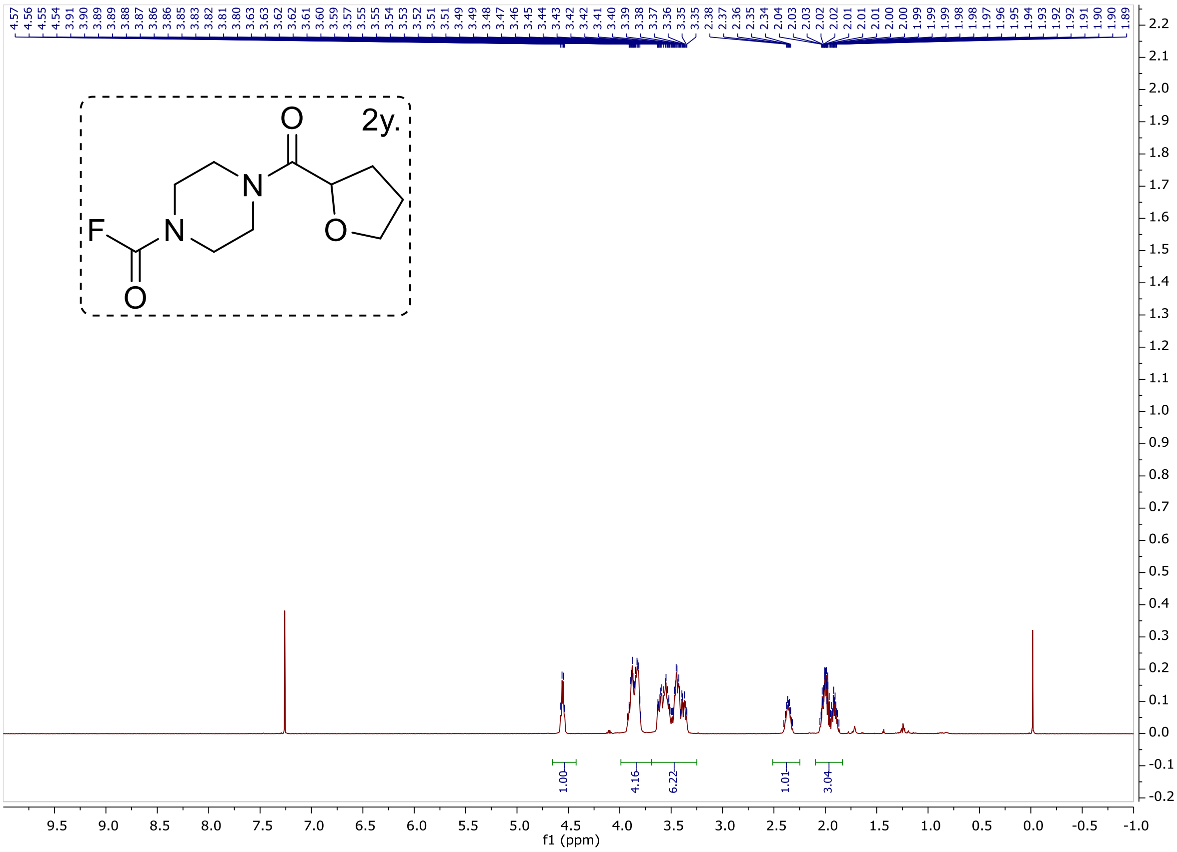

^{1}H NMR of 2y in $CDCl_3$ (500 MHz)

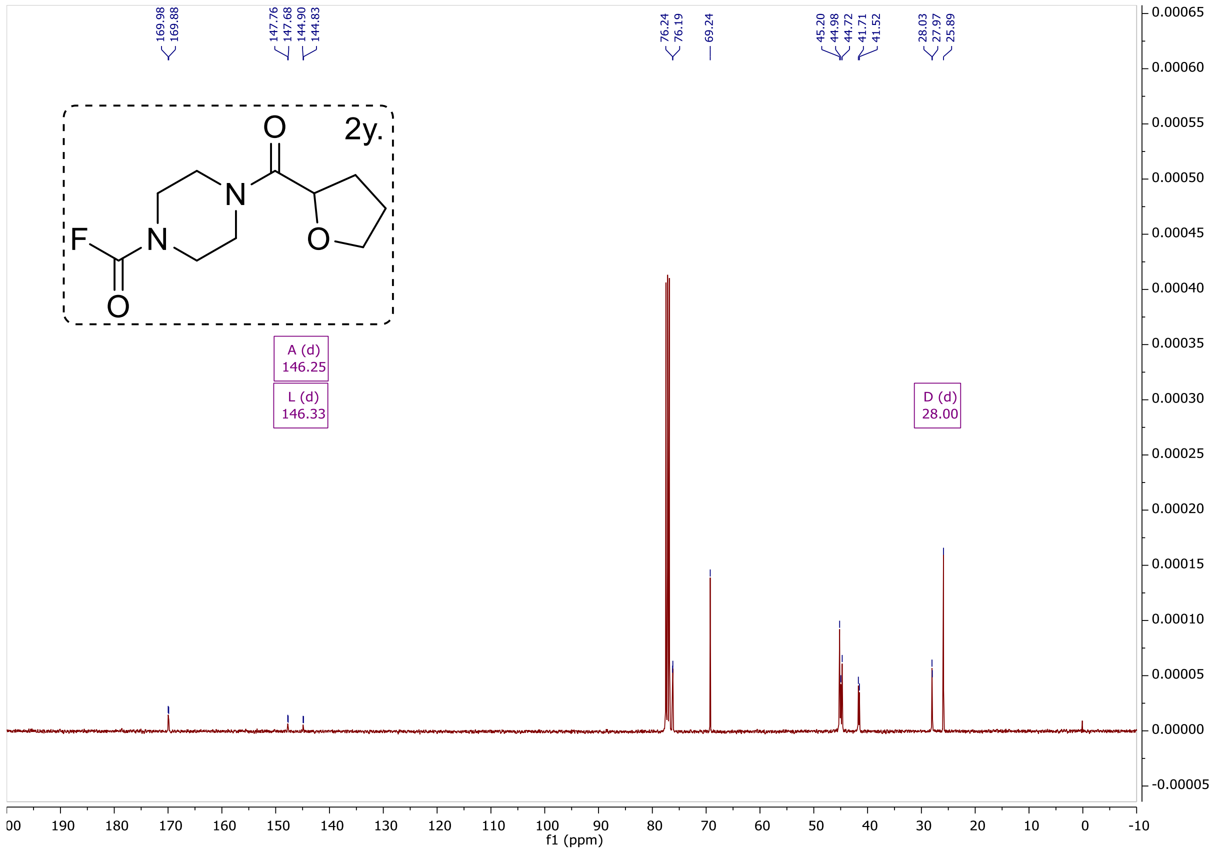

^{13}C NMR of 2y in $CDCl_3$ (101 MHz)

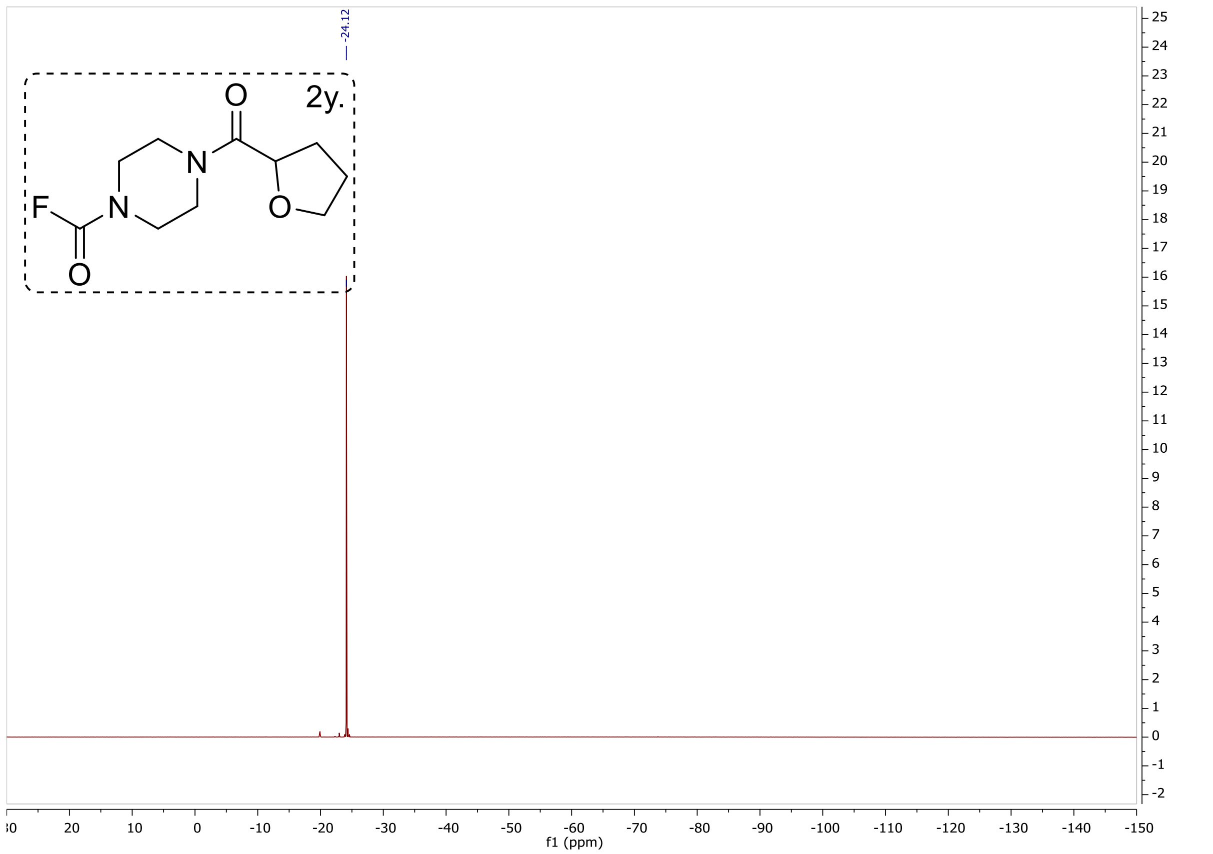

^{19}F NMR of 2y in $CDCl_3$ (376 MHz)

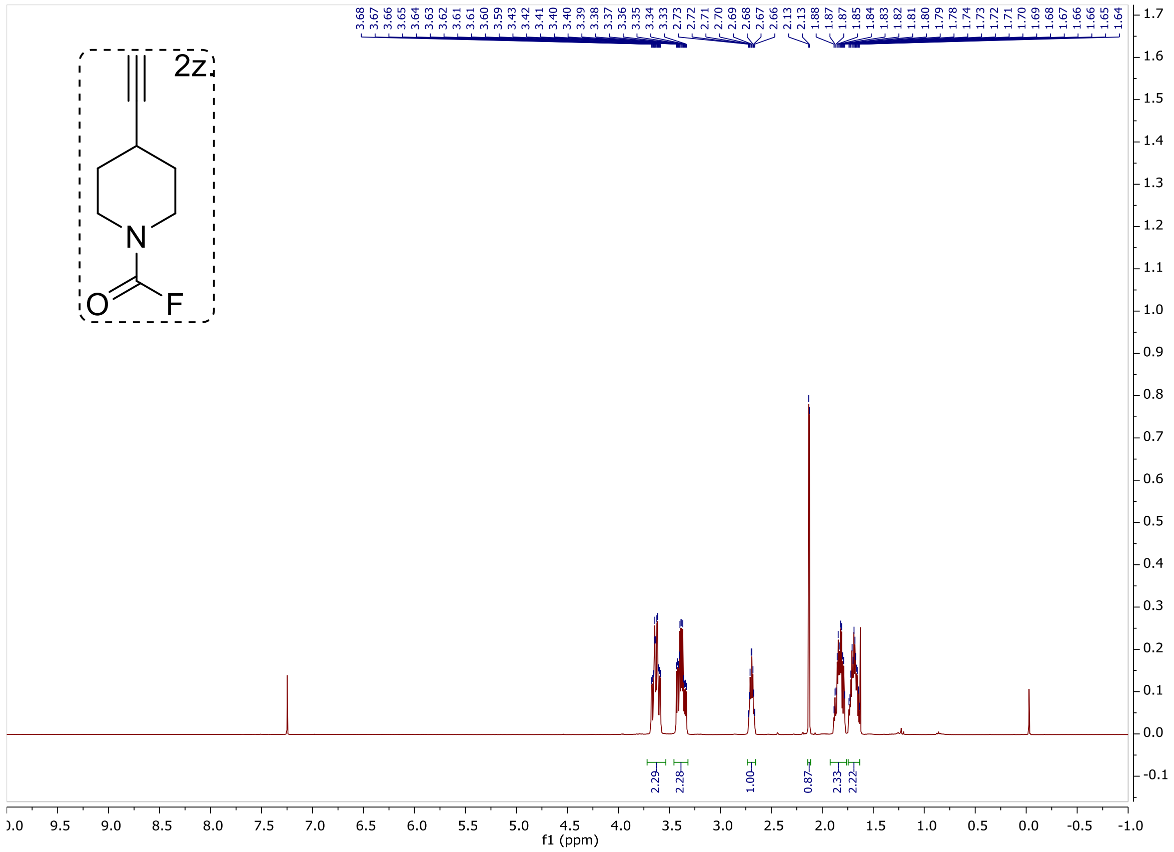

^{1}H NMR of 2z in $CDCl_3$ (400 MHz)

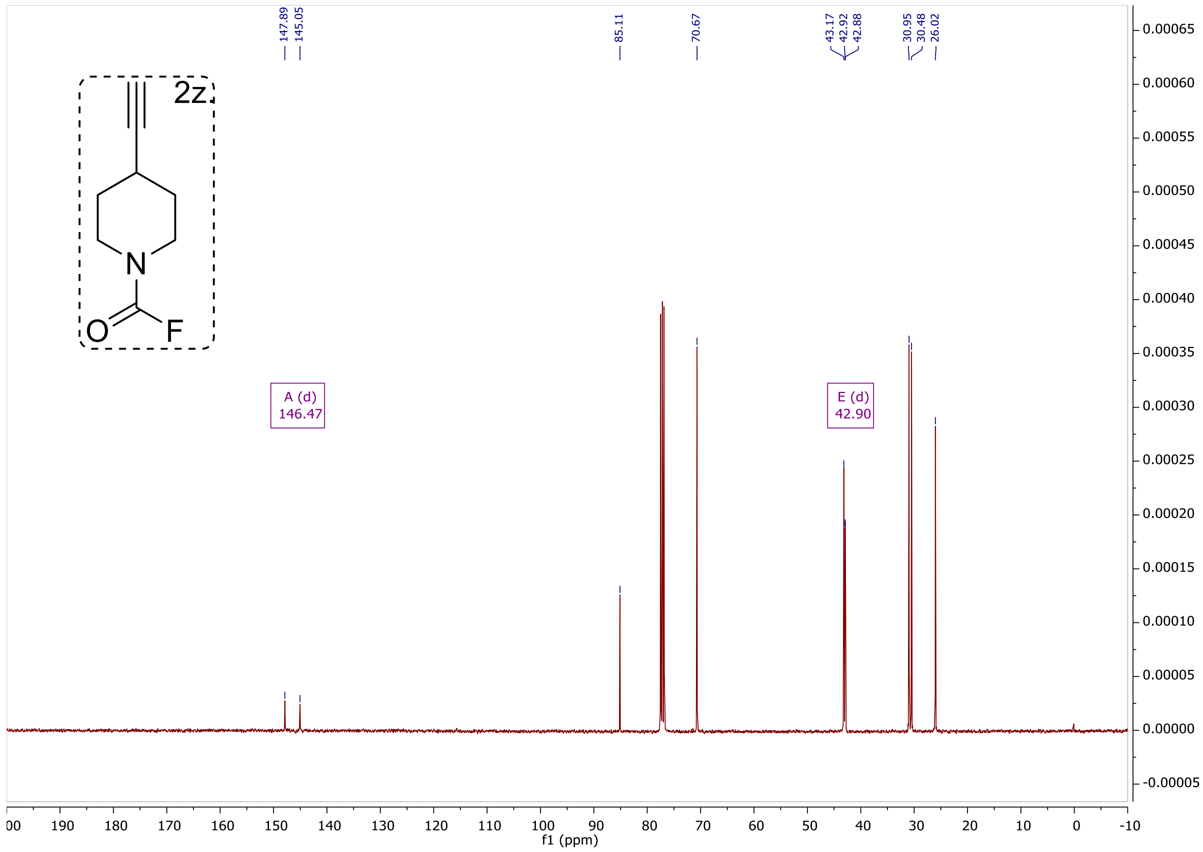

^{13}C NMR of 2z in $CDCl_3$ (101 MHz)

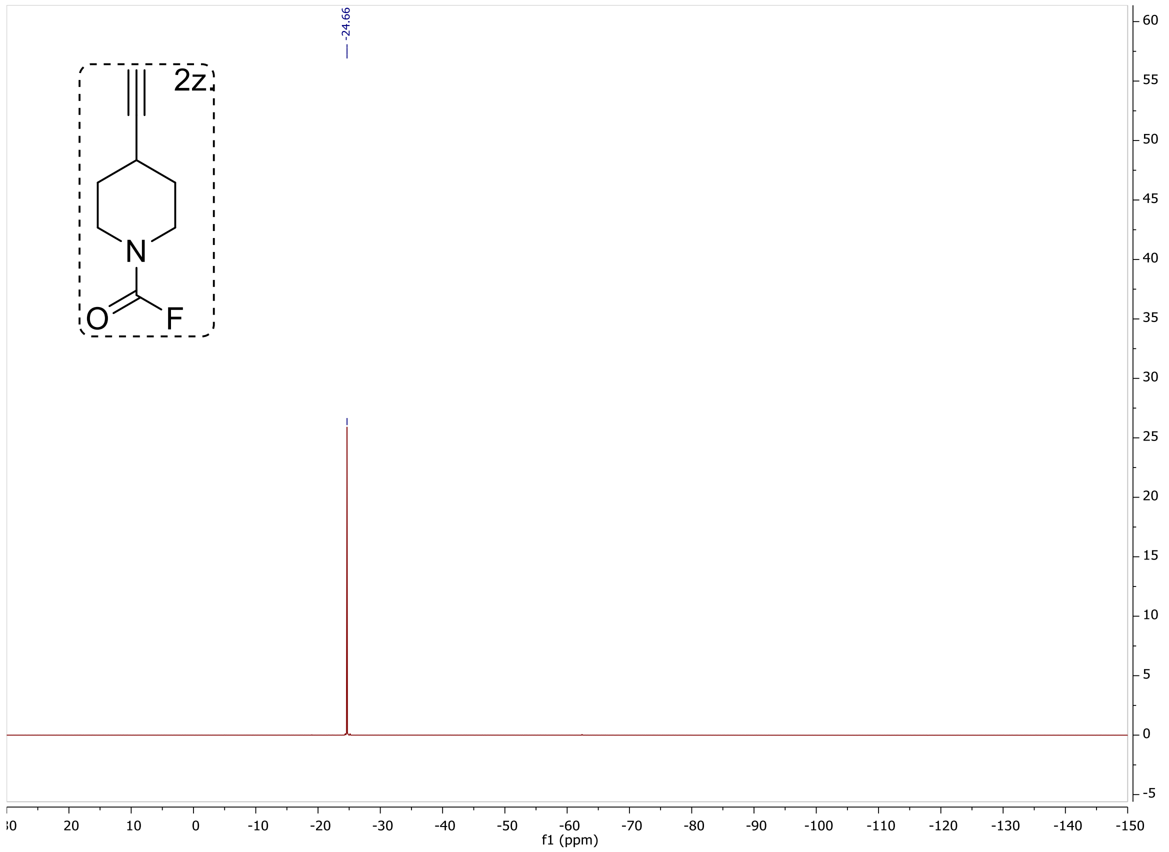

^{19}F NMR of 2z in $CDCl_3$ (376 MHz)

Bibliography

(1) Cadwallader, D.; Tiburcio, T. R.; Cieszynski, G. A.; Le, C. M. Synthesis of Carbamoyl Fluorides Using a Difluorophosgene Surrogate Derived from Difluorocarbene and Pyridine N-Oxides. *J. Org. Chem* **2022,** 87, 11457-11468.
(2) Buzzetti, L.; Puriņš, M.; Greenwood, P. D. G.; Waser, J. Enantioselective Carboetherification/Hydrogenation for the Synthesis of Amino Alcohols Via a Catalytically Formed Chiral Auxiliary. *J. Am. Chem. Soc.* **2020,** 142, 17334-17339.
(3) Hutskalova, V.; Bou Hamdan, F.; Sparr, C. Decarboxylative Nickel- and Photoredox-Catalyzed Aminocarbonylation of (Hetero)Aryl Bromides. *Org. Lett.* **2023**.
(4) Bonnefoy, C.; Chefdeville, E.; Tourvieille, C.; Panossian, A.; Hanquet, G.; Leroux, F.; Toulgoat, F.; Billard, T. Study of Carbamoyl Fluoride: Synthesis, Properties and Applications. *Chem. Eur. J.* **2022,** 28, e202201589.
(5) Turksoy, A.; Scattolin, T.; Bouayad-Gervais, S.; Schoenebeck, F. Facile Access to Agocf$_3$ and Its New Applications as a Reservoir for Ocf$_2$ for the Direct Synthesis of N−Cf$_3$, Aryl or Alkyl Carbamoyl Fluorides. *Chem. Eur. J.* **2020,** 26, 2183-2186.
(6) Tang, H.-J.; Shi, X.-M.; Zhu, X.-Y.; Wang, C.-Q.; Feng, C. Oxidation of Difluorocarbene by Pyridine N-Oxide and Ensuing Access to Carbamoyl Fluorides†. *Chin. J. Chem.* **2023,** 41, 2981-2987.
